# Functional Assessment of Genetic Variants with Outcomes Adapted to Clinical Decision-Making

**DOI:** 10.1371/journal.pgen.1006096

**Published:** 2016-06-06

**Authors:** Pierre Thouvenot, Barbara Ben Yamin, Lou Fourrière, Aurianne Lescure, Thomas Boudier, Elaine Del Nery, Anne Chauchereau, David E. Goldgar, Claude Houdayer, Dominique Stoppa-Lyonnet, Alain Nicolas, Gaël A. Millot

**Affiliations:** 1 Institut Curie, PSL Research University, Paris, France; 2 CNRS UMR 3244, Paris, France; 3 Sorbonne Universités, UPMC Univ Paris 06, Paris, France; 4 Institut Curie, PSL Research University, Department of Translational Research, the Biophenics High-Content Screening Laboratory, Cell and Tissue Imaging Facility (PICT-IBiSA), Paris, France; 5 INSERM U981, Villejuif, France; 6 Univ Paris-Sud, UMR981, Villejuif, France; 7 Institut Gustave Roussy, Villejuif, France; 8 Department of Dermatology, University of Utah School of Medicine, Salt Lake City, Utah, United States of America; 9 Service de Génétique Oncologique, Institut Curie, Paris, France; 10 INSERM U830, Paris, France; 11 Université Paris Descartes, Paris, France; Cleveland Clinic Genomic Medicine Institute, UNITED STATES

## Abstract

Understanding the medical effect of an ever-growing number of human variants detected is a long term challenge in genetic counseling. Functional assays, based on *in vitro* or *in vivo* evaluations of the variant effects, provide essential information, but they require robust statistical validation, as well as adapted outputs, to be implemented in the clinical decision-making process. Here, we assessed 25 pathogenic and 15 neutral missense variants of the *BRCA1* breast/ovarian cancer susceptibility gene in four *BRCA1* functional assays. Next, we developed a novel approach that refines the variant ranking in these functional assays. Lastly, we developed a computational system that provides a probabilistic classification of variants, adapted to clinical interpretation. Using this system, the best functional assay exhibits a variant classification accuracy estimated at 93%. Additional theoretical simulations highlight the benefit of this ready-to-use system in the classification of variants after functional assessment, which should facilitate the consideration of functional evidences in the decision-making process after genetic testing. Finally, we demonstrate the versatility of the system with the classification of siRNAs tested for human cell growth inhibition in high throughput screening.

## Author Summary

Human genetics has entered a new age with the advent of next generation sequencing. However, this great advance also comes with new concerns. Currently, the extensive use of multi-gene panels, whole exome and whole genome sequencing, is generating an ever-growing number of new DNA sequence variations detected in the disease-predisposing genes of human patients. The pathogenic or neutral status of these variants needs to be known before planning any medical act or follow-up. We show here that the status of the variants identified in the BRCA1 breast/ovarian cancer susceptibility gene can be estimated thanks to experimental systems using yeast cells and a novel computational model. Importantly, this model provides a probabilistic classification of variants, opening the possibility to integrate results from functional assays into clinical decision-making. Moreover, our computational model is directly compatible with all kinds of experimental system without any requirement for skills in statistics thanks to ready-to-use online tools. We believe that this work is a step forward in the clinical interpretation of human genetic variants.

## Introduction

Genetic tests, that aim to identify disease-associated germline variants in the genome of patients and relatives, have greatly expanded these last years, together with the number of predisposing genes scrutinized [1]. Genetic tests are proposed by genetic counselors to identify the carriers of genetic variants and to define appropriate clinical follow-ups and treatments for these carriers. The detection of a variant can have severe psychological and physical consequences for the tested patients, depending on whether the variant is known to be pathogenic (associated with disease development), neutral (not related to disease development) or of unknown significance (VUS). Thus, clinical decision-making after genetic testing requires the establishment of reliable variant classifications. The best support is to use methods that attribute a probability of pathogenicity for each variant identified. Because genetic/epidemiological methods, such as co-segregation, case-control, co-occurrence and familial data analyses, provide such probabilities [2], they remain the gold standard in clinical decision-making after genetic testing (see an example in S1 Table). However, genetic/epidemiological methods are time consuming, as they require a substantial amount of observations. Moreover, they are unsuitable for a large number of variants identified, for instance when the number of known carriers is rare. As genetic tests are evolving towards the use of multi-gene panels, whole exome and whole genome sequencing [3], the number of VUS detected is inevitably increasing [1], which stresses the need to improve variant classification [3].

Functional assays have been designed to circumvent the limitations of genetic/epidemiological methods. The generic "functional assay" term refers to *in vitro* and *in vivo* systems, able to classify VUS by assessing their influence on protein function or conformation [4]. Functional assays have been widely developed for genes involved in cancers [5] and *BRCA1* has become the leading gene analyzed, with 23 different assays proposed, to date [6]. However, despite the genuine interest for strategies that alleviate the limitations of genetic/epidemiological methods, the main challenge of functional assessment remains in its inclusion into clinical decision-making. Indeed, most of the functional assays lack statistical validation [4]. Moreover, analyses are usually based on visually defined cut-offs [6]. Finally, except in rare cases [7,8], the resulting variant classifications lack the probability of pathogenicity provided by genetic/epidemiological methods.

Here, we used experimental as well as computational approaches to overcome these limitations. We evaluated the clinical utility of four different *BRCA1* functional assays, designed in yeast cells, by assessing 40 *BRCA1* missense mutations, previously classified by genetic/epidemiological methods. To interpret these results, we developed a novel approach, referred to as "Mann-Whitney-Wilcoxon (MWW) method", that defines a non-arbitrary best cut-off value between the neutral and pathogenic variants and that refines variant ranking in data from functional assays. We also developed a computational system that transforms the dual classification between "pathogenic" or "neutral", provided by the non-arbitrary best cut-off, to a probabilistic classification adapted to clinical decision-making. This system of classification, referred to as "probability system", uses the fluctuation of the best cut-off to derive probabilities of pathogenicity for each assessed variant. We show the benefit of our computational model, coupling the MWW method and the probability system, using the experimental data from the four *BRCA1* functional assays and using theoretical simulations. We also illustrate that our model is adapted to experimental systems far beyond the genetic variant assessment, with the probabilistic classification of small interfering RNAs (siRNAs) tested for human cell growth inhibition in high throughput screening.

## Results

### Forty missense mutations to evaluate the colony size assay

**Fig 1:**
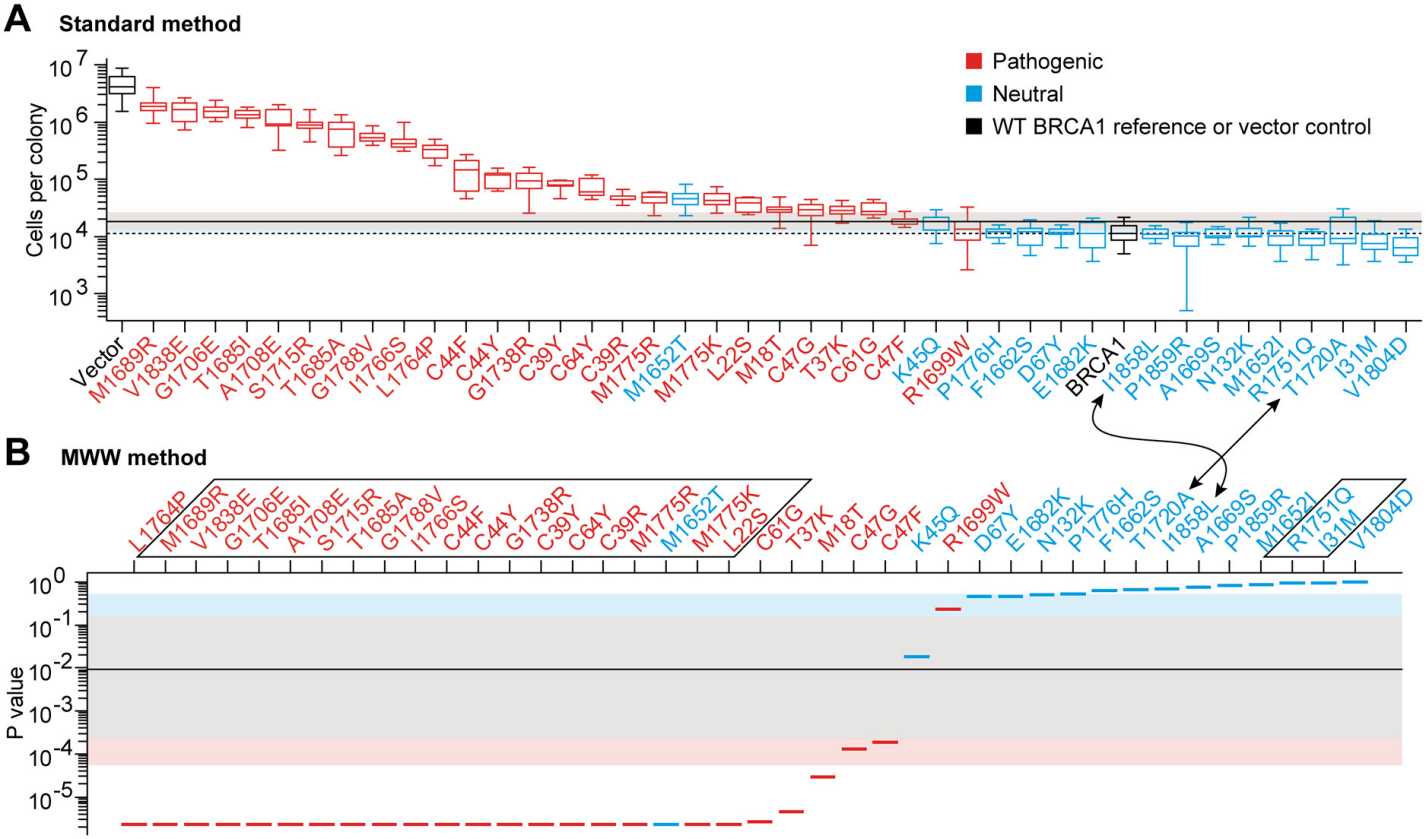
Relative position of the variants in the Colony Size assay and fluctuation of the best cut-off. (**A**) Waterfall distribution of colony sizes, according to median values (standard method). Boxplot representation results from 9 (mutants) or 36 (BRCA1 and Vector) colony size values. The red and blue colors of the boxes indicate the pathogenic and neutral mutations, respectively, according to their prior classification. Box central bar, median; box, interquartile range (50% of the distribution); whiskers, extreme values; dotted horizontal line, median of BRCA1; thick horizontal line, experimental best cut-off (see S2 Fig). The distribution of the best cut-off fluctuation, obtained after random sampling (bootstrap), of the 9 mutants and 36 BRCA1 values, is visualized by the pink, grey and light blue areas, that delimit 4%, 90% and 4.9% of the distribution, respectively, which altogether represents a total coverage of 98.9%. (**B**) Waterfall distribution according to p values (MWW method). The p value assigned to each variant is symbolized by a segment. The upside-down representation facilitates the comparison of the mutation arrangement with the one obtained in **A**. Arrows pinpoint a modification of the mutation rank depending on the method used. Framed mutations indicate identical p values (see S4 Table). Segment colors, thick horizontal line and colored areas, as in **A**.

The colony size assay is a *BRCA1* functional assay, that has been designed in the yeast model organism, which allows rapid, large-scale and cost-effective variant assessment [9], but has never been subjected to clinical validation yet. In this functional assay, expression of the full length wild type (WT) BRCA1 protein in yeast, induces a growth defect [9–11]. Indeed, after 63 hours of growth on an agarose plate, a single yeast cell gives rise to a colony varying between 5,000 and 21,000 cells (Fig 1A, BRCA1), while colonies reach several millions of cells without protein expression (Fig 1A, Vector control). To ascertain the utility of this assay in clinical medicine, we selected 40 BRCA1 missense mutations, according to their neutral or pathogenic classification by genetic/epidemiological methods (S1 Fig and S2 Table). We confirm that pathogenic missense mutations restore the proliferation rate of yeast cells [10,11]. Indeed, pathogenic mutations have a global tendency to give rise to the biggest colonies, while colony sizes arising from neutral mutations remain close to those of the WT BRCA1 reference (Fig 1A). However, the Colony Size assay does not fully discriminate between pathogenic and neutral mutants. Indeed, variant medians appeared to continuously decrease from M1689R (highest median) to V1804D (lowest median), without clear gap between the pathogenic and neutral regions. Moreover, the neutral M1652T mutation is clearly within the pathogenic sector and the pathogenic R1699W mutation slightly overlaps the neutral region. In such situations, it is critical to have a sound evaluation of the sensitivity, specificity and accuracy of the assay (see the definitions in the S1 Text), which depends on a non-arbitrary and optimal cut-off setting.

### The standard method to define the best cut-off

The standard method is based on the Youden's index, a classical approach to compute the sensitivity and specificity in a dataset. Using this, the cut-off of 17,910 cells per colony gives the best combined sensitivity and specificity, with 96% (24/25) and 93% (14/15) respectively (Table 1 and S2A Fig). In total, 95% (38/40) of the mutations are correctly classified. The M1652T neutral mutation is misclassified as pathogenic and the pathogenic R1699W mutation is misclassified as neutral (S3 Table). From now on, we refer to "experimental best cut-off", "experimental sensitivity", "experimental specificity" and "experimental accuracy" as the best cut-off, sensitivity, specificity and accuracy obtained from the experimental data.

**Table 1:** Experimental sensitivity, specificity and accuracy of functional assays and siRNA screening using the experimental best cut-off.

	Standard method					MWW method				
Assay	Best cut-off	Youden's index	Sensitivity	Specificity	Accuracy	Best cut-off	Youden's index	Sensitivity	Specificity	Accuracy
Colony Size	17 910	0.89	24/25 = 0.96	14/15 = 0.93	38/40 = 0.95	0.009	0.89	24/25 = 0.96	14/15 = 0.93	38/40 = 0.95
Liquid Medium	0.172	0.81	22/25 = 0.88	14/15 = 0.93	36/40 = 0.90	0.00023	0.80	20/25 = 0.80	15/15 = 1.00	35/40 = 0.88
Spot Formation	0.295	0.77	21/25 = 0.84	14/15 = 0.93	35/40 = 0.88	0.165	0.77	21/25 = 0.84	14/15 = 0.93	35/40 = 0.88
Yeast Localization	0.057	0.69	19/25 = 0.76	14/15 = 0.93	33/40 = 0.83	0.186	0.69	19/25 = 0.76	14/15 = 0.93	33/40 = 0.83
siRNA	604	1.00	1/1 = 1.00	2/2 = 1.00	3/3 = 1.00	0.00044	1.00	1/1 = 1.00	2/2 = 1.00	3/3 = 1.00

"Accuracy" is the number of mutations or siRNA correctly classified. Sensitivities, specificities and accuracies, associated with the experimental best cut-off, were referred to as "experimental", to distinguish them from those computed using the probability system of variant classification.

### The MWW method to define the best cut-off

The disadvantage of the standard method is that mutations are characterized by a single value, here by the median of colony sizes, which can lead to paradoxes in the mutant classification. For instance, the neutral I1858L mutation displays a median of cells per colony higher than the median of the neutral T1720A mutation. Thus, in the mutant ranking, I1858L is closer to the pathogenic group of mutations than T1720A (arrows in Fig 1A). However, T1720A has three values out of nine over the experimental best cut-off, which are thus in the pathogenic area, while I1858L has none (S3A Fig). Therefore, in terms of dispersion range, T1720A could be considered as "more pathogenic" than I1858L. To overcome such paradoxes in variant classification, we developed a nonparametric approach to define the best non-arbitrary cut-off value, that takes into account more information from distributions than the median value alone. This method is based on the MWW test [12–14]. Since the p value of this test provides a quantification of the overlap between two distributions (S4 Fig), we compared each mutant distribution to the WT BRCA1 distribution. The p values obtained defined relative positions of the mutant distributions using the WT BRCA1 distribution as a reference position (Fig 1B and S4 Table). Contrary to the standard method described above, the cut-off used to compute the sensitivity and specificity parameters is a p value. Any mutant with a p value below the p value cut-off, indicates a mutant classified as pathogenic. In contrast, a mutant distribution with a p value over the p value cut-off is considered as neutral. Strikingly, the MWW method solves the paradoxes observed with the standard method, since T1720A is closer to the pathogenic group of mutations than I1858L (arrows in Fig 1B). Moreover, the experimental sensitivity and specificity remains unchanged (Table 1 and S2E Fig). This confirms that the M1652T and R1699W mutations cannot be correctly classified by the Colony Size assay, even when using more information from the experimental data than the variant medians alone. However, it also emphasizes that the variant classification, provided by the MWW method, does not diminish the high sensitivity and specificity of the assay. From this, we conclude that the MWW method is a reliable alternative to the standard method to define a non-arbitrary cut-off in data from functional assessments.

### The probability system of classification

**Fig 2:**
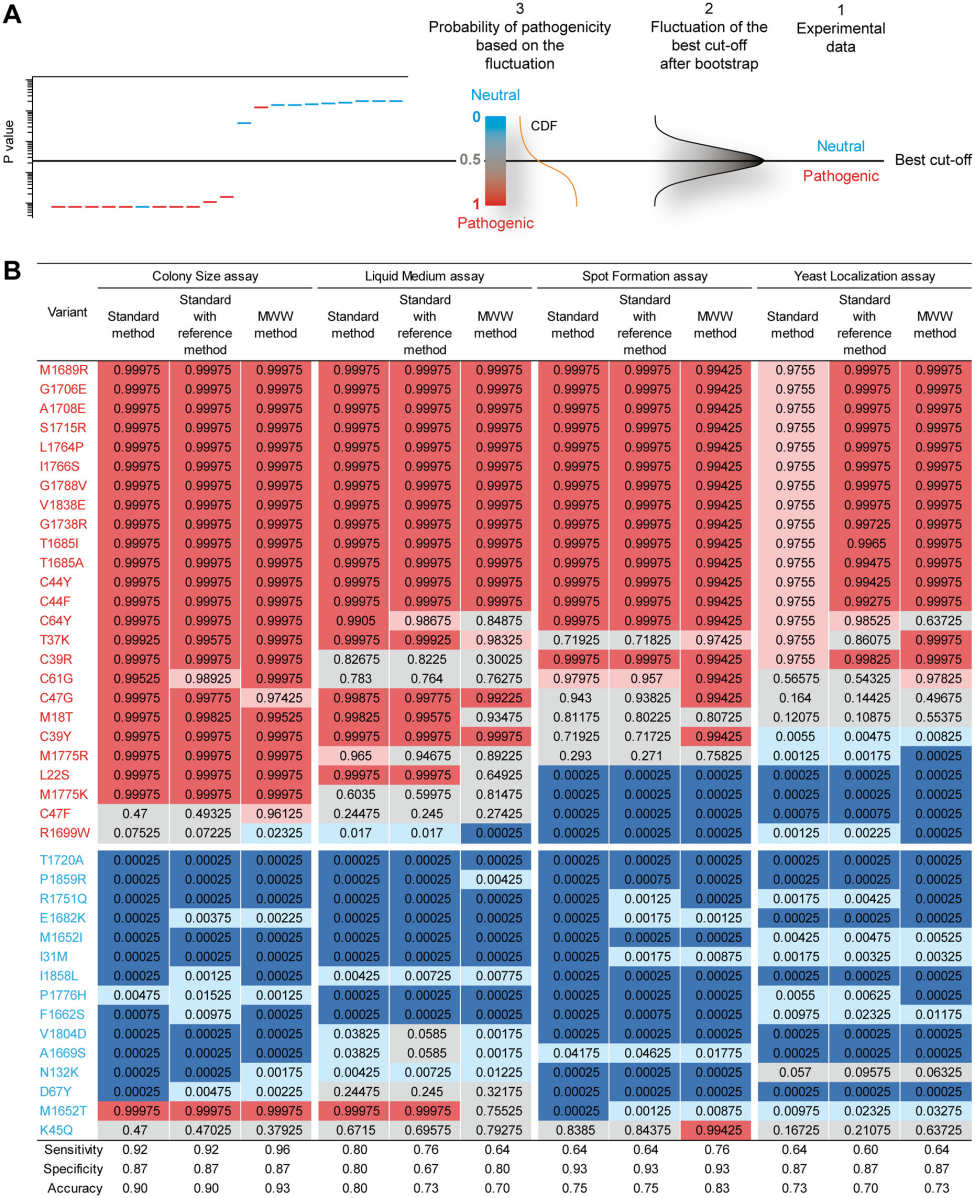
Variant classification using the probability system. (**A**) Schematic of the probability system of classification. The left figure depicts a theoretical waterfall distribution of pathogenic and neutral missense mutations, as in Fig 1B. Horizontal black line, experimental best cut-off. (1) Variant classification according to the experimental best cut-off (method used in Table 1). (2) Distribution of the best cut-off generated by bootstrap analysis from the experimental data. (3) Cumulative distribution function (CDF) derived from the distribution of the best cut-off. This CDF provides a probabilistic classification of the variants, depending on their positions in the CDF. (**B**) Classification of the *BRCA1* variants assessed in four functional assays. Colored background in the table indicates the five-class nomenclature, as in S1 Table. Names in red and blue indicate the pathogenic and neutral mutations, respectively, according to their prior classification. The sensitivity, specificity and accuracy computation are detailed in S6 Table.

Recently, two-component models have been proposed for the probabilistic classification of variants based on functional assessment. These parametric models require the normal distribution of the neutral and pathogenic values [7,8]. However, as shown in S5A Fig, the Colony Size assay is poorly compatible with such models, due to the bimodal distribution of the pathogenic values. Therefore, we designed an alternative nonparametric and more versatile system of classification. This system is based on the fact that the best cut-off is a random variable that fluctuates, depending on the experimental values. We asked what the variant classification would be, using the Colony Size assay, taking this fluctuation into account. For this, we performed sampling with replacement (bootstrap) of the colony size values, by randomly choosing 9 values among the 9 from each mutant, and 36 values among the 36 from the BRCA1 reference control. Next, using this new set of sampled data, we applied the standard or MWW method to obtain the best cut-off. We repeated this procedure a large number of times, which allowed us to define a best cut-off distribution for the standard and MWW methods (S5 Table). We also used a third method, referred to as "standard with reference method". It is similar to the standard method, except that the best cut-off distribution obtained includes the fluctuation of the WT BRCA1 reference, as explained in the S1 Text. Notably, the standard with reference method allows an additional comparison with the MWW method, which also includes the fluctuation of the WT BRCA1 reference. Finally, we designed the probability system of classification. This system allows to assign a probability of pathogenicity to each assessed variant, using the best cut-off fluctuation (Fig 2A and S6 Fig). The rationale is that the farther a variant is from the core of the best cut-off fluctuation, the more robust is its classification as either pathogenic or neutral. A probability close to 1 indicates that the variant can be classified as pathogenic, with a low risk of misclassification as neutral due to the fluctuation of the best cut-off. A probability close to 0 indicates that the variant can be classified as neutral, with a low risk of misclassification as pathogenic due to the fluctuation of the best cut-off. Finally, a probability of 0.5 designates no preferential classification as either neutral or pathogenic (variant completely unknown). With such probabilities, the five-class nomenclature proposed by Plon et al [26]. (S1 Table) can be directly applied to functional assays. Probabilities obtained for the Colony Size assay are shown in Fig 2B. Strikingly, a level of uncertainty was generated, notably with variants classified as "uncertain" (class 3). This highlights the critical influence of the best cut-off fluctuation in variant classification. In addition, the MWW method exhibits the best accuracy, with 37/40 mutations correctly classified versus 36/40 for the standard and standard with reference methods. When including the number of misclassified mutations, the MWW method shows a balance of 35 mutations, *ex-aequo* with the two other methods (S6 Table). Altogether, these results confirm the possibility to use the MWW method in variant classification. In addition, the probability system seems to be an effective and simple way to obtain a probabilistic classification of variants in functional assessment.

### Evaluation of three additional functional assays

We validated three other functional assays, by assessing the same 40 mutations used in the Colony Size assay. The Liquid Medium assay monitors the growth defect of yeast cells expressing BRCA1 (S7 and S8 Figs), as in the Colony Size assay, but in liquid instead of solid medium [11]. The Spot Formation assay is derived from the observation that the BRCA1-mCherry fusion protein accumulates in a single aggregate in the nucleus of yeast cells. This aggregate is referred to as "spot" due to its visual signature using fluorescent microscopy. We previously showed that pathogenic missense mutations decrease the proportion of cells showing one spot [11]. Here, we confirmed this effect (S9 and S10 Figs). The last assay tested was the Yeast Localization assay. Whereas cytoplasmic spots are rare in yeast cells expressing the WT BRCA1 protein, this event has a tendency to increase in the presence of pathogenic mutations [11]. Here, we confirmed this effect (S11 and S12 Figs). However, albeit promising, none of these three assays provided a better discrimination than the Colony Size assay, to distinguish between pathogenic and neutral variants. This was notably shown by the experimental sensitivity and specificity computed (Table 1 and S3 Table).

### The classification model challenged by the four functional assays

We took advantage of the experimental differences among the four assays (recapitulated in S7 Table) to detect potential flaws in the MWW method. In contrast, the MWW method constantly overcomes the incoherent ranking generated by the standard method (see examples in S7, S9 and S11 Figs). This is achieved without reducing the experimental accuracy compared to the standard method (Table 1), except for a minor decrease in the Liquid Medium assay (88% versus 90%). Also, no flaws were detected in the probability system, which would result in an unexpected high level of misclassifications (Fig 2B). Interestingly, accuracy of the MWW method is globally better than in the standard or standard with reference method, with the best accuracy of 93% in the Colony Size assay, 83% in the Spot formation assay, and with the best *ex-aequo* accuracy of 73% in the Yeast Localization assay (Fig 2B). Variant misclassification was slightly higher in the MWW method, compared to the two other methods, with one more misclassification in the Colony Size and in the Spot Formation assays, one less in the Liquid Medium assay, and *ex-aequo* in the Yeast Localization assay ("Total number of variants misclassified" column in S6 Table), even if the balance between accuracy and misclassification maintains the MWW method as the best one, *ex-aequo* with the standard method ("Balance" column in S6 Table). Finally, contrary to the MWW method, the standard method suffers from a lack of sensitivity in the Yeast Localization assay, since none of the pathogenic mutations are classified as class 5 (Fig 2B). Overall, the analysis of four functional assays did not reveal any major flaw in the probability system of classification. In addition, the results obtained with the MWW method confirm the possibility to classify variants using more information from the variant distribution than the median value alone.

### The classification model in theoretical simulations

To complete the detection of potential flaws in our classification model, we analyzed theoretical situations. A reference situation was designed, similar to that in the Colony Size assay (S8 Table). Next, different parameters were scrutinized: the position of the pathogenic mutations (S13 Fig), neutral mutations (S14 Fig), or WT BRCA1 reference (S15 Fig), the initial sensitivity and specificity of the assay before using the probability system (S16 Fig), the number of neutral and pathogenic variants used (S17 Fig), the number of values in the variants and in the WT reference distributions (S18 Fig), and the range of the variant and WT reference distributions (S19 Fig). Results are recapitulated in S9 Table and summarized in Table 2. The standard with reference method shows strong usage limitations, notably when the WT reference exhibits a negative median or a median close to zero (Table 2 and S15E Fig, middle panel). Interestingly, the MWW method is not affected by such situations. The main limitation detected is an extreme situation in which the WT reference distribution falls outside of the range of the neutral and pathogenic distributions (e.g., S15A Fig, left panel), which impairs the sensitivity of the probability system of classification (Table 2 and S15E Fig, right panel). Except for this extreme situation, we confirm the efficient behavior of our classification model, coupling the MWW method and the probability system: (1) when the pathogenic and neutral distributions are strictly identical, all the mutations are classified as class 3 (Table 2 and S13D Fig, right panel), (2) the sensitivity and specificity of the probability system of classification increase when pathogenic mutations move away from the WT BRCA1 reference distribution (S13D Fig, right panel), and (3) when pathogenic mutations are contaminated by neutral mutations (experimental specificity reduced), the sensitivity of the probability system of classification is decreased (Table 2 and S16E–S16G Fig, right panel), and vice versa. This last result is an important criterion for classification, since unknown mutants that would be located in a pathogenic region containing neutral mutations, could not be formally classified as pathogenic. Therefore, it is noteworthy that the experimental sensitivity and specificity values are taken into account by our classification model. Interestingly, the model is poorly sensitive to the number of neutral or pathogenic mutations used to validate a given assay (S17E–S17G Fig, right panel), as long as the number of values in the dataset is high enough (S18E–S18G Fig, right panel). Supplemental information is provided in the S1 Text. This notably includes an extensive analysis of the best cut-off fluctuation, which explains the lack of sensitivity of the standard method, mentioned above in the Yeast localization assay (Fig 2B) and also shown in theoretical situations (see the legend of S13 Fig). It also contains specific procedures for variant classification (e.g., Bayesian inference, combination of functional results, assessment of VUS), as well as procedures to fit the proposed model to other situations. It finally includes the ProClass toolbox that generates the probabilistic classification of variants, adapted to most kind of functional assays.

**Table 2:** Usage limits of the probability system of classification.

Method	Limits	Figure
All of them	- Weak experimental specificity	S16
	- Weak experimental sensitivity	S16
	- Low number of values in the data set	S18
	- No dispersion in both the WT reference and the mutant distributions	S19
Standard	- Close neutral and pathogenic medians	S13,S14
Standard with reference	- Close neutral and pathogenic medians	S13,S14
	- WT reference median close to zero and fluctuation of the raw best cut-off far from this median	S15
	- WT reference with a negative median	S15
MWW	- Close neutral, pathogenic and WT reference distributions	S13,S14
	- WT reference distribution outside of the range of the neutral and pathogenic distributions	S15

This table recapitulates the results obtained with the three standard, standard with reference and MWW methods, when challenged by theoretical distributions. Usage limits were defined as situations in which the neutral and pathogenic mutations, used to generate the fluctuation of the best cut-off, are finally not classified as class 1/2 and 4/5, respectively (neutral mutations finally classified as class 3, 4 or 5, and pathogenic mutations finally classified as class 3, 2 or 1).

### The classification model in high throughput screening

**Fig 3:**
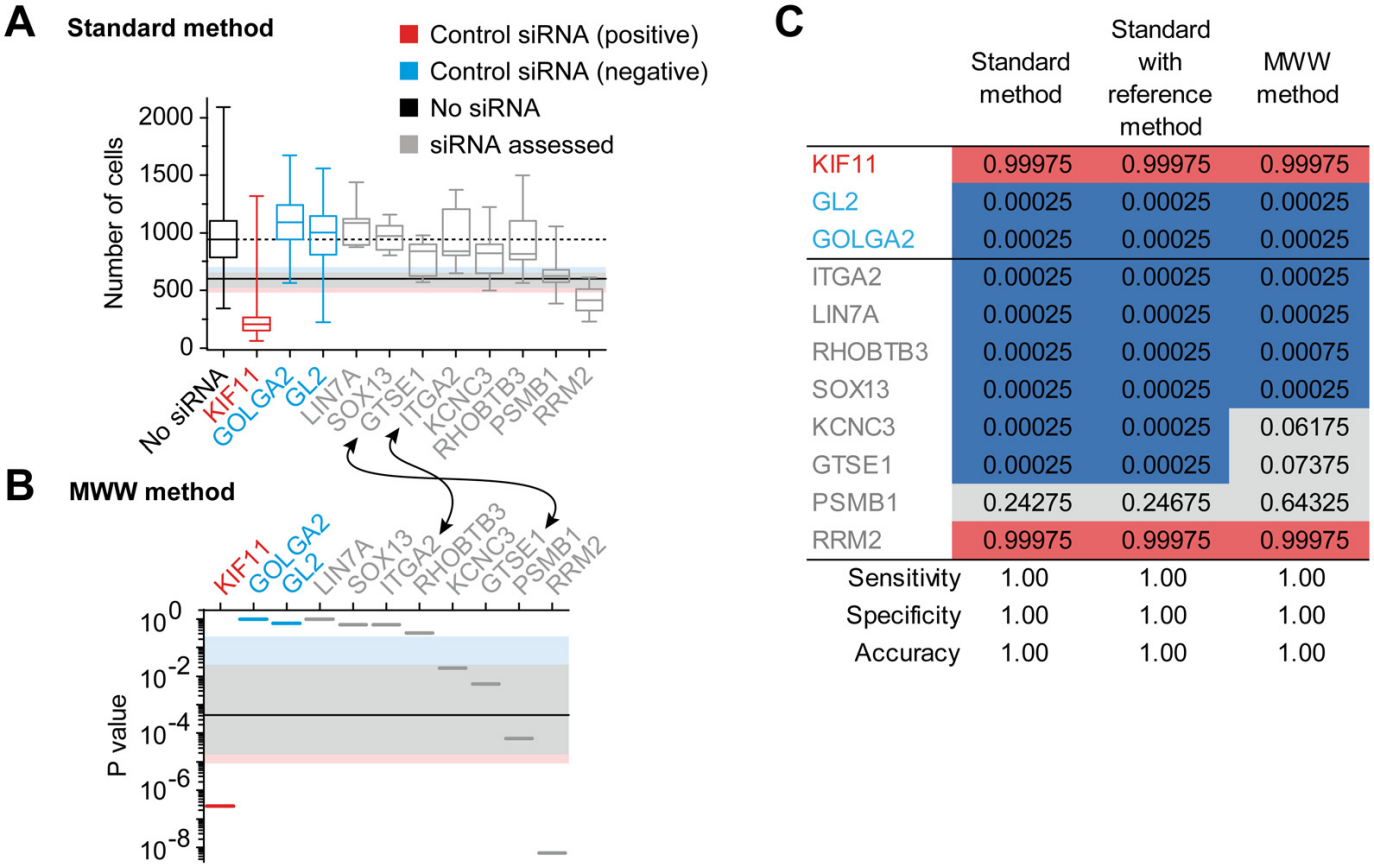
High throughput siRNA screening and fluctuation of the best cut-off. An average of 150 human prostate tumoral cells were plated, treated with siRNAs targeting the indicated gene and grown for 72 hours before cell counting. The WT reference (No siRNA), the positive control of cell growth inhibition (siKIF11), the two negative controls of cell growth inhibition (siGOLGA2 and siGL2) and 8 among 406 siRNA targeted genes are shown. The complete analysis of the 406 targeted genes is available using the ProClass toolbox and the included siRNA full.txt file, as explained at the end of the README.doc file. (**A**) Waterfall distribution of cell growth after siRNA treatment, according to median values (standard method). As in Fig 1A, except that boxplot representation results from 12 (siRNA) or 1,140 (No siRNA) values. (**B**) Waterfall distribution according to p values (MWW method), as in Fig 1B. (**C**) Classification of the siRNA targeted genes, as in Fig 2B, except that probabilities are related to cell growth inhibition, with the corresponding five-class nomenclature: "no inhibition" (blue, class1), "likely no inhibition" (light blue, class2), "unclear inhibition" (grey, class3), "likely inhibition" (light red, class4) and "inhibition" (red, class5).

We wondered if the classification model developed for genetic variants could be easily extended to other decision-making situations. The analysis of theoretical situations showed that variant classification remains accurate when only one neutral and one pathogenic variant are available (S17E–S17G Fig). This indicates that the fluctuation of the best cut-off supports decision-making in situations represented by a limited number of positive and negative controls. To confirm this, we analyzed data from 406 genes targeted by small interfering RNAs (siRNAs), screened for their capability to inhibit the proliferation of a human prostate tumoral cell line (Fig 3). The "No siRNA" control, the siKIF11 positive control and the siGOLGA2 and siGL2 negative controls were treated as WT reference, pathogenic and neutral variants, respectively. Structure of the data is reported in S7 Table. As in the BRCA1 functional assays, the experimental accuracy was not impaired using the MWW method, compared to the standard method (Table 1). In addition, no flaws were detected in the probability system, since the accuracy remained at 1, whatever the standard, standard with reference or MWW method used (Fig 3C). Finally, the advantage of the MWW method is again highlighted in the final classification of the screened siRNAs. Indeed, in the siRNA ranking, based on the median values, siGTSE1 is closer to the negative controls than siITGA2 (Fig 3A). By taking the distribution of these two siRNAs into account, the MWW method switches their ranking position (Fig 3B), so that siGTSE1 is finally classified as "unclear effect on cell growth inhibition" (Fig 3C, MWW method), instead of "no cell growth inhibition" (Fig 3C, standard and standard with reference methods). Thus, this demonstrates that our probabilistic model is also adapted to the classification of experimental data far beyond the functional assessment of genetic variants.

## Discussion

We provide the statistical validation of four *BRCA1* functional assays, as well as a classification model that facilitates the incorporation of functional assay results into clinical decision-making. The probabilistic model is based on the fluctuation of the best cut-off, which is driven by the fluctuation of the experimental data. Thus, the variant classification provided reflects the robustness of a cut-off-based decision-making towards data fluctuation. The model has the advantage to be nonparametric, easy to handle and easy to adapt to most kind of functional assays. Moreover, among the variants incorporated in functional assays, the model only depends on those previously classified by genetic/epidemiological methods as pathogenic or neutral. It is not influenced by unknown variants, meaning that the subsequent incorporation of unknown variants in a functional assay does not require a new analysis of the best cut-off fluctuation. These features of our model contrast with parametric models, proposed for variant classification [7,8].

We achieved a widespread analysis of the best cut-off fluctuation dedicated to decision-making (completed in the S1 Text). This analysis is focused on the classification of genetic variants, but it is also valid for other decision-making situations compatible with our classification model, such as high throughput siRNA screenings. Using many different kinds of data structures (four BRCA1 functional assays, one siRNA screen and 93 theoretical situations), three different methods of best cut-off fluctuation were scrutinized: the standard, the standard with reference and the MWW methods. From this study, we conclude that the standard with reference method is poorly compatible with a versatile classification model, due to important lacks of accuracy when the WT reference exhibits a negative median or a median close to zero (S15E Fig, middle panel). The standard method has the advantage to support decision-making in experimental situations devoid of a WT reference. The MWW method has the advantage to use more information from the distribution of the classified elements than the median value alone. This refines the ranking and the final probabilistic classification. Contrary to the standard method, the MWW method is adapted to experimental situations in which the neutral and pathogenic variants (or the negatives and positives controls) are represented by a single value if the WT reference encompasses a significant number of different values (S18E Fig, compare the left and right panels for the mutant with one value). However, the MWW method is poorly adapted to experimental situations where the WT reference distribution is more or less outside of the range of the neutral and pathogenic distributions (e.g., S15A Fig, left panel). Thus, we propose to prioritize the MWW method if the data are compatible with this method, notably if the WT reference is well embedded in the neutral/negative distributions, or if the WT reference is between the neutral/negative and the pathogenic/positive distributions, and to use the standard method otherwise. The different methods are proposed in the ProClass toolbox available online (see the S1 Text).

Interestingly, none of the four yeast assays is able to correctly classify the R1699W pathogenic and the K45Q neutral variants. Pathogenicity of R1699W has been long-established in independent studies, using different genetic/epidemiological methods [15–17], confirming that yeast cells are unable to detect the deleterious impact of R1699W [18]. This emphasizes that the mechanism of R1699W, leading to tumor development, is different from the other pathogenic missense variants of *BRCA1*. It is probably related to a protein-binding defect without major BRCA1 structural destabilization [19]. The classification of K45Q has been established by a single epidemiological study [20], with little evidences of neutrality (e.g., probability of being pathogenic of 11% by family history prediction). However, the absence of any functional impact has been confirmed in three different functional assays using mammalian cells [21,22], which stresses a specific effect of K45Q in yeast cells, that remains to be explained.

Finally, this work showed that the yeast organism can be used to classify variants positioned in both Nter and Cter parts of BRCA1. Among the four assays analyzed, the Colony Size assay is the most accurate (93%) and the most robust to data fluctuation (one class 3 variant). The Liquid Medium and Yeast Localization assays may also be attractive for diagnosis due to the absence of false negative results detected, notably when using the MWW method. Interestingly, the Yeast Localization assay allows the identification of pathogenic variants that delocalize the BRCA1 protein into the cytoplasm. If confirmed in human cells, this assay could define subcategories in the pathogenic variants of BRCA1, based on different cellular mechanisms leading to tumor development.

## Methods

### Plasmids

All plasmids are derived from pJL48 [11], a modified version of pESC-URA (Agilent Technologies), in which the *MYC* epitope has been removed by *Sal*I-*Xho*I digestion and vector ligation. In this plasmid expression of the cDNA is controlled by the *GAL1* promoter, inducible by galactose and repressed by glucose. The backbone of the human *BRCA1* (MIM# 113705) cDNA used, corresponds to the AY888184.1 GenBank sequence with a TGA stop codon instead of TAG. To facilitate the cloning of BRCA1 missense mutations, silent mutations were inserted in the cDNA to generate 4 new restriction sites: SalI (c.1020A>C + c.1023T>C), AvrII (c.4662A>T), FseI (c.4839T>G + c.4842A>G + c.4845T>C) and XhoI (c.5502C>T). Of note, the WT BRCA1 and BRCA1-mCherry plasmids used in this study (pPT60 and pPT63 respectively, see S10 Table) are different from the pJL45 and pGM40 plasmids, used in our previous publication [11], by the addition of the 4 restriction sites. The 40 missense mutations were generated by targeted mutagenesis (Genscript Company, Piscataway, NJ, USA) on intermediate plasmids. Next, we inserted the mutated cDNA fragment into the pPT60 and pPT63 plasmids by a single digestion—ligation step. All resulting plasmid constructs were verified by sequencing the promoter, the full cDNA and the terminator.

### Yeast strains

Transformation of the *Saccharomyces cerevisiae* haploid BY4741 or YKR082W-GFP strains were performed as previously described [11]. The strains generated are referenced in S11 Table. To facilitate the description, we referred to the cells transformed with pESC-URA as the name of the cDNA inserted into the plasmid. Thus, "BRCA1" refers to yeast cells transformed with the plasmid containing the WT BRCA1 cDNA; "M18T" refers to yeast cells transformed with the plasmid containing the M18T mutated version of the BRCA1 cDNA; and "vector" refers to yeast cells transformed with the same plasmid without inserted cDNA. Three independent transformants per strain, also referred to as "clones", were selected after each transformation. We observed that lithium acetate transformation can result in diploidisation of haploid cells. To control this, the ploidy of each clone was verified by FACS analysis, using the yeast strain BY4741 (haploid) and BY4743 (diploid) as a control. Next, in the different assays, cells were grown in glycerol-lactate medium (GL-URA) as previously described [11]. Addition of galactose in the medium (GAL) induced the expression of *BRCA1*, while addition of glucose (GLU) strongly repressed the expression of *BRCA1*.

### Fluorescence-activated cell sorting (FACS) analysis

Cells were grown in log phase in YPD medium (1% yeast extract, 2% peptone, 2% dextrose, 60 μM Adenine, 8 μM NaOH). 10^7^ cells were collected and put at 4°C to block the cell cycle. Cells were centrifuged at 4°C and resuspended in 70% ethanol. After 1 hour incubation at room temperature (RT), cells were centrifuged and resuspended in freshly made sodium citrate [50 mM] pH7. Sonication was performed to dissociate cell aggregates (Vibracell and probe CV33 (Bioblock Scientific, Illkirch, France), pulse 30%, time 15 seconds). Cells were centrifuged and resuspended in sodium citrate [50 mM] pH7 + RNAse A [0.25 mg/ml]. After 1 hour incubation at 50°C, cells were centrifuged, resuspended in sodium citrate [50 mM] pH7 + Propidium Iodide [16 μg/ml] and analyzed using an Accuri (BD Bioscience, San Jose, CA, USA).

### Colony size assay

This assay was previously named "small colony phenotype" (SCP) assay. The method already published [11] was slightly improved as follows: (1) GL-URA+galactose and GL-URA+glucose plates were incubated 63 hours and 50 hours respectively (instead of 52 hours), and (2) the biggest colony of each plate, representing the size of at least five other colonies on the plate, was chosen for cell counting. This prevents the choice of rare but extremely big colonies (outliers). For the simultaneous assessment of 10 variants by a single technician, the time required between the delivery of the intermediate plasmids (see above) and the final results is 20 days.

### Liquid medium assay

This method already published [11] was slightly improved as follows: during glucose induction, cells were diluted at 0.5×10^6^ cells/ml (instead of 10^6^ cells/ml) for the 15 hour culture time at 30°C. Galactose induction conditions remained as before. For the simultaneous assessment of 10 variants by a single technician, the time required between the delivery of the intermediate plasmids (see above) and the final results is 20 days.

### Spot formation and yeast localization assays

This method already described [11] was slightly improved as follows. Briefly, Nup133-GFP cells, expressing the WT or mutated BRCA1 protein fused to mCherry, were induced for 4 hours with galactose before analysis using live fluorescent microscopy. The previously named "yeast localization phenotype" (YLP) assay [11] was subdivided into two assays in this study. The Spot Formation assay monitors the proportion of cells showing a single aggregate of WT or mutated BRCA1, visible in fluorescent microscopy, without considering the intracellular localization. This aggregate is also referred to as "spot". Cells with several aggregates were not considered in this assay. The Yeast Localization assay monitors the proportion of spot volumes localized in the cytoplasm of yeast cells. Picture acquisitions were previously described [11]. For each clone, at least three fields, containing at least 100 cells, were acquired. For the Spot Formation assay, the number of cells showing one spot was manually counted. Next, the proportion of cells containing one spot was computed by dividing the number of cells showing one spot to the total number of cells (one value per clone). For the Yeast Localization assay, images of the three fields were deconvoluted [23] and the volume Vol_ij_ of each spot i, in the field j, was measured using the 3D Object Counter plugin [24] of ImageJ. Next, each spot was manually categorized as "inside" or "outside" the nucleus. Finally, the proportion of volume outside the nucleus was computed using the formula (Σ_*i*_Σ_*j*_*Vol*_*ij*/*outside*_) / (Σ_*i*_Σ_*j*_*Vol*_*ij*/*outside*_ + Σ_*i*_Σ_*j*_*Vol*_*ij*/*inside*_), which led to one value per each clone assessed. This proportion quantifies the cytoplasmic localization of the mCherry protein fused to BRCA1. For the simultaneous assessment of 10 variants by a single technician, using the Spot Formation and Yeast Localization assays, the time required between the delivery of the intermediate plasmids (see above) and the final results is 21 days.

### High throughput siRNA screening

IGR-CaP1 epithelial cells, derived from a human prostate primary tumor [25], were plated in 384-well plates at 750 cells/well, were allowed to adhere overnight and then were transfected with a single siRNA from a siRNA library targeting 406 different genes. siKIF11, siGL2 and siGOLGA2 were used as controls. After 72 hours, cells were fixed and nuclei were stained with DAPI. Images were acquired with an INCell 2000 automated wide-field system (GE Healthcare, Little Chalfont, UK) and cell counts were quantified in each well with the INCell Analyzer workstation software (GE Healthcare). The pictures analyzed represent 20% of the well surface, which corresponds to an average of 150 cells initially plated for this surface.

### Statistical and computational methods

Statistical and computational methods, as well as R source codes, are provided in the S1 Text.

## Supporting Information

S1 Supporting Information Joined Supplementary Methods, Figures and Tables. (PDF)

S1 Text Supplementary Methods. (PDF)

S1 Fig Position of the BRCA1 missense mutations selected. RING domain (amino acid 8–96); BRCT, BRCA1 C-terminal domains (amino acid 1646–1736 and 1760–1855). Pathogenic and neutral mutations are in red and blue, respectively. Fourteen mutations (3 neutral and 11 pathogenic) map within the RING domain. An additional neutral mutation, N132K, flanks the Cter part of this domain, resulting in 15 mutations located in the Nter extremity of BRCA1. Twenty-five mutations (11 neutral and 14 pathogenic) lie in the BRCT domain, at the Cter extremity of the protein. Of note, the RING domain suffers from a lack of neutral missense mutations classified by genetic/epidemiological methods, explaining why only 3 neutral mutations from our selected panel, lie in this domain. Moreover, no pathogenic missense mutations, between the amino acids 65 and 1684, are documented in the BRCA1 mutation databases (S2 Table). Therefore, this study was restricted to the RING and BRCT domains of BRCA1.
(PDF)

S2 Fig Experimental best cut-off, experimental sensitivity and experimental specificity of functional assays. (**A-D**) Standard method. The medians of the mutant distributions were ordered (as in the waterfall distribution, Fig 1A) and each average position between two consecutive medians was defined as a cut-off. For example, in Fig 1A, the cut-off between the two first mutations, M1689R and V1838E, was (1,877,333 + 1,621,333) / 2 = 1,749,333 cells per colony. Next, sensitivity was defined as the proportion of pathogenic mutant medians above (for the Colony Size, Liquid Medium and Yeast Localization assays) or below (for the Spot Formation assay) a selected cut-off. The associated specificity was defined as the proportion of neutral mutant medians below (Colony Size, Liquid Medium and Yeast Localization assays) or above (Spot Formation assay) the same selected cut-off. For example, for the cut-off between M1689R and V1838E in Fig 1A, the sensitivity was 1/25 = 4% and the specificity was 15/15 = 100%. Sensitivity and specificity were computed for each cut-off (left panels). Areas surrounding the curves delimit the 95% confidence interval according to the binomial law. The ROC curve (right panel) pinpoints the best cut-off (black number), meaning the cut-off that maximizes both sensitivity and specificity of the assay. Precisely, the best cut-off is the one associated with the highest vertical distance of the ROC curve to the dotted diagonal. This highest vertical distance is referred to as "Youden's index", which is equal to max[sensitivity + specificity—1]. In other words, the best cut-off is the cut-off of the Youden's index. Other cut-off values are also positioned on the ROC curve (grey numbers). Blue, red and orange dots on the curves of the left and right panels represent the different cut-offs tested. The black vertical bar, in the left panel, pinpoints the best cut-off defined on the ROC curve. (**E-H**) MWW method. As in **A-D** for mutant p values, instead of mutant medians. In all assays, sensitivity was defined as the proportion of pathogenic mutant p values below a selected cut-off, and the associated specificity was defined as the proportion of neutral mutant p values above the same selected cut-off. (**A**, **E**) Colony Size assay. (**B**, **F**) Liquid Medium assay. (**C**, **G**) Spot Formation assay. (**D**, **H**) Yeast Localization assay.
(PDF)

S3 Fig Supplemental information in the colony size assay. (**A**) Dotplot distribution of colony sizes. For each missense variant, the nine represented values result from three independent clones examined in three independent experiments. For the BRCA1 reference and the Vector control, the 36 values result from three independent clones examined in twelve independent experiments (represented in the three panels, except for the Vector values absent in the top panel). Grey bar, median; dotted horizontal line, median of BRCA1; black horizontal line, experimental best cut-off. The top panel (Nter extremity of BRCA1) has a y-axis scale magnified compared to the middle and bottom panels (Cter extremity of BRCA1). (**B**) As in **A** with glucose instead of galactose media (see the S1 Text) to verify that each clone had no intrinsic growth defect, independent of WT or mutated BRCA1 expression. The three independent clones from **A** were examined in one experiment.
(PDF)

S4 Fig The MWW method. (**A**) Upper-sided MWW test. The theoretical examples are based on the Colony Size assay but are also valid for the Liquid Medium and Yeast Localization assays. Each distribution of the WT BRCA1 reference (black) and the missense mutation (purple) are composed of 8 theoretical values, represented by 8 dots in the diagram. The p value of the MWW test is used to score the overlap of the mutant and the WT BRCA1 distributions. See the S1 Text for full details. From left to right: (1) when all the mutant values are below the BRCA1 values, the upper-sided MWW test results in a p value close to 1; (2) the p value decreases when the mutant distribution begins to overlap the BRCA1 distribution; (3) the p value is approximately 0.5 when the two distributions completely overlap; (4) the p value continues to decrease when the mutant distribution is above the BRCA1 distribution, with a partial overlap; (5) finally, the p value is lowest when the mutant distribution is fully above the BRCA1 distribution. In theory, neutral and pathogenic mutations should have a p value close to 0.5 and 0, respectively, as depicted by the color scale below the diagram. However, the absolute p value attributed to each variant is not determinant. What is significant is the relative positions between the mutant distributions, indicated by the p values, using the WT BRCA1 distribution as a reference position. The lowest p values represent systematically the pathogenic mutations, and the highest the neutral mutations. Thus, the upper-sided MWW test is used when pathogenic mutations are above the neutral ones in the experimental data. (**B**) Lower-sided MWW test. All of the theoretical examples shown are based on the Spot Formation assay. As in the upper-sided MWW test, the lowest and highest p values still represent the pathogenic and neutral mutations, respectively, but the pathogenic mutations are below the neutral ones in the experimental data.
(PDF)

S5 Fig Distribution of the pathogenic and neutral values. (**A**) Colony Size assay. The left panel exhibits dotplot distributions. Boxplots provide distribution parameters: box central bar, median; box, interquartile range (50% of the distribution); whiskers, extreme values. The middle panel shows the normal Quantile-Quantile (QQ) plot of the pathogenic values. Dots forming a straight line suggest that the values are normally distributed. Black line, straight line through the quantiles 25% and 75%. The right panel shows the normal QQ plot of the neutral values. (**B**) Liquid Medium assay. (**C**) Spot Formation assay. (**D**) Yeast Localization assay.
(PDF)

S6 Fig Description of the probability system of classification. (**A**) As in Fig 2A. (**B**) Theoretical example showing how the values from the best cut-off fluctuation, derived from the MWW method, are converted into probabilities of pathogenicity. Top table: best cut-off distribution composed of 10 best cut-off values, resulting from 10 bootstraps (n_bootstrap_ = 10). The probability attributed to each best cut-off value was 1 / n_bootstrap_. Bottom table: cumulative distribution functions (CDF) generated from the best cut-off distribution. In this table, probabilities of each repeated cut-off value were summed. For instance, the best cut-off value of 0.9 is repeated 4 times in the top table, leading to a probability of 0.4. The CDF represents the sum of the probabilities present in the second row of the bottom table. Three CDF were computed. The first reaches the cumulated probability of 1. The second begins with the cumulated probability of 0. The third is the average of the two first CDF. This average CDF delivers the probability of pathogenicity used to classify variants. Right panel: plot of the average CDF. To classify a variant (e.g., M18T), the variant p value, derived from the MWW method, is positioned on the x-axis (vertical grey bar). Next, the closest average CDF value is attributed to the variant as a probability of pathogenicity. In this example, the best cut-off value, closest to M18T, is 0.5. Thus, the corresponding probability 0.75 is attributed to M18T. (**C-E**) Average CDF of the Colony Size (CS), Liquid Medium (LM), Spot Formation (SF) and Yeast Localization (YL) assays, obtained with the standard (**C**), standard with reference (**D**) or MWW method (**E**). The same procedure, described in **B**, was applied to the 2,000 best cut off values obtained for each assay and each method used. The CDF is ascending when the pathogenic mutations are above the neutral ones, and descending when the pathogenic mutations are below. The number of different best cut-off values is indicated (n = 2,000 when no identical best cut-off values within distributions).
(PDF)

S7 Fig Relative position of the variants in the Liquid Medium assay and fluctuation of the best cut-off. (A-B) As in Fig 1. One OD unit corresponds to 10^8^ cells / ml. Arrows pinpoint the ranking of the L22S and C47G mutations, which is improved using the MWW method, as explained in the main text introducing this method. The incoherent ranking observed with the standard method results from L22S that exhibits four values below the experimental best cut-off while C47G has none (S8A Fig).
(PDF)

S8 Fig Supplemental information in the liquid medium assay. (A-B) Same as for the Colony Size assay (S3 Fig). One OD unit corresponds to 10^8^ cells / ml.
(PDF)

S9 Fig Relative position of the variants in the Spot Formation assay and fluctuation of the best cut-off. (A-B) As in Fig 1, except that boxplots and p values resulted from 3 (mutants) or 12 (BRCA1) values. Arrows pinpoint the ranking of the M18T and C39Y mutations, which is improved using the MWW method, as explained in the main text introducing this method. The incoherent ranking observed with the standard method results from M18T that exhibits one value above the experimental best cut-off (shown by the top whisker overlaying the thick horizontal line) while C39Y has none.
(PDF)

S10 Fig Supplemental information in the Spot Formation assay. Nup133-GFP cells, expressing the WT or mutated BRCA1 protein, fused to mCherry, were analyzed using live fluorescent microscopy. (**A**) Examples of images acquired. Nup133-GFP allows visualization of the nuclear membrane within the cell, in the green channel. Overlayed images of GFP and mCherry (Merge) as well as transillumination images (Trans) are also shown. Scale bar, 5 μm. (**B**) Image quantifications. Bars and whiskers indicate median and extreme values for each distribution, respectively. For each assessed clone, the total number of cells showing one spot, two spots, more than two spots, or a diffusive signal, was counted. Three clones were assessed once, for each missense mutation, and 4 times for the WT BRCA1 reference. Thus, each bar in the diagram is the result of 3 values, for each missense mutation, and 12 values for the WT BRCA1 reference. In the Spot Formation assay, only the "1 spot" category is considered. The dotted horizontal line represents the median of BRCA1. (**C**) Dotplot representation of the 12 BRCA1 values. The equivalent dotplot distribution of each mutant is shown in **B**, with the 3 values from each mutant represented by the top of the dark grey bar and the two whisker extremities, and also in S9A Fig, where the 3 values correspond to the median bar and the two whisker extremities.
(PDF)

S11 Fig Relative position of the variants in the Yeast Localization assay and fluctuation of the best cut-off. (**A-B**) As in Fig 1, except that the boxplots and p values are the results of 3 (mutants) or 12 (BRCA1) values. Delocalization of the mCherry fluorescent signal from the nucleus ranges from 0 (no cytoplasmic delocalization) to 1 (full cytoplasmic delocalization). Arrows pinpoint the ranking of the A1669S and D67Y mutations, which is improved using the MWW method, as explained in the main text introducing this method. The incoherent ranking observed with the standard method results from A1669S that exhibits one value above the experimental best cut-off while D67Y has none. (**C**) Dotplot representation of the 12 BRCA1 values forming the BRCA1 boxplot in **A**. The equivalent dotplot distribution of each mutant is shown in **A**, with the 3 values from each mutant represented by the median bar and the two whisker extremities.
(PDF)

S12 Fig Supplemental information in the yeast localization assay. Fluorescent images acquired in the Yeast Localization assay, as in S10A Fig. The arrow points to rare cytoplasmic spot in cells expressing the WT BRCA1-mCherry protein. Scale bar, 2 μm.
(PDF)

S13 Fig Effect of the position of the pathogenic mutations on the probability system of classification (theoretical situation). The parameters of the theoretical distributions used are detailed in S8 Table. The reference situation is as follows: n_mutant_ = 9, n_BRCA1_ = 36, n_neutral_ = 15 and n_pathogenic_ = 25. In addition, medians and ranges of the neutral and WT BRCA1 distributions were made systematically equal. Distributions of the neutral and pathogenic mutations were identical, except for the shift of the pathogenic values from the neutral mutations, according to the formula v_ij_ + 36 × s, with s representing the shift intensity and v_ij_ representing the value i of the pathogenic mutation j. When s = 0, pathogenic and neutral distributions are identical. Fluctuations from the best cut-off were obtained exactly as performed for the Colony Size, Liquid Medium, Spot Formation and Yeast Localization assays. (**A-C**) Examples of shift intensities and best cut-off fluctuation results. The graphs depicted are similar to those in Fig 1, except that the standard, standard with reference and MWW methods are shown respectively on the left, middle and right of the figure. In the standard and standard with reference methods, boxplots are replaced by dotplots with the median of the distributions indicated by a grey segment. The s values are indicated (top left). In the subsequent supplemental figures, the position of the pathogenic mutation medians are as in **C** (s = 2). The grey horizontal line indicates the median of the best cut-off fluctuation. (**D**) Probabilities of pathogenicity obtained for the neutral (blue line) and pathogenic variants (red line), depending on the shift intensity of the pathogenic mutations. Y-axis, log10(p / (1—p)) with p being the probability of pathogenicity of the variants (0 corresponds to p = 0.5); right colored classes, five-class nomenclature with the horizontal grey lines showing the 0.99, 0.95, 0.05 and 0.001 limits of the classes (see S1 Table). In the standard method, the slight erratic curves and the lack of specificity sometimes observed (blue line in the class 2 instead of class 1) is due to the fact that this method generates a low number of different best cut-off values (between 8 and 64) in the best cut-off distributions, as explained in the S1 Text. As summarized in S9 Table, these results confirm that the probability system of classification is an efficient variant classifier. Indeed, whatever method is used, when the pathogenic and neutral distributions are strictly identical, they all locate inside the class 3 area (i.e., the system cannot classify any variants in such kind of functional assay). Moreover, the probability system of classification is improved when the pathogenic mutations shift from the neutral sector towards the pathogenic sector, since the probability of pathogenicity increases for the pathogenic variants and decreases for the neutral ones.
(PDF)

S14 Fig Effect of the position of the neutral mutations on the probability system of classification (theoretical situation). See S13 Fig for details. Neutral mutations were shifted according to the formula v_ij_ + 36 × s, with s representing the shift intensity and v_ij_ representing the value i of the neutral mutation j (when s = 0, medians and extreme values of the BRCA1 and neutral distributions are identical. When s = 2, pathogenic and neutral distributions are identical). (**A-D**) Examples of shift intensities and best cut-off fluctuation results. The s values are indicated (top left). (**E**) Probabilities of pathogenicity obtained for the neutral (blue line) and pathogenic variants (red line), depending on the shift intensity of the neutral mutations. As summarized in S9 Table, these results highlight divergences between the different methods. With the standard method and the standard with reference methods (**E**, left and middle panels), sensitivity and specificity of the probability system of classification decrease when the neutral mutations approach the pathogenic mutations. With the MWW method (**E**, right panel), the probability system of classification results in a complete misclassification of the pathogenic mutations when the neutral distributions do not overlap the WT reference distribution (s ≥ 1). Of note, these analyses treat extreme situations. In practice, the WT reference should be well embedded within the neutral distributions. The opposite situation would raise question about the WT reference or neutral mutations used.
(PDF)

S15 Fig Effect of the position of the WT BRCA1 reference on the probability system of classification (theoretical situation). See S13 Fig for details. Values of the WT BRCA1 distribution were shifted according to the formula v_i_ + 36 × s, with s representing the shift intensity and v_i_ representing the value i of the BRCA1 reference (when s = 0, medians and extreme values of the neutral and BRCA1 distributions are identical. When s = 2, medians and extreme values of the pathogenic and BRCA1 distributions are identical). Of note, these theoretical analyses treat extreme situations. In practice, the WT reference should be well embedded in the neutral distributions. The opposite situation would raise question about the WT reference or neutral mutations used. (**A-D**) Examples of shift intensities and best cut-off fluctuation results. The s values are indicated (top left). (**E**) Probabilities of pathogenicity obtained for the neutral (blue line) and pathogenic variants (red line), depending on the shift intensity of the WT reference. As summarized in S9 Table, these results highlight divergences between the different methods. As expected, the standard method is not affected by the position of the WT BRCA1 distribution (**E**, left panel). In contrast, the standard with reference method is strongly influenced by the position of this reference (**E**, middle panel). When the WT BRCA1 median shifts towards the null value, sensitivity and specificity of the probability system of classification are decreased, with a complete loss of sensitivity and specificity (i.e., systematic classification as class 3) when the WT BRCA1 median is null (s ≈ -0.514). This was expected since the standard with reference method is based on best cut-off values divided by the WT BRCA1 median. Thus, a division by zero generates relative best cut-offs with an infinite value. Such issues are compensated only when best cut-offs are close to the WT BRCA1 median. This was shown in the Liquid Medium and Yeast Localization assays. Using the standard or standard with reference method provided similar variant classification (Fig 2B), even if the WT BRCA1 medians of these assays approached zero, with 0.144 and 0.03 respectively (S4 Table). In conclusion, a situation, in which the WT reference median is close to zero, with the fluctuation of the raw best cut-off far from this median, will guarantee a weak sensitivity and specificity of the probability system of classification. Concerning the standard with reference method, it is also noteworthy that a negative value of the WT reference median (s < -0.514) inverts the classification (**E**, middle panel), as expected, regardless of the values from the neutral and pathogenic mutations. When comparing the standard with reference method versus the MWW method, the later has the advantage of being independent of the WT reference values, as only overlapping distributions matter. Specificity of the probability system of classification is not affected by the position of the WT reference, contrary to sensitivity (**E**, right panel). The main weakness of the MWW method occurs when the WT reference distribution falls outside of the range of the neutral and pathogenic distributions (as in **A**, left panel), which generates misclassification of the pathogenic mutations as neutral.
(PDF)

S16 Fig Effect of the experimental sensitivity and specificity on the probability system of classification (theoretical situation). See S13 Fig for details. The experimental sensitivity and specificity were modulated by assigning certain pathogenic mutants in the neutral region and certain neutral mutants in the pathogenic region, respectively. The experimental sensitivity and specificity values indicated were those obtained with the experimental best cut-off, as explained in S2 Fig. These values are referred to as "initial" sensitivity and specificity, as opposed to the sensitivity and specificity of the probability system of classification, obtained after bootstrap analysis. (**A-D**) Examples of experimental sensitivities/specificities and best cut-off fluctuation results. (**E-G**) Probabilities of pathogenicity obtained for the neutral (blue line) and pathogenic variants (red line), depending on decreases from experimental specificity (**E**), experimental sensitivity (**F**) or both (**G**). As summarized in S9 Table, these results confirm that the probability system of classification is an efficient variant classifier. A decrease of the experimental specificity indicates that the pathogenic area is contaminated by neutral variants, which reduces the probability of pathogenicity of the pathogenic variants (class 5 towards class 3). In the same manner, a decrease of the experimental sensitivity indicates that the neutral area is contaminated by pathogenic variants, which enhances the probability of pathogenicity of the neutral variants (class 1 towards class 3). This was observed using the three standard, standard with reference and MWW methods. Of note, the situations studied used systematically: experimental sensitivity + experimental specificity ≥ 1 (otherwise representing an inappropriate use of the experimental information, i.e., pathogenic and neutral sectors incorrectly positioned).
(PDF)

S17 Fig Effect of the number of neutral and pathogenic mutations on the probability system of classification (theoretical situation). See S13 Fig for details. (**A-D**) Examples showing the number of neutral and pathogenic mutations tested, with best cut-off fluctuation results. (**E-G**) Probabilities of pathogenicity obtained for the neutral (blue line) and pathogenic variants (red line), following a decrease in the number of neutral mutations (**E**), pathogenic mutations (**F**) or both (**G**). As summarized in S9 Table, these results show that the probability system is poorly sensitive to the number of neutral and pathogenic mutations incorporated, whatever method is used.
(PDF)

S18 Fig Effect of the number of mutant and BRCA1 values on the probability system of classification (theoretical situation). See S13 Fig for details. Number of values was modulated so that the range and median of the distributions remained the same, as shown in S8 Table. (**A-D**) Examples showing the number of mutant or BRCA1 values tested, with best cut-off fluctuation results. (**E-G**) Probabilities of pathogenicity obtained for the neutral (blue line) and pathogenic variants (red line), following a decrease in the number of mutant values (**E**), BRCA1 values (**F**), or both (**G**). As summarized in S9 Table, these results confirm that the probability system of classification is an efficient variant classifier. Whatever method is used, a decreasing number of values in the dataset affects the probabilities of both the pathogenic and neutral variants (**G**), which tend toward 0.5 (class 3). Thus, the probability system prevents decision-making when data is lacking. As expected, the standard method is not affected by the number of BRCA1 values (**F**, left panel). The standard with reference and the MWW methods are insensitive to the number of mutant values if the number of BRCA1 values is high (**E**, middle and right panels). However, a decrease in the number of BRCA1 values lowers the probability of pathogenicity of the pathogenic variants (**F**, middle and right panels), but with a strong recovery when the fluctuation of the best cut-off is no longer influenced by the fluctuation of the WT reference (n_BRCA1_ = 1). Of note, the best cut-off does not fluctuate when n_mutant_ = 1 and n_BRCA1_ = 1 (**G**), which results in a probability of pathogenicity equal to 0.5 for both the pathogenic and neutral variants. Moreover, using the standard method, when n_mutant_ = 9, the classification of the neutral mutations is class 2 (**E**, left panel), which explains the lack of specificity frequently observed in S13–S19 Figs.
(PDF)

S19 Fig Effect of the range of mutant and BRCA1 distributions on the probability system of classification (theoretical situation). See S13 Fig for details. Distribution ranges were modulated so that medians remained the same, as shown in S8 Table. The range factor r, indicated on the graphs, illustrates the relative dispersion of the distributions. When r = 0, the dispersion is null. (**A-D**) Examples showing the ranges of the mutant and BRCA1 distributions tested, with best cut-off fluctuation results. (**E-G**) Probabilities of pathogenicity obtained for the neutral (blue line) and pathogenic variants (red line), following a range decrease of the mutant distributions (**E**), BRCA1 distribution (**F**), or both (**G**). As summarized in S9 Table, these results indicate that the probability system of classification is affected mainly when the range of the BRCA1 and mutant distributions is null, whatever method is used. In this situation, the fluctuation of the best cut-off is null and all the mutations are considered as absolutely unknown (probability of pathogenicity equal to 0.5).
(PDF)

S20 Fig Western blot analysis. After 4 hours of BRCA1 expression, lysates of 6 x 10^6^ cells were examined for the presence of the protein (theoretical size: 200 kDa) with an anti-BRCA1 antibody. Tubulin or Actin was used as a loading control and was probed using an anti-Tubulin or anti-Actin antibody on the same membrane after stripping the first labeling. Signal intensities of full lanes, relatively to the BRCA1 lane, are indicated below. Of note, protein levels three times higher than the WT BRCA1 protein level (normalized to 1) systematically correspond to pathogenic mutations. (**A**) BRCA1 (Colony Size and Liquid Medium assays). (**B**) BRCA1-mCherry (Spot Formation and Yeast Localization assays). (**C-G**) Dotplot with the Spearman coefficient of correlation indicated. Pathogenic and neutral mutations, as well as the WT BRCA1 reference, are represented by a red, blue or black dot, respectively. (**C**) Correlation between the relative signal intensities of **A** and **B**. (**D-E**) Correlation between the relative signal intensities of **A** and medians of the Colony Size or Liquid Medium assay. (**F-G**), correlation between the relative signal intensities of **B** and medians of the Spot Formation or Yeast Localization assay.
(PDF)

S21 Fig Exact probability distribution of the best cut-off in the standard, standard with reference and MWW methods (theoretical situation). The theoretical situation was analyzed as follows: one neutral and one pathogenic mutation (n_neutral_ = 1 and n_pathogenic_ = 1), with two values per mutant (n_mutant_ = 2, value 1 and 2 for the neutral mutant, and value 3 and 4 for the pathogenic mutant) and two values in the WT BRCA1 reference (n_BRCA1_ = 2, value 1 and 2). (**A**) The graphs depicted are similar to those in Fig 1, except that boxplots are replaced by dotplots with median of the distributions indicated by a grey segment. The black horizontal line represents the experimental best cut-off. The best cut fluctuations (colored areas) are not represented but quantiles are shown in **F**. (**B**) Table recapitulating all of the possible results when sampling 2 values, with replacement, among the 2 neutral, 2 pathogenic and 2 WT BRCA1 values. Each row is a different combination that provides a best cut-off value, for each method used. The framed row highlights the combination identical to the experimental situation in **A**. In this simple situation (1 neutral and 1 pathogenic variant), the best cut off computed, in each row, is the median of the two variant medians (standard method), the median of the two variant medians divided by the WT BRCA1 median (standard with reference method) and the median of the two variant p values (MWW method). (**C-E**) Variant classification using the probability system, with the standard (**C**), standard with reference (**D**) and MWW (**E**) methods, as in S6B Fig. Colored numbers in the table correspond to the different probabilities of pathogenicity designed by the model. The color code respects the five-class nomenclature depicted in S1 Table: grey, class 3; light blue, class 2; pink, class 4. Positions of the neutral and pathogenic variants are represented by a blue and red arrow, respectively. The number below each arrow designates the variant value used in the probability system to attribute the probability of pathogenicity, which corresponds to the median, median divided by the WT reference median or p value, indicated in the framed row of **B**. For instance, in the standard method (**C**), the pathogenic variant, with a median of 3.5, has the probability 0.94 (class 3). (**F**) Variant classification using the quantile system. Quantiles were computed from the 27 best cut-off values from **B**, for each method. The colored background defines the intervals within the best cut-off distribution, as explained in S23 Fig. Arrows depict the position of the neutral and pathogenic variants, as in **C-E**.
(PDF)

S22 Fig Additional information about the classification model. (**A**) Schematic of the exact best cut-off distribution influenced by different parameters, assuming no ties. The number of neutral (n_neutral_) and pathogenic (n_pathogenic_) variants influence the exact best cut-off distribution only if the number of values per mutant (n_mutant_) is above 1. The number of values in the WT reference (n_BRCA1_) does not influence the exact best cut-off distribution in the standard method, only in the standard with reference and MWW methods. (**B**) Schematic of the approximate best cut-off distribution influenced by the number of bootstraps performed. Importantly, a single bootstrap (n_bootstrap_ = 1) does not lead to the experimental best cut-off, except if n_best exact_ = 1. (**C**) Accuracy of the probability and quantile systems of classification. The schematic illustration is valid, using either the exact or approximate best cut-off distribution. (**D**) Correcting factor f_cor_ used in the probability system of classification, depending on the parameter a (see the S1 Text). f_cor_ = (n_neutral_ + n_pathogenic_) / (n_neutral_ + n_pathogenic_ + a). The framed value (a = 2) was the value used in S13–S15 Tables.
(PDF)

S23 Fig Description of the quantile system of classification. (**A**) The left figure depicts a theoretical waterfall distribution of pathogenic and neutral missense mutations, as in Fig 1A. (1) Variant classification according to the experimental best cut-off. This cut-off (horizontal black line), that maximizes the experimental sensitivity and specificity in the waterfall distribution, is obtained by ROC curve analysis, as in S2 Fig. In the case of the Colony Size assay, mutations above the best cut-off are classified as pathogenic and mutations below are classified as neutral. (2) Bootstrap analysis provides a fluctuation of the best cut-off, depending on the values of the mutations and the WT BRCA1 reference randomly chosen. The fluctuating best cut-off values form a distribution, as depicted in the schematic. (3) Quantile system of variant classification according to the fluctuation of the best cut-off. The reasoning is the following: the distribution of the fluctuating best cut-off defines quantiles (Q) that delimit the probability of the presence of this variable. As an example, the quantile Q0.99 is the value that separates the 99% lowest values from the 1% highest values in a distribution. This means that the probability to have the best cut-off above the quantile Q0.99 is 1%. Thus, in the Colony Size assay using the standard method, a mutation with the median above the quantile Q0.99 can be considered as pathogenic with a 1% probability of error. Indeed, this mutation could be neutral, but only if the best cut-off is above the median, which has a 1% probability, or less, to occur. This reasoning allows separation of the best cut-off distribution into 5 intervals, based on the five-class nomenclature proposed by Plon et al [26], with each interval defining the probability of the best cut-off presence within the waterfall distribution. (**B**) Quantiles that delimit the 5 intervals of classification according to the assay and the method used. CS, Colony Size; LM, Liquid Medium; SF, Spot Formation; YL, Yeast Localization assay. Note that the quantiles differ, depending on whether the pathogenic mutations are above or below the best cut-off. For instance, in the standard method, the quantiles of the Colony Size assay are Q0.99, Q0.95, Q0.05 and Q0.001 (pathogenic mutants above the best cut-off), while quantiles are Q0.01, Q0.05, Q0.95 and Q0.999 in the Spot Formation assay (pathogenic mutants below the best cut-off). However, these two cases generate the same intervals (e.g., probability 1% for the class 5, see **C** and **D**). Cut-off values corresponding to these quantiles are listed in S5 Table for each assay and for each method. (**C**) Interval limits in the case of the Colony Size assay, using the standard or the standard with reference method. P(X > Q0.99) = 1% is the probability to obtain the best cut-off variable X strictly over the quantile Q0.99, shown here as 26,222 cells per colony for the standard method, and 2.416 x 11,200 (BRCA1 median of the experimental data) = 27,062 cells per colony for the standard with reference method. (**D**) Interval limits in the case of the Colony Size assay, using the MWW method.
(PDF)

S24 Fig Variant classification using the quantile system. Names in red and in blue indicate the pathogenic and neutral mutations, respectively, according to their prior classification. See also S16 Table. The black frames pinpoint the divergent classification compared to that in the probability system (Fig 2B).
(PDF)

S25 Fig Quantile system of classification (theoretical situation). Effect of different experimental parameters was assessed in theoretical situations, exactly as for the probability system of classification, meaning that the best cut-off fluctuations depicted were those used in S13–S19 Figs. Red line, position of the median or p value of the pathogenic mutants; blue line, position of the median or p value of the neutral mutants. The pink, grey and blue areas define intervals within the best cut-off distribution, as explained in S23 Fig. For clarity, the extreme red and blue areas were not displayed. Sensitivity of the quantile system is maximal when the red line is beyond the pink area. Specificity is maximal when the blue line is beyond the light blue area. Accuracy is maximal when sensitivity and specificity are maximal. Finally, sensitivity, specificity and accuracy of the quantile system are null when both lines are in the grey area, or in the wrong side of the best cut-off fluctuation. (**A-D**) Evolution of the best cut-off fluctuation depending on either the shift intensity of the pathogenic mutations (**A**), or the shift intensity of the neutral mutations (**B**), or the shift intensity of the WT reference (**C**), or the experimental sensitivity and specificity (**D**). The corresponding panels, depicted for the probability system of classification, are shown in S13D Fig for **A**, S14E Fig for **B**, S15E Fig for **C** and S16E–S16G Fig for **D**. As summarized in S17 Table, these results did not reveal any flaws. The quantile system behaves as the probability system, in these situations.
(PDF)

S26 Fig Quantile system of classification (theoretical situation). Evolution of the best cut-off fluctuation depending on either the number of neutral and pathogenic mutations (**A**) or the number of mutant and BRCA1 values (**B**), as in S25 Fig. The corresponding panels, depicted for the probability system of classification, are shown in S17E–S17G Fig for **A** and S18E–S18G Fig for **B**. As summarized in S17 Table, these results reveal a major flaw in the quantile system of classification. Using the standard and MWW methods, the sensitivity and specificity is maximal, regardless of the number of values present within the mutant or BRCA1 distributions (**B**). Using the standard with reference method, the sensitivity is affected when the number of values in the BRCA1 distribution is decreased, but is maximal when n_BRCA1_ = 1. Thus, contrary to the probability system, the quantile system is not correctly influenced by the amount of experimental values resulting from functional assessment.
(PDF)

S27 Fig Quantile system of classification (theoretical situation). Evolution of the best cut-off fluctuation, depending on the range of the mutant and BRCA1 distributions, as in S25 Fig. The corresponding panels, depicted for the probability system of classification, are shown in S19E–S19G Fig. As summarized in S17 Table, these results reveal a major flaw in the quantile system of classification. A null range means that all of the values, present in a distribution, are identical (ties). Because ties are related to a low measurement accuracy, an efficient variant classifier should penalize a high number of ties in a dataset, which is not observed here, whatever method is used.
(PDF)

S1 Table IARC variant classification. Five-class nomenclature proposed by the International Agency for Research on Cancer (IARC) for variant classification, with specific recommendations for clinical management, depending on the probability of pathogenicity obtained by epidemiological methods [26].
(XLS)

S2 Table BRCA1 mutations selected. ^a^ Empty cell, no data. ^b^ HGVS: human genome variation society (http://www.hgvs.org/mutnomen/). ^c^ IARC classification as in S1 Table. ^d^ UMD-BRCA1 database (29-January-2015, http://www.umd.be/BRCA1/). ^e^ LOVD-IARC database (29-January-2015, http://hci-exlovd.hci.utah.edu/home.php?select_db=BRCA1). ^f^ LOVD Leiden database (29-January-2015, http://databases.lovd.nl/shared/genes/BRCA1). This database gathers all information from the literature, including functional assays, which explains the high level of ambiguous results. Blue cell, neutral; red cell, pathogenic; grey cell, conflicting reports. ^g^ Mutations recommended by the ENIGMA consortium. C64Y is namely classified as "Clinically important" which is here converted to "4/5".
(XLS)

S3 Table Misclassified mutations using the experimental best cut-offs. Experimental best cut-offs from Table 1.
(XLS)

S4 Table Experimental data from the 4 functional assays and the siRNA screening. Relative median, median divided by the WT BRCA1 median or by the No siRNA median; sample size, number of values; framed mutations indicate identical p values. The "ties" column indicates the number of values repeated. For instance, in the first row, one value is repeated twice, another one four times, and a third twice ("ties" is the statistical term used to designate "identical values").
(XLS)

S5 Table Distribution of the best cut-offs after bootstrap analysis. Values obtained after sampling, with replacement, using the original data obtained from the four functional assays and the siRNA screen (see the bootstrap procedure A in the method section). Q indicates the quantile (Q0.050 is the quantile 5%). For the standard with reference method, the results shown represent values either relative to the median of the WT reference (BRCA1 reference or No siRNA), which allows an immediate comparison of the cut-off distributions between the different assays (top), or the same values multiplied by the experimental median of the WT reference observed in the corresponding assay (bottom). For example, 0.960 is the quantile 0.1% in the Colony Size assay, which corresponds to 11,200 x 0.960 = 10,754 cells per colony. Experimental medians used are 11,200 cells per colony (Colony Size assay), 0.144 OD600 (Liquid Medium assay), 32% (Spot Formation assay), 3% (Yeast Localization assay) and 945 (siRNA screen), as indicated in S4 Table. Of note, in the standard method, the WT reference was ignored during the sampling. Thus, the resulting fluctuation of the best cut-offs does not depend on the fluctuation of the WT reference. This explains why distributions were narrowed in the standard method, compared to the standard with reference method. For example, 12,133 cells per colony is the quantile 0.1% in the Colony Size assay, but in the standard with reference method, the same quantile is slightly farther from the distribution median, with a value of 11,200 x 0.960 = 10,754 cells per colony.
(XLS)

S6 Table Quantitative analysis of Fig 2B. "Balance" indicates the number of mutations correctly classified (class 1 + 2 + 4 + 5) minus the total number of mutations misclassified. "Sensitivity" and "Specificity" represent the number of variants correctly classified, divided by the number of variants in the prior classification (npathogenic = 25 and nneutral = 15). "Accuracy" is the number of mutations correctly classified (class 1 + 2 + 4 + 5) divided by the total number of mutations (n = 40).
(XLS)

S7 Table Features of the assays. See S4 Table for details.
(XLS)

S8 Table Examples of theoretical situations analyzed. The "reference situation" column indicates the initial values and parameter settings. From this, distribution parameters were modified and the resulting variant classification was scrutinized. The separating factor s (BRCA1, neutral or pathogenic) shifts the values of the distribution, according to the formula v_ij_ + 36 × s, with v_ij_ representing the value i of the distribution j (BRCA1, neutral or pathogenic). The range factor r modulates the extreme values of a distribution, according to the formula me ± 17.5 × r, where me represents the median of the distribution. Whatever the value of r, the values of any distribution are equally spread.
(XLS)

S9 Table Effect of functional assay parameters upon the probability system of classification. Sensitivity and specificity are defined here as the probabilities of pathogenicity (p) attributed to the pathogenic and neutral mutations, respectively, which reflects the accuracy of the probability system of variant classification. Sensitivity is maximal if p ≥ 0.95 for the pathogenic mutations. Specificity is maximal if p < 0.05 for the neutral mutations. Misclassification is defined as p ≥ 0.95 for the neutral mutations and p < 0.05 for the pathogenic mutations. The experimental sensitivity and specificity derives from the experimental best cut-off, as explained in S2 Fig. They differ from the sensitivity and specificity described above, since they are related to the initial position of the pathogenic and neutral mutants before random sampling. +++, very influenced; 0, no effect.
(XLS)

S10 Table Plasmids used. ^a^ Mutation and deletion nomenclature according to the human genome variation society (http://www.hgvs.org/mutnomen/). ^b^ The BRCA1 cDNA used in Millot et.al [11], corresponds to the AY888184.1 GenBank sequence, slightly modified in this study (see the Methods section).
(XLS)

S11 Table Yeast strains used. ^a^ "mCherry" was omitted in the main text. For instance, the P1859R-mCherry strain was referred to as "P1859R".
(XLS)

S12 Table Average CDF of the probability system of classification when using the standard method. See S6C Fig for details. The 272 middle values of the average CDF, derived from the Colony Size assay, were removed to simplify the table. Colored numbers indicate the 5-class nomenclature (S1 Table): dark blue, class 1; light blue, class 2; pink, class4; red, class 5. This table was used to attribute the probabilities of pathogenicity depicted in Fig 2B. For instance, in the Colony Size assay, the median of P1776H is 12,253 cells per colony. The closest best cut-off value of this median belongs to rank 8, meaning that, when using the standard method, the probability of pathogenicity attributed to P1776H is 0.00475. Of note, if a best cut-off value is not repeated, among the 2,000 best cut-off values used to derive the CDF, then the associated probability is 1 / 2,000 = 0.0005 for this value. This indicates the minimal probability incrementation between two non repeated consecutive best cut-off values in the CDF (probability unit). In the Yeast Localization assay, no variant can be classified as class 5 due to a lack of unrepeated values at the "pathogenic" side of the best cut-off distribution (the highest cut-off value, 0.2151616, is present 97 times, leading to a probability of 0.0485 and an average cumulative probability of 0.97550 in the CDF, which is inferior to the 0.99 probability threshold of the class 5)).
(XLS)

S13 Table Basic and corrected probability of pathogenicity when using the standard method. See Fig 2B for details. Values in the "Probability" columns are those in Fig 2B. Mutations are ordered as in Fig 2B to facilitate comparisons. Odds in favor of pathogenicity are the ratio p_i_ / (1—p_i_), with p_i_ being the probability of pathogenicity of the variant i. The Liquid Medium and Yeast Localization assays were not included in the combined odds, since the Liquid Medium and Colony Size, as well as the Spot Formation and Yeast Localization, are not independent assays. Combined probabilities of pathogenicity result from the ratio O_i_ / (1 + O_i_), with O_i_ being the combined odds of the variant i. Probabilities were also corrected according to nneutral + npathogenic = 40 and a = 2 (see the S1 Text). CS, Colony Size assay; SF, Spot Formation assay.
(XLS)

S14 Table Basic and corrected probability of pathogenicity when using the standard with reference method. See S13 Table for details.
(XLS)

S15 Table Basic and corrected probability of pathogenicity when using the MWW method. See S13 Table for details.
(XLS)

S16 Table Quantitative analysis of S24 Fig. Framed numbers highlight the differences with the results obtained using the probability system of classification (S6 Table).
(XLS)

S17 Table Effect of functional assay parameters upon the quantile system of classification. Sensitivity and specificity are defined here as the distance between the best cut-off fluctuation and the position of the pathogenic and neutral mutations, respectively, which reflects the accuracy of the quantile system of variant classification. For instance, the MWW method exhibits a null sensitivity in variant classification if the p values of the pathogenic mutations are in the grey area (class 3), and shows a maximal sensitivity in variant classification if the p values are in the pink area (class 4) or beyond (class 5). Misclassification is defined as class 4 or 5 for the neutral mutations and class 2 or 1 for the pathogenic mutations. See S9 Table for further details. The framed text indicates differences, as compared to the probability system of classification (S9 Table)
(XLS)
