# Supplementary material for: Functional Assessment of Genetic Variants with Outcomes Adapted to Clinical Decision-Making
Source: PLoS Genet. 2016 Jun 6;12(6):e1006096. doi: 10.1371/journal.pgen.1006096 (PMC4894565; doi:10.1371/journal.pgen.1006096)
Supplement: S1 Supporting Information — (PDF) [file pgen.1006096.s001.pdf]

Supplementary Methods

Outline

1. Definitions
2. Symbols frequently used
3. Colony size and Liquid Medium assays in glucose media
4. Western blot
5. The ProClass toolbox developed for the probabilistic classification of experimental data
6. Experimental data assembling
7. The MWW method
8. Sensitivity and specificity computation using the standard method
9. Difference between the standard method and the standard with reference methods
10. Sensitivity and specificity computation using the MWW method
11. Best cut-off, best sensitivity and best specificity computation
12. Bootstrap procedure A to define three different fluctuations of the best cut-off
13. Quantile computation after bootstrap procedure A
14. Probability system of classification
15. Exact best cut-off distribution
16. Properties of the probability of pathogenicity
   16.1. Associated risk
   16.2. Paucity of experimental data impairs the variant classification
   16.3. Paucity of bootstrap performed impairs the variant classification
   16.4. Accuracy of the probability system (exact distribution)
   16.5. Accuracy of the probability system (bootstrap)
17. Combined probability of pathogenicity
18. Independent functional assays
19. Corrected probability of pathogenicity
20. Incorporation of the probability of pathogenicity into posterior probability models (Bayesian inference)
21. The WT reference
22. Procedure to include new data (VUS) in the validated Colony Size, Liquid Medium, Spot Formation or Yeast Localization assays
23. Procedure to adapt the classification model to other functional assays
24. Advantages of these procedures compared to the 2-component models of variant classification
25. Limits of the nonparametric model (MWW method and probability system)
26. Procedure to adapt the model to other experiment system requiring a decision-making based on cut-off
27. The quantile system is not adapted to variant classification
   27.1. Presentation of the quantile system and variant classification
   27.2. The quantile system improves the variant classification when data is lacking
28. References

1. Definitions

<u>Mutant, mutation</u>: in this manuscript, these terms are equivalent to "variant", with no pathogenic connotation.

<u>Ties:</u> statistical term used to designate "identical values".

<u>WT reference:</u> backbone cDNA sequence carrying each assessed variant.

<u>Cut-off:</u> value that allows separation of the mutations into two categories: pathogenic or neutral. Depending on the functional assay, the pathogenic category lies above or below the cut-off, with the neutral category in the opposite position.

<u>Sensitivity:</u> proportion of pathogenic mutations correctly classified. In functional assessment, this is equivalent to the proportion of pathogenic mutations in the pathogenic area. This area lies above or below the cut-off, depending on the assay used.

<u>Specificity:</u> proportion of neutral mutations correctly classified. In functional assessment, this is equivalent to the proportion of neutral mutations in the neutral area. This area lies above or below the cut-off, depending on the assay used.

<u>Accuracy</u>: proportion of mutations correctly classified.

Best cut-off: cut-off value associated with the Youden's index (see **S2 Fig**).

Best sensitivity and specificity: values associated with the best cut-off, and thus with the Youden's index. The term "best" is frequently omitted as the sensitivity and specificity of an assay is always the best among the possible sensitivities and specificities.

Experimental data: data from experiments, as opposed to data from bootstrap analysis, which corresponds to a computer-assisted sampling of the experimental data.

Experimental best cut-off: best cut-off obtained from experimental data, as opposed to the best cut-off obtained from bootstrap analysis.

Experimental sensitivity, specificity and accuracy: best sensitivity, best specificity and best accuracy obtained from experimental data and associated with the experimental best cut-off, as opposed to the sensitivity, specificity and accuracy of the probability systems of classification, obtained after bootstrap analysis.

Initial sensitivity and specificity: equivalent to experimental best sensitivity and specificity. The term "initial" can be used to designate the sensitivity and specificity of the data (experimental or theoretical), since they are computed using the initial position of the neutral and pathogenic mutations, before bootstrap analysis.

Standard method: medians of the mutants are used to find the best cut-off.

Standard with reference method: as in the standard method, except that the best cut-off identified is divided by the median of the WT BRCA1 reference. Thus, the best cut-off is a value relative to the BRCA1 median value, with no unit. In fact, the standard with reference method is not different from the standard method for the experimental data. However, these methods differ during bootstrap analysis, since the raw and relative best cut-offs fluctuate differently.

Raw best cut-off: the term "raw" indicates that the best cut-off value is not divided by the WT BRCA1 median. Therefore, the raw best cut-off has the unit of the experimental data (e.g., cells per colony in the Colony Size assay).

Relative best cut-off: The term "relative" indicates that the best cut-off value is divided by the WT BRCA1 median.

MWW method: as in the standard method, except that each mutant is not represented by a median value but by a p value, as explained in **S4 Fig**.

CDF: cumulative distribution function, see **S6 Fig**.

Probability system of classification: system that uses the fluctuation of the best cut-off to derive probabilities of pathogenicity for each assessed variant (**S6 Fig**). Such probabilities allow the use of a five-class nomenclature to classify variants, as shown in **S1 Table**. Of note, the system is based on an average CDF.

Sensitivity of the probability system of classification: proportion of pathogenic mutations within the class 4 or 5 (see **Fig 2B**). This sensitivity has to be distinguished from the experimental sensitivity.

Specificity of the probability system of classification: proportion of neutral mutations within the class 1 or 2 (see **Fig 2B**). This specificity has to be distinguished from the experimental specificity.

Probability unit: lowest potential incrementation within the average CDF. In an exact distribution, this unit is equal to 1 / $n_{best\ exact}$ if at least three consecutive best cut-off values from the exact best cut-off distribution are not repeated. Otherwise, the value of the unit is higher. In an approximate distribution (bootstrap), this unit is equal to 1 / $n_{bootstrap}$ if at least three consecutive best cut-off values from the best cut-off distribution are not repeated. Otherwise, the value of the unit is higher.

Quantile system of classification: this system is an alternative to the probability system of classification. It is a very simple approach to generate probabilistic classifications.

However, as demonstrated below, this system is not adapted to the classification of variants.

System of classification: designates either the probability or the quantile system.

Accuracy of a system of classification: capability of the probability or quantile system to attribute a class 1 or 2 to the neutral mutations, and a class 4 or 5 to the pathogenic mutations. Of note, a completely accurate system never attributes the class 3 and never misclassifies variants. A system fully inaccurate attributes the class 3 only, or totally misclassifies the neutral and pathogenic variants.

Classification model: computational model that combines a method of best cut-off computation (standard, standard with reference or MWW method) and a system of classification (probability or quantile system).

2. Symbols frequently used

$n_{neutral}$	number of neutral mutations
$n_{pathogenic}$	number of pathogenic mutations
n_{mutant}	number of values within every mutant (implicating an equal number of values between each mutant)
n_{BRCA1}	number of values in the WT BRCA1 reference
$n_{bootstrap}$	number of bootstraps performed to estimate the best cut-off fluctuation
n_{best}	number of best cut-off values obtained after bootstrap ($n_{best} = n_{bootstrap}$)
n_{diff}	number of different best cut-off values obtained after bootstrap
$n_{best\ exact}$	number of best cut-off values in the exact best cut-off distribution (equal to the number of sampling possibilities)
$n_{exact\ diff}$	number of different best cut-off values in the exact best cut-off distribution

3. Colony size and Liquid Medium assays in glucose media

All of the clones from the Colony Size assay were assessed in glucose media to control the absence of any intrinsic growth defect, which would disturb the classification of the pathogenic missense mutations (**S3B Fig**). The rare clones, 10% below the median of the BRCA1 or Vector cells, after glucose induction, were removed from the study before analysis in galactose media.

As for the Colony Size assay, all of the clones from the Liquid Medium assay were assessed in glucose media to control the absence of any intrinsic growth defect (**S8B Fig**). Of note, the 126 clones used in the Colony Size and Liquid Medium assays were the same, except for 2 clones, due to the screening in glucose media.

4. Western blot

Western blots were performed as previously described [11]. One among three independent clones from each category was selected for analysis. Membranes were probed with an anti-BRCA1 monoclonal antibody (MS110, Calbiochem, Billerica, MA, USA, 1:200 dilution), then with a secondary peroxidase-conjugated anti-mouse antibody (Jackson Immunoresearch, West Grove, PA, USA, 1:10,000 dilution). To control for loading variation, stripped membranes were probed with an anti-Tubulin antibody (YL1/2, AbD serotec, Oxford, UK, 1:2,000 dilution) followed by a secondary peroxidase-conjugated anti-rat antibody (Jackson Immunoresearch, 1:5,000 dilution), or with an anti-β actin antibody (8224, abcam, 1:5,000 dilution) followed by a secondary peroxidase-conjugated anti-mouse antibody (Jackson Immunoresearch, 1:10,000 dilution). The ImageJ software was used to quantify signal intensities in every lane (full lanes quantified).

Western-blot analyses were performed to ascertain that growth recoveries were not related to any defect in BRCA1 protein expression (**S20 Fig**). In contrast, mutations showing the largest

number of cells per colony were associated with an increase in protein levels. This confirms what we previously reported for the Y1853X mutation and truncated forms of BRCA1 [11], and suggests that protein levels, clearly higher than the WT BRCA1 control level, predict a classification as pathogenic by the Colony Size, Liquid Medium, Spot Formation or Yeast Localization assay.

5. The ProClass toolbox developed for the probabilistic classification of experimental data

The Probabilistic Classification (ProClass) toolbox compiles the R codes [29] and raw data used in this article. The toolbox is designed with the purpose to facilitate the analysis of most kind of functional assay data, and to provide a probabilistic classification of variants. This toolbox is also adapted to other experimental data, provided that they include at least one positive and one negative control. ProClass is available on line at: http://xfer.curie.fr/get/tvsjyy4dUno/ProClass_toolbox.zip. The following sections 6 to 14 describe the computational procedures used in ProClass. Sections 22 and 23 explain how to integrate ProClass during functional assessment.

6. Experimental data assembling

As mentioned in the description of the functional assays, three independent transformants, also referred to as "clones", were selected for each transformation. This means that each strain, described in **S11 Table**, is represented by three clones. For the Colony Size assay, the 40 missense mutations were separated into four batches of 10 mutations. In the first batch, the three clones from each mutation were analyzed in three independent experiments. The three clones from the WT BRCA1 and the Vector strain were systematically used as a control. This gave 9 colony size values for each WT BRCA1, mutated BRCA1 or Vector control strain in the first batch. At this stage, no normalization was performed, meaning that the dispersion of the 9 values includes both the inter-experiment and the inter-clonal variation. The same was performed for the three other batches of 10 missense mutations, with the WT BRCA1 and the Vector strains as a control. Next, to assemble the results from the four batches, the following formula was applied: $x_{ijk} \times \tilde{x}_{BRCA1\ batch\ 1} \,/\, \tilde{x}_{BRCA1\ batch\ k}$, with x_{ijk} being the colony size value of the clone i (i = 1 to 3) in the experiment j (j = 1 to 3) for the batch k (k = 2 to 4), and with $\tilde{x}_{BRCA1\ batch\ 1}$ and $\tilde{x}_{BRCA1\ batch\ k}$ being the median value of the 9 colony size values from the WT BRCA1 strain in the batch 1 and k respectively. This means that, in batch 2, 3 and 4, the values were adjusted such that the median value of the 9 WT BRCA1 colony size values is equal to the WT BRCA1 median value of batch 1. This also means that the inter-batches variation was not considered. For the WT BRCA1 and Vector strains, the 9 adjusted values of the 4 batches were kept, meaning that the final distribution of these two strains was composed of 36 values.

The same method was applied to the Liquid Medium assay. Of note, the clones used in the Colony Size assay were mostly the same used in the Liquid Medium assay, which allows the comparison of the relative efficiency of these two assays. For the Spot Formation assay, the same method was used, except that the three clones from each strain were assessed once in independent experiments, which led to 3 instead of 9 values. Finally, after adjusting and assembling the values from the four batches, the distributions of the mutant and WT BRCA1 strains were composed of 3 and 12 values respectively. No Vector control strain was used for this assay ("no spot formation" is the theoretical negative control). For the Yeast Localization assay, the same method as for the Spot Formation assay was applied, but without data normalization, due to the WT BRCA1 values close to zero, which otherwise would severely amplify data variation. Of note, the images used in the Spot Formation assay were the same

used in the Yeast Localization assay, which allows the comparison of the relative efficiency of these two assays.

7. The MWW method

The distribution of each mutant was compared to the distribution of the WT BRCA1 using the Mann-Whitney-Wilcoxon (MWW) test. The p value of this test gives the probability to obtain the observed overlap of the two compared distributions, in the random sample, assuming that the two distributions are identical in the population from which is performed the sampling (statistical population). The null hypothesis H_0 is $P(x_{mutant\ i} > x_{BRCA1\ j}) = P(x_{mutant\ i} < x_{BRCA1\ j})$. If H_0 is true, this means that the probability $P(x_{mutant\ i} > x_{BRCA1\ j})$, of having a mutant value $x_{mutant\ i}$ above a BRCA1 value $x_{BRCA1\ j}$, is equal to the probability $P(x_{mutant\ i} < x_{BRCA1\ j})$, of having a mutant value $x_{mutant\ i}$ below a BRCA1 value $x_{BRCA1\ j}$. In an upper-sided MWW test, the alternative hypothesis is that the mutant values tend to be above the BRCA1 values, which is written as $P(x_{mutant\ i} > x_{BRCA1\ j}) > P(x_{mutant\ i} < x_{BRCA1\ j})$ [30]. Importantly, the p values computed here are not used to reject or not reject the null hypothesis of the test, but to quantify the overlap between the mutant and the WT BRCA1 distributions (**S4 Fig**). This defines relative positions of the mutant distributions using the WT BRCA1 distribution as a reference position. Thus, the assumptions necessary for the MWW test [30] are not required here. Relative positions are limited to the environment of the WT BRCA1 distribution. Indeed, pathogenic variants showing no overlap with the WT BRCA1 distribution have the same relative position (same p value), even if the functional assay identifies differences between them. Normal approximation and continuity correction were systematically applied to anticipate the presence of identical values (ties). For that, the R function used was wilcox.test(..., exact=FALSE, correct=TRUE). Of note, using these parameters, an increasing number of ties has a tendency to decrease the p value computed. With $n_{mutant} = 9$ and $n_{BRCA1} = 36$ (Colony Size and Liquid Medium assays), the p values of the MWW test theoretically range from 2.3e-6 to 1 without ties, and from 2e-11 to 1 with a maximum number of ties. With $n_{mutant} = 3$ and $n_{BRCA1} = 12$ (Spot Formation and Yeast Localization assay), the p values of the MWW test theoretically range from 0.0058 to 0.9962 without ties, and from 0.00014 to 1 with a maximum number of ties. In addition, particular results have to be mentioned when using correct= TRUE and one-tailed tests. For instance, the p value obtained for two distributions that perfectly overlap, (e.g., wilcox.test(1:3, 1:3, alternative = "less", exact=FALSE, correct=TRUE)) is slightly upper than 0.5. This has no consequence in variant classification, since p values are used as relative positions, as long as the number of values per variant remains identical. In addition, the p value obtained for two distributions that show the same unique value (e.g., wilcox.test(c(1,1,1), c(1,1,1), alternative = "less", exact=FALSE, correct=TRUE)) is equal to 1. In such extreme situations, it is recommended to use correct=FALSE.

For the Colony Size, Liquid Medium and Yeast Localization assays, upper-sided MWW tests were performed (the hypothesis being that the distributions of the pathogenic mutations are above the distribution of the WT BRCA1 reference). The R function used was wilcox.test(WT reference values, mutant i values, alternative = "less", exact=FALSE, correct=TRUE). For the Spot Formation assay, lower-sided MWW tests were performed (the hypothesis being that the distributions of the pathogenic mutations lie below the distribution of the WT BRCA1 reference). The R function used was wilcox.test(WT reference values, mutant i values, alternative = "greater", exact=FALSE, correct=TRUE). Results are summarized in **S4 Table**.

8. Sensitivity and specificity computation using the standard method

Medians from the mutant distributions were ordered (as in the waterfall representation, **Fig 1A**) and means were computed between every two consecutive medians. These mean values

were defined as all possible cut-offs within the ordered medians. Sensitivity and specificity were computed for each cut-off. Sensitivity corresponded to the proportion of pathogenic mutant medians above (Colony Size, Liquid Medium and Yeast Localization assays) or below (Spot Formation assay) the cut-off. Specificity corresponded to the proportion of neutral mutant medians below (Colony Size, Liquid Medium and Yeast Localization assays) or above (Spot Formation assay) the cut-off. Of note, cut-offs above or below all of the medians were not considered. This means that the sensitivity/specificity of (0, 1) and (1, 0) were excluded from the study. In **S2 Fig**, the confidence intervals of the sensitivity and specificity were computed with the binom.test(..., alternative = "two.sided", conf.level = 0.95) function of R, considering that the theoretical sensitivity or specificity is 100% (p = 1). The receiver operating characteristic (ROC) curve offers a visual representation of the sensitivities and specificities computed. For the x-axis, it is common to use 1-specificity more than specificity, as 1-specificity represents the false positive rate (FPR). The dotted line showed in **S2 Fig** indicates the positive diagonal, for which sensitivities = 1 - specificities. The top left corner of the ROC chart corresponds to sensitivity = 1 and specificity = 1 (1 - specificity = 0), which represents the optimal situation for any given assay.

9. Difference between the standard method and the standard with reference methods

We distinguish the standard method from the standard with reference method. The first uses raw cut-offs, meaning that the cut-off values are not divided by the median of the WT BRCA1 reference (see the definitions above). Thus, the cut-off values have the unit of the experimental data (e.g., cells per colony in the Colony Size assay). In the standard with reference method, cut-off values are divided by the median of the WT BRCA1 reference distribution. Nothing changes between these 2 methods when working with the experimental data (i.e., experimental sensitivity and specificity are the same for both methods). However, results change when performing bootstrap analysis, because in the standard with reference method, the fluctuation of the best cut-off is influenced by the fluctuation of the WT BRCA1 reference, which is not the case in the standard method, that only depends on sampling the neutral and pathogenic mutant values (described below).

10. Sensitivity and specificity computation using the MWW method

The same as the standard method was applied, but for ordered p values (e.g., **Fig 1B**) instead of medians. Thus, the final cut-offs analyzed in ROC curves were the intermediate p values between the ordered mutant p values.

11. Best cut-off, best sensitivity and best specificity computation

In the standard or MWW method, the best compromise between the highest sensitivity and specificity was determined by the nonparametric empirical Youden's index [31-33], which is defined as max[sensitivity_i + specificity_i - 1] for each cut-off i. The Youden's index corresponds to the dot on the ROC curve that maximizes the vertical distance between the positive diagonal and the ROC curve. Such dots were pinpointed as black numbers in **S2 Fig**. The best cut-off was defined as the cut-off of the Youden's index. The best sensitivity and best specificity were defined as those associated with the best cut-off. The standard method and the standard with reference methods generated systematically the same best experimental sensitivity and best experimental specificity, regardless of the data analyzed, since the best cut-off is the same in these two methods, except for the unit (raw best cut-off in the standard method, and relative best cut-off in the standard with reference method).

12. Bootstrap procedure A to define three different fluctuations of the best cut-off

Nonparametric random samplings [34] were performed as follows. The number of sampled values was systematically the number of values available in each mutant and in the WT BRCA1 reference. Thus, for each mutant, n_{mutant} values were randomly chosen with replacement. The same was performed for the BRCA1 distribution, with n_{BRCA1} values randomly chosen with replacement. As an example, using the Colony Size assay, the number of values randomly chosen was $n_{mutant} = 9$ and $n_{BRCA1} = 36$, since respectively 9 and 36 values were available in these distributions. In the Spot Formation assay, the number of values randomly chosen was $n_{mutant} = 3$ and $n_{BRCA1} = 12$, since respectively 3 and 12 values were available in these distributions. Next, using this new set of sampled data, we applied the three standard, standard with reference and MWW methods. In the standard method, medians of the mutant distributions were ordered, raw cut-offs were computed as described above, and the raw best cut-off value associated with the Youden's index of the ROC curve, was saved. In the standard with reference method, the raw best cut-off, identified by the standard method, was divided by the median of the WT BRCA1 reference computed on the new sample data and was saved. In the MWW method, p values for each mutant were computed as described above, and ordered. Next, cut-offs were computed as described above, and the best cut-off value, associated with the Youden's index of the ROC curve, was saved. In each of the three methods, if several cut-offs lead to the Youden's index, the median of these cut-offs was considered as the best cut-off. This procedure was repeated 2,000 times, to obtain 2,000 best cut-off values for each of the three methods. Next, the 2,000 bootstraps were repeated 20 times. This procedure is referred to as "bootstrap procedure A", which, in summary, generated 20 sets of 2,000 best cut-offs for each of the standard, standard with reference and MWW methods.

It is important to mention that, in the standard with reference method, best cut-off values are multiplication factors of the BRCA1 median value. This means that fluctuation of the BRCA1 median is included in the best cut-off fluctuation. For instance, with the Colony Size assay: during the random sampling i, if the raw best cut-off found is $b^*_{raw\,i} = 25{,}000$ cells per colony (the star indicates that the value comes from bootstrapping) and if the BRCA1 median is $\tilde{x}^*_{BRCA1\,i} = 10{,}000$, then the relative best cut-off is $b^*_i = b^*_{raw\,i}/\tilde{x}^*_{BRCA1\,i} = 2.5$. In the random sampling i+1, if $b^*_{raw\,i+1} = 25{,}000$ cells per colony and if $\tilde{x}^*_{BRCA1\,i+1} = 5{,}000$, then the relative best cut-off is $b^*_{i+1} = 5$. These examples highlight the fact that the relative best cut-off values can change, even if the raw best cut-off value of the standard method remains the same.

13. Quantile computation after bootstrap procedure A

In the first set of the bootstrap procedure A, a total of eleven quantiles, 0.1%, 0.5%, 1%, 2.5%, 5%, 50% (median), 95%, 97.5%, 99%, 99.5% and 99.9%, were computed from the 2,000 best cut-off values of the standard method (using the type 7 method of the quantile() function of R). This procedure was repeated for all of the 20 sets. Next, medians of the 20 values from each of the eleven quantiles were computed (of note, these quantiles could have been directly computed from the 20 × 2,000 = 40,000 best cut-off values, but medians of quantiles have the advantage to buffer the fluctuation of extreme quantiles). The same was performed for the standard with reference and MWW methods. Thus, eleven median quantiles were obtained for each of the three methods, shown in **S5 Table**. Of note, the basic (non-studentized pivotal) method was used to obtain the quantiles [35].

14. Probability system of classification

This system is based on rank methods [14]. The following procedure was separately applied to the three standard, standard with reference and MWW methods. In each of the 20 bootstrap

sets, resulting from the bootstrap procedure A, the 2,000 best cut-off values were sorted by ascending or descending order, depending on the position of the neutral and pathogenic sectors in the assay and depending on the method used: ascending order if the lowest values correspond to the neutral sector and the highest to the pathogenic sector (e.g., Colony Size assay + standard method), and descending order in the opposite case (e.g., MWW method, whatever assay is used). Next, the median of the 20 first values, from the 20 sorted sets, was computed, and the same for the next 1,999 subsequent ranks, to obtain a median distribution of the sorted best cut-offs. Next, the probability 1 / n_{best} = 0.0005 was attributed to each of the n_{best} = 2,000 values of the median distribution (see the theoretical example in **S6A-B Fig**). Probabilities were summed in the case of identical values, which finally resulted in n_{diff} values in the median distribution of the sorted best cut-offs (e.g., n_{diff} = 154 in the Yeast Localization assay, using the standard method, as shown in **S6C Fig** and **S12 Table**). From this, two cumulative distribution function (CDF) were generated, by simply carrying out the cumulative sum of the n_{diff} probabilities, with the first CDF that ranged from the probability p_1 to $p_{n\ diff} = 1$ and the second CDF that ranged from 0 to $p_{n\ diff - 1} < 1$. Next, the mean of the two CDF was computed, which resulted in an average CDF that associated a probability ($0 < p_i < 1$) for the n_{diff} different best cut-off values. This average CDF was obtained for each of the Colony Size, Liquid Medium, Spot Formation and Yeast Localization assays and for each of the standard, standard with reference and MWW methods (**S6C-E Fig**).

To attribute a probability of pathogenicity to an assessed variant, using the standard method, the median of this variant was positioned within the raw best cut-off values of the average CDF. Next, the probability of the average CDF, closest to the variant median, was assigned to the variant as a probability of pathogenicity (see the example in **S6B Fig**). The same procedure was applied to the standard with reference method, except that the median of the variant was divided by the median of the WT BRCA1 reference median (i.e., 11,200 cells per colony in the Colony Size assay), to fit the average CDF composed of relative best cut-off values. The same procedure was applied to the MWW method, except that the p value of the variant was used to fit the average CDF. The five-class nomenclature [26] was used to categorize the probabilities of pathogenicity, as in genetic/epidemiological methods (**S1 Table**).

Of note, interest in averaging the two initial CDF lies in the removal of the probabilities 0 and 1 in the resulting CDF, which could create infinite values during subsequent conversions. The second interest is that the average CDF copes with the absence of best cut-off fluctuation. In such situation, the average CDF is represented by a single best cut off value (equal to the experimental best cut-off) which has the probability 0.5. This means that in the absence of best cut-off fluctuation, the classification proposed by a given functional assay, is systematically "variant completely unknown". The convergence towards 0.5 is illustrated using the boundaries of the average CDF. If the number of best cut-off values is n_{best}, then the lowest probability within the CDF is $1/n_{best}$. Thus, in the CDF1, the boundaries of the cumulated probabilities are:

$$\left[\frac{1}{n_{best}}\ ;\ 1\right]$$

n_{best}: number of best cut-off values obtained after bootstrap

And in the CDF2:

$$\left[0\ ;\ 1-\frac{1}{n_{best}}\right]$$

Thus, in the average CDF, the boundaries are:

$$\left[\frac{\frac{1}{n_{best}}+0}{2}\ ;\ \frac{1+1-\frac{1}{n_{best}}}{2}\right]=\left[\frac{1}{2n_{best}}\ ;\ 1-\frac{1}{2n_{best}}\right]$$

And when $n_{best} = 1$, both boundaries of the average CDF are equal to 0.5.

15. Exact best cut-off distribution

In certain situations, it is possible to use the exact best cut-off distribution, instead of performing bootstrap analysis. This exact distribution is defined as all of the sampling possibilities, when sampling with replacement n_{mutant} values among the n_{mutant} available, for each variant, and n_{BRCA1} values among the n_{BRCA1} available for the WT reference. An example is shown in **S21B Fig**. The number of sampling possibilities can be predicted as follows. For one variant, composed of n_{mutant} different values, the number of sampling possibilities is:

$$\binom{2n_{mutant}-1}{n_{mutant}}$$

n_{mutant}: number of values in each mutant (implicating the same number in the different mutants)

With $n_{neutral}$ and $n_{pathogenic}$ variants, composed of the same number of values n_{mutant}, all different, the number of sampling possibilities is:

$$\binom{2n_{mutant}-1}{n_{mutant}}^{n_{neutral}+n_{pathogenic}}$$

$n_{neutral}$: number of neutral mutations
$n_{pathogenic}$: number of pathogenic mutations

Finally, with the inclusion of the WT reference (standard with reference and MWW methods), composed of n_{BRCA1} different values, the number of sampling possibilities is:

$$\binom{2n_{mutant}-1}{n_{mutant}}^{n_{neutral}+n_{pathogenic}}\times\binom{2n_{BRCA1}-1}{n_{BRCA1}}$$

n_{BRCA1}: number of values in the WT BRCA1 reference

As an example, with one neutral and one pathogenic mutation ($n_{neutral} = 1$ and $n_{pathogenic} = 1$), containing two values per mutant ($n_{mutant} = 2$) and two values in the WT BRCA1 reference ($n_{BRCA1} = 2$), the number of sampling possibilities is 27 (27 rows in the table of **S21B Fig**).
In the standard method, the formula shows that, if $n_{mutant} = 1$, then the exact best cut-off distribution corresponds to the experimental best cut-off, and the same for the standard with reference and MWW methods if, additionally, $n_{BRCA1} = 1$ (**S22A Fig**). The formula also shows that the number of sampling possibilities rapidly increases with the number of mutant

values, BRCA1 values, neutral variants and pathogenic variants, which prevents the use of the exact best cut-off distribution in most cases. For instance, in the Colony Size assay, the maximum number of sampling possibilities reaches 2e175 using the standard method and 6e195 using the MWW method. The bootstrap procedure has the advantage to by-pass this combinatory issue. However, it is important to mention that bootstrap affords an approximate distribution of the best cut-off, and that the quality of the approximation increases with the number of bootstraps $n_{bootstrap}$ performed.

Of note, two different sampling possibilities can result in the same best cut-off (for instance, the best cut-off between 10 and 20, and between 5 and 25, is 15 in both cases). Thus, even if the number of mutant values, BRCA1 values, neutral variants and pathogenic variants, are high (generating a high number of sampling possibilities), the final number of different best cut-off values $n_{exact\ diff}$, forming the average CDF of the probability system of classification, can be very low, even within an exact distribution.

16. Properties of the probability of pathogenicity

16.1. Associated risk

The probability of pathogenicity computed is the probability to have the best cut-off value below (ascending average CDF) or above (descending average CDF) the considered mutant, which is related to the probability of misclassification of this mutant, due to the fluctuation of the best cut-off. As an example, in the Colony Size assay using the standard method (ascending average CDF, **S6C Fig**, left panel), a variant with a probability of pathogenicity of 0.99 indicates that the best cut-off variable has a 99% chance to be below the variant median. In this example, the pathogenic area is above the best cut-off (**Fig 1A**), which means that this variant has a 1% probability of being classified as neutral (i.e., best cut-off above the variant median), due to the fluctuation of the best cut-off. In the same manner, again from the Colony Size assay using the standard method, a variant with a probability of pathogenicity of 0.001 indicates that the best cut-off variable has 0.1% chance to be below the variant median, which means that this variant has a 0.1% probability of being classified as pathogenic (i.e., best cut-off below the variant median), due to the fluctuation of the best cut-off, and, thus, has a 99.9% probability of being classified as neutral (i.e., best cut-off above the variant median). For descending average CDF, like in the Colony Size assay using the MWW method (**S6E Fig**, left panel), the reasoning is the opposite. It is important to mention that, contrary to genetic/epidemiological methods, for which the probability of pathogenicity computed measures a direct association of the variant with disease, here the probability of pathogenicity computed evaluates the risk to misclassify a variant, due to the fluctuation of the best cut-off, that depends on the fluctuation of the experimental data when performing the variant assessments. Finally, this probability of pathogenicity estimates the reproducibility of the variant classification obtained, following functional assessment.

16.2. Paucity of experimental data impairs the variant classification

As illustrated in the sections 16.4 and 16.5, the accuracy of the probability system decreases when the best cut-off distribution is composed of only a few different best cut-off values. Ultimately, when the best cut-off distribution is represented by one value (**S22A,C Figs**), the probability of pathogenicity 0.5 (class 3) is systematically attributed to all of the variants, regardless of their relative position. The weak number of different best cut-off values can result from (1) a low number of experimental replicates (**S18G Fig**), (2) a low number of neutral and pathogenic variants incorporated (except if the number of experimental replicates is high), and (3) a weak measurement accuracy leading to many ties (see the case of null ranges in **S19G Fig**). Thus, the probability system has the advantage to penalize functional assays with a paucity of experimental data. This situation is illustrated in the Yeast

localization assay using the standard method (**Fig 2B**). With n_{diff} = 154 (**S6C Fig** and **S12 Table**) none of the pathogenic mutations was classified as class 5.

16.3. Paucity of bootstrap performed impairs the variant classification

As illustrated in sections 16.4 and 16.5, accuracy of the probability system is decreased when the best cut-off distribution is composed of very few different best cut-off values, which is the case if the number of bootstraps performed ($n_{bootstrap}$) is low (**S22B Fig**). In contrast, if $n_{bootstrap}$ is high, then the accuracy of the probability system will only depends on the experimental data (**S22A Fig**).

16.4. Accuracy of the probability system (exact distribution)

The accuracy of a system of classification is defined as the capability to assign a class 1 or 2 to the neutral variant, and a class 4 or 5 to the pathogenic variants. A completely accurate system will never assign the class 3. A system fully inaccurate will assign the class 3 only, or will totally misclassify the neutral and pathogenic variants. In the probability system of classification, the accuracy is related to the probability unit of the average CDF. This probability unit is defined as the lowest potential incrementation within the average CDF or, which is equivalent, as the probability associated with a best cut-off value not repeated in the best cut-off distribution. In an exact distribution, this unit is equal to 1 / $n_{best\ exact}$, meaning 1 / 27 = 0.04 in **S21B Fig**. As shown in section 14, this defines the lowest and highest potential boundaries of the average CDF, derived from the exact distribution:

$$\left[\frac{1}{2n_{best\ exact}}\ ;\ 1-\frac{1}{2n_{best\ exact}}\right]$$

$n_{best\ exact}$: number of best cut-off values in the exact best cut-off distribution (equal to the number of sampling possibilities)

In the probability system of classification, this implies that a neutral variant cannot have a probability of pathogenicity less than 1 / ($2n_{best\ exact}$). In the same manner, a pathogenic variant cannot have a probability of pathogenicity more than 1 - 1 / ($2n_{best\ exact}$). Thus, if $n_{best\ exact} \leq 10$, the probability system will be unable to classify variants as class 2 or 1, because the lower boundary will not be less than 0.05 (**S1 Table**). Such limitations of the probability system of classification are recapitulated below:

Class 1 and 2: $\frac{1}{2n_{best\ exact}} < 0.05 \rightarrow n_{best\ exact} > 10$ (Relations 1)

Class 1: $\frac{1}{2n_{best\ exact}} < 0.001 \rightarrow n_{best\ exact} > 500$

Class 4 and 5: $1-\frac{1}{2n_{best\ exact}} \geq 0.95 \rightarrow n_{best\ exact} \geq 10$

Class 5: $1-\frac{1}{2n_{best\ exact}} > 0.99 \rightarrow n_{best\ exact} > 50$

Because $n_{best\ exact}$ is equal to the number of sampling possibilities, which is dependent on the number of values n_{mutant} in each mutant, the number of values n_{BRCA1} in the WT BRCA1 reference (except for the standard method), the number of neutral mutations $n_{neutral}$ (if n_{mutant} > 1) and the number of pathogenic mutations $n_{pathogenic}$ (if n_{mutant} > 1), this means that the accuracy of the probability system of classification is dependent on these parameters. As an example, in **S21D Fig**, with 1 neutral variant, 1 pathogenic variant, 2 values per variant and 2

values in the WT BRCA1 reference, the number of sampling possibilities is sufficient to allow the class 2 and class 4 classification, but not class 1 and class 5, in the standard with reference method.

Of note, an increasing number of ties (identical values) in the dataset, lowers the accuracy of the probability system of classification. Indeed, ties reduce the number of different best cut-off values, which reduces the interval of the average CDF (boundaries tend towards 0.5). Thus, the lowest $n_{best\ exact}$ values, indicated in Relation 1, are indicative. Moreover, the measurement accuracy has to be considered during functional assessment, to prevent ties.

The fact that two different sampling possibilities can result in the same computed best cut-off, also lowers the accuracy of the probability system of classification. As an example, even with no ties in the data set (**S21A Fig**), the reduced number of different best cut-off, finally obtained with the standard method (5 for 27 sampling possibilities), prevents the classification of variants other than class 3 (**S21C Fig**). This phenomenon, leading to identical best cut-off values, is not predictable, but it highlights again that the lowest $n_{best\ exact}$ values, indicated in Relation 1, are indicative.

16.5. Accuracy of the probability system (bootstrap)

The considerations, developed in the precedent section (16.4), are also valid when using the approximate best cut-off distribution (obtained by bootstrap), instead of the exact distribution, except that the number of bootstraps performed, $n_{bootstrap}$, is an additional parameter that influences the accuracy of the probability system of classification. More precisely, since the bootstrap procedure gives an estimation of the exact best cut-off distribution (**S22B Fig**), the effect of $n_{bootstrap}$ depends on $n_{best\ exact}$.

If $n_{bootstrap} << n_{best\ exact}$, then the lowest potential probability unit is:

$$\frac{1}{n_{bootstrap}}$$

$n_{bootstrap}$: number of bootstraps performed to estimate the best cut-off fluctuation

And the lowest and highest potential boundaries are:

$$\left[\frac{1}{2n_{bootstrap}}\ ;\ 1-\frac{1}{2n_{bootstrap}}\right]$$

If $n_{bootstrap} >> n_{best\ exact}$, then the lowest potential probability unit is:

$$\frac{1}{n_{best\ exact}}$$

$n_{best\ exact}$: number of best cut-off values in the exact best cut-off distribution (equal to the number of sampling possibilities)

And the lowest and highest potential boundaries are:

$$\left[\frac{1}{2n_{best\ exact}}\ ;\ 1-\frac{1}{2n_{best\ exact}}\right]$$

Using the probability system of classification, this implies that a neutral variant cannot have a probability of pathogenicity less than 1 / (2$n_{best\ exact}$), regardless of the number of bootstraps performed. This also implies that the lowest probability of pathogenicity of a neutral variant is overestimated if $n_{bootstrap}$ << $n_{best\ exact}$. In the same manner, a pathogenic variant cannot have a probability of pathogenicity more than 1 - 1 / (2$n_{best\ exact}$), regardless of the number of bootstraps performed. This also implies that the highest probability of pathogenicity of a pathogenic variant is underestimated if $n_{bootstrap}$ << $n_{best\ exact}$.
As an example, in **S21 Fig**, if two bootstraps are performed, then $n_{bootstrap}$ = 2 << $n_{best\ exact}$ = 27. If we consider that the two best cut off values, obtained with the standard with reference method, are different, then the average CDF is represented by the two values 0.25 and 0.75, with the boundaries [0.25; 0.75]. Thus, the probability of pathogenicity attributed to the neutral variant is 0.25, which is overestimated compared to the 0.02 probability given by the exact distribution (**S21D Fig**). In the same manner, the probability of pathogenicity attributed to the pathogenic variant is 0.75, which is underestimated as compared to the 0.98 probability given by the exact distribution (**S21D Fig**).
Of note, if the number of different best cut-off values is much lower than the number of bootstraps performed (n_{diff} << $n_{bootstrap}$), this suggests that the approximate best cut-off distribution is close to the exact one. For instance, in the Liquid Medium assay using the standard method (**S6C Fig**), the $n_{bootstrap}$ = 2,000 bootstraps generated an approximate best cut-off distribution, composed of n_{diff} = 126 different best cut-off values. Thus, in this case, the limitations of the probability system of classification are probably due to $n_{best\ exact}$ rather than $n_{bootstrap}$.

17. Combined probability of pathogenicity

Let us consider one of these three methods: standard, standard with reference or MWW. For each variant, four probabilities of pathogenicity were obtained, from the four Colony Size, Liquid Medium, Spot Formation and Yeast Localization assays (**S13-S15 Tables**). Combining these probabilities provides a final probability of pathogenicity. The model proposed was derived from the one used in genetic/epidemiological methods [15]. Probabilities were converted into odds in favor of pathogenicity using the formula:

$$O_i = \frac{p_i}{1 - p_i}$$

O_i: odds in favor of pathogenicity of the variant i ($0 \leq O_i < +\infty$)
p_i: probability of pathogenicity of the variant i ($0 \leq p_i \leq 1$)

With odds in favor of pathogenicity, the pathogenicity varies between 0 (absolutely neutral) and +∞ (absolutely pathogenic). A variant i, of fully unknown significance, has $p_i = 0.5$ and thus $O_i = 1$. Only independent probabilities can be combined. Thus, probabilities from the Liquid Medium assays were excluded from the computation of the combined probabilities, since the Colony Size and Liquid Medium results were derived from the same yeast clones. In the same manner, probabilities from the Yeast Localization assays were excluded, since the Spot Formation and Yeast Localization results were derived from the same yeast clones and the same microscope picture acquisitions. Next, odds from the Colony Size and Spot Formation assays were multiplied:

$$O_{comb\ i} = O_{CS\ i} \times O_{SF\ i}$$

$O_{comb\ i}$: combined odds in favor of pathogenicity of the variant i ($0 \leq O_{comb\ i} < +\infty$)

And the combined probability of pathogenicity was obtained using the formula:

$$p_{comb\ i} = \frac{O_{comb\ i}}{1 + O_{comb\ i}}$$

$p_{comb\ i}$: combined probability of pathogenicity of the variant i ($0 \leq p_{comb\ i} < 1$)

As a reminder, the accuracy of the probability system of classification is decreased together with n_{mutant}, n_{BRCA1}, $n_{neutral}$ and $n_{pathogenic}$, or together with $n_{bootstrap}$ (see sections 16.4 and 16.5). This means that the probabilities of pathogenicity attributed to the assessed variants will tend towards 0.5. Thus, it is remarkable that an assay, showing low n_{mutant}, n_{BRCA1}, $n_{neutral}$ and $n_{pathogenic}$ values, or for which a low number of bootstraps has been performed, will have a low contribution in the combined probability of pathogenicity. Indeed, when the pathogenicity of a variant tends towards 0.5, the odds tend towards 1, which has a null effect in odds multiplication.

18. Independent functional assays

We estimate that the independence of two functional assays cannot be evaluated *a posteriori*, for instance by comparing the results of these assays. Indeed, in the *a posteriori* evaluation, the notion of independence is based on the hypothesis that the pathogenic variants can have a random position in the pathogenic area of a waterfall distribution. Thus, if we compare two assays, challenged by the same set of pathogenic mutations, the random position of the pathogenic variants, in each assay, should result in a low correlation between the variant medians, which would confirm the independence between the two assays. However, it is known that certain pathogenic mutations have intermediate effects on protein function [36]. Thus, it is difficult to ascertain a random position of the pathogenic mutations in the pathogenic area. Focusing on neutral mutations, rather than on pathogenic mutations, would not solve this issue, since intermediate effects cannot be excluded for neutral mutations. Thus, we propose to evaluate the independence of two assays *a priori*, with the following criteria:

1) The two assessments must have been performed independently. This means that the results from each assay must come from different transfections, different cellular clones, different experimental times, etc. The fact that the plasmids used are not exactly the same reinforces the independence. For instance, in the Spot Formation assay, the plasmids code for the mCherry-BRCA1 fusion protein, but not in the Colony Size assay. Thus, the plasmids used are different in these two assays.

2) What is monitored must be different. Following this, the Colony Size assay and the Liquid Medium assay are not independent, since both monitor the cell growth of yeast cells. If assays are not clearly associated with a protein function, then the measurements must be different. For instance, the Colony Size and the Spot Formation assays are not related to a known function of BRCA1, but the Colony Size assay monitors cell growth, while the Spot Formation assay monitors the formation of a cellular aggregate. Thus, the measurement is different for these two assays.

Based on these criteria, The Colony Size and the Liquid Medium assays cannot be considered as independent, and the same for the Spot Formation and the Yeast Localization assays.

19. Corrected probability of pathogenicity

The fluctuation of the best cut is influenced by the experimental (initial) sensitivity and specificity of a functional assay (**S16 Fig**) but not by the number of neutral and pathogenic mutations used to determine these parameters (**S17 Fig**). This could be problematic in variant

classification, since a 100% sensitivity and specificity of an assay, resulting from 2 neutral and 2 pathogenic mutations assessed, are not reliable. To overcome this, we propose an approach to correct the odds in favor of pathogenicity, that takes into account the number of mutants used to evaluate a functional assay, as follows:

$$O_{cor\ i} = (O_i - 1) \times f_{cor} + 1 \qquad \text{if } O_i \geq 1$$

$$O_{cor\ i} = \frac{1}{\left(\frac{1}{O_i} - 1\right) \times f_{cor} + 1} \qquad \text{if } O_i < 1$$

$$f_{cor} = \frac{n_{neutral} + n_{pathogenic}}{n_{neutral} + n_{pathogenic} + a}$$

$$p_{cor\ i} = \frac{O_{cor\ i}}{1 + O_{cor\ i}}$$

$O_{cor\ i}$: corrected odds in favor of pathogenicity of the variant i
O_i: odds in favor of pathogenicity of the variant i ($0 \leq O_i < +\infty$)
f_{cor}: factor of correction
$n_{neutral}$: number of neutral mutations used in the assay validation
$n_{pathogenic}$: number of pathogenic mutations used in the assay validation
a: integer that modulates the impact of $n_{neutral}$ and $n_{pathogenic}$
$p_{cor\ i}$: corrected probability of pathogenicity of the variant i ($0 \leq p_{cor\ i} \leq 1$)

In this study, we chose a = 2 as a correcting factor (**S13-S15 Tables**), which results in f_{cor} = 0.5 for $n_{neutral} + n_{pathogenic} = 2$, f_{cor} = 0.8 for $n_{neutral} + n_{pathogenic} = 8$, f_{cor} = 0.9 for $n_{neutral} + n_{pathogenic} = 18$ and f_{cor} = 0.95 for $n_{neutral} + n_{pathogenic} = 38$ (**S22D Fig**). Since $n_{neutral}$ and $n_{pathogenic}$ are not distinguished in the correction, this method of correction is not appropriate if $n_{neutral}$ and $n_{pathogenic}$ are strongly unbalanced.

20. Incorporation of the probability of pathogenicity into posterior probability models (Bayesian inference)

The probability of pathogenicity derived from the best cut-off fluctuation can be used to compute a posterior probability of being pathogenic, considering the Bayes' theorem [37]:

$$O_{post\ i} = O_i \times O_{prior\ i}$$

$$O_{prior\ i} = \frac{p_{prior\ i}}{1 - p_{prior\ i}}$$

$O_{post\ i}$: posterior odds in favor of pathogenicity of the variant i ($0 \leq O_{post\ i} < +\infty$)
O_i: odds in favor of pathogenicity of the variant i ($0 \leq O_i < +\infty$)
$O_{prior\ i}$: prior odds in favor of pathogenicity of the variant i ($0 \leq O_{prior\ i} < +\infty$)
$p_{prior\ i}$: prior probability of pathogenicity of the variant i ($0 \leq p_{prior\ i} \leq 1$)

The prior probability represents the probability of pathogenicity of the variant i before any functional assessment. A prior probability of 0.5 can be used in the absence of any prior information. In this case, $O_{post\ i}$ = Oi. Prior probabilities can be provided by the GVGD Align model [38]. Then, the posterior probability of being pathogenic is obtained following:

$$p_{post\ i} = \frac{O_{post\ i}}{1 + O_{post\ i}}$$

$p_{post\ i}$: posterior probability of pathogenicity of the variant i ($0 \le p_{post\ i} \le 1$)

This method can also be applied to the combined odds ($O_{comb\ i}$) and the corrected odds ($O_{cor\ i}$), described above.

21. The WT reference

It is recommended to systematically add a WT reference control during variant assessment [4]. However, this may lead to divergent usage of this reference when interpreting results. The WT reference can either be included in the neutral category or be considered as a particular case, outside of the two neutral and pathogenic categories. The rational of the first choice is that the WT reference is by definition neutral (not pathogenic). The second choice is ruled by three concerns. First, since the WT reference distribution usually contains a number of values larger than in the other neutral distributions, the WT reference could have a strong weight in the final variant interpretation, if incorporated as an additional variant in the neutral category. Second, the WT reference corresponds to the backbone cDNA sequence, present in the expression plasmid, in which the mutations are introduced. In other words, neutral mutations have two differences in their sequences, compared two by two, while they only have one difference compared to the WT reference. Additionally, the variant classification obtained could be modified using another WT reference sequence, because of potential variant-sequence interactions. Thus, the WT reference represents more than an additional neutral variant. Third, variants showing intermediate effects have been reported [36]. This highlights the benefit of the WT reference taken as a special case, beyond the neutral and pathogenic categories, because a reference would be necessary for the identification of neutral variants with intermediate effects.

22. Procedure to include new data (VUS) in the validated Colony Size, Liquid Medium, Spot Formation or Yeast Localization assays

- Download the ProClass toolbox (see section 5).
- Read carefully the *README.doc* downloaded document.
- Request the desired plasmids, presented in this study.
- Generate the plasmids containing the VUS (new batch of variants).
- Perform the experiment as described above. For instance, with the Colony Size assay, test three independent clones in three independent experiments to obtain 9 final values for the VUS. Add the WT BRCA1 reference (plasmid pPT60 or pPT63 depending on the chosen assay), as well as the pathogenic G1706E (plasmid pPT147 or pPT161) and the neutral R1751Q (plasmid pPT119 or pPT120) mutations as a control. Add the Vector control (pJL48 plasmid) for the Colony Size or the Liquid Medium assays. This means that 9 values are also obtained for the WT BRCA1 reference and for each control.
- Consider the results as a new batch and include these results in the downloaded table (e.g., Colony Size data.txt table). For the G1706E, R1751Q and Vector controls, change the name of the new values, like G1706E.bis, in order to prevent the fusion of the new values with the values of G1706E already present in the table. In addition, set these G1706E.bis, R1751Q.bis and Vector.bis controls as "Other.reference" in the "Prior_classif" column.
- Execute the code of the *Code data analysis and representation.doc* file with the adapted settings (for the Colony Size assay, use the *Colony Size data proba 2000x20 type7 replac noref less (MWW).txt* file to generate the probability of being pathogenic using the MWW method).

- In the normalized data obtained, verify that the median of the controls (G1706E.bis, R1751Q.bis and Vector.bis) fit approximately the median of the preexisting values (G1706E, R1751Q and Vector).
- If the medians fit, use the probability of being pathogenic provided in the output results of the executed code. Results can be combined, corrected and included in a posterior probability model, as explained above.
- If the median of the controls (G1706E.bis, R1751Q.bis and Vector.bis) do not fit the median of the preexisting values (G1706E, R1751Q and Vector), a new best cut-off fluctuation has to be generated. For that, remove the ".bis" in the name of the controls, in the data table, and follow the complete instructions provided in the *README.doc* downloaded document.

23. Procedure to adapt the classification model to other functional assays

- Download the ProClass toolbox (see section 5).
- Select several neutral and pathogenic mutations formally classified by genetic/epidemiological methods. Favor, if possible, a similar number of neutral and pathogenic variants.
- Design the experiment. It is counseled to plan independent experiments and to have the WT reference systematically present in each experiment.
- Generate the expression vectors, carrying either the WT reference control, or the different neutral or pathogenic mutations.
- Fix the number of values per mutation that has to be obtained (e.g., 9 values per mutation in the Colony Size assay). This number must be the same for each variant (neutral, pathogenic and unknown). This number must also be systematically respected when subsequently adding neutral and pathogenic mutations, in order to improve the sensitivity and specificity of the functional assay, or when subsequently adding VUS for classification. In addition, the number of values expected for the WT reference control should be high, in order to improve the sensitivity of variant classification (**S18F Fig**).
- Of note, if the data show different numbers of values per neutral, pathogenic and unknown variants, the code of the *Code data analysis and representation.doc* file will reduce the data as explained in the *README.doc* file.
- Perform the experiments.
- Apply the complete instructions provided in the *README.doc* downloaded document.
- Results can be combined, corrected and included in a posterior probability model, as explained above.

24. Advantages of these procedures compared to the 2-component models of variant classification

Recently, statistical models of variant classification have been proposed [7,8]. These models: (1) are parametric (assumption about the distribution of the data in the statistical population), (2) require high statistical skills to analyze the data and fit the model and (3) need to be recomputed when additional VUS are included in the model, for classification purposes.

The model we propose alleviates these constraints. This model: (1) is nonparametric (no assumption about the distribution of the data in the statistical population), (2) does not require statistical skills to be handled and (3), following certain conditions, does not need recomputation when additional VUS are included. Moreover, the probability of pathogenicity computed with our model can be incorporated into posterior probability models (Bayesian inference), as described above, meaning that they can be handled in the manner as the probabilities computed by the 2-component models.

25. Limits of the nonparametric model (MWW method and probability system)

1) The number of values per variants (neutral, pathogenic and UV) must be the same. This allows a balanced contribution of each neutral and pathogenic variant to the best cut-off fluctuation, as well as a correct adequacy between the unknown variants assessed and the best cut-off distribution used to classify them. The R code, available online, manages different number of values but at the cost of loss of information, as the code reduces the number of values from all the variants to the lowest existing in the data.
2) A WT reference must be systematically present in each experiment performed. In addition, the MWW method requires that the WT reference is well embedded in the distribution of the neutral values (e.g., **S5A Fig**, left panel). A WT reference falling outside of the range of the neutral and pathogenic distributions impairs the sensitivity of the functional assay (**S15 Fig**). In such situation, it is recommended to use the standard method instead of the MWW method.
3) It is counseled to have at least 3 values per variant and more than 3 values for the WT reference (**S18 Fig**). In theory, our nonparametric model does not require a minimum number of values. However, the greater the number is, the better is the sensitivity and specificity of the model (see section 16 above). Of note, with a single value per mutation and per WT reference, the fluctuation of the best cut-off is null (the probability of being pathogenic, assigned to each variant, is systematically 0.5).

26. Procedure to adapt the model to other experiment system requiring a decision-making based on cut-off

The procedure described in section 23 can be applied to all situations, based on two categories (applying the standard method) or two categories + a reference category (applying the MWW method), as long as the best cut-off is able to fluctuate during bootstrap computation. If the two categories are divided into subcategories, as "variants" in the pathogenic and neutral categories, then the best cut-off fluctuation will be guaranteed with at least two different values in each subcategory. If the two categories are not subdivided, then the best cut-off fluctuation will be guaranteed with at least two different values in each category. Additionally, in the MWW method, the reference should be composed of at least two different values. Of note, if the data show different numbers of values per categories, the code of the *Code data analysis and representation.doc* file will reduce the data as explained in the *README.doc* file. Such data adjustment has been applied to the siRNA data presented in **Fig 3**. The initial data are made of 864 values for the positive control (siKIF11), 288 values for the negative controls (siGOLGA2 and siGL2) and 12 values for the unknown siRNAs assessed. The code reduces the data to 12 values for siKIF11, siGOLGA2 and siGL2, before any subsequent analysis. The reference category is not concerned by this adjustment.

27. The quantile system is not adapted to variant classification

<u>27.1. Presentation of the quantile system and variant classification</u>

We developed another approach to classify variants, referred to as "quantile system", which is very easy to apply. The quantile system is similar to the "grey zone approach" [39,40] but is extended to n zones. Since the classification as either pathogenic or neutral is dependent on the position of the variant above or below the best cut-off, we reasoned that the farther a variant is from the core of the best cut-off fluctuation, the more robust is its classification as either pathogenic or neutral. The quantile system consists of (1) overlapping the best cut-off fluctuation with the waterfall distribution of the mutants, (2) defining intervals in the best cut-off distribution associated to the five-class nomenclature proposed by Plon et al. (see **S1 Table** and **Fig 1**) and (3) classifying variants according to the position of their median (standard and standard with reference methods) or p value (MWW method) in the 5 intervals.

For instance, in **Fig 1B**, the p value of K45Q is located within the grey area. Thus, the variant is classified as class 3. The quantile system is fully documented in **S23 Fig**. The classification obtained in the four functional assays is depicted in **S24 Fig**. Globally, little differences were observed when comparing the classification obtained with the quantile (**S16 Table**) and the probability systems (**S6 Table**). Of note, the light blue, grey and pink areas depicted in **Fig 1**, **Fig 3A-B** and **S7, S9, S11, S13-S19**, **S25-S27 Figs** correspond to class 2, 3 and 4 of the quantile system, respectively.

<u>27.2. The quantile system improves the variant classification when data is lacking</u>

To detect potential flaws in the quantile system of classification, we recapitulated the analysis of theoretical situations, performed for the probability system (**S25-S27 Figs** and **S17 Table**). The results reveal a major flaw in the variant classification provided by the quantile system, as it does not penalize the paucity of data (**S26B** and **S27 Figs**). This was confirmed with the analysis from an exact best cut-off distribution (**S21F Fig**). In fact, the highest sensitivity, specificity and accuracy of the quantile system is reached when the best cut-off does not fluctuate (**S22C Fig**), which is favored by the paucity of data. In conclusion, the quantile system is not adapted to variant classification. This also suggests that the "grey zone approach" [39,40] is not an efficient method to identify a level of uncertainty within a given dataset.

28. References

27. Huh WK, Falvo JV, Gerke LC, Carroll AS, Howson RW, et al. (2003) Global analysis of protein localization in budding yeast. Nature 425: 686-691.
28. Winzeler EA, Shoemaker DD, Astromoff A, Liang H, Anderson K, et al. (1999) Functional characterization of the S. cerevisiae genome by gene deletion and parallel analysis. Science 285: 901-906.
29. R Core Team (2014) R: A Language and Environment for Statistical Computing. Vienna, Austria: R Foundation for Statistical Computing.
30. Conover WJ (1999) Practical nonparametric statistics third edition: Editions John Wiley & Sons, inc. 584 p.
31. Youden WJ (1950) Index for rating diagnostic tests. Cancer 3: 32-35.
32. Fluss R, Faraggi D, Reiser B (2005) Estimation of the Youden Index and its associated cutoff point. Biom J 47: 458-472.
33. Perkins NJ, Schisterman EF (2006) The inconsistency of "optimal" cutpoints obtained using two criteria based on the receiver operating characteristic curve. Am J Epidemiol 163: 670-675.
34. Kulesa A, Krzywinski M, Blainey P, Altman N (2015) Points of Significance: Sampling distributions and the bootstrap. Nat Meth 12: 477-478.
35. Carpenter J, Bithell J (2000) Bootstrap confidence intervals: when, which, what? A practical guide for medical statisticians. Stat Med 19: 1141-1164.
36. Spurdle AB, Whiley PJ, Thompson B, Feng B, Healey S, et al. (2012) BRCA1 R1699Q variant displaying ambiguous functional abrogation confers intermediate breast and ovarian cancer risk. J Med Genet 49: 525-532.
37. Lindor NM, Guidugli L, Wang X, Vallee MP, Monteiro AN, et al. (2012) A review of a multifactorial probability-based model for classification of BRCA1 and BRCA2 variants of uncertain significance (VUS). Hum Mutat 33: 8-21.
38. Tavtigian SV, Byrnes GB, Goldgar DE, Thomas A (2008) Classification of rare missense substitutions, using risk surfaces, with genetic- and molecular-epidemiology applications. Hum Mutat 29: 1342-1354.

39. Ray P, Le Manach Y, Riou B, Houle TT (2010) Statistical evaluation of a biomarker. Anesthesiology 112: 1023-1040.
40. Cannesson M, Le Manach Y, Hofer CK, Goarin JP, Lehot JJ, et al. (2011) Assessing the diagnostic accuracy of pulse pressure variations for the prediction of fluid responsiveness: a "gray zone" approach. Anesthesiology 115: 231-241.

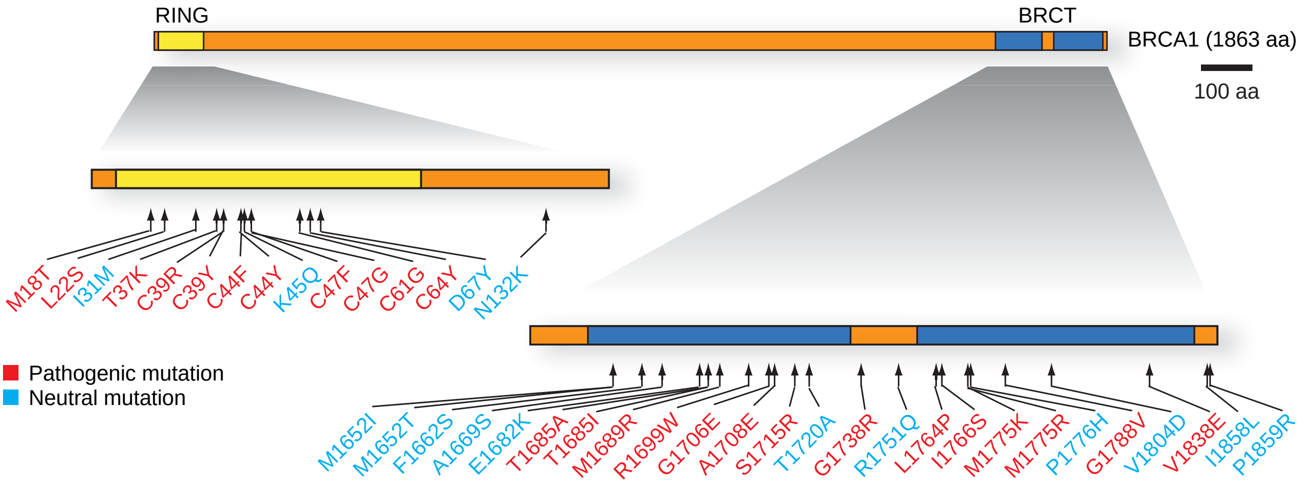

S1 Fig. Position of the BRCA1 missense mutations selected

RING domain (amino acid 8–96); BRCT, BRCA1 C-terminal domains (amino acid 1646–1736 and 1760–1855). Pathogenic and neutral mutations are in red and blue, respectively. Fourteen mutations (3 neutral and 11 pathogenic) map within the RING domain. An additional neutral mutation, N132K, flanks the Cter part of this domain, resulting in 15 mutations located in the Nter extremity of BRCA1. Twenty-five mutations (11 neutral and 14 pathogenic) lie in the BRCT domain, at the Cter extremity of the protein. Of note, the RING domain suffers from a lack of neutral missense mutations classified by genetic/epidemiological methods, explaining why only 3 neutral mutations from our selected panel, lie in this domain. Moreover, no pathogenic missense mutations, between the amino acids 65 and 1684, are documented in the BRCA1 mutation databases (S2 Table). Therefore, this study was restricted to the RING and BRCT domains of BRCA1.

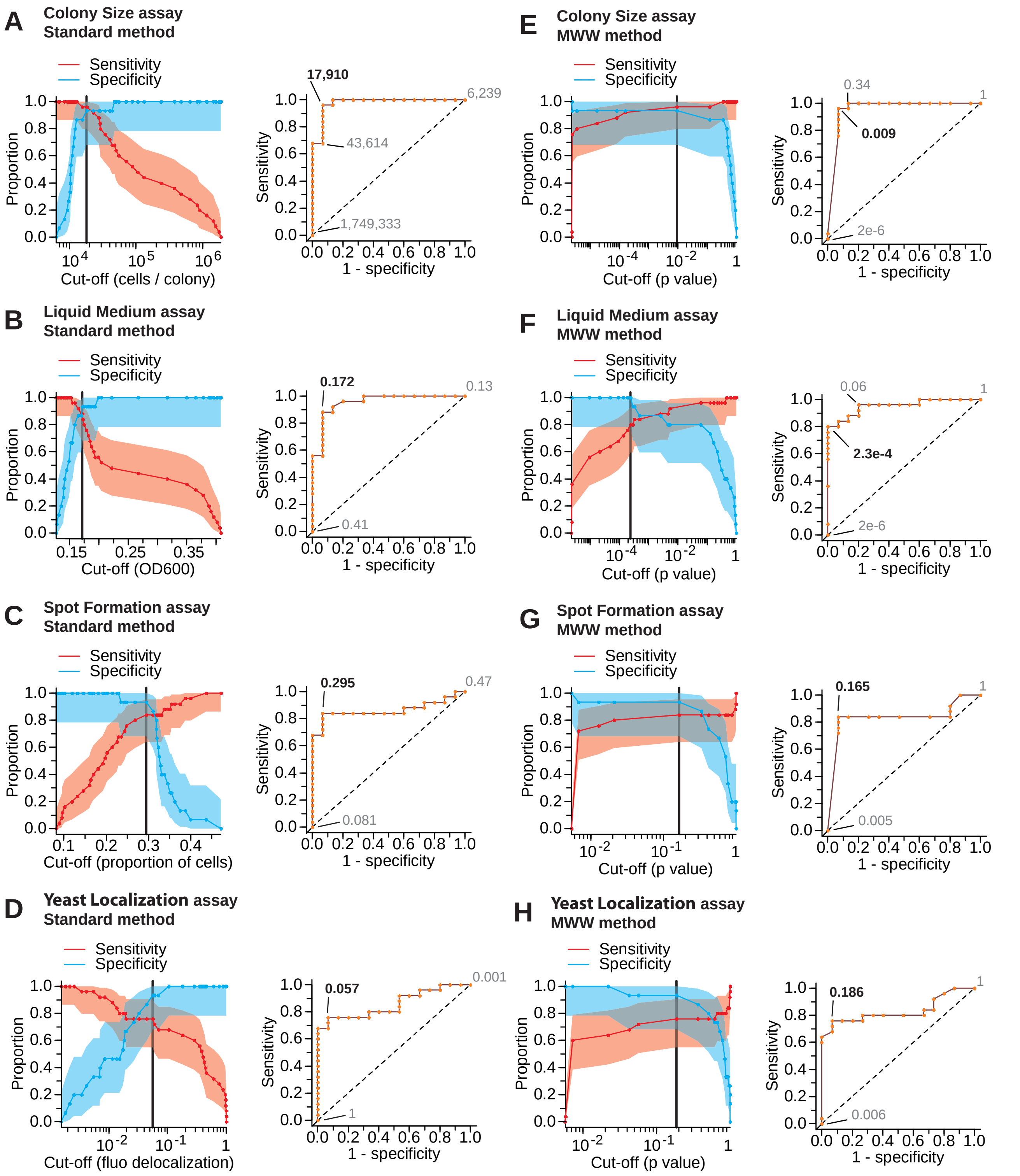

S2 Fig. Experimental best cut-off, experimental sensitivity and experimental specificity of functional assays

(**A-D**) Standard method. The medians of the mutant distributions were ordered (as in the waterfall distribution, Fig 1A) and each average position between two consecutive medians was defined as a cut-off. For example, in Fig 1A, the cut-off between the two first mutations, M1689R and V1838E, was (1,877,333 + 1,621,333) / 2 = 1,749,333 cells per colony. Next, sensitivity was defined as the proportion of pathogenic mutant medians above (for the Colony Size, Liquid Medium and Yeast Localization assays) or below (for the Spot Formation assay) a selected cut-off. The associated specificity was defined as the proportion of neutral mutant medians below (Colony Size, Liquid Medium and Yeast Localization assays) or above (Spot Formation assay) the same selected cut-off. For example, for the cut-off between M1689R and V1838E in Fig 1A, the sensitivity was 1/25 = 4% and the specificity was 15/15 = 100%. Sensitivity and specificity were computed for each cut-off (left panels). Areas surrounding the curves delimit the 95% confidence interval according to the binomial law. The ROC curve (right panel) pinpoints the best cut-off (black number), meaning the cut-off that maximizes both sensitivity and specificity of the assay. Precisely, the best cut-off is the one associated with the highest vertical distance of the ROC curve to the dotted diagonal. This highest vertical distance is referred to as "Youden's index", which is equal to max[sensitivity + specificity - 1]. In other words, the best cut-off is the cut-off of the Youden's index. Other cut-off values are also positioned on the ROC curve (grey numbers). Blue, red and orange dots on the curves of the left and right panels represent the different cut-offs tested. The black vertical bar, in the left panel, pinpoints the best cut-off defined on the ROC curve.

(**E-H**) MWW method. As in **A-D** for mutant p values, instead of mutant medians. In all assays, sensitivity was defined as the proportion of pathogenic mutant p values below a selected cut-off, and the associated specificity was defined as the proportion of neutral mutant p values above the same selected cut-off.

(**A**, **E**) Colony Size assay.

(**B**, **F**) Liquid Medium assay.

(**C**, **G**) Spot Formation assay.

(**D**, **H**) Yeast Localization assay.

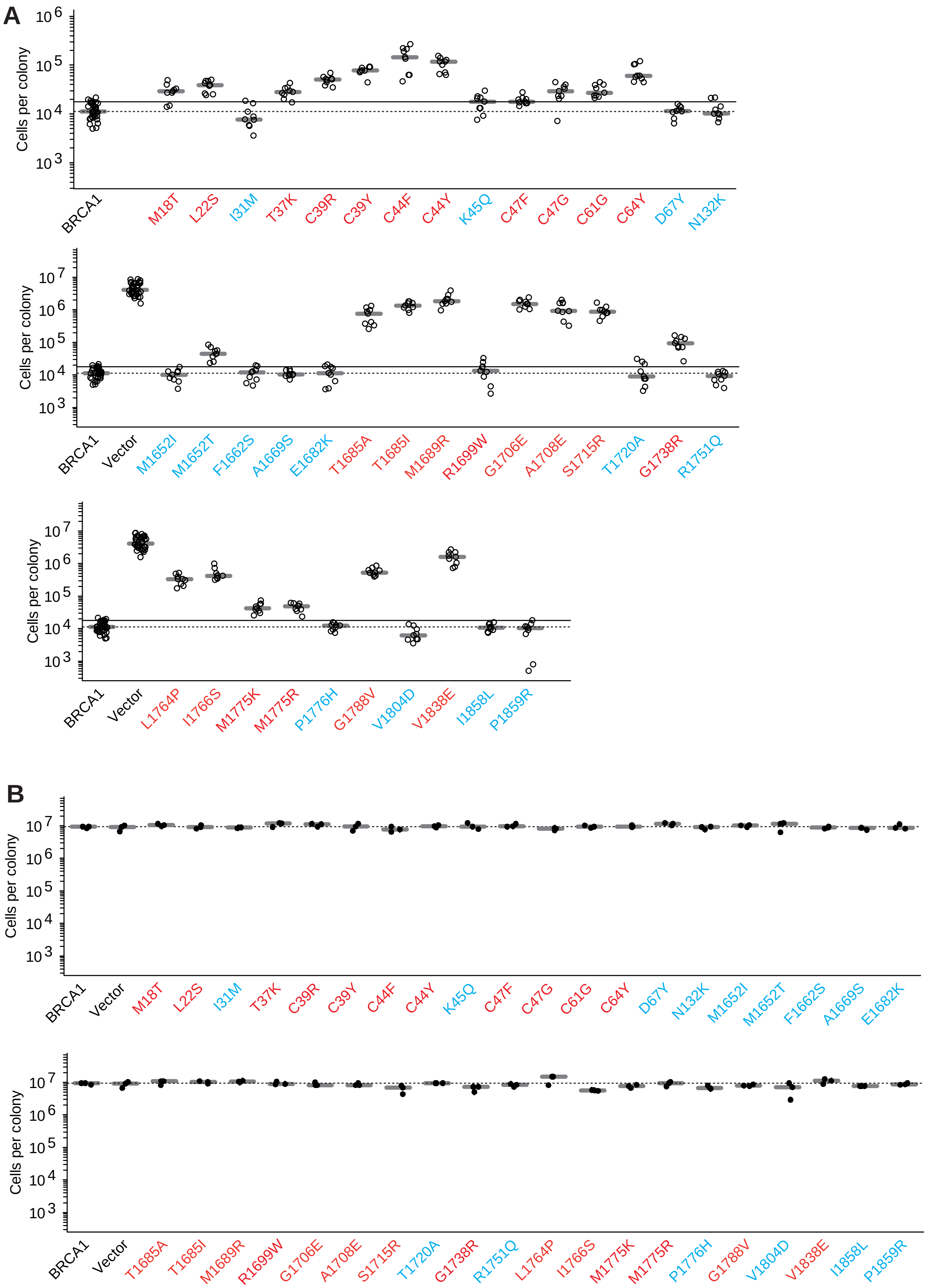

S3 Fig. Supplemental information in the Colony Size assay

(**A**) Dotplot distribution of colony sizes. For each missense variant, the nine represented values result from three independent clones examined in three independent experiments. For the BRCA1 reference and the Vector control, the 36 values result from three independent clones examined in twelve independent experiments (represented in the three panels, except for the Vector values absent in the top panel). Grey bar, median; dotted horizontal line, median of BRCA1; black horizontal line, experimental best cut-off. The top panel (Nter extremity of BRCA1) has a y-axis scale magnified compared to the middle and bottom panels (Cter extremity of BRCA1).

(**B**) As in **A** with glucose instead of galactose media (see the S1 Text) to verify that each clone had no intrinsic growth defect, independent of WT or mutated BRCA1 expression. The three independent clones from **A** were examined in one experiment.

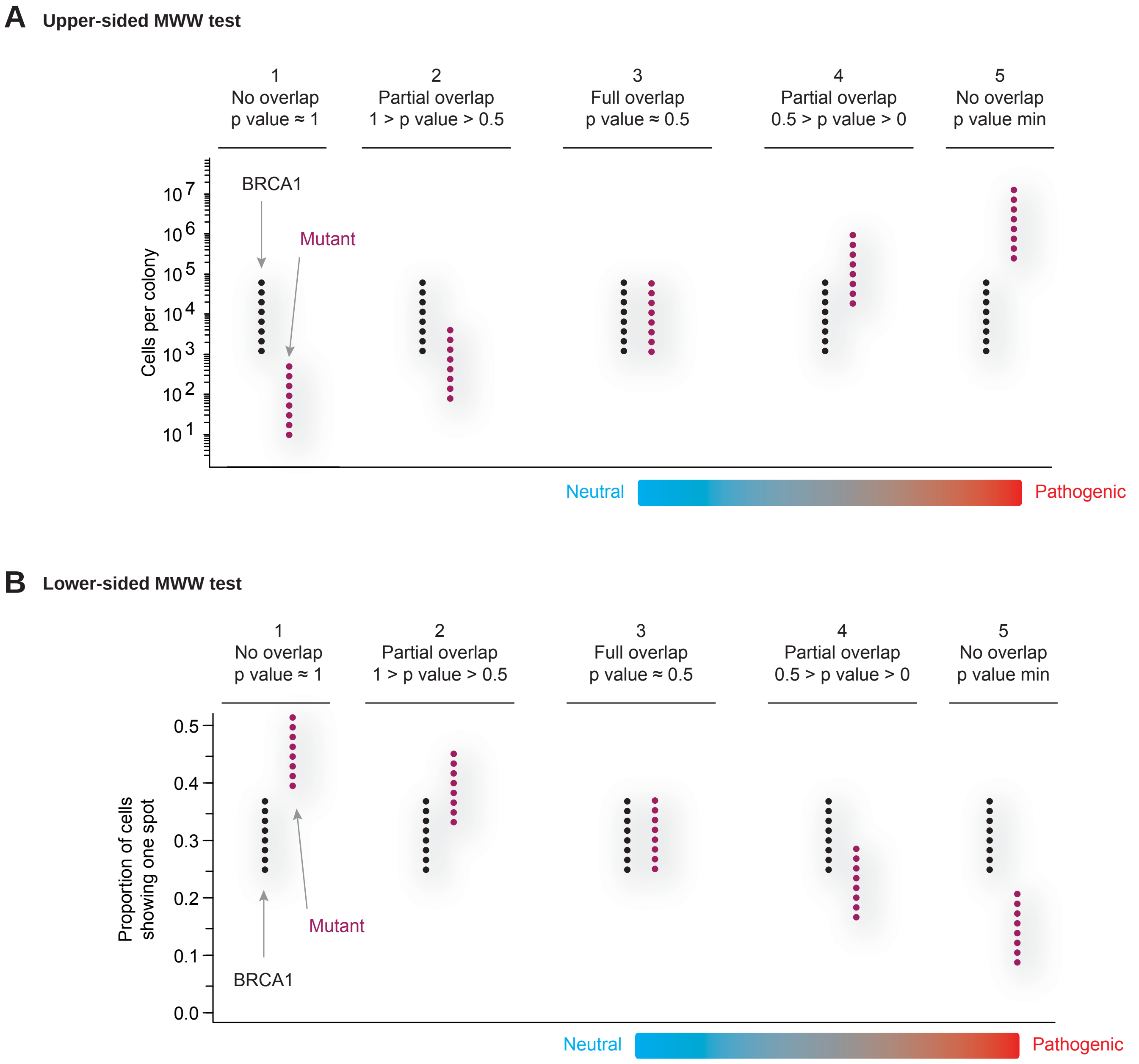

S4 Fig. The MWW method

(**A**) Upper-sided MWW test. The theoretical examples are based on the Colony Size assay but are also valid for the Liquid Medium and Yeast Localization assays. Each distribution of the WT BRCA1 reference (black) and the missense mutation (purple) are composed of 8 theoretical values, represented by 8 dots in the diagram. The p value of the MWW test is used to score the overlap of the mutant and the WT BRCA1 distributions. See the S1 Text for full details. From left to right: (1) when all the mutant values are below the BRCA1 values, the upper-sided MWW test results in a p value close to 1; (2) the p value decreases when the mutant distribution begins to overlap the BRCA1 distribution; (3) the p value is approximately 0.5 when the two distributions completely overlap; (4) the p value continues to decrease when the mutant distribution is above the BRCA1 distribution, with a partial overlap; (5) finally, the p value is lowest when the mutant distribution is fully above the BRCA1 distribution. In theory, neutral and pathogenic mutations should have a p value close to 0.5 and 0, respectively, as depicted by the color scale below the diagram. However, the absolute p value attributed to each variant is not determinant. What is significant is the relative positions between the mutant distributions, indicated by the p values, using the WT BRCA1 distribution as a reference position. The lowest p values represent systematically the pathogenic mutations, and the highest the neutral mutations. Thus, the upper-sided MWW test is used when pathogenic mutations are above the neutral ones in the experimental data.

(**B**) Lower-sided MWW test. All of the theoretical examples shown are based on the Spot Formation assay. As in the upper-sided MWW test, the lowest and highest p values still represent the pathogenic and neutral mutations, respectively, but the pathogenic mutations are below the neutral ones in the experimental data.

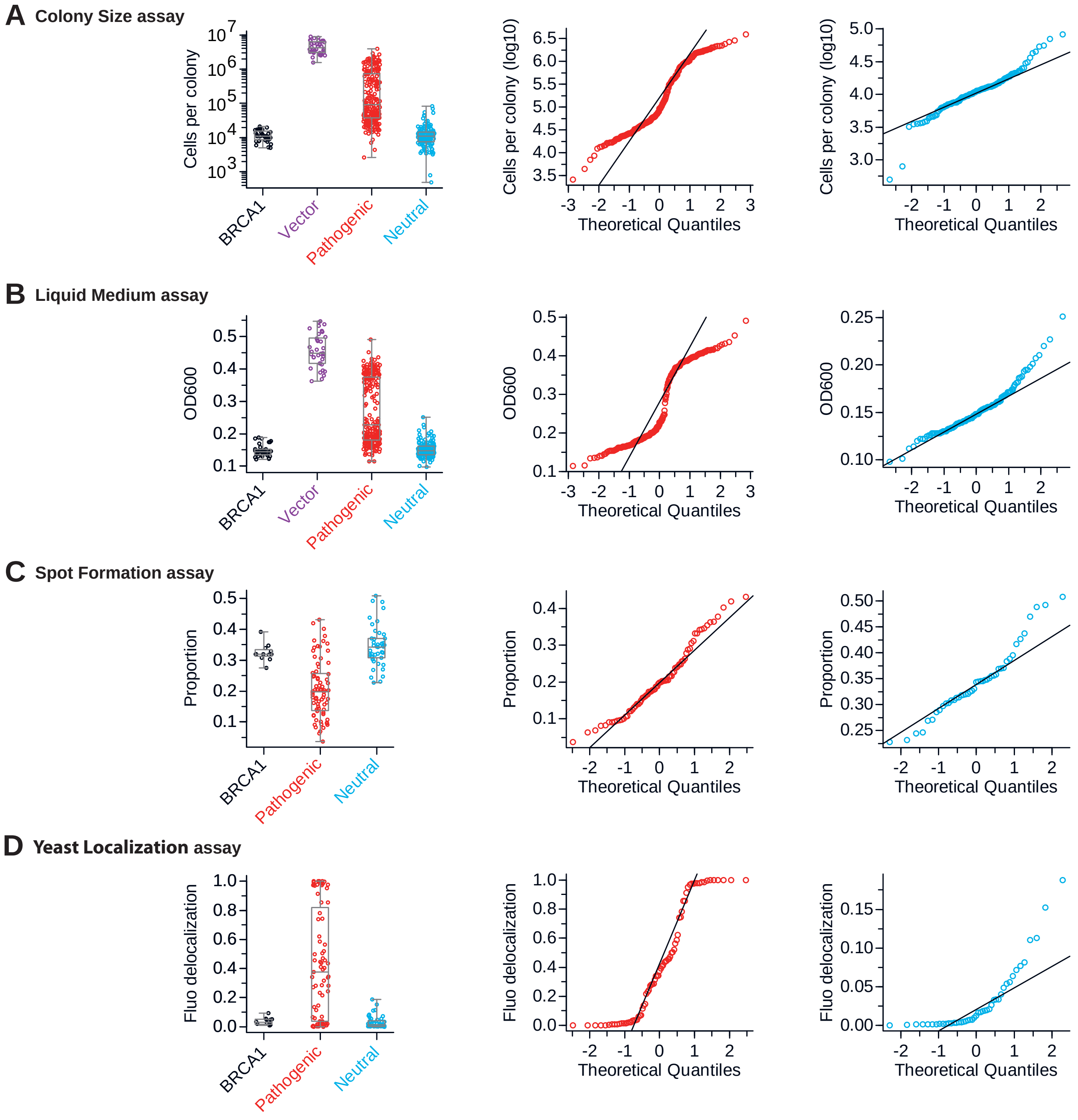

S5 Fig. Distribution of the pathogenic and neutral values

(**A**) Colony Size assay. The left panel exhibits dotplot distributions. Boxplots provide distribution parameters: box central bar, median; box, interquartile range (50% of the distribution); whiskers, extreme values. The middle panel shows the normal Quantile-Quantile (QQ) plot of the pathogenic values. Dots forming a straight line suggest that the values are normally distributed. Black line, straight line through the quantiles 25% and 75%. The right panel shows the normal QQ plot of the neutral values.

(**B**) Liquid Medium assay.

(**C**) Spot Formation assay.

(**D**) Yeast Localization assay.

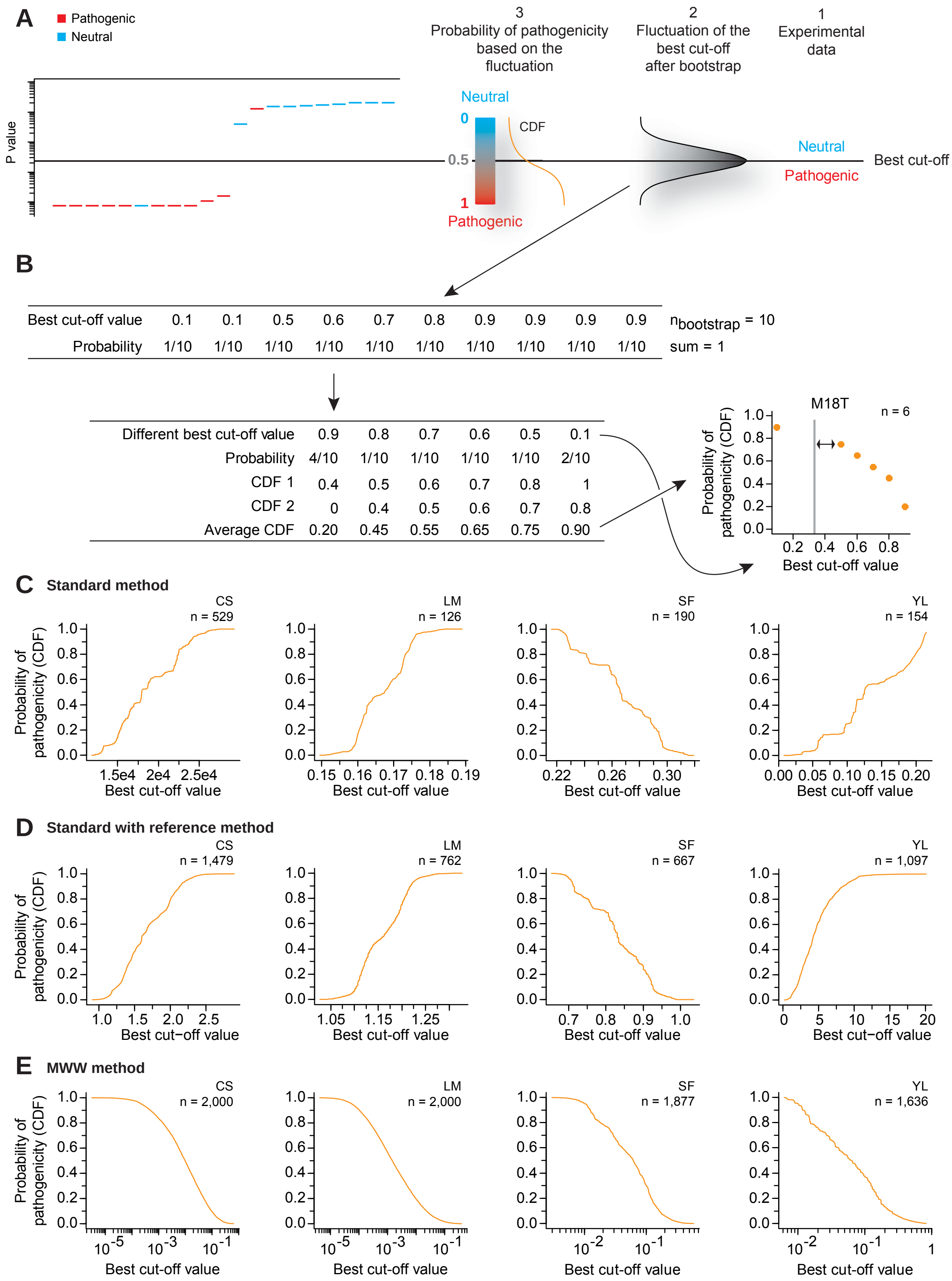

S6 Fig. Description of the probability system of classification

(**A**) As in Fig 2a.

(**B**) Theoretical example showing how the values from the best cut-off fluctuation, derived from the MWW method, are converted into probabilities of pathogenicity. Top table: best cut-off distribution composed of 10 best cut-off values, resulting from 10 bootstraps ($n_{bootstrap}$ = 10). The probability attributed to each best cut-off value was 1 / $n_{bootstrap}$. Bottom table: cumulative distribution functions (CDF) generated from the best cut-off distribution. In this table, probabilities of each repeated cut-off value were summed. For instance, the best cut-off value of 0.9 is repeated 4 times in the top table, leading to a probability of 0.4. The CDF represents the sum of the probabilities present in the second row of the bottom table. Three CDF were computed. The first reaches the cumulated probability of 1. The second begins with the cumulated probability of 0. The third is the average of the two first CDF. This average CDF delivers the probability of pathogenicity used to classify variants. Right panel: plot of the average CDF. To classify a variant (e.g., M18T), the variant p value, derived from the MWW method, is positioned on the x-axis (vertical grey bar). Next, the closest average CDF value is attributed to the variant as a probability of pathogenicity. In this example, the best cut-off value, closest to M18T, is 0.5. Thus, the corresponding probability 0.75 is attributed to M18T.

(**C-E**) Average CDF of the Colony Size (CS), Liquid Medium (LM), Spot Formation (SF) and Yeast Localization (YL) assays, obtained with the standard (**C**), standard with reference (**D**) or MWW method (**E**). The same procedure, described in **B**, was applied to the 2,000 best cut off values obtained for each assay and each method used. The CDF is ascending when the pathogenic mutations are above the neutral ones, and descending when the pathogenic mutations are below. The number of different best cut-off values is indicated (n = 2,000 when no identical best cut-off values within distributions).

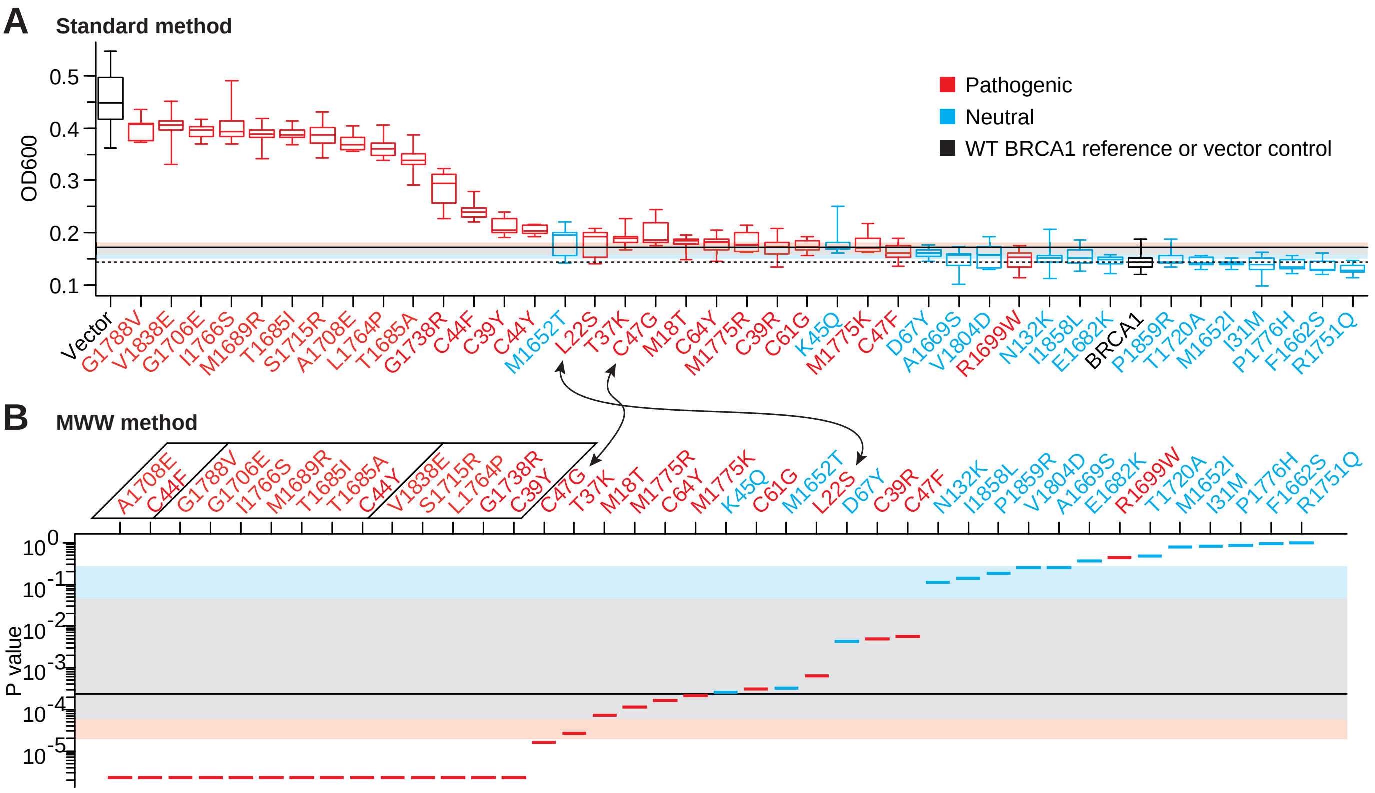

S7 Fig. Relative position of the variants in the Liquid Medium assay and fluctuation of the best cut-off

(**A-B**) As in Fig 1. One OD unit corresponds to 10^8 cells / ml. Arrows pinpoint the ranking of the L22S and C47G mutations, which is improved using the MWW method, as explained in the main text introducing this method. The incoherent ranking observed with the standard method results from L22S that exhibits four values below the experimental best cut-off while C47G has none (S8A Fig).

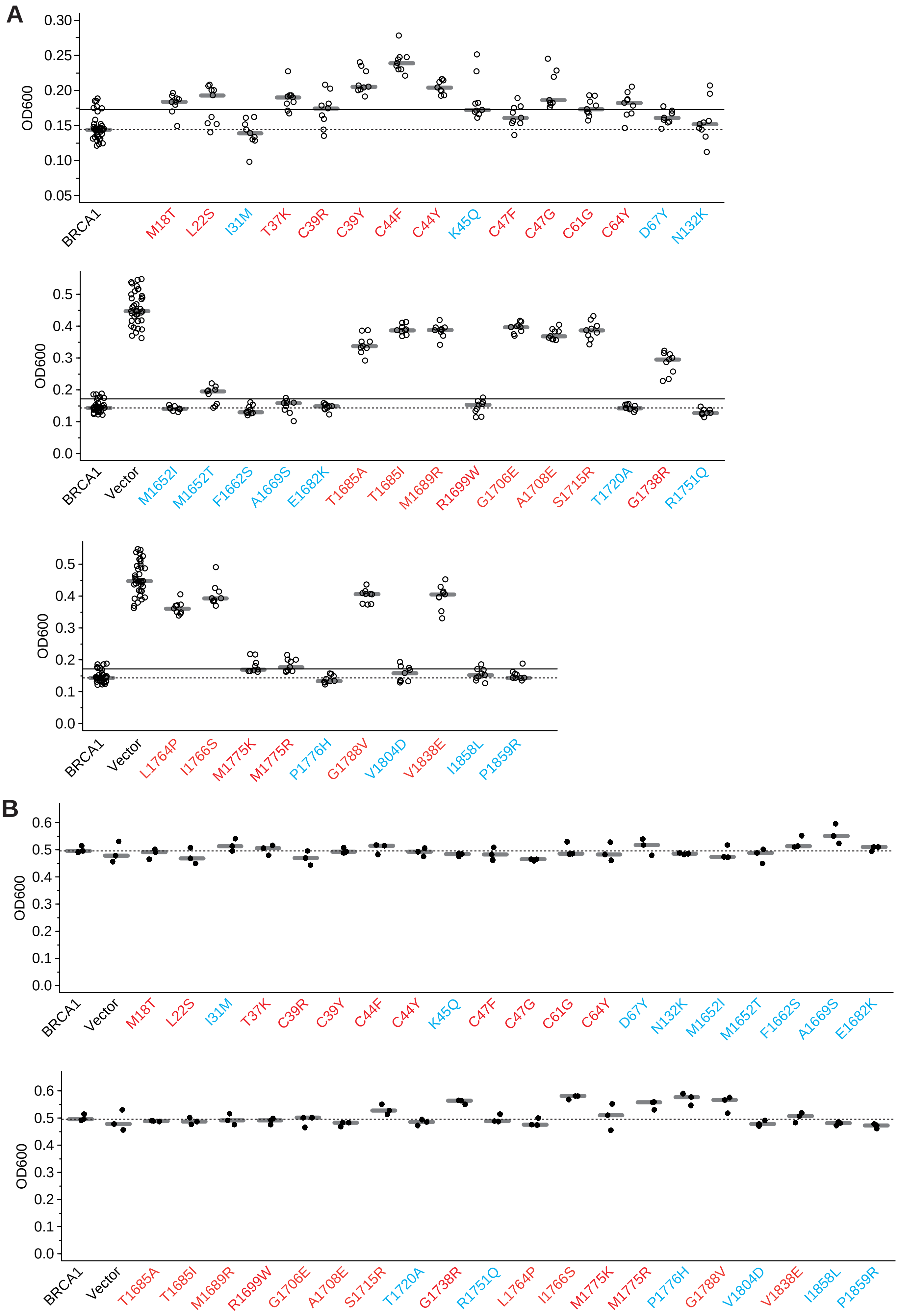

S8 Fig. Supplemental information in the Liquid Medium assay

(**A-B**) Same as for the Colony Size assay (S3 Fig). One OD unit corresponds to 10^8 cells / ml.

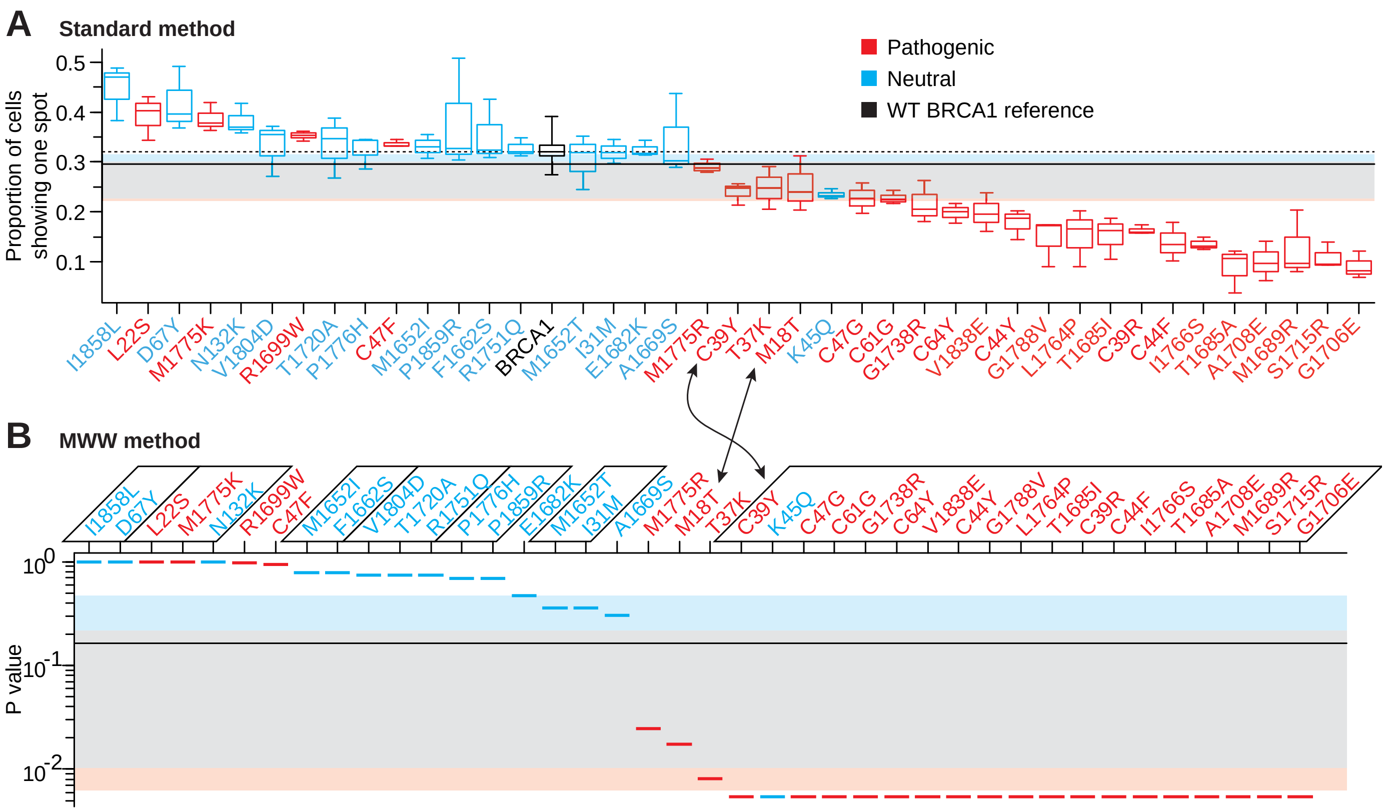

S9 Fig. Relative position of the variants in the Spot Formation assay and fluctuation of the best cut-off

(**A-B**) As in Fig 1, except that boxplots and p values resulted from 3 (mutants) or 12 (BRCA1) values. Arrows pinpoint the ranking of the M18T and C39Y mutations, which is improved using the MWW method, as explained in the main text introducing this method. The incoherent ranking observed with the standard method results from M18T that exhibits one value above the experimental best cut-off (shown by the top whisker overlaying the thick horizontal line) while C39Y has none.

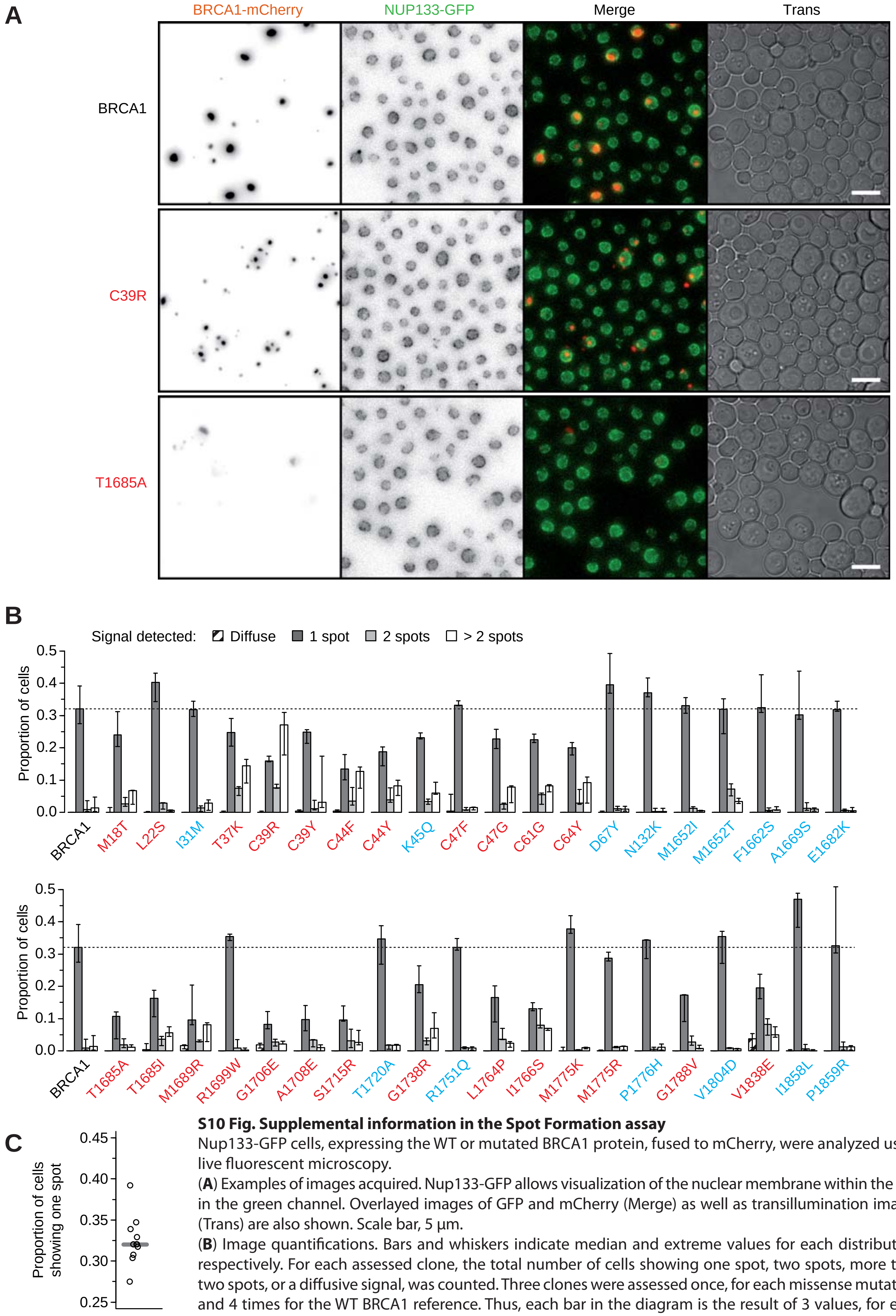

S10 Fig. Supplemental information in the Spot Formation assay

Nup133-GFP cells, expressing the WT or mutated BRCA1 protein, fused to mCherry, were analyzed using live fluorescent microscopy.

(**A**) Examples of images acquired. Nup133-GFP allows visualization of the nuclear membrane within the cell, in the green channel. Overlayed images of GFP and mCherry (Merge) as well as transillumination images (Trans) are also shown. Scale bar, 5 µm.

(**B**) Image quantifications. Bars and whiskers indicate median and extreme values for each distribution, respectively. For each assessed clone, the total number of cells showing one spot, two spots, more than two spots, or a diffusive signal, was counted. Three clones were assessed once, for each missense mutation, and 4 times for the WT BRCA1 reference. Thus, each bar in the diagram is the result of 3 values, for each missense mutation, and 12 values for the WT BRCA1 reference. In the Spot Formation assay, only the "1 spot" category is considered. The dotted horizontal line represents the median of BRCA1.

(**C**) Dotplot representation of the 12 BRCA1 values. The equivalent dotplot distribution of each mutant is shown in **B**, with the 3 values from each mutant represented by the top of the dark grey bar and the two whisker extremities, and also in S9A Fig, where the 3 values correspond to the median bar and the two whisker extremities.

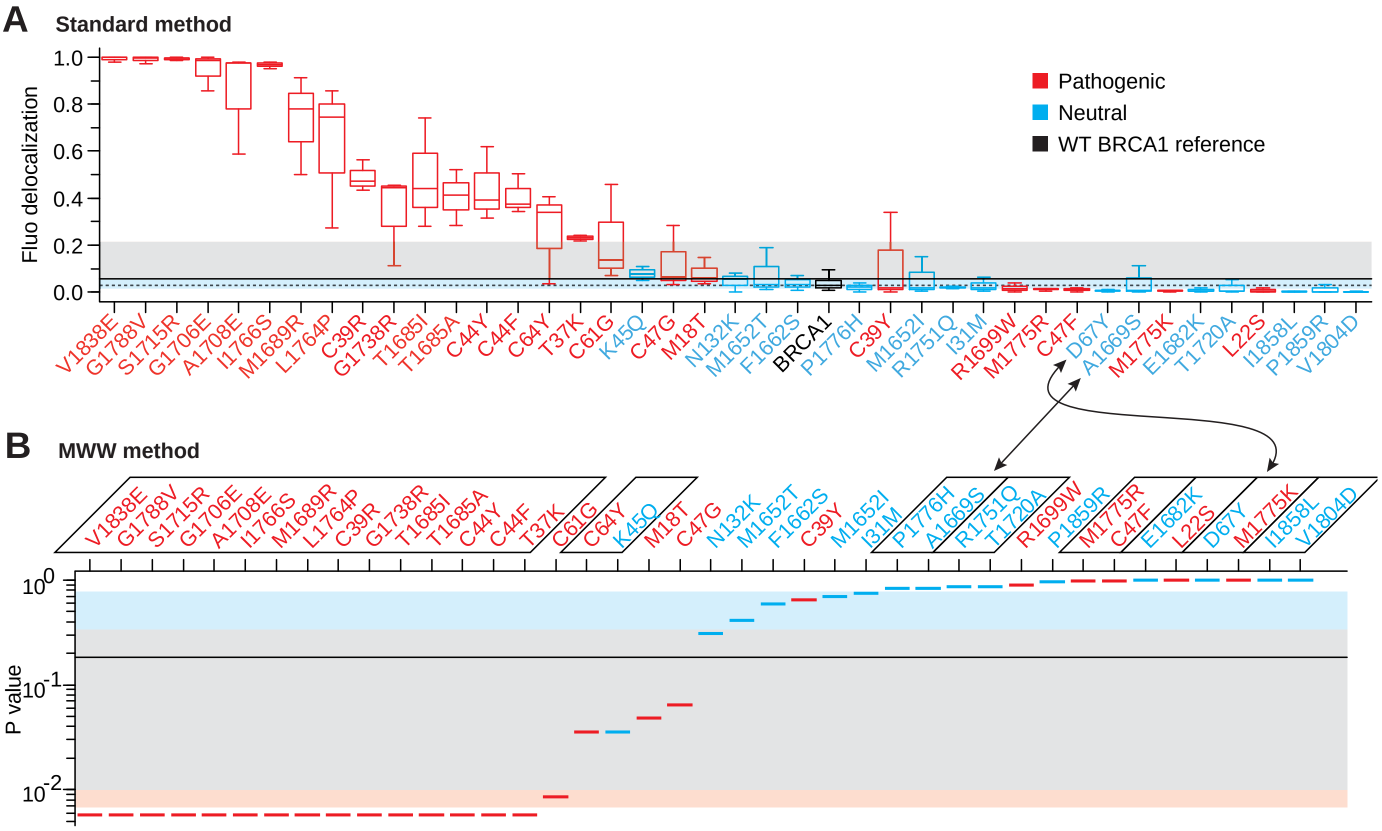

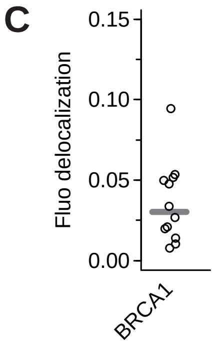

S11 Fig. Relative position of the variants in the Yeast Localization assay and fluctuation of the best cut-off

(**A-B**) As in Fig 1, except that the boxplots and p values are the results of 3 (mutants) or 12 (BRCA1) values. Delocalization of the mCherry fluorescent signal from the nucleus ranges from 0 (no cytoplasmic delocalization) to 1 (full cytoplasmic delocalization). Arrows pinpoint the ranking of the A1669S and D67Y mutations, which is improved using the MWW method, as explained in the main text introducing this method. The incoherent ranking observed with the standard method results from A1669S that exhibits one value above the experimental best cut-off while D67Y has none.

(**C**) Dotplot representation of the 12 BRCA1 values forming the BRCA1 boxplot in **A**. The equivalent dotplot distribution of each mutant is shown in **A**, with the 3 values from each mutant represented by the median bar and the two whisker extremities.

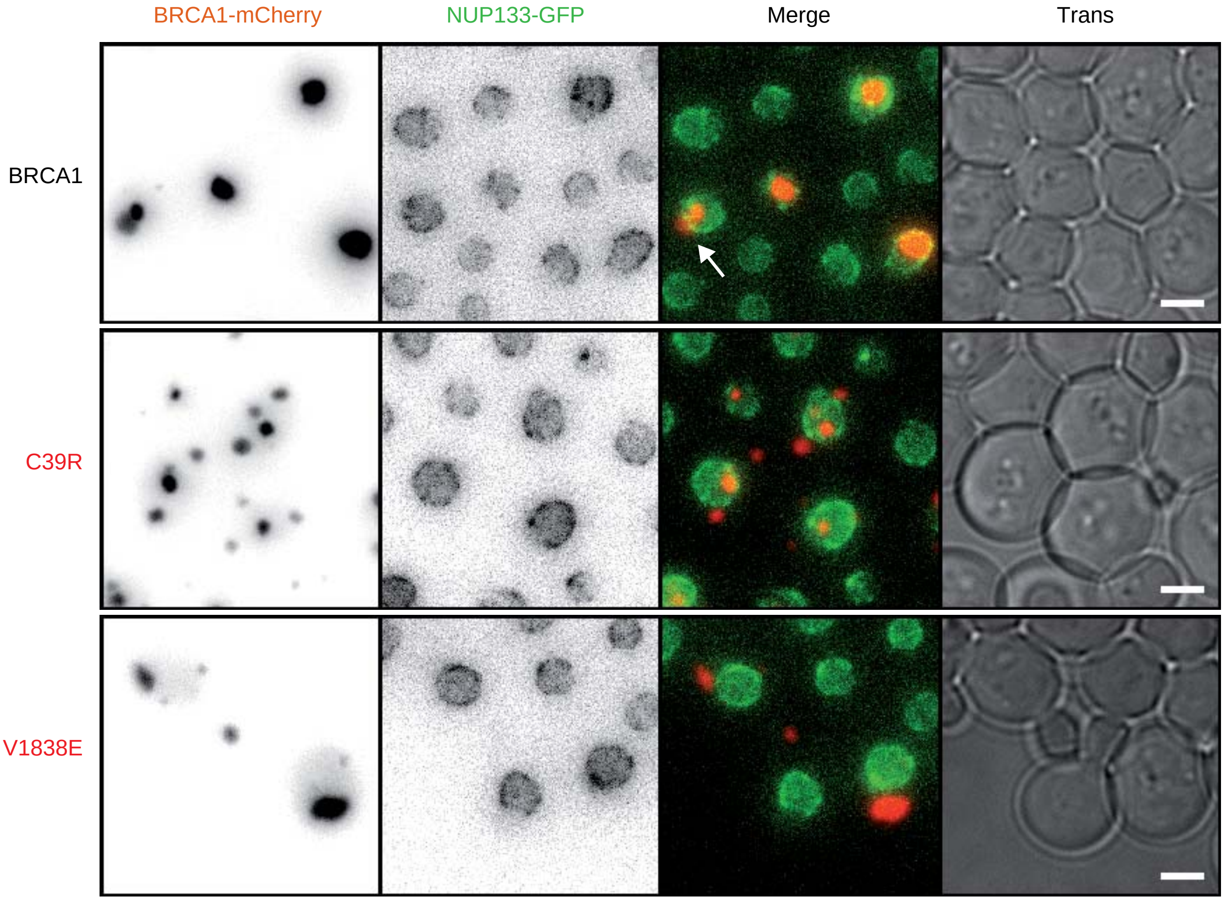

S12 Fig. Supplemental information in the Yeast Localization assay

Fluorescent images acquired in the Yeast Localization assay, as in S10A Fig. The arrow points to rare cytoplasmic spot in cells expressing the WT BRCA1-mCherry protein. Scale bar, 2 µm.

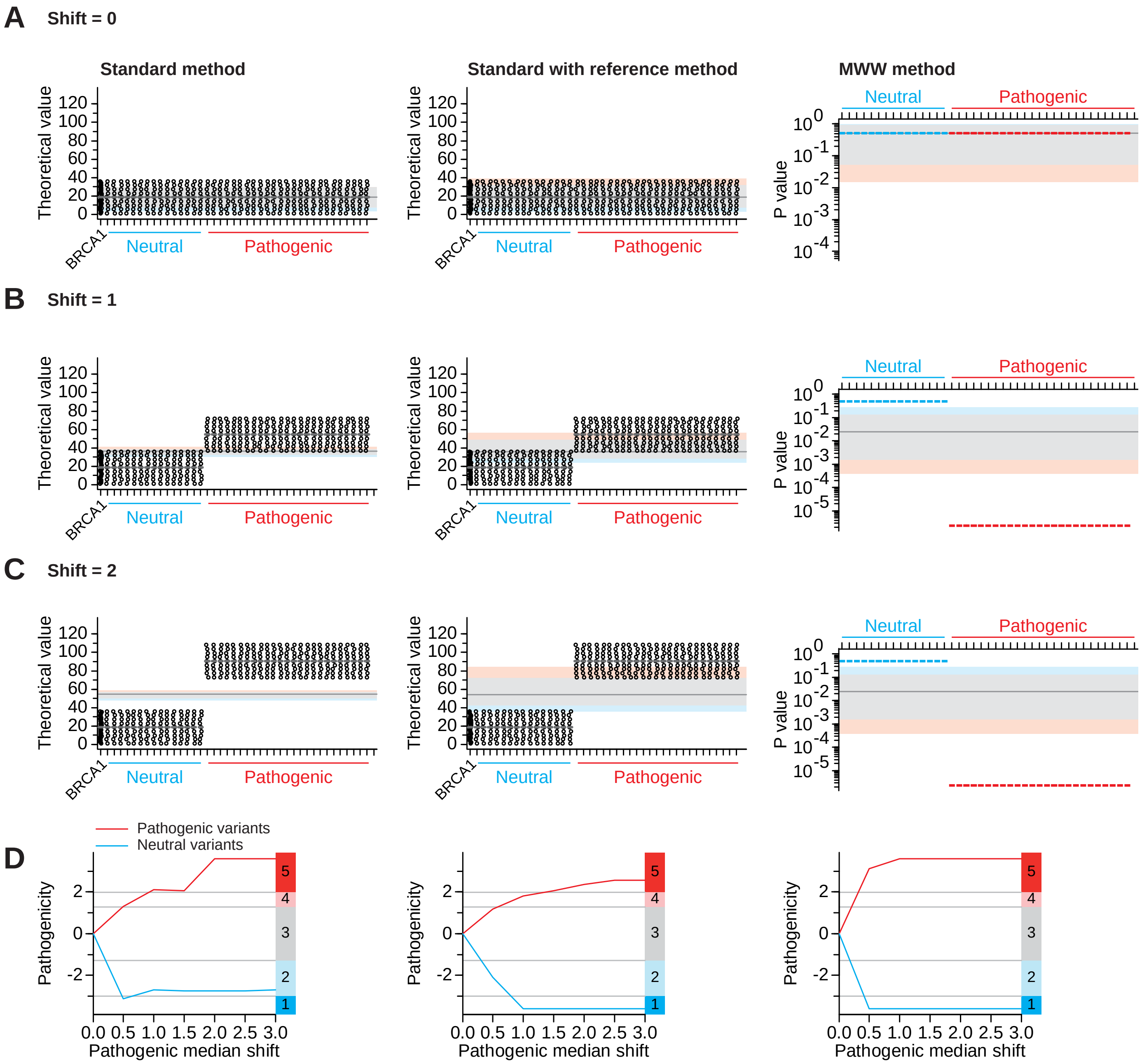

S13 Fig. Effect of the position of the pathogenic mutations on the probability system of classification (theoretical situation)
The parameters of the theoretical distributions used are detailed in Table S8. The reference situation is as follows: $n_{mutant} = 9$, $n_{BRCA1} = 36$, $n_{neutral} = 15$ and $n_{pathogenic} = 25$. In addition, medians and ranges of the neutral and WT BRCA1 distributions were made systematically equal. Distributions of the neutral and pathogenic mutations were identical, except for the shift of the pathogenic values from the neutral mutations, according to the formula $v_{ij} + 36 \times s$, with s representing the shift intensity and v_{ij} representing the value i of the pathogenic mutation j. When s = 0, pathogenic and neutral distributions are identical. Fluctuations from the best cut-off were obtained exactly as performed for the Colony Size, Liquid Medium, Spot Formation and Yeast Localization assays.
(**A-C**) Examples of shift intensities and best cut-off fluctuation results. The graphs depicted are similar to those in Fig 1, except that the standard, standard with reference and MWW methods are shown respectively on the left, middle and right of the figure. In the standard and standard with reference methods, boxplots are replaced by dotplots with the median of the distributions indicated by a grey segment. The s values are indicated (top left). In the subsequent supplemental figures, the position of the pathogenic mutation medians are as in **C** (s = 2). The grey horizontal line indicates the median of the best cut-off fluctuation.
(**D**) Probabilities of pathogenicity obtained for the neutral (blue line) and pathogenic variants (red line), depending on the shift intensity of the pathogenic mutations. Y-axis, log10(p / (1 - p)) with p being the probability of pathogenicity of the variants (0 corresponds to p = 0.5); right colored classes, five-class nomenclature with the horizontal grey lines showing the 0.99, 0.95, 0.05 and 0.001 limits of the classes (see S1 Table). In the standard method, the slight erratic curves and the lack of specificity sometimes observed (blue line in the class 2 instead of class 1) is due to the fact that this method generates a low number of different best cut-off values (between 8 and 64) in the best cut-off distributions, as explained in the S1 Text.
As summarized in S9 Table, these results confirm that the probability system of classification is an efficient variant classifier. Indeed, whatever method is used, when the pathogenic and neutral distributions are strictly identical, they all locate inside the class 3 area (i.e., the system cannot classify any variants in such kind of functional assay). Moreover, the probability system of classification is improved when the pathogenic mutations shift from the neutral sector towards the pathogenic sector, since the probability of pathogenicity increases for the pathogenic variants and decreases for the neutral ones.

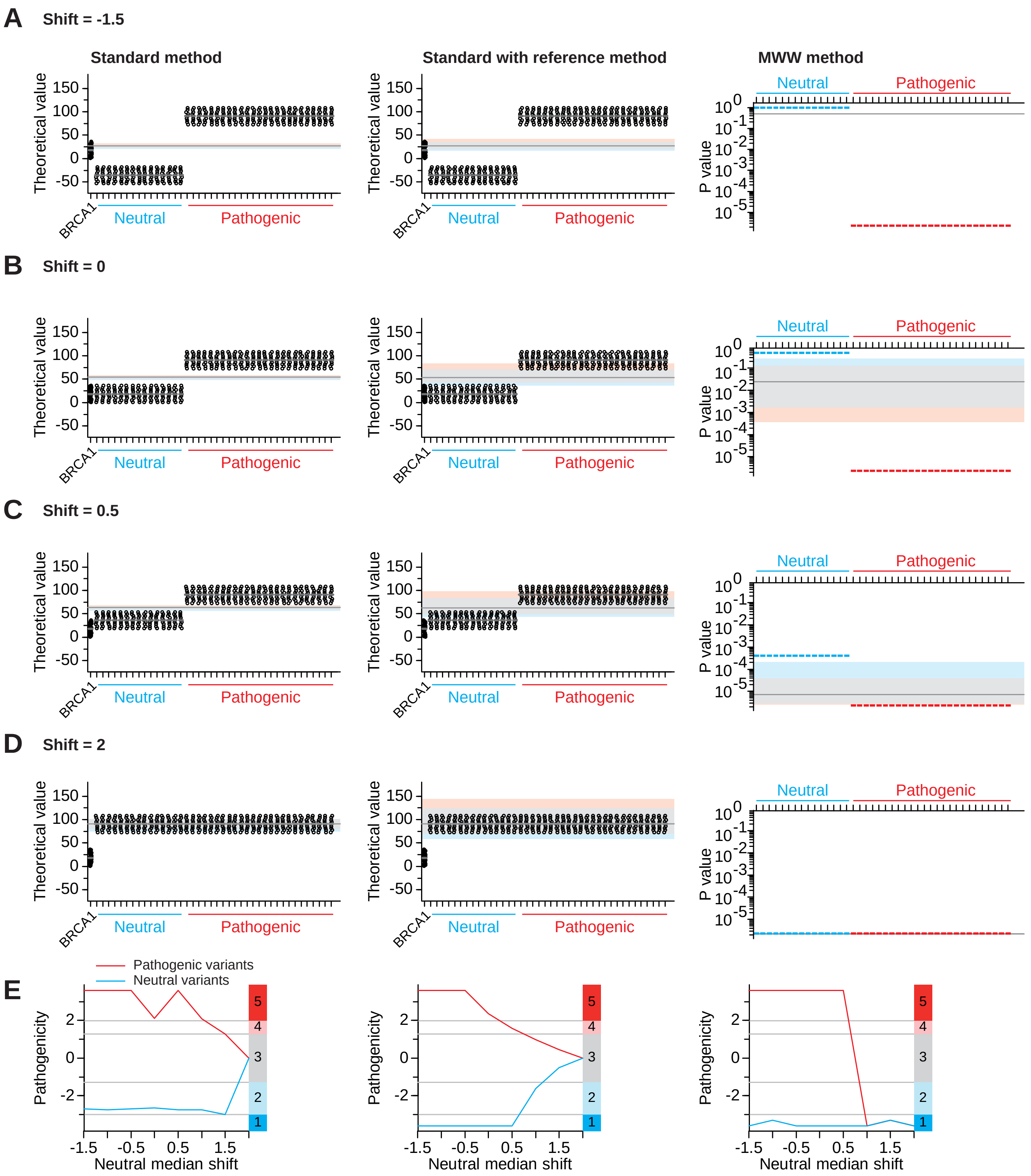

S14 Fig. Effect of the position of the neutral mutations on the probability system of classification (theoretical situation)

See S13 Fig for details. Neutral mutations were shifted according to the formula $v_{ij} + 36 \times s$, with s representing the shift intensity and v_{ij} representing the value i of the neutral mutation j (when s = 0, medians and extreme values of the BRCA1 and neutral distributions are identical. When s = 2, pathogenic and neutral distributions are identical).

(**A-D**) Examples of shift intensities and best cut-off fluctuation results. The s values are indicated (top left).

(**E**) Probabilities of pathogenicity obtained for the neutral (blue line) and pathogenic variants (red line), depending on the shift intensity of the neutral mutations.

As summarized in S9 Table, these results highlight divergences between the different methods. With the standard method and the standard with reference methods (**E**, left and middle panels), sensitivity and specificity of the probability system of classification decrease when the neutral mutations approach the pathogenic mutations. With the MWW method (**E**, right panel), the probability system of classification results in a complete misclassification of the pathogenic mutations when the neutral distributions do not overlap the WT reference distribution ($s \geq 1$). Of note, these analyses treat extreme situations. In practice, the WT reference should be well embedded within the neutral distributions. The opposite situation would raise question about the WT reference or neutral mutations used.

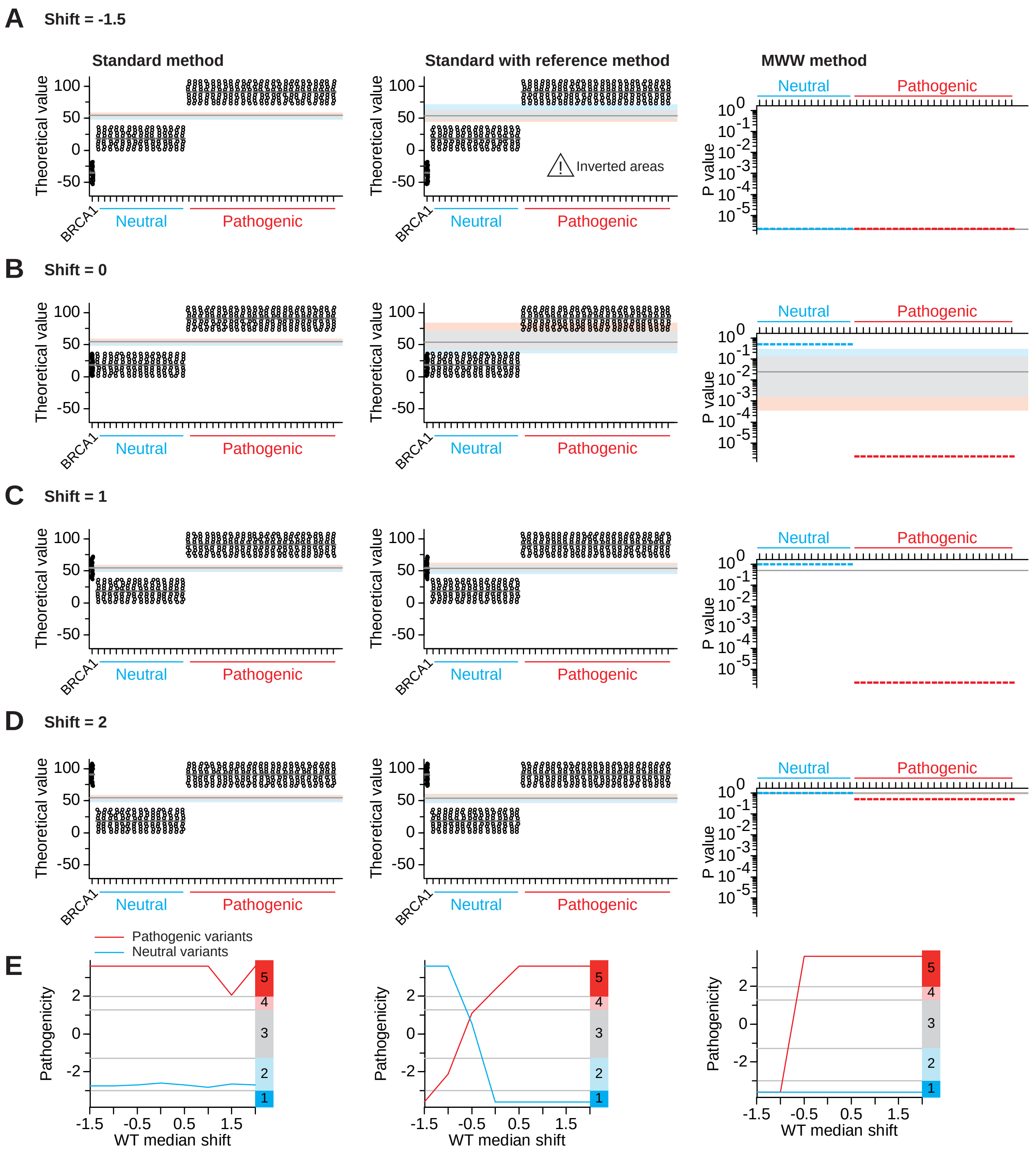

S15 Fig. Effect of the position of the WT BRCA1 reference on the probability system of classification (theoretical situation)
See S13 Fig for details. Values of the WT BRCA1 distribution were shifted according to the formula $v_i + 36 \times s$, with s representing the shift intensity and v_i representing the value i of the BRCA1 reference (when s = 0, medians and extreme values of the neutral and BRCA1 distributions are identical. When s = 2, medians and extreme values of the pathogenic and BRCA1 distributions are identical). Of note, these theoretical analyses treat extreme situations. In practice, the WT reference should be well embedded in the neutral distributions. The opposite situation would raise question about the WT reference or neutral mutations used.
(**A-D**) Examples of shift intensities and best cut-off fluctuation results. The s values are indicated (top left).
(**E**) Probabilities of pathogenicity obtained for the neutral (blue line) and pathogenic variants (red line), depending on the shift intensity of the WT reference.
As summarized in S9 Table, these results highlight divergences between the different methods. As expected, the standard method is not affected by the position of the WT BRCA1 distribution (**E**, left panel). In contrast, the standard with reference method is strongly influenced by the position of this reference (**E**, middle panel). When the WT BRCA1 median shifts towards the null value, sensitivity and specificity of the probability system of classification are decreased, with a complete loss of sensitivity and specificity (i.e., systematic classification as class 3) when the WT BRCA1 median is null ($s \approx -0.514$). This was expected since the standard with reference method is based on best cut-off values divided by the WT BRCA1 median. Thus, a division by zero generates relative best cut-offs with an infinite value. Such issues are compensated only when best cut-offs are close to the WT BRCA1 median. This was shown in the Liquid Medium and Yeast Localization assays. Using the standard or standard with reference method provided similar variant classification (Fig 2B), even if the WT BRCA1 medians of these assays approached zero, with 0.144 and 0.03 respectively (S4 Table). In conclusion, a situation, in which the WT reference median is close to zero, with the fluctuation of the raw best cut-off far from this median, will guarantee a weak sensitivity and specificity of the probability system of classification. Concerning the standard with reference method, it is also noteworthy that a negative value of the WT reference median ($s < -0.514$) inverts the classification (**E**, middle panel), as expected, regardless of the values from the neutral and pathogenic mutations. When comparing the standard with reference method versus the MWW method, the later has the advantage of being independent of the WT reference values, as only overlapping distributions matter. Specificity of the probability system of classification is not affected by the position of the WT reference, contrary to sensitivity (**E**, right panel). The main weakness of the MWW method occurs when the WT reference distribution falls outside of the range of the neutral and pathogenic distributions (as in **A**, left panel), which generates misclassification of the pathogenic mutations as neutral.

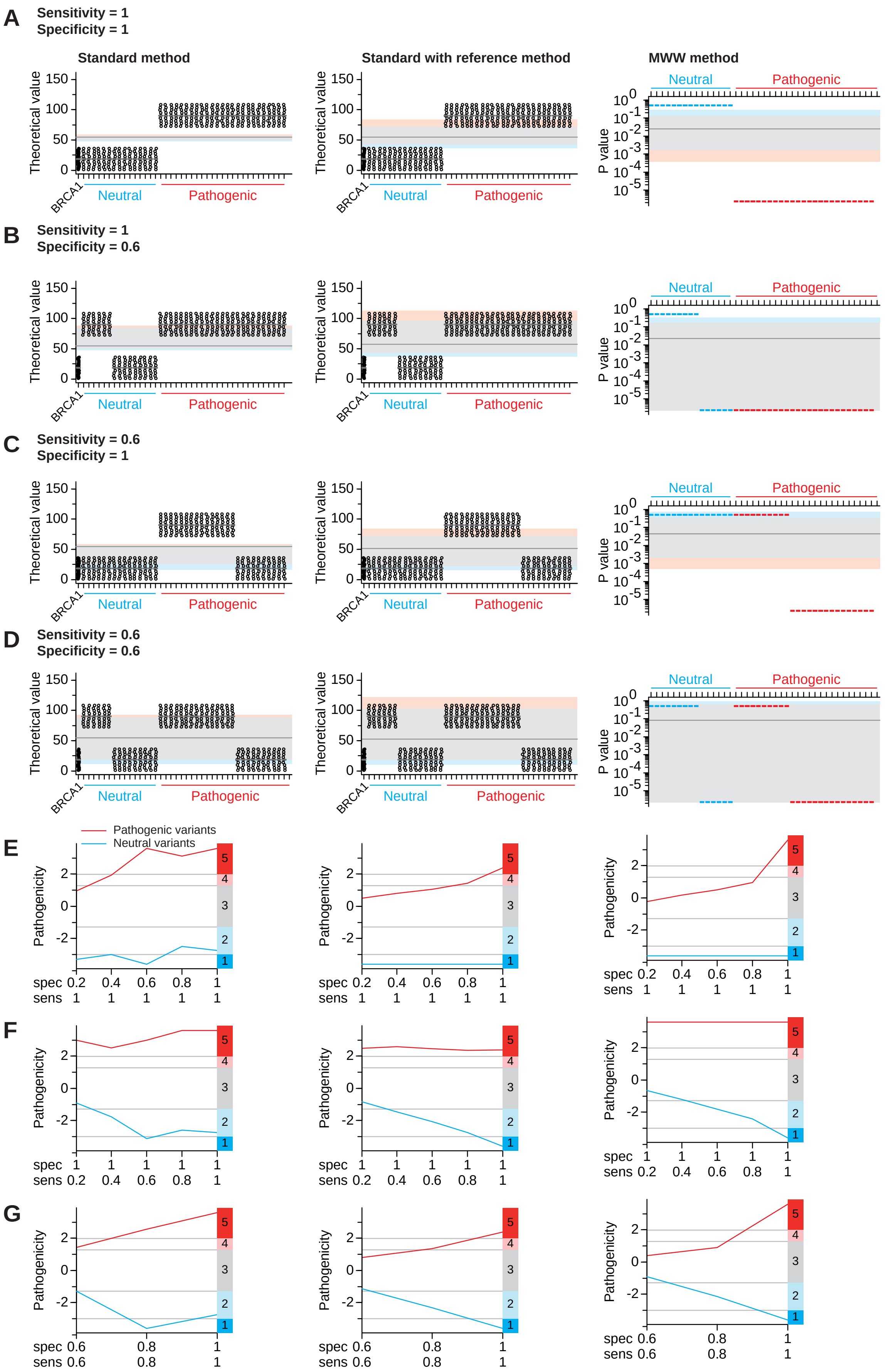

S16 Fig. Effect of the experimental sensitivity and specificity on the probability system of classification (theoretical situation)
See S13 Fig for details. The experimental sensitivity and specificity were modulated by assigning certain pathogenic mutants in the neutral region and certain neutral mutants in the pathogenic region, respectively. The experimental sensitivity and specificity values indicated were those obtained with the experimental best cut-off, as explained in S2 Fig. These values are referred to as "initial" sensitivity and specificity, as opposed to the sensitivity and specificity of the probability system of classification, obtained after bootstrap analysis.
(**A-D**) Examples of experimental sensitivities/specificities and best cut-off fluctuation results.
(**E-G**) Probabilities of pathogenicity obtained for the neutral (blue line) and pathogenic variants (red line), depending on decreases from experimental specificity (**E**), experimental sensitivity (**F**) or both (**G**).
As summarized in S9 Table, these results confirm that the probability system of classification is an efficient variant classifier. A decrease of the experimental specificity indicates that the pathogenic area is contaminated by neutral variants, which reduces the probability of pathogenicity of the pathogenic variants (class 5 towards class 3). In the same manner, a decrease of the experimental sensitivity indicates that the neutral area is contaminated by pathogenic variants, which enhances the probability of pathogenicity of the neutral variants (class 1 towards class 3). This was observed using the three standard, standard with reference and MWW methods. Of note, the situations studied used systematically: experimental sensitivity + experimental specificity ≥ 1 (otherwise representing an inappropriate use of the experimental information, i.e., pathogenic and neutral sectors incorrectly positioned).

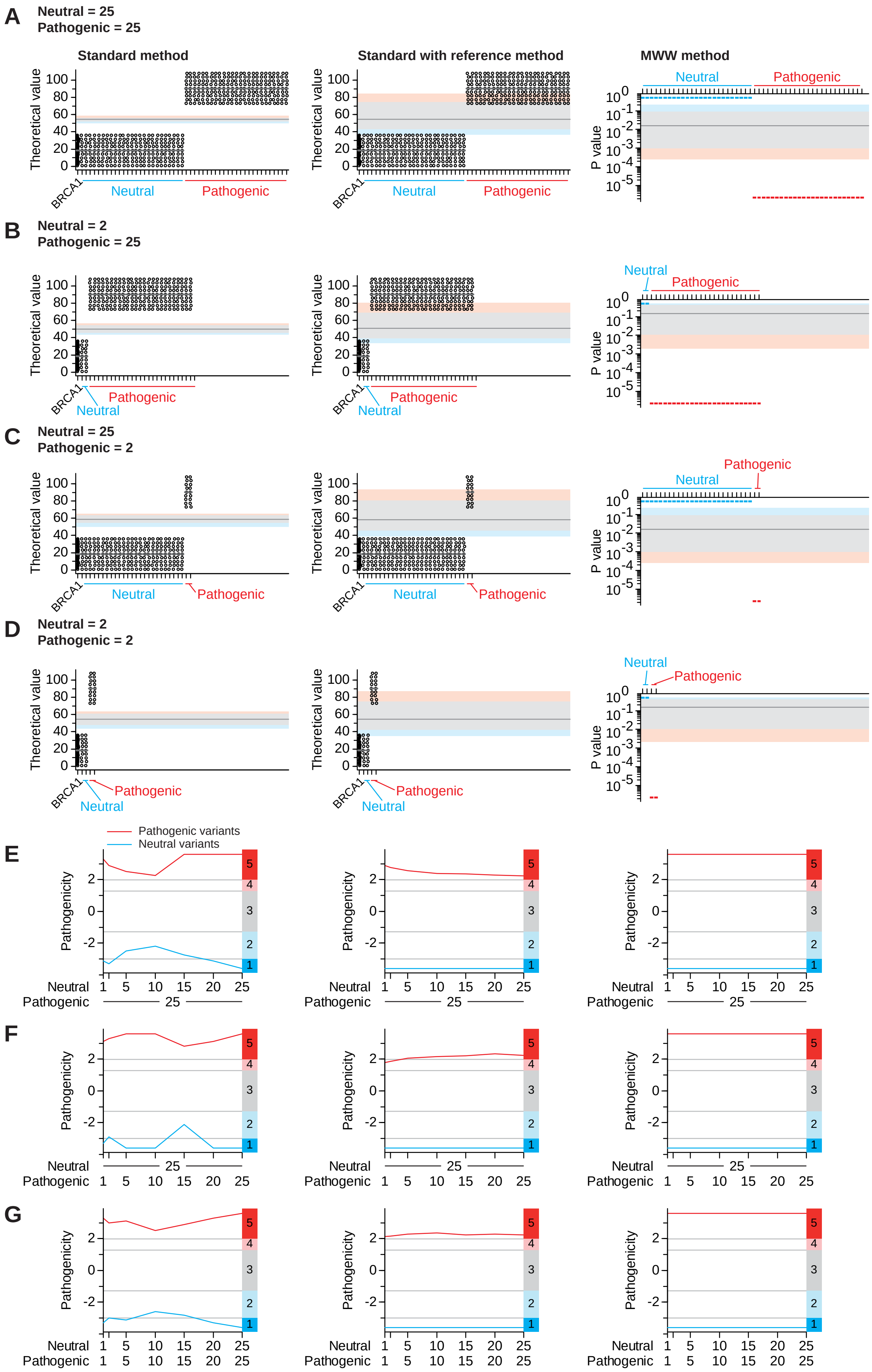

S17 Fig. Effect of the number of neutral and pathogenic mutations on the probability system of classification (theoretical situation)

See S13 Fig for details.

(**A-D**) Examples showing the number of neutral and pathogenic mutations tested, with best cut-off fluctuation results.

(**E-G**) Probabilities of pathogenicity obtained for the neutral (blue line) and pathogenic variants (red line), following a decrease in the number of neutral mutations (**E**), pathogenic mutations (**F**) or both (**G**).

As summarized in S9 Table, these results show that the probability system is poorly sensitive to the number of neutral and pathogenic mutations incorporated, whatever method is used.

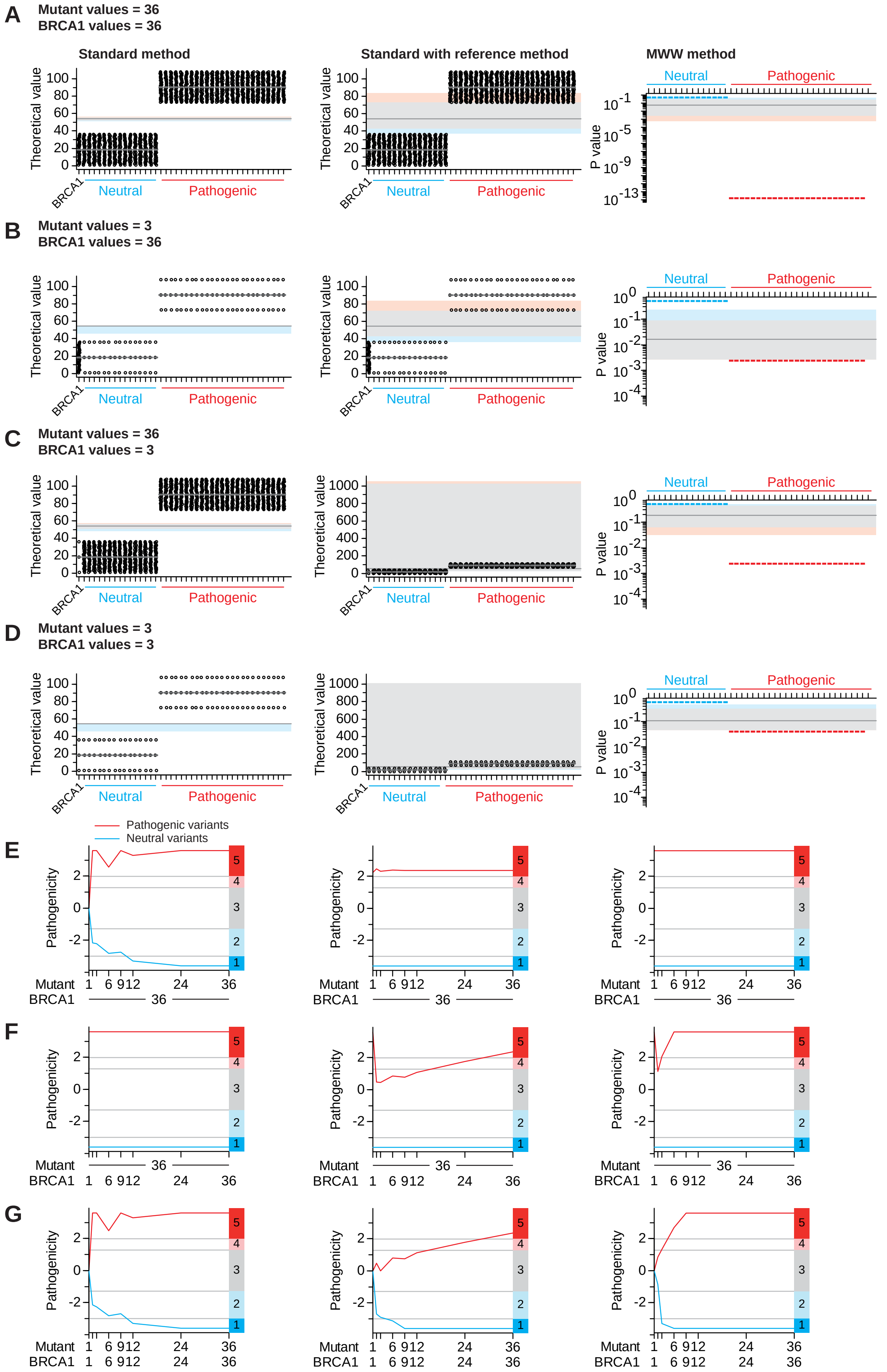

S18 Fig. Effect of the number of mutant and BRCA1 values on the probability system of classification (theoretical situation)

See S13 Fig for details. Number of values was modulated so that the range and median of the distributions remained the same, as shown in S8 Table.

(**A-D**) Examples showing the number of mutant or BRCA1 values tested, with best cut-off fluctuation results.

(**E-G**) Probabilities of pathogenicity obtained for the neutral (blue line) and pathogenic variants (red line), following a decrease in the number of mutant values (**E**), BRCA1 values (**F**), or both (**G**).

As summarized in S9 Table, these results confirm that the probability system of classification is an efficient variant classifier. Whatever method is used, a decreasing number of values in the dataset affects the probabilities of both the pathogenic and neutral variants (**G**), which tend toward 0.5 (class 3). Thus, the probability system prevents decision-making when data is lacking. As expected, the standard method is not affected by the number of BRCA1 values (**F**, left panel). The standard with reference and the MWW methods are insensitive to the number of mutant values if the number of BRCA1 values is high (**E**, middle and right panels). However, a decrease in the number of BRCA1 values lowers the probability of pathogenicity of the pathogenic variants (**F**, middle and right panels), but with a strong recovery when the fluctuation of the best cut-off is no longer influenced by the fluctuation of the WT reference (n_{BRCA1} = 1). Of note, the best cut-off does not fluctuate when n_{mutant} = 1 and n_{BRCA1} = 1 (**G**), which results in a probability of pathogenicity equal to 0.5 for both the pathogenic and neutral variants. Moreover, using the standard method, when n_{mutant} = 9, the classification of the neutral mutations is class 2 (**E**, left panel), which explains the lack of specificity frequently observed in S13-S19 Figs.

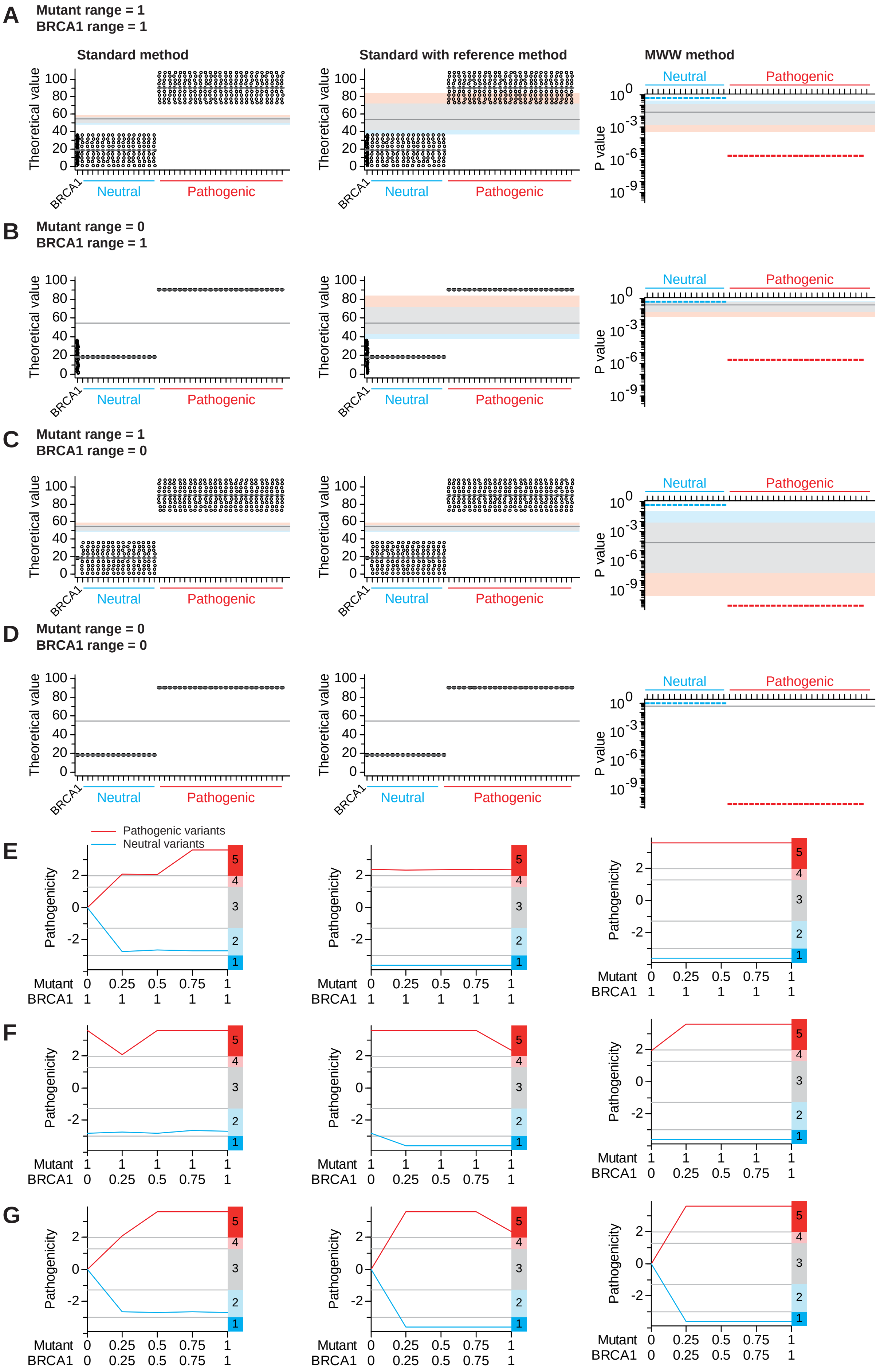

S19 Fig. Effect of the range of mutant and BRCA1 distributions on the probability system of classification (theoretical situation)

See S13 Fig for details. Distribution ranges were modulated so that medians remained the same, as shown in S8 Table. The range factor r, indicated on the graphs, illustrates the relative dispersion of the distributions. When r = 0, the dispersion is null.

(**A-D**) Examples showing the ranges of the mutant and BRCA1 distributions tested, with best cut-off fluctuation results.

(**E-G**) Probabilities of pathogenicity obtained for the neutral (blue line) and pathogenic variants (red line), following a range decrease of the mutant distributions (**E**), BRCA1 distribution (**F**), or both (**G**).

As summarized in S9 Table, these results indicate that the probability system of classification is affected mainly when the range of the BRCA1 and mutant distributions is null, whatever method is used. In this situation, the fluctuation of the best cut-off is null and all the mutations are considered as absolutely unknown (probability of pathogenicity equal to 0.5).

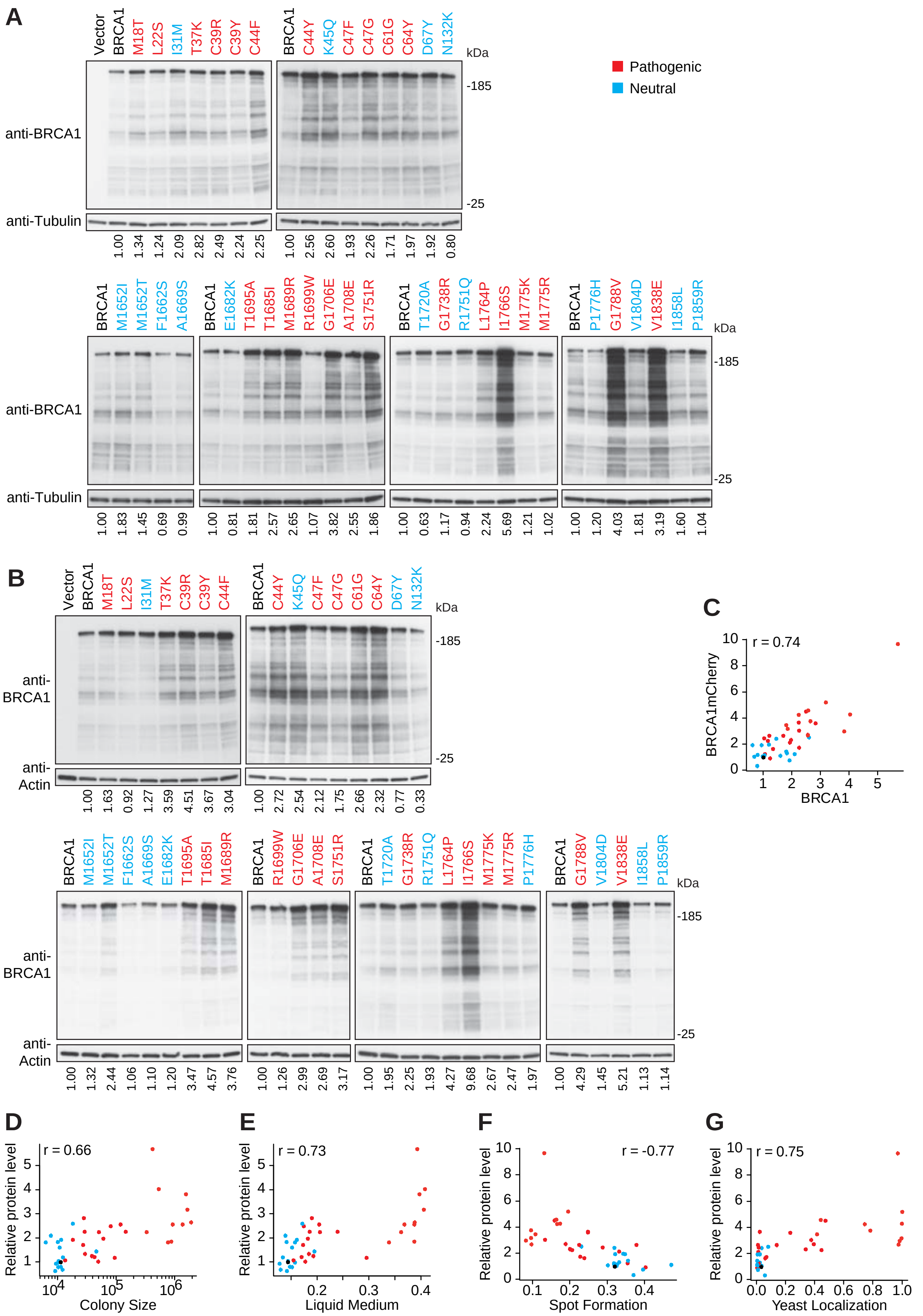

S20 Fig. Western blot analysis

After 4 hours of BRCA1 expression, lysates of 6 x 10^6 cells were examined for the presence of the protein (theoretical size: 200 kDa) with an anti-BRCA1 antibody. Tubulin or Actin was used as a loading control and was probed using an anti-Tubulin or anti-Actin antibody on the same membrane after stripping the first labeling. Signal intensities of full lanes, relatively to the BRCA1 lane, are indicated below. Of note, protein levels three times higher than the WT BRCA1 protein level (normalized to 1) systematically correspond to pathogenic mutations.

(**A**) BRCA1 (Colony Size and Liquid Medium assays).

(**B**) BRCA1-mCherry (Spot Formation and Yeast Localization assays).

(**C-G**) Dotplot with the Spearman coefficient of correlation indicated. Pathogenic and neutral mutations, as well as the WT BRCA1 reference, are represented by a red, blue or black dot, respectively.

(**C**) Correlation between the relative signal intensities of **A** and **B**.

(**D-E**) Correlation between the relative signal intensities of **A** and medians of the Colony Size or Liquid Medium assay.

(**F-G**), correlation between the relative signal intensities of **B** and medians of the Spot Formation or Yeast Localization assay.

A

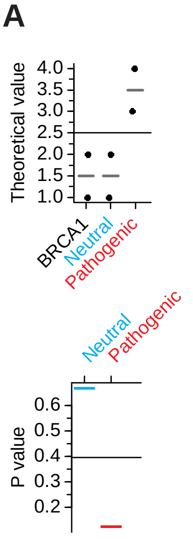

B

Sampling possibility n°	WT BRCA1 reference		Neutral variant				Pathogenic variant				Best cut-off value		
	Value 1	Value 2	Value 1	Value 2	Median	P value	Value 1	Value 2	Median	P value	Standard method	Standard with reference method	MWW method
1	1	1	1	1	1.0	1.000	3	3	3.0	0.097	2.00	2.000	0.54848
2	1	1	1	1	1.0	1.000	3	4	3.5	0.110	2.25	2.250	0.55517
3	1	1	1	1	1.0	1.000	4	4	4.0	0.097	2.50	2.500	0.54848
4	1	1	1	2	1.5	0.309	3	3	3.0	0.097	2.25	2.250	0.20275
5	1	1	1	2	1.5	0.309	3	4	3.5	0.110	2.50	2.500	0.20944
6	1	1	1	2	1.5	0.309	4	4	4.0	0.097	2.75	2.750	0.20275
7	1	1	2	2	2.0	0.097	3	3	3.0	0.097	2.50	2.500	0.09697
8	1	1	2	2	2.0	0.097	3	4	3.5	0.110	2.75	2.750	0.10365
9	1	1	2	2	2.0	0.097	4	4	4.0	0.097	3.00	3.000	0.09697
10	1	2	1	1	1.0	0.933	3	3	3.0	0.110	2.00	1.333	0.52176
11	1	2	1	1	1.0	0.933	3	4	3.5	0.123	2.25	1.500	0.52792
12	1	2	1	1	1.0	0.933	4	4	4.0	0.110	2.50	1.667	0.52176
13	1	2	1	2	1.5	0.667	3	3	3.0	0.110	2.25	1.500	0.38892
14	1	2	1	2	1.5	0.667	3	4	3.5	0.123	2.50	1.667	0.39507
15	1	2	1	2	1.5	0.667	4	4	4.0	0.110	2.75	1.833	0.38892
16	1	2	2	2	2.0	0.309	3	3	3.0	0.110	2.50	1.667	0.20944
17	1	2	2	2	2.0	0.309	3	4	3.5	0.123	2.75	1.833	0.21559
18	1	2	2	2	2.0	0.309	4	4	4.0	0.110	3.00	2.000	0.20944
19	2	2	1	1	1.0	0.985	3	3	3.0	0.097	2.00	1.000	0.54089
20	2	2	1	1	1.0	0.985	3	4	3.5	0.110	2.25	1.125	0.54757
21	2	2	1	1	1.0	0.985	4	4	4.0	0.097	2.50	1.250	0.54089
22	2	2	1	2	1.5	0.933	3	3	3.0	0.097	2.25	1.125	0.51508
23	2	2	1	2	1.5	0.933	3	4	3.5	0.110	2.50	1.250	0.52176
24	2	2	1	2	1.5	0.933	4	4	4.0	0.097	2.75	1.375	0.51508
25	2	2	2	2	2.0	1.000	3	3	3.0	0.097	2.50	1.250	0.54848
26	2	2	2	2	2.0	1.000	3	4	3.5	0.110	2.75	1.375	0.55517
27	2	2	2	2	2.0	1.000	4	4	4.0	0.097	3.00	1.500	0.54848

C Probability system & standard method

Different best cut-off value	2	2.25	2.5	2.75	3
Repeat	3	6	9	6	3
Probability	0.11	0.22	0.33	0.22	0.11
CDF1	0.11	0.33	0.67	0.89	1.00
CDF2	0.00	0.11	0.33	0.67	0.89
Average CDF	0.06	0.22	0.50	0.78	0.94

1.5 3.5

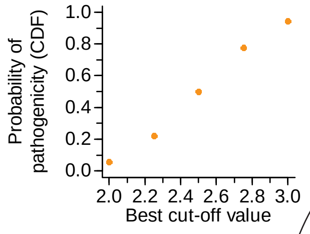

D Probability system & standard with reference method

Different best cut-off value	1.000	1.125	1.250	1.333	1.375	1.500	1.667	1.833	2.000	2.250	2.500	2.750	3.000
Repeat	1	2	3	1	2	3	3	2	2	2	3	2	1
Probability	0.04	0.07	0.11	0.04	0.07	0.11	0.11	0.07	0.07	0.07	0.11	0.07	0.04
CDF1	0.04	0.11	0.22	0.26	0.33	0.44	0.56	0.63	0.70	0.78	0.89	0.96	1.00
CDF2	0.00	0.04	0.11	0.22	0.26	0.33	0.44	0.56	0.63	0.70	0.78	0.89	0.96
Average CDF	0.02	0.07	0.17	0.24	0.30	0.39	0.50	0.59	0.67	0.74	0.83	0.93	0.98

1 2.33

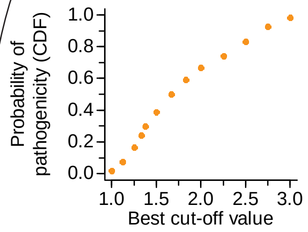

E Probability system & MWW method

Different best cut-off value	0.5552	0.5485	0.5476	0.5409	0.5279	0.5218	0.5151	0.3951	0.3889	0.2156	0.2094	0.2028	0.1037	0.0970
Repeat	2	4	1	2	1	3	2	1	2	1	3	2	1	2
Probability	0.07	0.15	0.04	0.07	0.04	0.11	0.07	0.04	0.07	0.04	0.11	0.07	0.04	0.07
CDF1	0.07	0.22	0.26	0.33	0.37	0.48	0.56	0.59	0.67	0.70	0.81	0.89	0.93	1.00
CDF2	0.00	0.07	0.22	0.26	0.33	0.37	0.48	0.56	0.59	0.67	0.70	0.81	0.89	0.93
Average CDF	0.04	0.15	0.24	0.30	0.35	0.43	0.52	0.57	0.63	0.69	0.76	0.85	0.91	0.96

0.667 0.123

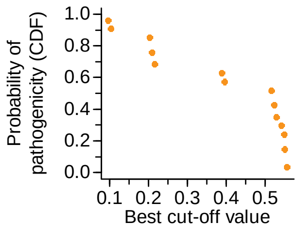

F Quantile system

	Standard method				Standard with reference method				MWW method			
Quantile	0.99	0.95	0.05	0.001	0.99	0.95	0.05	0.001	0.999	0.95	0.05	0.01
Best cut-off value	3	3	2	2	2.935	2.750	1.125	1.003	0.555	0.553	0.099	0.097

3.5 1.5 2.33 1 0.667 0.123

S21 Fig. Exact probability distribution of the best cut-off in the standard, standard with reference and MWW methods (theoretical situation)

The theoretical situation was analyzed as follows: one neutral and one pathogenic mutation ($n_{neutral} = 1$ and $n_{pathogenic} = 1$), with two values per mutant ($n_{mutant} = 2$, value 1 and 2 for the neutral mutant, and value 3 and 4 for the pathogenic mutant) and two values in the WT BRCA1 reference ($n_{BRCA1} = 2$, value 1 and 2).

(**A**) The graphs depicted are similar to those in Fig 1, except that boxplots are replaced by dotplots with median of the distributions indicated by a grey segment. The black horizontal line represents the experimental best cut-off. The best cut fluctuations (colored areas) are not represented but quantiles are shown in **F**.

(**B**) Table recapitulating all of the possible results when sampling 2 values, with replacement, among the 2 neutral, 2 pathogenic and 2 WT BRCA1 values. Each row is a different combination that provides a best cut-off value, for each method used. The framed row highlights the combination identical to the experimental situation in **A**. In this simple situation (1 neutral and 1 pathogenic variant), the best cut off computed, in each row, is the median of the two variant medians (standard method), the median of the two variant medians divided by the WT BRCA1 median (standard with reference method) and the median of the two variant p values (MWW method).

(**C-E**) Variant classification using the probability system, with the standard (**C**), standard with reference (**D**) and MWW (**E**) methods, as in S6B Fig. Colored numbers in the table correspond to the different probabilities of pathogenicity designed by the model. The color code respects the five-class nomenclature depicted in S1 Table: grey, class 3; light blue, class 2; pink, class 4. Positions of the neutral and pathogenic variants are represented by a blue and red arrow, respectively. The number below each arrow designates the variant value used in the probability system to attribute the probability of pathogenicity, which corresponds to the median, median divided by the WT reference median or p value, indicated in the framed row of **B**. For instance, in the standard method (**C**), the pathogenic variant, with a median of 3.5, has the probability 0.94 (class 3).

(**F**) Variant classification using the quantile system. Quantiles were computed from the 27 best cut-off values from **B**, for each method. The colored background defines the intervals within the best cut-off distribution, as explained in S23 Fig. Arrows depict the position of the neutral and pathogenic variants, as in **C-E**.

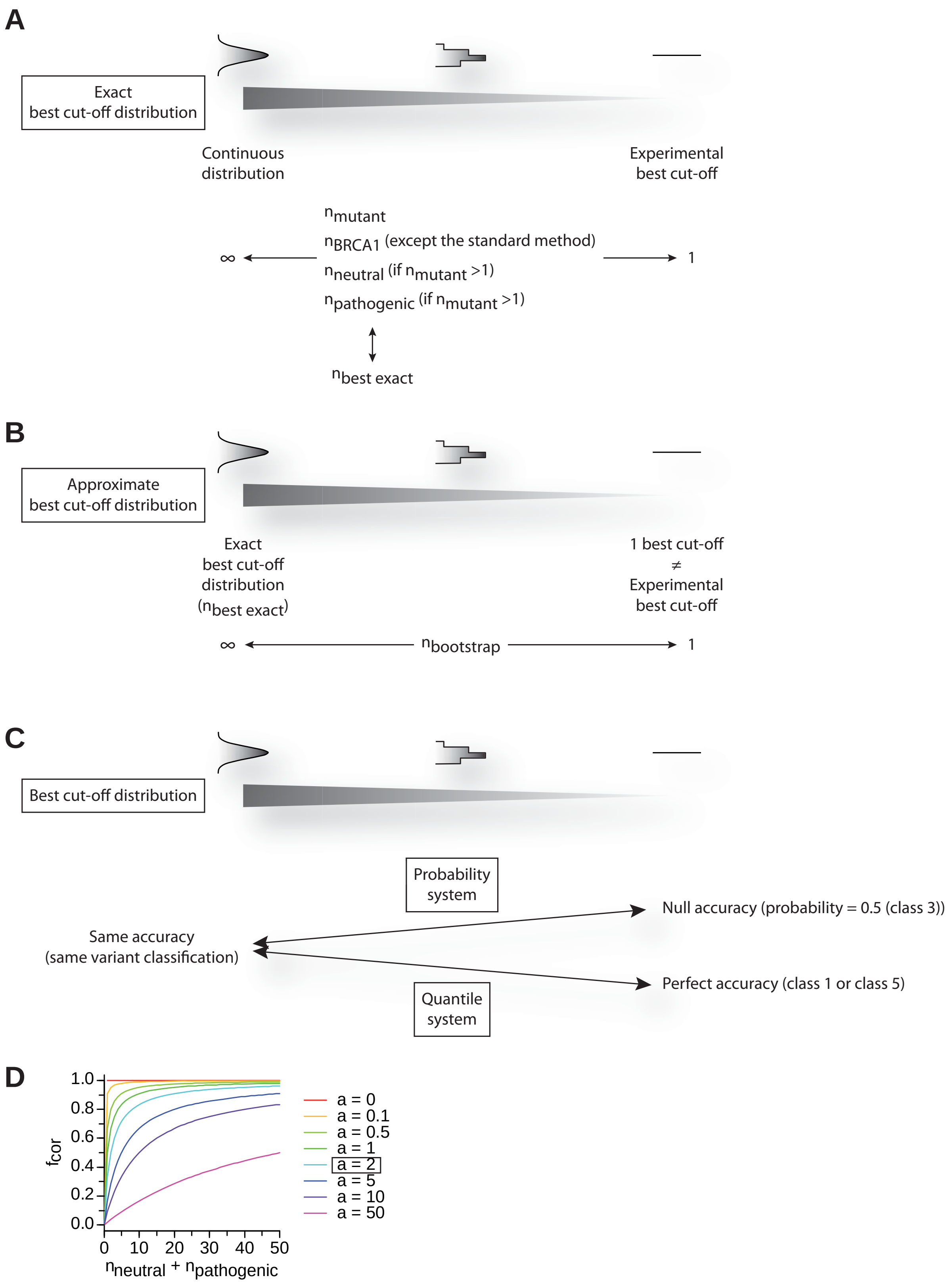

S22 Fig. Additional information about the classification model

(**A**) Schematic of the exact best cut-off distribution influenced by different parameters, assuming no ties. The number of neutral ($n_{neutral}$) and pathogenic ($n_{pathogenic}$) variants influence the exact best cut-off distribution only if the number of values per mutant (n_{mutant}) is above 1. The number of values in the WT reference (n_{BRCA1}) does not influence the exact best cut-off distribution in the standard method, only in the standard with reference and MWW methods.
(**B**) Schematic of the approximate best cut-off distribution influenced by the number of bootstraps performed. Importantly, a single bootstrap ($n_{bootstrap} = 1$) does not lead to the experimental best cut-off, except if $n_{best\ exact} = 1$.
(**C**) Accuracy of the probability and quantile systems of classification. The schematic illustration is valid, using either the exact or approximate best cut-off distribution.
(**D**) Correcting factor fcor used in the probability system of classification, depending on the parameter a (see the S1 Text). $f_{cor} = (n_{neutral} + n_{pathogenic}) / (n_{neutral} + n_{pathogenic} + a)$. The framed value (a = 2) was the value used in S13-S15 Tables.

A

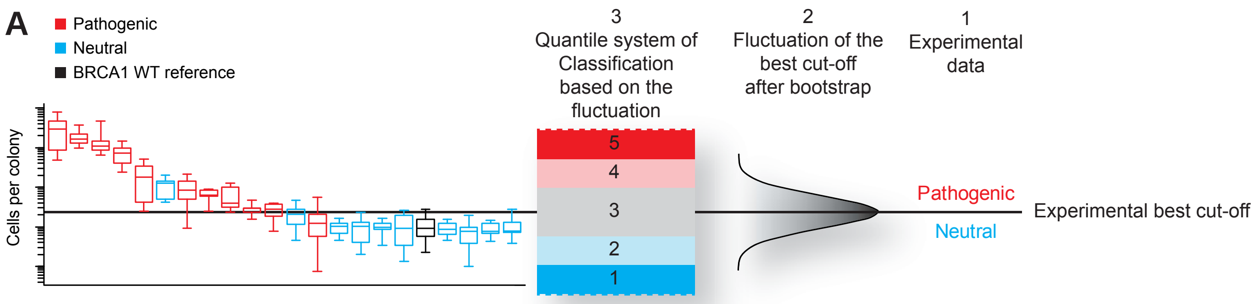

B

Class		Quantile in the 2 standard methods				Quantile in the MWW method			
		CS	LM	SF	YL	CS	LM	SF	YL
Definitely pathogenic	5	Q0.990	Q0.990	Q0.010	Q0.990	Q0.010	Q0.010	Q0.010	Q0.010
Likely pathogenic	4	Q0.950	Q0.950	Q0.050	Q0.950	Q0.050	Q0.050	Q0.050	Q0.050
Uncertain	3	Q0.050	Q0.050	Q0.950	Q0.050	Q0.950	Q0.950	Q0.950	Q0.950
Likely neutral	2	Q0.001	Q0.001	Q0.999	Q0.001	Q0.999	Q0.999	Q0.999	Q0.999
Definitely neutral	1								

C **Colony Size assay Standard methods**

Class	Probability	Corresponding cut-off values (standard method)		Corresponding cut-off values (standard with reference method)		
		Quantile	Cut-off value (cells per colony)	Quantile	Cut-off value (BRCA1 median fold)	Cut-off value (cells per colony)
5	P(X > Q0.99) = 1%	Q0.99	26,222	Q0.99	2.416	27,062
4	P(Q0.95 ≤ X ≤ Q0.99) = 4%	Q0.95	24,728	Q0.95	2.236	25,047
3	P(Q0.05 ≤ X < Q0.95) = 95%	Q0.05	13,200	Q0.05	1.173	13,143
2	P(Q0.001 ≤ X < Q0.05) = 4.9%	Q0.001	12,133	Q0.001	0.960	10,754
1	P(X < Q0.001) = 0.1%					

x 11,200

D **Colony Size assay MWW method**

Class	Probability	Corresponding cut-off values	
		Quantile	Cut-off value (p value)
5	P(X < Q0.01) = 1%	Q0.01	5.49e-5
4	P(Q0.01 ≤ X ≤ Q0.05) = 4%	Q0.05	0.00023
3	P(Q0.05 < X ≤ Q0.95) = 95%	Q0.95	0.152
2	P(Q0.95 < X ≤ Q0.999) = 4.9%	Q0.999	0.313
1	P(X > Q0.999) = 0.1%		

Figure S23. Description of the quantile system of classification

(**A**) The left figure depicts a theoretical waterfall distribution of pathogenic and neutral missense mutations, as in Fig 1A. (1) Variant classification according to the experimental best cut-off. This cut-off (horizontal black line), that maximizes the experimental sensitivity and specificity in the waterfall distribution, is obtained by ROC curve analysis, as in S2 Fig. In the case of the Colony Size assay, mutations above the best cut-off are classified as pathogenic and mutations below are classified as neutral. (2) Bootstrap analysis provides a fluctuation of the best cut-off, depending on the values of the mutations and the WT BRCA1 reference randomly chosen. The fluctuating best cut-off values form a distribution, as depicted in the schematic. (3) Quantile system of variant classification according to the fluctuation of the best cut-off. The reasoning is the following: the distribution of the fluctuating best cut-off defines quantiles (Q) that delimit the probability of the presence of this variable. As an example, the quantile Q0.99 is the value that separates the 99% lowest values from the 1% highest values in a distribution. This means that the probability to have the best cut-off above the quantile Q0.99 is 1%. Thus, in the Colony Size assay using the standard method, a mutation with the median above the quantile Q0.99 can be considered as pathogenic with a 1% probability of error. Indeed, this mutation could be neutral, but only if the best cut-off is above the median, which has a 1% probability, or less, to occur. This reasoning allows separation of the best cut-off distribution into 5 intervals, based on the five-class nomenclature proposed by Plon et al, with each interval defining the probability of the best cut-off presence within the waterfall distribution.
(**B**) Quantiles that delimit the 5 intervals of classification according to the assay and the method used. CS, Colony Size; LM, Liquid Medium; SF, Spot Formation; YL, Yeast Localization assay. Note that the quantiles differ, depending on whether the pathogenic mutations are above or below the best cut-off. For instance, in the standard method, the quantiles of the Colony Size assay are Q0.99, Q0.95, Q0.05 and Q0.001 (pathogenic mutants above the best cut-off), while quantiles are Q0.01, Q0.05, Q0.95 and Q0.999 in the Spot Formation assay (pathogenic mutants below the best cut-off). However, these two cases generate the same intervals (e.g., probability 1% for the class 5, see **C** and **D**). Cut-off values corresponding to these quantiles are listed in S5 Table for each assay and for each method.
(**C**) Interval limits in the case of the Colony Size assay, using the standard or the standard with reference method. P(X > Q0.99) = 1% is the probability to obtain the best cut-off variable X strictly over the quantile Q0.99, shown here as 26,222 cells per colony for the standard method, and 2.416 x 11,200 (BRCA1 median of the experimental data) = 27,062 cells per colony for the standard with reference method
(**D**) Interval limits in the case of the Colony Size assay, using the MWW method.

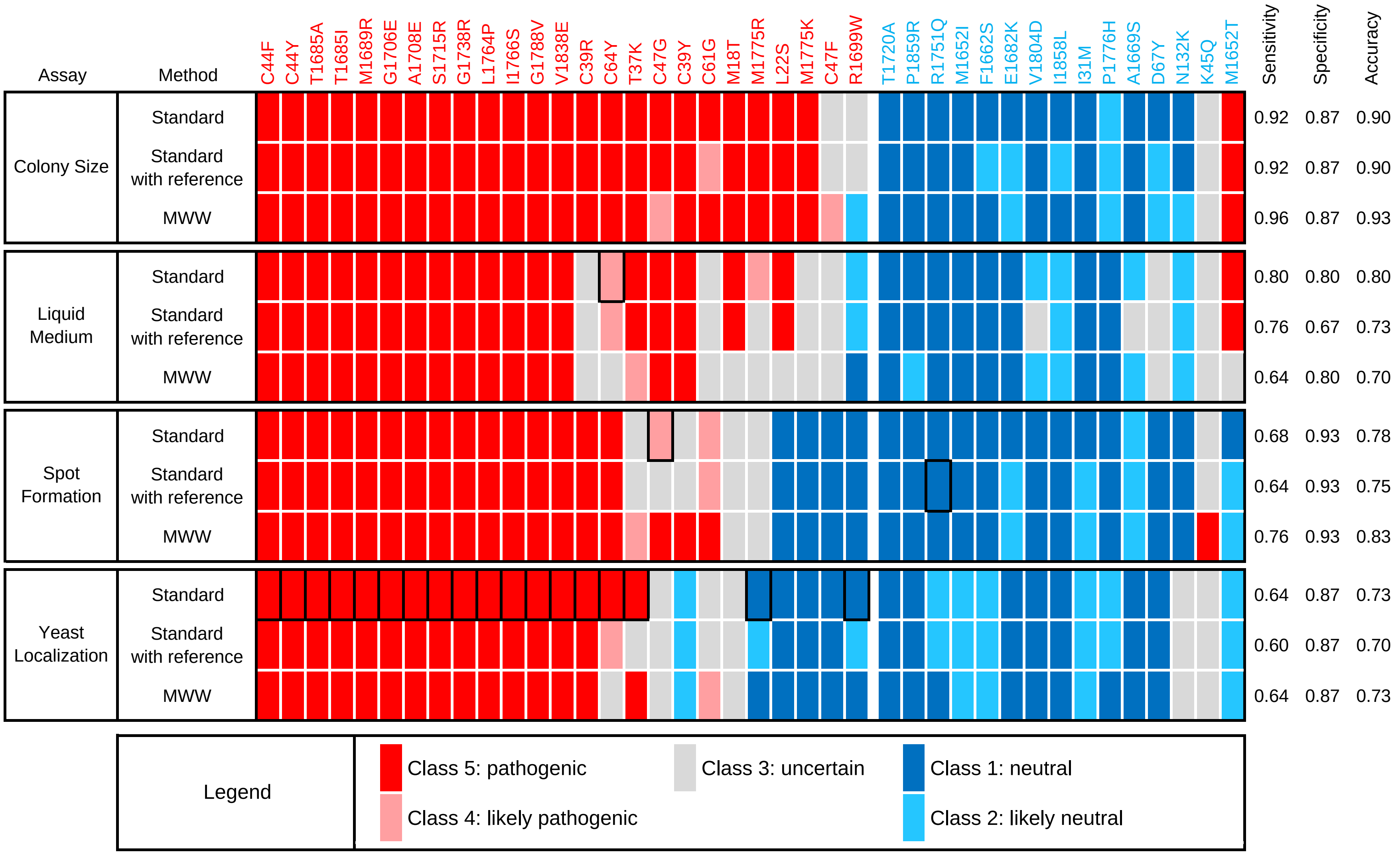

S24 Fig. Variant classification using the quantile system

Names in red and in blue indicate the pathogenic and neutral mutations, respectively, according to their prior classification. See also S16 Table. The black frames pinpoint the divergent classification compared to that in the probability system (Fig 2B).

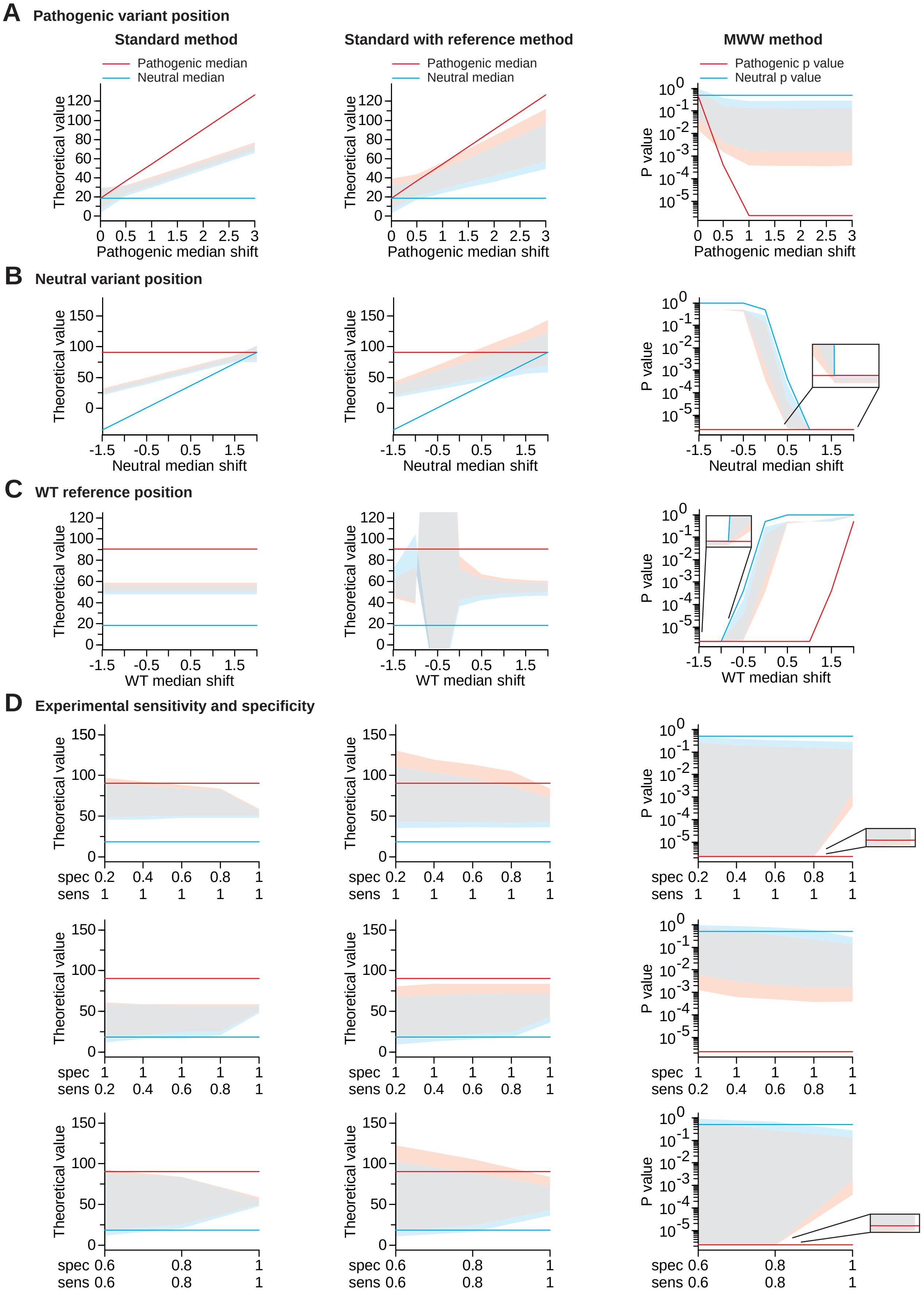

S25 Fig. Quantile system of classification (theoretical situation)

Effect of different experimental parameters was assessed in theoretical situations, exactly as for the probability system of classification, meaning that the best cut-off fluctuations depicted were those used in S13-S19 Figs. Red line, position of the median or p value of the pathogenic mutants; blue line, position of the median or p value of the neutral mutants. The pink, grey and blue areas define intervals within the best cut-off distribution, as explained in S23 Fig. For clarity, the extreme red and blue areas were not displayed. Sensitivity of the quantile system is maximal when the red line is beyond the pink area. Specificity is maximal when the blue line is beyond the light blue area. Accuracy is maximal when sensitivity and specificity are maximal. Finally, sensitivity, specificity and accuracy of the quantile system are null when both lines are in the grey area, or in the wrong side of the best cut-off fluctuation.

(**A-D**) Evolution of the best cut-off fluctuation depending on either the shift intensity of the pathogenic mutations (**A**), or the shift intensity of the neutral mutations (**B**), or the shift intensity of the WT reference (**C**), or the experimental sensitivity and specificity (**D**). The corresponding panels, depicted for the probability system of classification, are shown in S13D Fig for **A**, S14E Fig for **B**, S15E Fig for **C** and S16E-G Fig for **D**.

As summarized in S17 Table, these results did not reveal any flaws. The quantile system behaves as the probability system, in these situations.

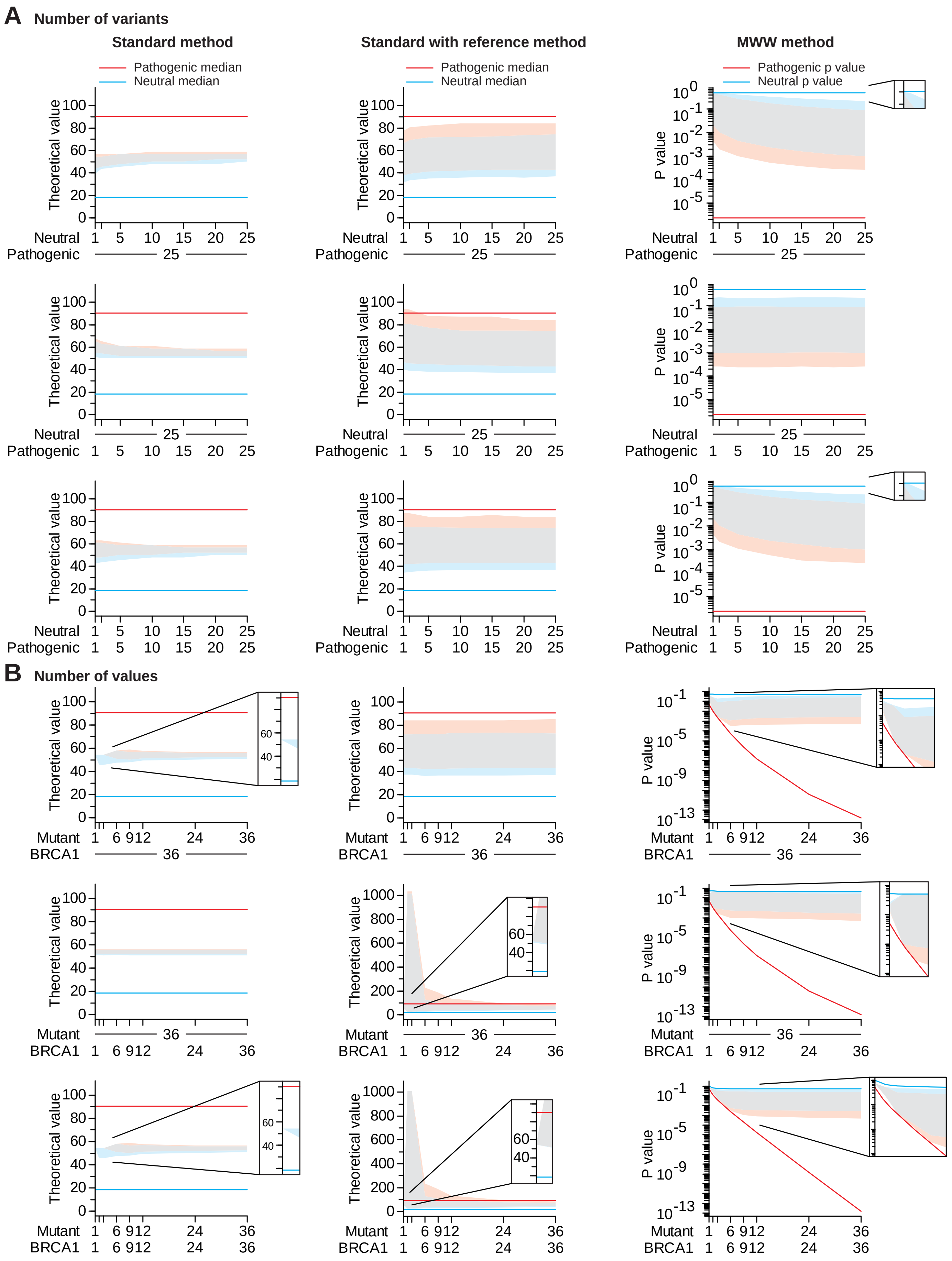

S26 Fig. Quantile system of classification (theoretical situation)

Evolution of the best cut-off fluctuation depending on either the number of neutral and pathogenic mutations (**A**) or the number of mutant and BRCA1 values (**B**), as in S25 Fig. The corresponding panels, depicted for the probability system of classification, are shown in S17E-G Fig for **A** and S18E-G Fig for **B**.

As summarized in S17 Table, these results reveal a major flaw in the quantile system of classification. Using the standard and MWW methods, the sensitivity and specificity is maximal, regardless of the number of values present within the mutant or BRCA1 distributions (**B**). Using the standard with reference method, the sensitivity is affected when the number of values in the BRCA1 distribution is decreased, but is maximal when $n_{BRCA1} = 1$. Thus, contrary to the probability system, the quantile system is not correctly influenced by the amount of experimental values resulting from functional assessment.

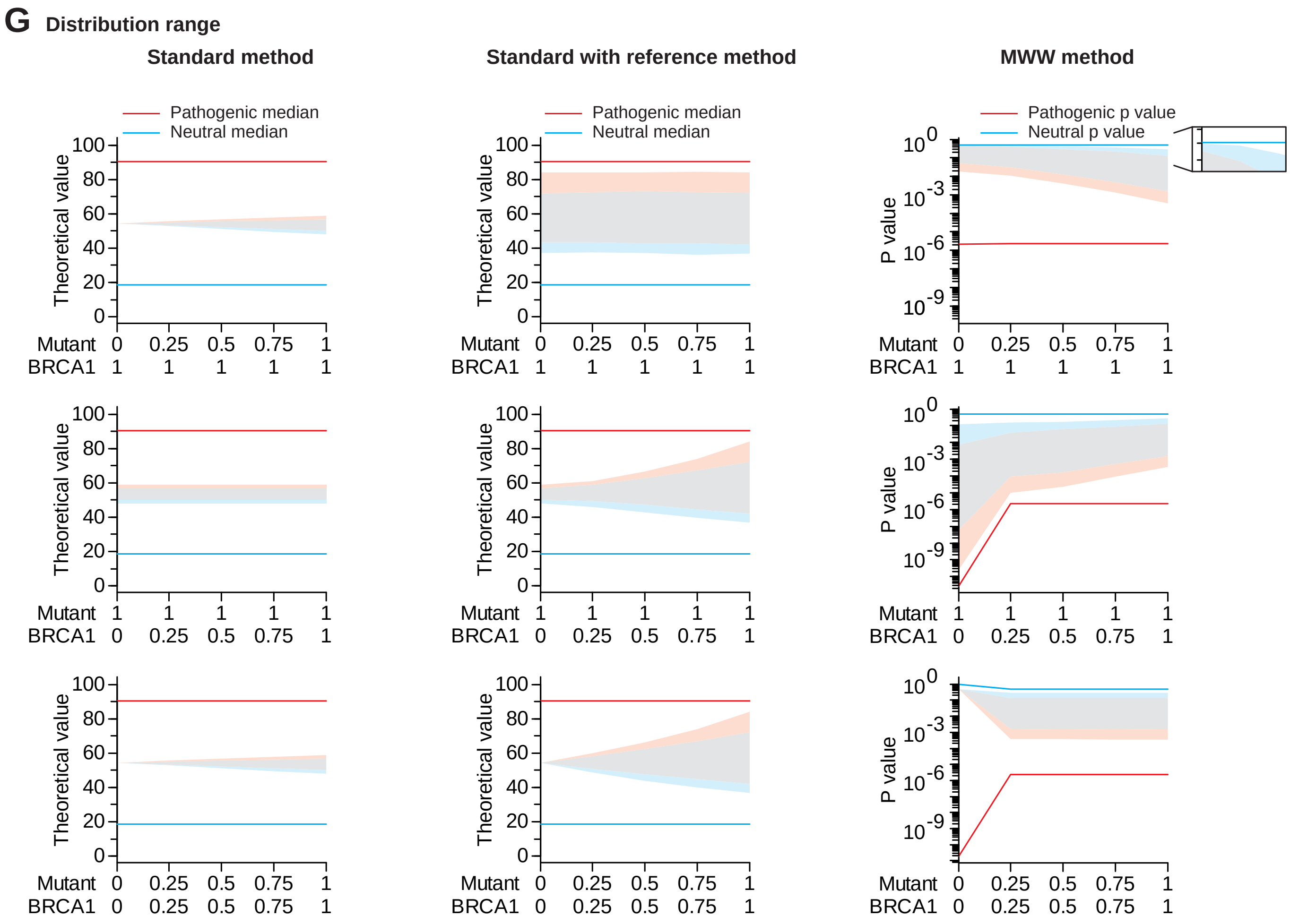

S27 Fig. Quantile system of classification (theoretical situation)
Evolution of the best cut-off fluctuation, depending on the range of the mutant and BRCA1 distributions, as in S25 Fig. The corresponding panels, depicted for the probability system of classification, are shown in S19E-G Fig.
As summarized in S17 Table, these results reveal a major flaw in the quantile system of classification. A null range means that all of the values, present in a distribution, are identical (ties). Because ties are related to a low measurement accuracy, an efficient variant classifier should penalize a high number of ties in a dataset, which is not observed here, whatever method is used.

S1 Table. IARC variant classification

Class	Description	Probability of pathogenicity	Clinical decision-making	
			Genetic testing	Surveillance recommendations
5	Definitely pathogenic	$p > 0.99$	Test at-risk relatives	Full high-risk surveillance
4	Likely pathogenic	$0.99 \geq p \geq 0.95$	Test at-risk relatives	Full high-risk surveillance
3	Uncertain (Variant of unknown significance)	$0.95 > p \geq 0.05$	Do not use as predictive testing in at-risk relatives	Counsel based on family history and other risk factors
2	Likely neutral	$0.05 > p \geq 0.001$	Do not use as predictive testing in at-risk relatives	Counsel as if no mutation detected
1	Neutral	$p < 0.001$	Do not use as predictive testing in at-risk relatives	Counsel as if no mutation detected

Five-class nomenclature proposed by the International Agency for Research on Cancer (IARC) for variant classification, with specific recommendations for clinical management, depending on the probability of pathogenicity obtained by epidemiological methods (Plon et al., 2008).

S2 Table. BRCA1 Mutations selected [a]

Variant	Nomenclature HGVS [b]	DNA Nomenclature HGVS	UMD-BRCA1 [c, d]	LOVD-IARC [c, e]	LOVD Leiden [f]	Millot et. al., 2012 [c, g]
M18T	p.Met18Thr	c.53T>C	3	4	Ambiguous	4
L22S	p.Leu22Ser	c.65T>C		5	Predicted pathogenic	5
I31M	p.Ile31Met	c.93C>G			Predicted neutral	
T37K	p.Thr37Lys	c.110C>A		5	Predicted pathogenic	5
C39R	p.Cys39Arg	c.115T>C	5	5	As mutant control	5
C39Y	p.Cys39Tyr	c.116G>A			Impact on protein function	
C44F	p.Cys44Phe	c.131G>T	5		Predicted pathogenic	
C44Y	p.Cys44Tyr	c.131G>A	5	5		5
K45Q	p.Lys45Gln	c.133A>C	2	1	Predicted neutral	1
C47F	p.Cys47Phe	c.140G>T	5			
C47G	p.Cys47Gly	c.139T>G			Predicted pathogenic	
C61G	p.Cys61Gly	c.181T>G	5	5	Ambiguous	5
C64Y	p.Cys64Tyr	c.191G>A	5			4 / 5
D67Y	p.Asp67Tyr	c.199G>T	1	1	Predicted neutral	1
N132K	p.Asn132Lys	c.396C>A	2	1	Ambiguous	1
M1652I	p.Met1652Ile	c.4956G>A	1	1	Ambiguous	1
M1652T	p.Met1652Thr	c.4955T>C	2	1	Ambiguous	1
F1662S	p.Phe1662Ser	c.4985T>C			Predicted neutral	1
A1669S	p.Ala1669Ser	c.5005G>T	3		Predicted neutral	
E1682K	p.Glu1682Lys	c.5044G>A		1	Predicted neutral	1
T1685A	p.Thr1685Ala	c.5053A>G	3	5	Predicted pathogenic	5
T1685I	p.Thr1685Ile	c.5054C>T		5	Predicted pathogenic	5
M1689R	p.Met1689Arg	c.5066T>G		4	Predicted pathogenic	4
R1699W	p.Arg1699Trp	c.5095C>T	5	5	Ambiguous	5
G1706E	p.Gly1706Glu	c.5117G>A	4		Predicted pathogenic	5
A1708E	p.Ala1708Glu	c.5123C>A	5	5	Ambiguous	5
S1715R	p.Ser1715Arg	c.5143A>C		5	Predicted pathogenic	5
T1720A	p.Thr1720Ala	c.5158A>G	1	1	Predicted neutral	1
G1738R	p.Gly1738Arg	c.5212G>A		5	Predicted pathogenic	5
R1751Q	p.Arg1751Gln	c.5252G>A	2	1	Ambiguous	1
L1764P	p.Leu1764Pro	c.5291T>C	3	5	Ambiguous	5
I1766S	p.Ile1766Ser	c.5297T>G		5	Ambiguous	5
M1775K	p.Met1775Lys	c.5324T>A		5	Ambiguous	5
M1775R	p.Met1775Arg	c.5324T>G	5	5	Ambiguous	5
P1776H	p.Pro1776His	c.5327C>A		2	Predicted neutral	2
G1788V	p.Gly1788Val	c.5363G>T		5	Predicted pathogenic	5
V1804D	p.Val1804Asp	c.5411T>A	1	1	Ambiguous	1
V1838E	p.Val1838Glu	c.5513T>A		5	Predicted pathogenic	5
I1858L	p.Ile1858Leu	c.5572A>C	2	1	Predicted neutral	1
P1859R	p.Pro1859Arg	c.5576C>G		1	Predicted neutral	1

[a] Empty cell, no data

[b] HGVS: human genome variation society (http://www.hgvs.org/mutnomen/).

[c] IARC classification as in Table S1.

[d] UMD-BRCA1 database (29-January-2015, http://www.umd.be/BRCA1/).

[e] LOVD-IARC database (29-January-2015, http://hci-exlovd.hci.utah.edu/home.php?select_db=BRCA1).

[f] LOVD Leiden database (29-January-2015, http://databases.lovd.nl/shared/genes/BRCA1). This database gathers all information from the literature, including functional assays, which explains the high level of ambiguous results. Blue cell, neutral; red cell, pathogenic; grey cell, conflicting reports.

[g] Mutations recommended by the ENIGMA consortium. C64Y is namely classified as "Clinically important" which is here converted to "4/5".

S3 Table. Misclassified mutations using the experimental best cut-offs

Assay	Standard method	MWW method
Colony Size	M1652T<br>R1699W	M1652T<br>R1699W
Liquid Medium	M1652T<br>C47F<br>R1699W<br>M1775K	L22S<br>C39R<br>C47F<br>C61G<br>R1699W
Spot Formation	K45Q<br>L22S<br>C47F<br>R1699W<br>M1775K	K45Q<br>L22S<br>C47F<br>R1699W<br>M1775K
Yeast Localization	K45Q<br>L22S<br>C39Y<br>C47F<br>R1699W<br>M1775K<br>M1775R	K45Q<br>L22S<br>C39Y<br>C47F<br>R1699W<br>M1775K<br>M1775R

Experimental best cut-offs from Table 1.

S4 Table. Experimental data from the 4 functional assays and the siRNA screening

Assay	Name	Median	Relative median	MWW p value	Sample size	Ties
	BRCA1	11 200	1		n = 36	2,4,2
	Vector	4 146 055	370.18348	1.515234e-13	n = 36	0
	L1764P	328 807	29.35777	2.274705e-06	n = 9	2
	M1689R	1 877 333	167.61902	2.276350e-06	n = 9	0
	V1838E	1 621 333	144.76188	2.276350e-06	n = 9	0
	G1706E	1 536 000	137.14286	2.276350e-06	n = 9	0
	T1685I	1 354 667	120.95241	2.276350e-06	n = 9	0
	A1708E	924 771	82.56884	2.276350e-06	n = 9	0
	S1715R	878 291	78.41884	2.276350e-06	n = 9	0
	T1685A	760 367	67.88991	2.276350e-06	n = 9	0
	G1788V	531 282	47.43589	2.276350e-06	n = 9	0
	I1766S	416 410	37.17946	2.276350e-06	n = 9	0
	C44F	145 000	12.94643	2.276350e-06	n = 9	0
	C44Y	117 651	10.50455	2.276350e-06	n = 9	0
	G1738R	92 855	8.29063	2.276350e-06	n = 9	0
	C39Y	79 733	7.11902	2.276350e-06	n = 9	0
	C64Y	60 624	5.41286	2.276350e-06	n = 9	0
	C39R	49 867	4.45241	2.276350e-06	n = 9	0
	M1775R	49 299	4.40170	2.276350e-06	n = 9	0
	M1652T	45 108	4.02750	2.276350e-06	n = 9	0
Colony Size	M1775K	42 120	3.76071	2.276350e-06	n = 9	0
	L22S	38 507	3.43813	2.276350e-06	n = 9	0
	C61G	27 024	2.41286	2.606554e-06	n = 9	0
	T37K	28 053	2.50473	4.446440e-06	n = 9	0
	M18T	29 182	2.60554	2.951174e-05	n = 9	0
	C47G	29 000	2.58929	1.323210e-04	n = 9	0
	C47F	17 920	1.60000	1.835069e-04	n = 9	2
	K45Q	17 900	1.59821	0.0184710518614082525	n = 9	0
	R1699W	13 300	1.18750	0.2302354198061955237	n = 9	2
	D67Y	11 520	1.02857	0.4604291821344998481	n = 9	0
	E1682K	11 303	1.00920	0.4660662497636753332	n = 9	2
	N132K	10 200	0.91071	0.4943379145802865859	n = 9	0
	P1776H	12 253	1.09402	0.5169816950327198457	n = 9	0
	F1662S	11 966	1.06839	0.6279557960277021289	n = 9	0
	T1720A	9 042	0.80732	0.6492252229136958874	n = 9	2
	I1858L	10 773	0.96188	0.6853049210510774314	n = 9	2
	A1669S	10 243	0.91455	0.7566189006559524977	n = 9	0
	P1859R	10 300	0.91964	0.8465966574550906065	n = 9	2
	M1652I	10 070	0.89911	0.8532174576053266346	n = 9	2
	R1751Q	9 300	0.83036	0.9619810348434899572	n = 9	0
	I31M	7 600	0.67857	0.9619810348434899572	n = 9	0
	V1804D	6 239	0.55705	0.9976319137889332334	n = 9	0

	BRCA1	0.144	1		n = 36	2, 2, 2, 4, 2, 2, 2, 2
	Vector	0.448	3.11111	1.510622e-13	n = 36	2, 2
	A1708E	0.368	2.55556	2.264851e-06	n = 9	2, 2
	C44F	0.239	1.65972	2.264851e-06	n = 9	2, 2
	G1788V	0.407	2.82639	2.266490e-06	n = 9	2
	G1706E	0.397	2.75694	2.266490e-06	n = 9	2
	I1766S	0.393	2.72917	2.266490e-06	n = 9	2
	M1689R	0.388	2.69444	2.266490e-06	n = 9	2
	T1685I	0.387	2.68750	2.266490e-06	n = 9	2
	T1685A	0.338	2.34722	2.266490e-06	n = 9	2
	C44Y	0.204	1.41667	2.266490e-06	n = 9	2
	V1838E	0.405	2.81250	2.268131e-06	n = 9	0
	S1715R	0.387	2.68750	2.268131e-06	n = 9	0
	L1764P	0.361	2.50694	2.268131e-06	n = 9	0
	G1738R	0.295	2.04861	2.268131e-06	n = 9	0
	C39Y	0.205	1.42361	2.268131e-06	n = 9	0
	C47G	0.186	1.29167	1.594213e-05	n = 9	2, 2
	T37K	0.190	1.31944	2.606480e-05	n = 9	2
	M18T	0.184	1.27778	7.096329e-05	n = 9	0
	M1775R	0.177	1.22917	1.116480e-04	n = 9	2
Liquid Medium	C64Y	0.182	1.26389	1.641808e-04	n = 9	0
	M1775K	0.170	1.18056	2.152737e-04	n = 9	0
	K45Q	0.172	1.19444	2.526677e-04	n = 9	0
	C61G	0.173	1.20139	3.118077e-04	n = 9	0
	M1652T	0.195	1.35417	3.279441e-04	n = 9	0
	L22S	0.162	1.12500	6.346827e-04	n = 9	2
	D67Y	0.161	1.11806	0.00431088681736 17884	n = 9	2
	C39R	0.174	1.20833	0.0048755976324813856	n = 9	0
	C47F	0.161	1.11806	0.0057497334195825249	n = 9	2
	N132K	0.152	1.05556	0.1136660718705307938	n = 9	0
	I1858L	0.152	1.05556	0.1434656399958101347	n = 9	0
	P1859R	0.144	1.00000	0.1853384111507014043	n = 9	2
	V1804D	0.158	1.09722	0.2568378526562776765	n = 9	0
	A1669S	0.158	1.09722	0.2568447967519839059	n = 9	0
	E1682K	0.148	1.02778	0.3613056803591045973	n = 9	0
	R1699W	0.154	1.06944	0.4491694377259908277	n = 9	0
	T1720A	0.142	0.98611	0.4830127077145046077	n = 9	2
	M1652I	0.141	0.97917	0.8068922049917628403	n = 9	2, 2
	I31M	0.139	0.96528	0.8220402013577042810	n = 9	0
	P1776H	0.134	0.93056	0.8835070202068154854	n = 9	0
	F1662S	0.130	0.90278	0.9487627758129135724	n = 9	2
	R1751Q	0.128	0.88889	0.9979400197432480857	n = 9	3, 2

	BRCA1	0.32011331	1		n = 12	0
	I1858L	0.46986945	1.46782	0.994594772	n = 3	0
	D67Y	0.39541215	1.23523	0.994594772	n = 3	0
	L22S	0.40228548	1.25670	0.991872140	n = 3	0
	M1775K	0.37789528	1.18050	0.991872140	n = 3	0
	N132K	0.37003058	1.15594	0.991872140	n = 3	0
	R1699W	0.3539823	1.10580	0.982649712	n = 3	0
	C47F	0.33136225	1.03514	0.936900008	n = 3	0
	M1652I	0.33056577	1.03265	0.788446042	n = 3	0
	F1662S	0.32405386	1.01231	0.788446042	n = 3	0
	V1804D	0.35462842	1.10782	0.743893964	n = 3	0
	T1720A	0.34723473	1.08472	0.743893964	n = 3	0
	R1751Q	0.32063492	1.00163	0.743893964	n = 3	0
	P1776H	0.34312296	1.07188	0.694887867	n = 3	0
	P1859R	0.32621083	1.01905	0.694887867	n = 3	0
	E1682K	0.31781733	0.99283	0.470973919	n = 3	0
	M1652T	0.31916635	0.99704	0.357888106	n = 3	0
	I31M	0.31843575	0.99476	0.357888106	n = 3	0
	A1669S	0.3021984	0.94404	0.305112133	n = 3	0
	M1775R	0.2877229	0.89882	0.024638246	n = 3	0
Spot Formation	M18T	0.23936689	0.74776	0.017350288	n = 3	0
	T37K	0.24777513	0.77402	0.008127860	n = 3	0
	C39Y	0.24834054	0.77579	0.005405228	n = 3	0
	K45Q	0.23125	0.72240	0.005405228	n = 3	0
	C47G	0.22693483	0.70892	0.005405228	n = 3	0
	C61G	0.22481563	0.70230	0.005405228	n = 3	0
	G1738R	0.20485484	0.63994	0.005405228	n = 3	0
	C64Y	0.20008394	0.62504	0.005405228	n = 3	0
	V1838E	0.19506031	0.60935	0.005405228	n = 3	0
	C44Y	0.18793555	0.58709	0.005405228	n = 3	0
	G1788V	0.17286119	0.54000	0.005405228	n = 3	0
	L1764P	0.1658962	0.51824	0.005405228	n = 3	0
	T1685I	0.1631555	0.50968	0.005405228	n = 3	0
	C39R	0.15864589	0.49559	0.005405228	n = 3	0
	C44F	0.13445378	0.42002	0.005405228	n = 3	0
	I1766S	0.13080603	0.40862	0.005405228	n = 3	0
	T1685A	0.10702164	0.33432	0.005405228	n = 3	0
	A1708E	0.09740219	0.30427	0.005405228	n = 3	0
	M1689R	0.09630668	0.30085	0.005405228	n = 3	0
	S1715R	0.09555141	0.29849	0.005405228	n = 3	0
	G1706E	0.08198442	0.25611	0.005405228	n = 3	0

Yeast Localization	BRCA1	0.03011169	1		n = 12	0
	V1838E	1	33.20969	0.005733011	n = 3	2
	G1788V	0.997409639	33.12367	0.005769994	n = 3	0
	S1715R	0.994699804	33.03368	0.005769994	n = 3	0
	G1706E	0.984477893	32.69421	0.005769994	n = 3	0
	A1708E	0.973875384	32.34210	0.005769994	n = 3	0
	I1766S	0.968397449	32.16018	0.005769994	n = 3	0
	M1689R	0.781520693	25.95406	0.005769994	n = 3	0
	L1764P	0.743579501	24.69405	0.005769994	n = 3	0
	C39R	0.471397699	15.65497	0.005769994	n = 3	0
	G1738R	0.445016465	14.77886	0.005769994	n = 3	0
	T1685I	0.439432238	14.59341	0.005769994	n = 3	0
	T1685A	0.412329078	13.69332	0.005769994	n = 3	0
	C44Y	0.392917208	13.04866	0.005769994	n = 3	0
	C44F	0.375284774	12.46309	0.005769994	n = 3	0
	T37K	0.231694699	7.69451	0.005769994	n = 3	0
	C61G	0.136232412	4.52424	0.008619510	n = 3	0
	C64Y	0.338020871	11.22557	0.035598445	n = 3	0
	K45Q	0.07699986	2.55714	0.035598445	n = 3	0
	M18T	0.058713945	1.94987	0.048469094	n = 3	0
	C47G	0.065395263	2.17176	0.064817290	n = 3	0
	N132K	0.055921083	1.85712	0.306715686	n = 3	0
	M1652T	0.033521303	1.11323	0.414296538	n = 3	0
	F1662S	0.033474118	1.11167	0.585703462	n = 3	0
	C39Y	0.019811089	0.65792	0.640891935	n = 3	0
	M1652I	0.019508914	0.64789	0.693284314	n = 3	0
	I31M	0.017313313	0.57497	0.741998532	n = 3	0
	P1776H	0.020958299	0.69602	0.825927688	n = 3	0
	A1669S	0.007331425	0.24347	0.825927688	n = 3	0
	R1751Q	0.018504379	0.61452	0.860491843	n = 3	0
	T1720A	0.004224351	0.14029	0.860491843	n = 3	0
	R1699W	0.014217152	0.47215	0.890064145	n = 3	0
	P1859R	0.002212185	0.07347	0.964401555	n = 3	0
	M1775R	0.013772868	0.45739	0.974325834	n = 3	0
	C47F	0.010335247	0.34323	0.974325834	n = 3	0
	E1682K	0.004404384	0.14627	0.987364419	n = 3	0
	L22S	0.00298102	0.09900	0.987364419	n = 3	0
	D67Y	0.007577277	0.25164	0.994230006	n = 3	0
	M1775K	0.00712358	0.23657	0.994230006	n = 3	0
	I1858L	0.002357164	0.07828	0.996210204	n = 3	0
	V1804D	0.001647348	0.05471	0.996210204	n = 3	0
siRNA	No siRNA	945	1		n = 1,140	810 values at least twice
	KIF11	206	0.21799	2.78207E-07	n = 12	0
	GL2	1002	1.06032	0.712549331	n = 12	0
	GOLGA2	1091.636	1.15517	0.981009026	n = 12	0
	GTSE1	843	0.89206	0.005326083	n = 12	0
	ITGA2	840.5	0.88942	0.623738113	n = 12	0
	KCNC3	822.5	0.87037	0.019275675	n = 12	0
	LIN7A	1086.5	1.14974	0.957604398	n = 12	0
	PSMB1	625	0.66138	6.61498E-05	n = 12	0
	RHOBTB3	817.5	0.86508	0.318506324	n = 12	0
	RRM2	418	0.44233	6.51502E-09	n = 12	0
	SOX13	972	1.02857	0.623738124	n = 12	0

Relative median, median divided by the WT BRCA1 median or by the No siRNA median; sample size, number of values; framed mutations indicate identical p values. The "ties" column indicates the number of values repeated. For instance, in the first row, one value is repeated twice, another one four times, and a third twice ("ties" is the statistical term used to designate "identical values").

S5 Table. Distribution of the best cut-offs after bootstrap analysis

Method	Assay	Q0.001	Q0.005	Q0.010	Q0.025	Q0.050	Median (Q0.5)	Q0.950	Q0.975	Q0.990	Q0.995	Q0.999
Standard	Colony Size	12 133	12 276	12 633	12 969	13 200	17 950	24 728	25 894	26 222	26 979	27 464
	Liquid Medium	0.151	0.153	0.153	0.156	0.159	0.168	0.176	0.178	0.182	0.183	0.188
	Spot Formation	0.218	0.222	0.222	0.224	0.227	0.266	0.300	0.309	0.315	0.315	0.315
	Yeast Localization	0.015	0.020	0.034	0.035	0.054	0.125	0.212	0.215	0.215	0.215	0.215
	siRNA	434	467	480	504	523	599	658	668	679	688	704
Standard with reference	Colony Size	0.960	1.038	1.071	1.128	1.173	1.603	2.236	2.324	2.416	2.468	2.650
	Liquid Medium	1.038	1.052	1.060	1.078	1.096	1.161	1.231	1.250	1.267	1.275	1.309
	Spot Formation	0.664	0.680	0.687	0.696	0.706	0.830	0.942	0.964	0.983	0.984	1.000
	Yeast Localization	0.399	0.669	0.819	1.133	1.324	4.239	9.785	10.528	12.143	13.774	16.191
	siRNA	0.460	0.493	0.508	0.532	0.553	0.634	0.698	0.709	0.721	0.730	0.746
Standard with reference (value multiplied by the BRCA1 median)	Colony Size	10 754	11 626	11 993	12 630	13 143	17 950	25 047	26 034	27 062	27 644	29 678
	Liquid Medium	0.149	0.152	0.153	0.155	0.158	0.167	0.177	0.180	0.182	0.184	0.189
	Spot Formation	0.212	0.218	0.220	0.223	0.226	0.266	0.301	0.309	0.315	0.315	0.320
	Yeast Localization	0.012	0.020	0.025	0.034	0.040	0.128	0.295	0.317	0.366	0.415	0.488
	siRNA	434	466	480	503	523	599	659	670	681	690	705
MWW	Colony Size	1.08E-05	3.28E-05	5.49E-05	0.00013	0.00023	0.010	0.152	0.222	0.313	0.381	0.522
	Liquid Medium	6.31E-06	1.38E-05	1.94E-05	3.39E-05	5.72E-05	0.0015	0.045	0.075	0.123	0.166	0.268
	Spot Formation	0.0039	0.0053	0.0062	0.0081	0.010	0.062	0.217	0.271	0.344	0.386	0.473
	Yeast Localization	0.0065	0.0068	0.0069	0.0088	0.010	0.063	0.341	0.462	0.613	0.694	0.785
	siRNA	3.3E-06	6.43E-06	8.73E-06	1.34E-05	1.83E-05	0.000398	0.025	0.030	0.088	0.121	0.252

Values obtained after sampling, with replacement, using the original data obtained from the four functional assays and the siRNA screen (see the bootstrap procedure A in the method section). Q indicates the quantile (Q0.050 is the quantile 5%). For the standard with reference method, the results shown represent values either relative to the median of the WT reference (BRCA1 reference or No siRNA), which allows an immediate comparison of the cut-off distributions between the different assays (top), or the same values multiplied by the experimental median of the WT reference observed in the corresponding assay (bottom). For example, 0.960 is the quantile 0.1% in the Colony Size assay, which corresponds to 11,200 x 0.960 = 10,754 cells per colony. Experimental medians used are 11,200 cells per colony (Colony Size assay), 0.144 OD600 (Liquid Medium assay), 32% (Spot Formation assay), 3% (Yeast Localization assay) and 945 (siRNA screen), as indicated in S4 Table. Of note, in the standard method, the WT reference was ignored during the sampling. Thus, the resulting fluctuation of the best cut-offs does not depend on the fluctuation of the WT reference. This explains why distributions were narrowed in the standard method, compared to the standard with reference method. For example, 12,133 cells per colony is the quantile 0.1% in the Colony Size assay, but in the standard with reference method, the same quantile is slightly farther from the distribution median, with a value of 11,200 x 0.960 = 10,754 cells per colony.

S6 Table. Quantitative analysis of Fig 2B

Assay	Method	Number of variants correctly classified					Number of variants not classified			Number of variants misclassified			Balance	Sensitivity	Specificity	Accuracy
		Pathogenic in class 5	Pathogenic in class 5 + 4	Neutral in class 1	Neutral in class 1 + 2	Total (class 1 + 2 + 4 + 5)	Pathogenic in class 3	Neutral in class 3	Total	Pathogenic in class 1 or 2	Neutral in class 5 or 4	Total		Pathogenic in class 5 + 4	Neutral in class 1 + 2	Total (class 1 + 2 + 4 + 5)
Colony Size	Standard	23	23	12	13	36	2	1	3	0	1	1	35	0.92	0.87	0.90
	Standard with reference	22	23	8	13	36	2	1	3	0	1	1	35	0.92	0.87	0.90
	MWW	22	24	9	13	37	0	1	1	1	1	2	35	0.96	0.87	0.93
Liquid Medium	Standard	19	20	8	12	32	4	2	6	1	1	2	30	0.80	0.80	0.80
	Standard with reference	18	19	8	10	29	5	4	9	1	1	2	27	0.76	0.67	0.73
	MWW	15	16	7	12	28	8	3	11	1	0	1	27	0.64	0.80	0.70
Spot Formation	Standard	15	16	13	14	30	5	1	6	4	0	4	26	0.64	0.93	0.75
	Standard with reference	15	16	9	14	30	5	1	6	4	0	4	26	0.64	0.93	0.75
	MWW	18	19	10	14	33	2	0	2	4	1	5	28	0.76	0.93	0.83
Yeast Localization	Standard	0	16	7	13	29	3	2	5	6	0	6	23	0.64	0.87	0.73
	Standard with reference	14	15	7	13	28	4	2	6	6	0	6	22	0.60	0.87	0.70
	MWW	15	16	9	13	29	3	2	5	6	0	6	23	0.64	0.87	0.73

"Balance" indicates the number of mutations correctly classified (class 1 + 2 + 4 + 5) minus the total number of mutations misclassified. "Sensitivity" and "Specificity" represent the number of variants correctly classified, divided by the number of variants in the prior classification ($n_{pathogenic}$ = 25 and $n_{neutral}$ = 15). "Accuracy" is the number of mutations correctly classified (class 1 + 2 + 4 + 5) divided by the total number of mutations (n = 40).

S7 Table. Features of the assays

Assay	Features
Colony Size	n_{BRCA1} = 36 and n_{Mutant} = 9<br>3 mutant clones assessed in 3 independent experiments (9 values)<br>High dynamic range of the measurement (Vector median / BRCA1 median > 100)<br>A few ties in the BRCA1 distribution
Liquid Medium	n_{BRCA1} = 36 and n_{Mutant} = 9<br>3 mutant clones assessed in 3 independent experiments (9 values)<br>Low dynamic range of the measurement (Vector median / BRCA1 median < 4)<br>Many ties in the BRCA1 distribution and a few ties in mutant distributions<br>Weak precision of the measurement (3 digits max given by the spectrophotometer)
Spot Formation	n_{BRCA1} = 12 and n_{Mutant} = 3<br>3 mutant clones assessed independently (3 values)<br>Proportion (measurement between 0 and 1)<br>No ties<br>Opposite orientation (pathogenic mutations below the WT BRCA1 reference)
Yeast Localization	n_{BRCA1} = 12 and n_{Mutant} = 3<br>3 mutant clones assessed independently (3 values)<br>Proportion (measurement between 0 and 1)<br>BRCA1 reference median close to 0<br>Almost no ties
siRNA	$n_{reference}$ = 1,140 and n_{siRNA} = 12<br>Quadriplicates in plates and 3 independent experiments (12 values)<br>One positive (pathogenic) and 2 negative (neutral) controls<br>Many ties in the "No siRNA" reference<br>Opposite orientation (KIF11 positive control below the "No siRNA" reference)

See S4 Table for details.

S8 Table. Examples of theoretical situations analyzed

	Reference situation			Pathogenic distributions shifted (Figure S13)			Neutral distributions shifted (Figure S14)			WT BRCA1 distributions shifted (Figure S15)			Number of values modified (Figure S18)			Range modified (Figure S19)		
	BRCA1	neutral mutants	pathogenic mutants	BRCA1	neutral mutants	pathogenic mutants	BRCA1	neutral mutants	pathogenic mutants	BRCA1	neutral mutants	pathogenic mutants	BRCA1	neutral mutants	pathogenic mutants	BRCA1	neutral mutants	pathogenic mutants
	1	1	73	1	1	1	1	37	73	37	1	73	1	1	73	9.75	9.75	81.75
	2	5.38	77.38	2	5.38	5.38	2	41.38	77.38	38	5.38	77.38	18.5	18.5	90.5	10.25	11.9375	83.9375
	3	9.75	81.75	3	9.75	9.75	3	45.75	81.75	39	9.75	81.75	36	36	108	10.75	14.125	86.125
	4	14.13	86.13	4	14.13	14.13	4	50.13	86.13	40	14.13	86.13				11.25	16.3125	88.3125
	5	18.50	90.50	5	18.50	18.50	5	54.50	90.50	41	18.50	90.50				11.75	18.5	90.5
	6	22.88	94.88	6	22.88	22.88	6	58.88	94.88	42	22.88	94.88				12.25	20.6875	92.6875
	7	27.25	99.25	7	27.25	27.25	7	63.25	99.25	43	27.25	99.25				12.75	22.875	94.875
	8	31.63	103.63	8	31.63	31.63	8	67.63	103.63	44	31.63	103.63				13.25	25.0625	97.0625
	9	36	108	9	36	36	9	72	108	45	36	108				13.75	27.25	99.25
	10			10			10			46						14.25		
	11			11			11			47						14.75		
	12			12			12			48						15.25		
	13			13			13			49						15.75		
	14			14			14			50						16.25		
	15			15			15			51						16.75		
	16			16			16			52						17.25		
	17			17			17			53						17.75		
Values	18			18			18			54						18.25		
	19			19			19			55						18.75		
	20			20			20			56						19.25		
	21			21			21			57						19.75		
	22			22			22			58						20.25		
	23			23			23			59						20.75		
	24			24			24			60						21.25		
	25			25			25			61						21.75		
	26			26			26			62						22.25		
	27			27			27			63						22.75		
	28			28			28			64						23.25		
	29			29			29			65						23.75		
	30			30			30			66						24.25		
	31			31			31			67						24.75		
	32			32			32			68						25.25		
	33			33			33			69						25.75		
	34			34			34			70						26.25		
	35			35			35			71						26.75		
	36			36			36			72						27.25		
n	36	9	9	36	9	9	36	9	9	36	9	9	3	3	3	36	9	9
Median	18.5	18.5	90.5	18.5	18.5	18.5	18.5	54.5	90.5	54.5	18.5	90.5	18.5	18.5	90.5	18.5	18.5	90.5
Mean	18.5	18.5	90.5	18.5	18.5	18.5	18.5	54.5	90.5	54.5	18.5	90.5	18.5	18.5	90.5	18.5	18.5	90.5
Separating factor s BRCA1	0			0			0			1			0			0		
Separating factor s neutral		0			0			1			0			0			0	
Separating factor s pathogenic			2			0			2			2			2			2
Min value	1	1	73	1	1	1	1	37	73	37	1	73	1	1	73	9.75	9.75	81.75
Max value	36	36	108	36	36	36	36	72	108	72	36	108	36	36	108	27.25	27.25	99.25
Range	35	35	35	35	35	35	35	35	35	35	35	35	35	35	35	17.5	17.5	17.5
Range factor r	1	1	1	1	1	1	1	1	1	1	1	1	1	1	1	0.5	0.5	0.5

The "reference situation" column indicates the initial values and parameter settings. From this, distribution parameters were modified and the resulting variant classification was scrutinized. The separating factor s (BRCA1, neutral or pathogenic) shifts the values of the distribution, according to the formula $v_{ij} + 36 \times s$, with v_{ij} representing the value i of the distribution j (BRCA1, neutral or pathogenic). The range factor r modulates the extreme values of a distribution, according to the formula me ± 17.5 × r, where me represents the median of the distribution. Whatever the value of r, the values of any distribution are equally spread.

S9 Table. Effect of functional assay parameters upon the probability system of classification

Parameters	Standard method	Standard with reference method	MWW method	Figure
Position of pathogenic mutations	**+++**<br>Shift from the neutral sector towards the pathogenic sector increases both sensitivity and specificity.	**+++**<br>As in the standard method.	**+++**<br>As in the standard method.	S13
Position of neutral mutations	**+++**<br>Shift from the neutral sector towards the pathogenic sector decreases both sensitivity and specificity.	**+++**<br>As in the standard method.	**+++**<br>No effect, except a complete misclassification of the pathogenic mutations when the WT reference distribution is left on one side of all the neutral and pathogenic distributions with no overlap.	S14
Position of WT reference	**0**<br>No effect.	**+++**<br>Shift of the median towards the null value decreases sensitivity and specificity. Negative values of the WT reference median invert the classification of the neutral and pathogenic mutations.	**+++**<br>No effect, except a complete misclassification of the pathogenic mutations when the WT reference distribution falls outside of the range of the neutral and pathogenic distributions, with no overlap.	S15
Experimental sensitivity	**+++**<br>Decrease of the experimental sensitivity decreases specificity. This is advantageous, as the neutral region is contaminated by pathogenic mutations (presence of false negatives).	**+++**<br>As in the standard method.	**+++**<br>As in the standard method.	S16
Experimental specificity	**+++**<br>Decrease of the experimental specificity decreases sensitivity. This is advantageous, as the pathogenic region is contaminated by neutral mutations (presence of false positives).	**+++**<br>As in the standard method.	**+++**<br>As in the standard method.	S16
Number of neutral mutations	**0**<br>No clear effect.	**0**<br>No clear effect.	**0**<br>No effect.	S17
Number of pathogenic mutations	**0**<br>No clear effect.	**0**<br>No clear effect.	**0**<br>No effect.	S17
Number of mutant values	**+++**<br>Decreasing number decreases both sensitivity and specificity ($p = 0.5$ when $n_{mutant} = 1$).	**+++**<br>Decreasing number decreases both sensitivity and specificity, but only when the number of BRCA1 values concomitantly decreases ($p = 0.5$ when $n_{mutant} = n_{BRCA1} = 1$). Otherwise, no effect.	**+++**<br>As in the standard with reference method.	S18
Number of WT BRCA1 values	**0**<br>No effect.	**+++**<br>Loss of sensitivity with the decrease of the number of BRCA1 values, except when $n_{BRCA1} = 1$. The concomitant decrease of n_{mutant} and n_{BRCA1} decreases both sensitivity and specificity ($p = 0.5$ when $n_{mutant} = n_{BRCA1} = 1$).	**+++**<br>As in the standard with reference method.	S18
Range of mutant distributions	**+++**<br>Decreasing range of the mutant distributions decreases both sensitivity and specificity ($p = 0.5$ when the range is null).	**+++**<br>No effect, except a strong decrease of sensitivity and specificity ($p = 0.5$) when the mutant and the BRCA1 ranges are null.	**+++**<br>As in the standard with reference method.	S19
Range of WT BRCA1 distribution	**0**<br>No effect.	**+++**<br>No effect, except a strong decrease of sensitivity and specificity ($p = 0.5$) when the mutant and the BRCA1 ranges are null.	**+++**<br>As in the standard with reference method.	S19

Sensitivity and specificity are defined here as the probabilities of pathogenicity (p) attributed to the pathogenic and neutral mutations, respectively, which reflects the accuracy of the probability system of variant classification. Sensitivity is maximal if $p \geq 0.95$ for the pathogenic mutations. Specificity is maximal if $p < 0.05$ for the neutral mutations. Misclassification is defined as $p \geq 0.95$ for the neutral mutations and $p < 0.05$ for the pathogenic mutations. The experimental sensitivity and specificity derives from the experimental best cut-off, as explained in S2 Fig. They differ from the sensitivity and specificity described above, since they are related to the initial position of the pathogenic and neutral mutants before random sampling. +++, very influenced; 0, no effect.

S10 Table. Plasmids used

Name	cDNA	HGVS nomenclature[a]	Source[b]	Name	cDNA	HGVS nomenclature[a]	Source[b]
pJL48	None (Vector)		Millot et al., 2011				
pJL45	BRCA1		Millot et al., 2011	pGM40	BRCA1-mCherry		Millot et al., 2011
pPT60	BRCA1		This study	pPT63	BRCA1-mCherry		This study
pPT185	M18T	c.53T>C	This study	pPT195	M18T-mCherry	c.53T>C	This study
pPT175	L22S	c.65T>C	This study	pPT180	L22S-mCherry	c.65T>C	This study
pPT98	I31M	c.93C>G	This study	pPT99	I31M-mCherry	c.93C>G	This study
pPT176	T37K	c.110C>A	This study	pPT181	T37K-mCherry	c.110C>A	This study
pPT177	C39R	c.115T>C	This study	pPT182	C39R-mCherry	c.115T>C	This study
pPT142	C39Y	c.116G>A	This study	pPT156	C39Y-mCherry	c.116G>A	This study
pPT101	C44F	c.131G>T	This study	pPT102	C44F-mCherry	c.131G>T	This study
pPT186	C44Y	c.131G>A	This study	pPT196	C44Y-mCherry	c.131G>A	This study
pPT104	K45Q	c.133A>C	This study	pPT105	K45Q-mCherry	c.133A>C	This study
pPT143	C47F	c.140G>T	This study	pPT157	C47F-mCherry	c.140G>T	This study
pPT107	C47G	c.139T>G	This study	pPT108	C47G-mCherry	c.139T>G	This study
pPT187	C61G	c.181T>G	This study	pPT197	C61G-mCherry	c.181T>G	This study
pPT110	C64Y	c.191G>A	This study	pPT111	C64Y-mCherry	c.191G>A	This study
pPT178	D67Y	c.199G>T	This study	pPT183	D67Y-mCherry	c.199G>T	This study
pPT113	N132K	c.396C>A	This study	pPT114	N132K-mCherry	c.396C>A	This study
pPT188	M1652I	c.4956G>A	This study	pPT198	M1652I-mCherry	c.4956G>A	This study
pPT189	M1652T	c.4955T>C	This study	pPT199	M1652T-mCherry	c.4955T>C	This study
pPT144	F1662S	c.4985T>C	This study	pPT158	F1662S-mCherry	c.4985T>C	This study
pPT145	A1669S	c.5005G>T	This study	pPT159	A1669S-mCherry	c.5005G>T	This study
pPT190	E1682K	c.5044G>A	This study	pPT200	E1682K-mCherry	c.5044G>A	This study
pPT191	T1685A	c.5053A>G	This study	pPT201	T1685A-mCherry	c.5053A>G	This study
pPT146	T1685I	c.5054C>T	This study	pPT160	T1685I-mCherry	c.5054C>T	This study
pPT152	M1689R	c.5066T>G	This study	pPT166	M1689R-mCherry	c.5066T>G	This study
pPT116	R1699W	c.5095C>T	This study	pPT117	R1699W-mCherry	c.5095C>T	This study
pPT147	G1706E	c.5117G>A	This study	pPT161	G1706E-mCherry	c.5117G>A	This study
pPT192	A1708E	c.5123C>A	This study	pPT202	A1708E-mCherry	c.5123C>A	This study
pPT153	S1715R	c.5143A>C	This study	pPT167	S1715R-mCherry	c.5143A>C	This study
pPT193	T1720A	c.5158A>G	This study	pPT203	T1720A-mCherry	c.5158A>G	This study
pPT148	G1738R	c.5212G>A	This study	pPT162	G1738R-mCherry	c.5212G>A	This study
pPT119	R1751Q	c.5252G>A	This study	pPT120	R1751Q-mCherry	c.5252G>A	This study
pPT194	L1764P	c.5291T>C	This study	pPT204	L1764P-mCherry	c.5291T>C	This study
pPT149	I1766S	c.5297T>G	This study	pPT163	I1766S-mCherry	c.5297T>G	This study
pPT150	M1775K	c.5324T>A	This study	pPT164	M1775K-mCherry	c.5324T>A	This study
pPT155	M1775R	c.5324T>G	This study	pPT169	M1775R-mCherry	c.5324T>G	This study
pPT179	P1776H	c.5327C>A	This study	pPT184	P1776H-mCherry	c.5327C>A	This study
pPT151	G1788V	c.5363G>T	This study	pPT165	G1788V-mCherry	c.5363G>T	This study
pPT122	V1804D	c.5411T>A	This study	pPT123	V1804D-mCherry	c.5411T>A	This study
pPT222	V1838E	c.5513T>A	This study	pPT226	V1838E-mCherry	c.5513T>A	This study
pPT223	I1858L	c.5572A>C	This study	pPT227	I1858L-mCherry	c.5572A>C	This study
pPT125	P1859R	c.5576C>G	This study	pPT127	P1859R-mCherry	c.5576C>G	This study

[a] Mutation and deletion nomenclature according to the human genome variation society (http://www.hgvs.org/mutnomen/).

[b] The BRCA1 cDNA used in Millot et.al, 2011, corresponds to the AY888184.1 GenBank sequence, slightly modified in this study (see the Methods section).

S11 Table. Yeast strains used

Strain designation	Strain common name [a]	Genotype	Source	Strain designation	Strain common name [a]	Genotype	Source
BY4741		*MAT*a, *leu2Δ0, his3Δ1, ura3Δ0, met15Δ0*	Winzeler et al., 1999	YKR082W-GFP		*MAT*a, *leu2Δ0, his3Δ1, ura3Δ0, met15Δ0, nup133::NUP133-GFP(HIS3MX6)*	Huh et al., 2003
ORT5039	Vector	BY4741 + pJL48	Millot et al., 2011				
GMT335	BRCA1	BY4741 + pPT60	This study	GMT336	BRCA1-mCherry	YKR082W-GFP + pPT63	This study
GMT424	M18T	BY4741 + pPT185	This study	GMT435	M18T-mCherry	YKR082W-GFP + pPT195	This study
GMT483	L22S	BY4741 + pPT175	This study	GMT502	L22S-mCherry	YKR082W-GFP + pPT180	This study
GMT377	I31M	BY4741 + pPT98	This study	GMT409	I31M-mCherry	YKR082W-GFP + pPT99	This study
GMT484	T37K	BY4741 + pPT176	This study	GMT503	T37K-mCherry	YKR082W-GFP + pPT181	This study
GMT485	C39R	BY4741 + pPT177	This study	GMT504	C39R-mCherry	YKR082W-GFP + pPT182	This study
GMT486	C39Y	BY4741 + pPT142	This study	GMT505	C39Y-mCherry	YKR082W-GFP + pPT156	This study
GMT378	C44F	BY4741 + pPT101	This study	GMT410	C44F-mCherry	YKR082W-GFP + pPT102	This study
GMT425	C44Y	BY4741 + pPT186	This study	GMT436	C44Y-mCherry	YKR082W-GFP + pPT196	This study
GMT379	K45Q	BY4741 + pPT104	This study	GMT411	K45Q-mCherry	YKR082W-GFP + pPT105	This study
GMT487	C47F	BY4741 + pPT143	This study	GMT506	C47F-mCherry	YKR082W-GFP + pPT157	This study
GMT380	C47G	BY4741 + pPT107	This study	GMT438	C47G-mCherry	YKR082W-GFP + pPT108	This study
GMT426	C61G	BY4741 + pPT187	This study	GMT437	C61G-mCherry	YKR082W-GFP + pPT197	This study
GMT433	C64Y	BY4741 + pPT110	This study	GMT446	C64Y-mCherry	YKR082W-GFP + pPT111	This study
GMT488	D67Y	BY4741 + pPT178	This study	GMT507	D67Y-mCherry	YKR082W-GFP + pPT183	This study
GMT381	N132K	BY4741 + pPT113	This study	GMT423	N132K-mCherry	YKR082W-GFP + pPT114	This study
GMT427	M1652I	BY4741 + pPT188	This study	GMT439	M1652I-mCherry	YKR082W-GFP + pPT198	This study
GMT428	M1652T	BY4741 + pPT189	This study	GMT440	M1652T-mCherry	YKR082W-GFP + pPT199	This study
GMT463	F1662S	BY4741 + pPT144	This study	GMT473	F1662S-mCherry	YKR082W-GFP + pPT158	This study
GMT464	A1669S	BY4741 + pPT145	This study	GMT474	A1669S-mCherry	YKR082W-GFP + pPT159	This study
GMT429	E1682K	BY4741 + pPT190	This study	GMT441	E1682K-mCherry	YKR082W-GFP + pPT200	This study
GMT430	T1685A	BY4741 + pPT191	This study	GMT442	T1685A-mCherry	YKR082W-GFP + pPT201	This study
GMT489	T1685I	BY4741 + pPT146	This study	GMT508	T1685I-mCherry	YKR082W-GFP + pPT160	This study
GMT490	M1689R	BY4741 + pPT152	This study	GMT509	M1689R-mCherry	YKR082W-GFP + pPT166	This study
GMT382	R1699W	BY4741 + pPT116	This study	GMT413	R1699W-mCherry	YKR082W-GFP + pPT117	This study
GMT491	G1706E	BY4741 + pPT147	This study	GMT510	G1706E-mCherry	YKR082W-GFP + pPT161	This study
GMT431	A1708E	BY4741 + pPT192	This study	GMT443	A1708E-mCherry	YKR082W-GFP + pPT202	This study
GMT472	S1715R	BY4741 + pPT153	This study	GMT482	S1715R-mCherry	YKR082W-GFP + pPT167	This study
GMT432	T1720A	BY4741 + pPT193	This study	GMT444	T1720A-mCherry	YKR082W-GFP + pPT203	This study
GMT471	G1738R	BY4741 + pPT148	This study	GMT481	G1738R-mCherry	YKR082W-GFP + pPT162	This study
GMT383	R1751Q	BY4741 + pPT119	This study	GMT414	R1751Q-mCherry	YKR082W-GFP + pPT120	This study
GMT434	L1764P	BY4741 + pPT194	This study	GMT445	L1764P-mCherry	YKR082W-GFP + pPT204	This study
GMT466	I1766S	BY4741 + pPT149	This study	GMT476	I1766S-mCherry	YKR082W-GFP + pPT163	This study
GMT468	M1775K	BY4741 + pPT150	This study	GMT478	M1775K-mCherry	YKR082W-GFP + pPT164	This study
GMT467	M1775R	BY4741 + pPT155	This study	GMT477	M1775R-mCherry	YKR082W-GFP + pPT169	This study
GMT469	P1776H	BY4741 + pPT179	This study	GMT479	P1776H-mCherry	YKR082W-GFP + pPT184	This study
GMT470	G1788V	BY4741 + pPT151	This study	GMT480	G1788V-mCherry	YKR082W-GFP + pPT165	This study
GMT384	V1804D	BY4741 + pPT122	This study	GMT415	V1804D-mCherry	YKR082W-GFP + pPT123	This study
GMT493	V1838E	BY4741 + pPT222	This study	GMT498	V1838E-mCherry	YKR082W-GFP + pPT226	This study
GMT515	I1858L	BY4741 + pPT223	This study	GMT499	I1858L-mCherry	YKR082W-GFP + pPT227	This study
GMT385	P1859R	BY4741 + pPT125	This study	GMT416	P1859R-mCherry	YKR082W-GFP + pPT127	This study

[a] "mCherry" was omitted in the main text. For instance, the P1859R-mCherry strain was referred to as "P1859R".

S12 Table. Average CDF of the probability system of classification when using the standard method

Rank of the best cut-off values	Colony Size assay		Liquid Medium assay		Spot Formation assay		Yeast Localization assay	
	Best cut-off value (n = 529)	Probability of pathogenicity	Best cut-off value (n = 126)	Probability of pathogenicity	Best cut-off value (n = 190)	Probability of pathogenicity	Best cut-off value (n = 154)	Probability of pathogenicity
1	11853.3333	0.00025	0.149500	0.00025	0.31943533	0.00025	0.00818150	0.00025
2	11930.0000	0.00075	0.150500	0.00075	0.31609788	0.00075	0.01210510	0.00075
3	12132.9060	0.00150	0.150750	0.00125	0.31536359	0.00150	0.01509030	0.00125
4	12150.0000	0.00225	0.151000	0.00200	0.31499829	0.00300	0.01880518	0.00175
5	12200.0000	0.00275	0.151250	0.00275	0.31468908	0.00775	0.01904816	0.00225
6	12216.0685	0.00325	0.151500	0.00325	0.31243640	0.01300	0.01944921	0.00300
7	12230.0000	0.00400	0.151750	0.00375	0.31232318	0.01475	0.01955043	0.00425
8	12253.2478	0.00475	0.152000	0.00425	0.31212019	0.01550	0.01966000	0.00550
9	12276.4955	0.00675	0.152500	0.00675	0.31203042	0.01825	0.02580891	0.00625
10	12454.1285	0.00875	0.153000	0.01050	0.31196558	0.02075	0.02785367	0.00700
11	12595.5815	0.00925	0.153500	0.01375	0.31190075	0.02175	0.02819869	0.00775
12	12632.9060	0.01000	0.153750	0.01575	0.30964807	0.02300	0.02861547	0.00825
13	12650.0000	0.01075	0.154000	0.01700	0.30924209	0.02450	0.02975333	0.00875
14	12675.0000	0.01125	0.154500	0.02000	0.30840599	0.02575	0.03074321	0.00925
15	12700.0000	0.01175	0.155000	0.02275	0.30756988	0.02750	0.03399520	0.00975
16	12730.0000	0.01275	0.155250	0.02375	0.30749347	0.02925	0.03411158	0.01175
17	12753.2478	0.01400	0.155500	0.02550	0.30669004	0.03150	0.03412337	0.01425
18	12776.4955	0.01575	0.156000	0.02725	0.30497331	0.03375	0.03413517	0.01900
19	12825.0000	0.01725	0.156500	0.02800	0.30460801	0.03475	0.03454015	0.02350
20	12860.0428	0.01775	0.156750	0.02925	0.30429879	0.03650	0.03494513	0.02425
21	12920.0855	0.01925	0.157250	0.03025	0.30387171	0.03775	0.03495693	0.02475
22	12944.6758	0.02075	0.157500	0.03275	0.30326198	0.03825	0.03496873	0.02650
23	12969.2660	0.02475	0.157750	0.03525	0.30307933	0.03875	0.03590079	0.02850
24	13016.4705	0.02875	0.158000	0.03825	0.30292472	0.03925	0.03592438	0.03025
25	13063.6750	0.03200	0.158500	0.05500	0.30277011	0.04025	0.03788025	0.03175
26	13104.4790	0.03525	0.158750	0.06925	0.30204612	0.04175	0.04597766	0.03225
27	13145.2830	0.03925	0.159000	0.07225	0.30164013	0.04350	0.04609403	0.03275
28	13160.0268	0.04325	0.159500	0.10725	0.30051743	0.04500	0.04610583	0.03325
29	13172.6415	0.04375	0.159750	0.13975	0.30011145	0.04650	0.04611762	0.03425
30	13187.3853	0.04450	0.160000	0.16775	0.29996793	0.04900	0.04929481	0.03625
31	13200.0000	0.05225	0.160500	0.21125	0.29908809	0.05300	0.04945828	0.03775
32	13225.0000	0.05975	0.160750	0.22775	0.29845997	0.05575	0.05312493	0.03850
33	13255.1280	0.06750	0.161000	0.24475	0.29843925	0.05750	0.05361440	0.04575
34	13292.4115	0.07525	0.161500	0.28975	0.29817148	0.05975	0.05489660	0.05275
35	13544.1133	0.07575	0.161750	0.31875	0.29786544	0.06075	0.05617881	0.05700
36	13641.4648	0.07625	0.162000	0.32550	0.29755940	0.06275	0.05656693	0.06200
37	13666.0550	0.07675	0.162375	0.33225	0.29728122	0.06550	0.05695506	0.06475
38	13760.4640	0.07725	0.162500	0.36150	0.29700304	0.06875	0.05713629	0.06675
39	13842.0720	0.07775	0.162625	0.39100	0.29648933	0.09300	0.05731751	0.09300
40	13884.1743	0.07825	0.163000	0.40375	0.29620407	0.11550	0.05823459	0.11975
41	13896.7890	0.07875	0.163250	0.41675	0.29571581	0.11625	0.05884794	0.12075
42	13924.3530	0.07925	0.163500	0.42050	0.29569658	0.11675	0.05933556	0.12125
43	13951.9170	0.07975	0.163625	0.42375	0.29549359	0.11725	0.05951947	0.12200
44	13982.6863	0.08025	0.164000	0.43875	0.29547435	0.11950	0.06008882	0.12275
45	14013.4555	0.08125	0.164125	0.45375	0.29496065	0.14200	0.06065817	0.13275
46	14066.7890	0.08250	0.164250	0.45450	0.29444010	0.16300	0.06175647	0.14275
47	14120.1225	0.08325	0.164375	0.45550	0.29391954	0.17650	0.06285477	0.14375
48	14140.0613	0.08375	0.164500	0.46050	0.29388008	0.19000	0.06296903	0.14475
49	14196.7890	0.08425	0.164750	0.46525	0.29340069	0.19100	0.06362439	0.14550
50	14268.2663	0.08475	0.164875	0.46600	0.29296077	0.19350	0.06439401	0.15425
51	14286.9600	0.08550	0.165000	0.46825	0.29267582	0.19575	0.06485439	0.16275
52	14290.1468	0.08625	0.165125	0.47025	0.29239086	0.20625	0.06549231	0.16400
53	14297.6085	0.08675	0.165250	0.47075	0.29163329	0.21700	0.06792915	0.16525
54	14334.8230	0.08725	0.165375	0.47125	0.29087572	0.21850	0.07283799	0.16600
55	14346.7890	0.08850	0.165500	0.47300	0.29061887	0.21975	0.07578785	0.16725
56	14364.8475	0.08975	0.165750	0.47475	0.29036202	0.23200	0.08313352	0.16875
57	14382.9060	0.09025	0.165875	0.47525	0.28933620	0.24425	0.08649566	0.16975
58	14393.1620	0.09075	0.166000	0.47600	0.28831037	0.26450	0.08685300	0.17025
59	14431.6668	0.09125	0.166250	0.47675	0.28805130	0.28575	0.08872092	0.17125
60	14450.0000	0.09175	0.166500	0.48150	0.28779223	0.29300	0.09054727	0.17250
61	14465.0000	0.09225	0.166625	0.48625	0.28371174	0.31000	0.09098380	0.17325
62	14481.7095	0.09275	0.167000	0.48875	0.28354216	0.32125	0.09139695	0.17375
63	14521.3675	0.09325	0.167250	0.49150	0.28337259	0.32175	0.09254472	0.17525
64	14526.4955	0.09450	0.167500	0.50475	0.28311574	0.32250	0.09271581	0.17800
65	14532.5995	0.09575	0.167750	0.51800	0.28285889	0.32525	0.09382692	0.18000
66	14560.0428	0.09625	0.168000	0.52275	0.28243343	0.32825	0.09412014	0.18075
67	14585.9325	0.09700	0.168125	0.52725	0.28200797	0.34350	0.09523126	0.18125
68	14592.9663	0.09775	0.168250	0.52800	0.28129329	0.35825	0.09552447	0.19975
69	14593.0275	0.09825	0.168500	0.54275	0.28057862	0.35875	0.09580061	0.21975
70	14600.1225	0.09875	0.168750	0.55725	0.28043386	0.35925	0.09658185	0.22175

71	14625.7493	0.09925	0.169000	0.56650	0.28028910	0.36025	0.09685799	0.22325
72	14641.7095	0.09975	0.169125	0.57575	0.28006492	0.36350	0.09763923	0.23700
73	14670.0855	0.10075	0.169500	0.58775	0.27923886	0.36625	0.09768395	0.24975
74	14675.2135	0.10175	0.169750	0.60000	0.27898201	0.36750	0.09872591	0.25025
75	14680.3415	0.10225	0.170000	0.60350	0.27823857	0.36900	0.09981258	0.25100
76	14708.6165	0.10275	0.170250	0.60675	0.27749513	0.37000	0.10062218	0.25200
77	14713.9413	0.10325	0.170500	0.61350	0.27695367	0.37150	0.10181003	0.25350
78	14719.2660	0.10500	0.170625	0.62025	0.27641222	0.37275	0.10380747	0.25650
79	14726.6668	0.10675	0.170750	0.62125	0.27631041	0.37325	0.10448754	0.25900
80	14761.9495	0.10825	0.171000	0.63175	0.27620861	0.37600	0.10521180	0.26050
81	14762.6415	0.10975	0.171125	0.64175	0.27599589	0.37875	0.10589188	0.26200
82	14763.3335	0.11075	0.171250	0.64225	0.27578317	0.37925	0.10661614	0.28700
83	14813.6750	0.11375	0.171375	0.64275	0.27538497	0.37975	0.10745969	0.31175
84	14815.1708	0.11650	0.171500	0.64650	0.27526947	0.38125	0.10873090	0.32625
85	14816.6665	0.11850	0.171750	0.65075	0.27524893	0.38275	0.10894670	0.34075
86	14835.5305	0.12075	0.172000	0.67150	0.27498677	0.38350	0.11035103	0.34150
87	14862.1970	0.12175	0.172250	0.69250	0.27474580	0.38450	0.11056684	0.34225
88	14871.7945	0.12375	0.172500	0.72775	0.27450483	0.39300	0.11197117	0.34750
89	14875.8973	0.12575	0.172750	0.76225	0.27395974	0.40275	0.11207404	0.35400
90	14880.0000	0.12625	0.173000	0.78300	0.27360226	0.40500	0.11217690	0.36950
91	14887.6415	0.12700	0.173250	0.80375	0.27305716	0.40575	0.11291090	0.38400
92	14895.2830	0.12925	0.173500	0.81100	0.27291581	0.40650	0.11364490	0.41075
93	14914.3080	0.13150	0.173750	0.81850	0.27287319	0.40800	0.11386541	0.43725
94	14933.3330	0.13350	0.174000	0.82675	0.27220528	0.40950	0.11397992	0.43775
95	14940.1708	0.13525	0.174250	0.83500	0.27207789	0.41025	0.11408593	0.44125
96	14944.9573	0.13575	0.174500	0.85925	0.27206392	0.41100	0.11441089	0.44475
97	14948.5043	0.13625	0.174750	0.88375	0.27202130	0.41250	0.11449111	0.44525
98	14950.0000	0.14050	0.175000	0.89650	0.27171087	0.41725	0.11824779	0.44575
99	14966.4530	0.14475	0.175250	0.90975	0.27132135	0.42150	0.12255691	0.44625
100	14993.3335	0.14525	0.175500	0.92025	0.27116941	0.42375	0.12326857	0.46550
101	15002.5640	0.14575	0.176000	0.94250	0.27099445	0.42575	0.12400257	0.48550
102	15005.1280	0.14950	0.176250	0.95625	0.27062795	0.42775	0.12468658	0.48675
103	15016.8723	0.15400	0.176500	0.96050	0.27059229	0.42975	0.12473657	0.51500
104	15028.6165	0.15575	0.177000	0.96500	0.26962357	0.43050	0.12668009	0.54325
105	15034.3083	0.15725	0.177250	0.96625	0.26959030	0.43125	0.12701921	0.54375
106	15049.0520	0.15825	0.177500	0.96900	0.26924716	0.43175	0.12862360	0.54925
107	15053.3333	0.16025	0.177750	0.97175	0.26861919	0.43275	0.12935760	0.55550
108	15058.3333	0.16225	0.178000	0.97350	0.26847784	0.43375	0.13009160	0.56100
109	15065.0000	0.16275	0.178250	0.97525	0.26782189	0.43425	0.13687872	0.56575
110	15075.0000	0.16425	0.178500	0.97600	0.26781239	0.43475	0.13694975	0.56650
111	15081.6668	0.16575	0.179000	0.97700	0.26767104	0.43550	0.14507017	0.56775
112	15083.3335	0.16725	0.179500	0.97775	0.26759604	0.43625	0.14514120	0.56925
113	15084.9145	0.16900	0.180000	0.97900	0.26700559	0.45450	0.14596459	0.57025
114	15121.5810	0.17050	0.180250	0.98025	0.26696050	0.47300	0.14647450	0.57225
115	15132.4785	0.17175	0.180500	0.98075	0.26691542	0.48125	0.14700911	0.57425
116	15135.8975	0.17225	0.180750	0.98125	0.26666416	0.48925	0.14753188	0.57600
117	15138.4615	0.17350	0.181500	0.98525	0.26663271	0.49050	0.14806649	0.57775
118	15145.9830	0.17475	0.182000	0.99050	0.26624197	0.49250	0.14858926	0.57875
119	15155.8975	0.17625	0.182500	0.99375	0.26585123	0.51775	0.14887353	0.57975
120	15163.4190	0.17775	0.183000	0.99625	0.26500313	0.54250	0.15146827	0.58125
121	15173.3335	0.17975	0.183500	0.99725	0.26477402	0.54325	0.15175254	0.58275
122	15186.6668	0.18250	0.183750	0.99775	0.26476247	0.54375	0.15294295	0.58375
123	15190.0855	0.18425	0.184000	0.99825	0.26462652	0.54425	0.15362046	0.58475
124	15203.4188	0.18650	0.187500	0.99875	0.26421162	0.55550	0.15434728	0.59025
125	15210.0855	0.18875	0.188500	0.99925	0.26367016	0.56725	0.15540466	0.59600
126	15218.4615	0.18975	0.189000	0.99975	0.26363054	0.56825	0.15646204	0.59975
127	15228.4188	0.19075			0.26360978	0.56875	0.15667528	0.60350
128	15230.7690	0.19175			0.26334458	0.56925	0.15807961	0.60525
129	15234.0170	0.19225			0.26312871	0.57625	0.15969718	0.60800
130	15240.0000	0.19300			0.26309299	0.58375	0.16075456	0.61175
131	15248.3760	0.19375			0.26305727	0.60175	0.16181194	0.61425
132	15258.3333	0.19450			0.26251581	0.62050	0.16246944	0.61600
133	15260.6835	0.19550			0.26238261	0.62225	0.16312693	0.61875
134	15266.6665	0.19700			0.26211771	0.62275	0.16386093	0.62025
135	15272.9663	0.19825			0.26197435	0.63125	0.16459493	0.62450
136	15279.2660	0.19900			0.26069526	0.64025	0.16779732	0.62975
137	15306.6670	0.20050			0.26035916	0.64125	0.17099971	0.63525
138	15323.4190	0.20200			0.25981457	0.64175	0.17173371	0.64000
139	15329.7185	0.20325			0.25941617	0.65225	0.17246771	0.64825
140	15340.1710	0.20650			0.25927482	0.66550	0.17440866	0.65650
141	15356.9230	0.20925			0.25919376	0.66875	0.17634962	0.65975
142	15373.6750	0.20975			0.25869764	0.67125	0.17708362	0.66300
143	15380.8545	0.21025			0.25826182	0.69025	0.17781761	0.66650
144	15388.0340	0.21075			0.25797911	0.71050	0.18102183	0.67225
145	15394.0170	0.21250			0.25728536	0.71450	0.18422605	0.69100
146	15400.0000	0.21700			0.25635407	0.71525	0.19209883	0.73825
147	15406.8375	0.22025			0.25587461	0.71575	0.19477378	0.77025
148	15427.6415	0.22175			0.25543726	0.71625	0.19744873	0.78675
149	15434.4790	0.22325			0.25460612	0.71700	0.19969382	0.80350

150	15455.2830	0.22375	0.24996419	0.71775	0.20193891	0.82850
151	15464.3938	0.22575	0.24971023	0.71850	0.20587530	0.85425
152	15473.5045	0.22775	0.24913305	0.71925	0.20981169	0.90225
153	15484.3938	0.22900	0.24449111	0.72575	0.21248665	0.94975
154	15491.7523	0.23025	0.24390135	0.73225	0.21516160	0.97550
155	15502.6415	0.23075	0.24331159	0.74475		
156	15510.0000	0.23450	0.24307022	0.75725		
157	15515.0000	0.23825	0.24282884	0.76650		
158	15520.0000	0.23900	0.24198823	0.77775		
159	15520.6838	0.23975	0.24181878	0.78050		
160	15521.3675	0.24025	0.24164932	0.79275		
161	15527.3505	0.24075	0.24122902	0.80550		
162	15529.0520	0.24125	0.24080871	0.80900		
163	15533.3335	0.24250	0.23871076	0.81175		
164	15541.6668	0.24400	0.23661281	0.81625		
165	15550.0000	0.24550	0.23555322	0.82150		
166	15565.1280	0.24825	0.23549325	0.82325		
167	15574.7008	0.25025	0.23543329	0.82750		
168	15605.1280	0.25100	0.23490349	0.83150		
169	15615.8973	0.25175	0.23437369	0.83425		
170	15626.6665	0.25300	0.23291535	0.83700		
171	15632.1365	0.25425	0.23145701	0.83850		
172	15637.6065	0.25500	0.23086724	0.84025		
173	15645.4700	0.25575	0.23027748	0.84175		
174	15653.3335	0.25800	0.22968495	0.84400		
175	15666.6665	0.26050	0.22909241	0.87275		
176	15709.5953	0.26125	0.22803282	0.91350		
177	15733.3335	0.26375	0.22760370	0.92750		
178	15752.1368	0.26650	0.22717458	0.94300		
179	15770.9400	0.26825	0.22611498	0.96550		
180	15772.1368	0.26975	0.22393660	0.97975		
181	15773.3335	0.27075	0.22380458	0.98675		
182	15786.6670	0.27250	0.22367255	0.98725		
183	15810.0000	0.27375	0.22274630	0.98825		
184	15875.0855	0.27425	0.22201877	0.99300		
185	15900.1710	0.27575	0.22175472	0.99725		
186	15902.2223	0.27725	0.22129159	0.99775		
187	15920.1710	0.27850	0.22082846	0.99825		
188	15926.1538	0.28025	0.21805242	0.99875		
189	15944.1025	0.28125	0.21777202	0.99925		
190	15946.1540	0.28175	0.21613458	0.99975		
191	15950.0855	0.28225				
192	15954.0170	0.28300				
193	15960.0000	0.28575				
194	15994.0170	0.28825				
195	16000.0000	0.28900				
...	...	...				
468	24326.6665	0.94000				
469	24401.6665	0.94175				
470	24428.3180	0.94225				
471	24594.7858	0.94300				
472	24621.4373	0.94375				
473	24712.9050	0.94575				
474	24719.7858	0.94800				
475	24726.6665	0.94925				
476	24744.9845	0.95025				
477	24796.6365	0.95150				
478	24798.3183	0.95300				
479	24800.0000	0.95425				
480	24890.8255	0.95550				
481	24920.4128	0.95625				
482	24950.0000	0.95675				
483	24975.2295	0.95800				
484	25073.3335	0.95950				
485	25186.2385	0.96050				
486	25193.1193	0.96150				
487	25238.5320	0.96225				
488	25277.0640	0.96275				
489	25283.9448	0.96325				
490	25314.9083	0.96375				
491	25459.5720	0.96425				
492	25466.4528	0.96475				
493	25489.9695	0.96525				
494	25494.8318	0.96575				
495	25499.6940	0.96650				
496	25756.5750	0.96825				
497	25764.2200	0.96975				
498	25765.2905	0.97025				
499	25766.3610	0.97200				
500	25829.9695	0.97375				
501	25893.5780	0.97475				
502	25919.2660	0.97575				
503	25944.9540	0.97800				
504	25963.3030	0.98025				
505	25993.2725	0.98075				

506	26023.2420	0.98175
507	26038.6853	0.98275
508	26054.1285	0.98325
509	26150.4585	0.98375
510	26150.4590	0.98450
511	26176.1470	0.98525
512	26201.8350	0.98750
513	26219.2358	0.98975
514	26459.6940	0.99075
515	26574.3425	0.99175
516	26688.9910	0.99225
517	26716.5750	0.99325
518	26933.0275	0.99425
519	26978.4403	0.99475
520	27023.8530	0.99525
521	27139.4498	0.99575
522	27189.9085	0.99625
523	27198.1348	0.99675
524	27206.3610	0.99725
525	27280.7340	0.99775
526	27371.9880	0.99825
527	27463.2420	0.99875
528	28058.7158	0.99925
529	29286.5753	0.99975

See S6C Fig for details. The 272 middle values of the average CDF, derived from the Colony Size assay, were removed to simplify the table. Colored numbers indicate the 5-class nomenclature (S1 Table): dark blue, class 1; light blue, class 2; pink, class4; red, class 5. This table was used to attribute the probabilities of pathogenicity depicted in Fig 2B. For instance, in the Colony Size assay, the median of P1776H is 12,253 cells per colony. The closest best cut-off value of this median belongs to rank 8, meaning that, when using the standard method, the probability of pathogenicity attributed to P1776H is 0.00475. Of note, if a best cut-off value is not repeated, among the 2,000 best cut-off values used to derive the CDF, then the associated probability is 1 / 2,000 = 0.0005 for this value. This indicates the minimal probability incrementation between two non repeated consecutive best cut-off values in the CDF (probability unit). In the Yeast Localization assay, no variant can be classified as class 5 due to a lack of unrepeated values at the "pathogenic" side of the best cut-off distribution (the highest cut-off value, 0.2151616, is present 97 times, leading to a probability of 0.0485 and an average cumulative probability of 0.97550 in the CDF, which is inferior to the 0.99 probability threshold of the class 5).

S13 Table. Basic and corrected probability of pathogenicity when using the standard method

Variant	Colony Size assay				Liquid Medium Assay				Spot Formation assay				Yeast Localization assay				Combined odds (CS x SF)	Combined probability (CS x SF)	Corrected odds combined (CS x SF)	Corrected probability combined (CS x SF)
	Probability	Odds	Corrected odds	Corrected probability	Probability	Odds	Corrected odds	Corrected probability	Probability	Odds	Corrected odds	Corrected probability	Probability	Odds	Corrected odds	Corrected probability				
M1689R	0.99975	3 999	3 809	0.99974	0.99975	3 999	3 809	0.99974	0.99975	3 999	3 809	0.99974	0.9755	40	38	0.97434	15 992 001	1	14 505 579	1
G1706E	0.99975	3 999	3 809	0.99974	0.99975	3 999	3 809	0.99974	0.99975	3 999	3 809	0.99974	0.9755	40	38	0.97434	15 992 001	1	14 505 579	1
A1708E	0.99975	3 999	3 809	0.99974	0.99975	3 999	3 809	0.99974	0.99975	3 999	3 809	0.99974	0.9755	40	38	0.97434	15 992 001	1	14 505 579	1
S1715R	0.99975	3 999	3 809	0.99974	0.99975	3 999	3 809	0.99974	0.99975	3 999	3 809	0.99974	0.9755	40	38	0.97434	15 992 001	1	14 505 579	1
L1764P	0.99975	3 999	3 809	0.99974	0.99975	3 999	3 809	0.99974	0.99975	3 999	3 809	0.99974	0.9755	40	38	0.97434	15 992 001	1	14 505 579	1
I1766S	0.99975	3 999	3 809	0.99974	0.99975	3 999	3 809	0.99974	0.99975	3 999	3 809	0.99974	0.9755	40	38	0.97434	15 992 001	1	14 505 579	1
G1788V	0.99975	3 999	3 809	0.99974	0.99975	3 999	3 809	0.99974	0.99975	3 999	3 809	0.99974	0.9755	40	38	0.97434	15 992 001	1	14 505 579	1
V1838E	0.99975	3 999	3 809	0.99974	0.99975	3 999	3 809	0.99974	0.99975	3 999	3 809	0.99974	0.9755	40	38	0.97434	15 992 001	1	14 505 579	1
G1738R	0.99975	3 999	3 809	0.99974	0.99975	3 999	3 809	0.99974	0.99975	3 999	3 809	0.99974	0.9755	40	38	0.97434	15 992 001	1	14 505 579	1
T1685I	0.99975	3 999	3 809	0.99974	0.99975	3 999	3 809	0.99974	0.99975	3 999	3 809	0.99974	0.9755	40	38	0.97434	15 992 001	1	14 505 579	1
T1685A	0.99975	3 999	3 809	0.99974	0.99975	3 999	3 809	0.99974	0.99975	3 999	3 809	0.99974	0.9755	40	38	0.97434	15 992 001	1	14 505 579	1
C44Y	0.99975	3 999	3 809	0.99974	0.99975	3 999	3 809	0.99974	0.99975	3 999	3 809	0.99974	0.9755	40	38	0.97434	15 992 001	1	14 505 579	1
C44F	0.99975	3 999	3 809	0.99974	0.99975	3 999	3 809	0.99974	0.99975	3 999	3 809	0.99974	0.9755	40	38	0.97434	15 992 001	1	14 505 579	1
C64Y	0.99975	3 999	3 809	0.99974	0.9905	104	99	0.99003	0.99975	3 999	3 809	0.99974	0.9755	40	38	0.97434	15 992 001	1	14 505 579	1
T37K	0.99925	1 332	1 269	0.99921	0.99975	3 999	3 809	0.99974	0.71925	2.56	2.49	0.71326	0.9755	40	38	0.97434	3 413	0.99971	3 156	0.99968
C39R	0.99975	3 999	3 809	0.99974	0.82675	4.77	4.59	0.82119	0.99975	3 999	3 809	0.99974	0.9755	40	38	0.97434	15 992 001	1	14 505 579	1
C61G	0.99525	210	200	0.99501	0.783	3.61	3.48	0.77699	0.97975	48	46	0.97878	0.56575	1.30	1.29	0.56301	10 137	0.99990	9 207	0.99989
C47G	0.99975	3 999	3 809	0.99974	0.99875	799	761	0.99869	0.943	16.54	15.80	0.94049	0.164	0.2	0.2	0.16942	66 159	0.99998	60 190	0.99998
M18T	0.99975	3 999	3 809	0.99974	0.99825	570	543	0.99816	0.81175	4.31	4.15	0.80599	0.12075	0.14	0.14	0.12527	17 244	0.99994	15 822	0.99994
C39Y	0.99975	3 999	3 809	0.99974	0.99975	3 999	3 809	0.99974	0.71925	2.56	2.49	0.71326	0.0055	0.0055	0.0058	0.00577	10 245	0.99990	9 474	0.99989
M1775R	0.99975	3 999	3 809	0.99974	0.965	28	26	0.96338	0.293	0.41	0.43	0.29889	0.00125	0.0013	0.0013	0.00131	1 657	0.99940	1 624	0.99938
L22S	0.99975	3 999	3 809	0.99974	0.99975	3 999	3 809	0.99974	0.00025	0.00025	0.00026	0.00026	0.00025	0.00025	0.00026	0.00026	1	0.50000	1	0.50000
M1775K	0.99975	3 999	3 809	0.99974	0.6035	1.52	1.50	0.59955	0.00025	0.00025	0.00026	0.00026	0.00025	0.00025	0.00026	0.00026	1	0.50000	1	0.50000
C47F	0.47	0.89	0.89	0.47135	0.24475	0.32	0.33	0.25085	0.00025	0.00025	0.00026	0.00026	0.00075	0.00075	0.00079	0.00079	0.00022	0.00022	0.00023	0.00023
R1699W	0.07525	0.081	0.085	0.07842	0.017	0.017	0.018	0.01782	0.00025	0.00025	0.00026	0.00026	0.00125	0.0013	0.0013	0.00131	2.03E-5	2.03E-5	2.23E-5	2.23E-5
T1720A	0.00025	0.00025	0.00026	0.00026	0.00025	0.00025	0.00026	0.00026	0.00025	0.00025	0.00026	0.00026	0.00025	0.00025	0.00026	0.00026	6.25E-8	6.25E-8	6.89E-8	6.89E-8
P1859R	0.00025	0.00025	0.00026	0.00026	0.00025	0.00025	0.00026	0.00026	0.00025	0.00025	0.00026	0.00026	0.00025	0.00025	0.00026	0.00026	6.25E-8	6.25E-8	6.89E-8	6.89E-8
R1751Q	0.00025	0.00025	0.00026	0.00026	0.00025	0.00025	0.00026	0.00026	0.00025	0.00025	0.00026	0.00026	0.00175	0.0018	0.0018	0.00184	6.25E-8	6.25E-8	6.89E-8	6.89E-8
E1682K	0.00025	0.00025	0.00026	0.00026	0.00025	0.00025	0.00026	0.00026	0.00025	0.00025	0.00026	0.00026	0.00025	0.00025	0.00026	0.00026	6.25E-8	6.25E-8	6.89E-8	6.89E-8
M1652I	0.00025	0.00025	0.00026	0.00026	0.00025	0.00025	0.00026	0.00026	0.00025	0.00025	0.00026	0.00026	0.00425	0.0043	0.0045	0.00446	6.25E-8	6.25E-8	6.89E-8	6.89E-8
I31M	0.00025	0.00025	0.00026	0.00026	0.00025	0.00025	0.00026	0.00026	0.00025	0.00025	0.00026	0.00026	0.00175	0.0018	0.0018	0.00184	6.25E-8	6.25E-8	6.89E-8	6.89E-8
I1858L	0.00025	0.00025	0.00026	0.00026	0.00425	0.0043	0.0045	0.00446	0.00025	0.00025	0.00026	0.00026	0.00025	0.00025	0.00026	0.00026	6.25E-8	6.25E-8	6.89E-8	6.89E-8
P1776H	0.00475	0.0048	0.005	0.00499	0.00025	0.00025	0.00026	0.00026	0.00025	0.00025	0.00026	0.00026	0.0055	0.0055	0.0058	0.00577	1.19E-6	1.19E-6	1.32E-6	1.32E-6
F1662S	0.00075	0.00075	0.00079	0.00079	0.00025	0.00025	0.00026	0.00026	0.00025	0.00025	0.00026	0.00026	0.00975	0.0098	0.01	0.01023	1.88E-7	1.88E-7	2.07E-7	2.07E-7
V1804D	0.00025	0.00025	0.00026	0.00026	0.03825	0.04	0.042	0.04001	0.00025	0.00025	0.00026	0.00026	0.00025	0.00025	0.00026	0.00026	6.25E-8	6.25E-8	6.89E-8	6.89E-8
A1669S	0.00025	0.00025	0.00026	0.00026	0.03825	0.04	0.042	0.04001	0.04175	0.044	0.046	0.04366	0.00025	0.00025	0.00026	0.00026	1.09E-5	1.09E-5	1.20E-5	1.20E-5
N132K	0.00025	0.00025	0.00026	0.00026	0.00425	0.0043	0.0045	0.00446	0.00025	0.00025	0.00026	0.00026	0.057	0.06	0.063	0.05951	6.25E-8	6.25E-8	6.89E-8	6.89E-8
D67Y	0.00025	0.00025	0.00026	0.00026	0.24475	0.32	0.33	0.25085	0.00025	0.00025	0.00026	0.00026	0.00025	0.00025	0.00026	0.00026	6.25E-8	6.25E-8	6.89E-8	6.89E-8
M1652T	0.99975	3 999	3 809	0.99974	0.99975	3 999	3 809	0.99974	0.00025	0.00025	0.00026	0.00026	0.00975	0.0098	0.01	0.01023	1	0.50000	1	0.50000
K45Q	0.47	0.89	0.89	0.47135	0.6715	2.04	1.99	0.66605	0.8385	5.19	4.99	0.83312	0.16725	0.2	0.21	0.17272	4.60	0.82156	4.45	0.81655
Sensitivity	0.92 (23/25)			0.92 (23/25)	0.80 (20/25)			0.80 (20/25)	0.64 (16/25)			0.64 (16/25)	0.64 (16/25)			0.64 (16/25)		0.84 (21/25)		0.84 (21/25)
Specificity	0.87 (13/15)			0.87 (13/15)	0.80 (12/15)			0.80 (12/15)	0.93 (14/15)			0.93 (14/15)	0.87 (13/15)			0.87 (13/15)		0.87 (13/15)		0.87 (13/15)
Accuracy	0.90 (36/40)			0.90 (36/40)	0.80 (32/40)			0.80 (32/40)	0.75 (30/40)			0.75 (30/40)	0.73 (29/40)			0.73 (29/40)		0.85 (34/40)		0.85 (34/40)

See Fig 2B for details. Values in the "Probability" columns are those in Fig 2B. Mutations are ordered as in Fig 2B to facilitate comparisons. Odds in favor of pathogenicity are the ratio pi / (1 - pi), with pi being the probability of pathogenicity of the variant i. The Liquid Medium and Yeast Localization assays were not included in the combined odds, since the Liquid Medium and Colony Size, as well as the Spot Formation and Yeast Localization, are not independent assays. Combined probabilities of pathogenicity result from the ratio Oi / (1 + Oi), with Oi being the combined odds of the variant i. Probabilities were also corrected according to $n_{neutral} + n_{pathogenic} = 40$ and a = 2 (see the S1 Text). CS, Colony Size assay; SF, Spot Formation assay.

S14 Table. Basic and corrected probability of pathogenicity when using the standard with reference method

Variant	Colony Size assay				Liquid Medium Assay				Spot Formation assay				Yeast Localization assay				Combined odds (CS x SF)	Combined probability (CS x SF)	Corrected odds combined (CS x SF)	Corrected probability combined (CS x SF)
	Probability	Odds	Corrected odds	Corrected probability	Probability	Odds	Corrected odds	Corrected probability	Probability	Odds	Corrected odds	Corrected probability	Probability	Odds	Corrected odds	Corrected probability				
M1689R	0.99975	3 999	3 809	0.99974	0.99975	3 999	3 809	0.99974	0.99975	3 999	3 809	0.99974	0.99975	3 999	3 809	0.99974	15 992 001	1	14 505 579	1
G1706E	0.99975	3 999	3 809	0.99974	0.99975	3 999	3 809	0.99974	0.99975	3 999	3 809	0.99974	0.99975	3 999	3 809	0.99974	15 992 001	1	14 505 579	1
A1708E	0.99975	3 999	3 809	0.99974	0.99975	3 999	3 809	0.99974	0.99975	3 999	3 809	0.99974	0.99975	3 999	3 809	0.99974	15 992 001	1	14 505 579	1
S1715R	0.99975	3 999	3 809	0.99974	0.99975	3 999	3 809	0.99974	0.99975	3 999	3 809	0.99974	0.99975	3 999	3 809	0.99974	15 992 001	1	14 505 579	1
L1764P	0.99975	3 999	3 809	0.99974	0.99975	3 999	3 809	0.99974	0.99975	3 999	3 809	0.99974	0.99975	3 999	3 809	0.99974	15 992 001	1	14 505 579	1
I1766S	0.99975	3 999	3 809	0.99974	0.99975	3 999	3 809	0.99974	0.99975	3 999	3 809	0.99974	0.99975	3 999	3 809	0.99974	15 992 001	1	14 505 579	1
G1788V	0.99975	3 999	3 809	0.99974	0.99975	3 999	3 809	0.99974	0.99975	3 999	3 809	0.99974	0.99975	3 999	3 809	0.99974	15 992 001	1	14 505 579	1
V1838E	0.99975	3 999	3 809	0.99974	0.99975	3 999	3 809	0.99974	0.99975	3 999	3 809	0.99974	0.99975	3 999	3 809	0.99974	15 992 001	1	14 505 579	1
G1738R	0.99975	3 999	3 809	0.99974	0.99975	3 999	3 809	0.99974	0.99975	3 999	3 809	0.99974	0.99725	363	345	0.99711	15 992 001	1	14 505 579	1
T1685I	0.99975	3 999	3 809	0.99974	0.99975	3 999	3 809	0.99974	0.99975	3 999	3 809	0.99974	0.9965	285	271	0.99633	15 992 001	1	14 505 579	1
T1685A	0.99975	3 999	3 809	0.99974	0.99975	3 999	3 809	0.99974	0.99975	3 999	3 809	0.99974	0.99475	189	181	0.99449	15 992 001	1	14 505 579	1
C44Y	0.99975	3 999	3 809	0.99974	0.99975	3 999	3 809	0.99974	0.99975	3 999	3 809	0.99974	0.99425	173	165	0.99397	15 992 001	1	14 505 579	1
C44F	0.99975	3 999	3 809	0.99974	0.99975	3 999	3 809	0.99974	0.99975	3 999	3 809	0.99974	0.99275	137	130	0.99239	15 992 001	1	14 505 579	1
C64Y	0.99975	3 999	3 809	0.99974	0.98675	74	71	0.98611	0.99975	3 999	3 809	0.99974	0.98525	67	64	0.98454	15 992 001	1	14 505 579	1
T37K	0.99575	234	223	0.99554	0.99925	1 332	1 269	0.99921	0.71825	2.55	2.48	0.71227	0.86075	6.18	5.93	0.85580	597	0.99833	552	0.99819
C39R	0.99975	3 999	3 809	0.99974	0.8225	4.63	4.46	0.81688	0.99975	3 999	3 809	0.99974	0.99825	570	543	0.99816	15 992 001	1	14 505 579	1
C61G	0.98925	92	88	0.98872	0.764	3.24	3.13	0.75791	0.957	22	21	0.95504	0.54325	1.19	1.18	0.54136	2 048	0.99951	1 863	0.99946
C47G	0.99775	443	422	0.99764	0.99775	443	422	0.99764	0.93825	15.19	14.52	0.93556	0.14425	0.17	0.18	0.14931	6 738	0.99985	6 132	0.99984
M18T	0.99825	570	543	0.99816	0.99575	234	223	0.99554	0.80225	4.06	3.91	0.79639	0.10875	0.12	0.13	0.11296	2 314	0.99957	2 125	0.99953
C39Y	0.99975	3 999	3 809	0.99974	0.99975	3 999	3 809	0.99974	0.71725	2.54	2.46	0.71128	0.00475	0.0048	0.005	0.00499	10 144	0.99990	9 383	0.99989
M1775R	0.99975	3 999	3 809	0.99974	0.94675	17.78	16.98	0.94438	0.271	0.37	0.38	0.27704	0.00175	0.0018	0.0018	0.00184	1 487	0.99933	1 459	0.99932
L22S	0.99975	3 999	3 809	0.99974	0.99975	3 999	3 809	0.99974	0.00025	0.00025	0.00026	0.00026	0.00025	0.00025	0.00026	0.00026	1	0.50000	1	0.50000
M1775K	0.99975	3 999	3 809	0.99974	0.59975	1.50	1.47	0.59591	0.00025	0.00025	0.00026	0.00026	0.00025	0.00025	0.00026	0.00026	1	0.50000	1	0.50000
C47F	0.49325	0.97	0.97	0.49357	0.245	0.32	0.34	0.25110	0.00025	0.00025	0.00026	0.00026	0.00075	0.00075	0.00079	0.00079	0.00024	0.00024	0.00026	0.00026
R1699W	0.07225	0.078	0.081	0.07532	0.017	0.017	0.018	0.01782	0.00025	0.00025	0.00026	0.00026	0.00225	0.0023	0.0024	0.00236	1.95E-5	1.95E-5	2.14E-5	2.14E-5
T1720A	0.00025	0.00025	0.00026	0.00026	0.00025	0.00025	0.00026	0.00026	0.00025	0.00025	0.00026	0.00026	0.00025	0.00025	0.00026	0.00026	6.25E-8	6.25E-8	6.89E-8	6.89E-8
P1859R	0.00025	0.00025	0.00026	0.00026	0.00025	0.00025	0.00026	0.00026	0.00075	0.00075	0.00079	0.00079	0.00025	0.00025	0.00026	0.00026	1.88E-7	1.88E-7	2.07E-7	2.07E-7
R1751Q	0.00025	0.00025	0.00026	0.00026	0.00025	0.00025	0.00026	0.00026	0.00125	0.0013	0.0013	0.00131	0.00425	0.0043	0.0045	0.00446	3.13E-7	3.13E-7	3.45E-7	3.45E-7
E1682K	0.00375	0.0038	0.004	0.00394	0.00025	0.00025	0.00026	0.00026	0.00175	0.0018	0.0018	0.00184	0.00025	0.00025	0.00026	0.00026	6.60E-6	6.60E-6	7.27E-6	7.27E-6
M1652I	0.00025	0.00025	0.00026	0.00026	0.00025	0.00025	0.00026	0.00026	0.00025	0.00025	0.00026	0.00026	0.00475	0.0048	0.005	0.00499	6.25E-8	6.25E-8	6.89E-8	6.89E-8
I31M	0.00025	0.00025	0.00026	0.00026	0.00025	0.00025	0.00026	0.00026	0.00175	0.0018	0.0018	0.00184	0.00325	0.0033	0.0034	0.00341	4.38E-7	4.38E-7	4.83E-7	4.83E-7
I1858L	0.00125	0.0013	0.0013	0.00131	0.00725	0.0073	0.0077	0.00761	0.00025	0.00025	0.00026	0.00026	0.00025	0.00025	0.00026	0.00026	3.13E-7	3.13E-7	3.45E-7	3.45E-7
P1776H	0.01525	0.015	0.016	0.01599	0.00025	0.00025	0.00026	0.00026	0.00025	0.00025	0.00026	0.00026	0.00625	0.0063	0.0066	0.00656	3.87E-6	3.87E-6	4.27E-6	4.27E-6
F1662S	0.00975	0.0098	0.01	0.01023	0.00025	0.00025	0.00026	0.00026	0.00075	0.00075	0.00079	0.00079	0.02325	0.024	0.025	0.02436	7.39E-6	7.39E-6	8.14E-6	8.14E-6
V1804D	0.00025	0.00025	0.00026	0.00026	0.0585	0.062	0.065	0.06107	0.00025	0.00025	0.00026	0.00026	0.00025	0.00025	0.00026	0.00026	6.25E-8	6.25E-8	6.89E-8	6.89E-8
A1669S	0.00025	0.00025	0.00026	0.00026	0.0585	0.062	0.065	0.06107	0.04625	0.048	0.051	0.04834	0.00025	0.00025	0.00026	0.00026	1.21E-5	1.21E-5	1.33E-5	1.33E-5
N132K	0.00025	0.00025	0.00026	0.00026	0.00725	0.0073	0.0077	0.00761	0.00025	0.00025	0.00026	0.00026	0.09575	0.11	0.11	0.09958	6.25E-8	6.25E-8	6.89E-8	6.89E-8
D67Y	0.00475	0.0048	0.005	0.00499	0.245	0.32	0.34	0.25110	0.00025	0.00025	0.00026	0.00026	0.00025	0.00025	0.00026	0.00026	1.19E-6	1.19E-6	1.32E-6	1.32E-6
M1652T	0.99975	3 999	3 809	0.99974	0.99975	3 999	3 809	0.99974	0.00125	0.0013	0.0013	0.00131	0.02325	0.024	0.025	0.02436	5	0.83347	5	0.83347
K45Q	0.47025	0.89	0.89	0.47159	0.69575	2.29	2.23	0.68997	0.84375	5.40	5.19	0.83846	0.21075	0.27	0.28	0.21672	4.79	0.82739	4.63	0.82245
Sensitivity	0.92 (23/25)			0.92 (23/25)	0.76 (19/25)			0.76 (19/25)	0.64 (16/25)			0.64 (16/25)	0.60 (15/25)			0.60 (15/25)		0.84 (21/25)		0.84 (21/25)
Specificity	0.87 (13/15)			0.87 (13/15)	0.67 (10/15)			0.67 (10/15)	0.93 (14/15)			0.93 (14/15)	0.87 (13/15)			0.87 (13/15)		0.87 (13/15)		0.87 (13/15)
Accuracy	0.90 (36/40)			0.90 (36/40)	0.73 (29/40)			0.73 (29/40)	0.75 (30/40)			0.75 (30/40)	0.70 (28/40)			0.70 (28/40)		0.85 (34/40)		0.85 (34/40)

See S13 Table for details.

S15 Table. Basic and corrected probability of pathogenicity when using the MWW method

Variant	Colony Size assay				Liquid Medium Assay				Spot Formation assay				Yeast Localization assay				Combined odds (CS x SF)	Combined probability (CS x SF)	Corrected odds combined (CS x SF)	Corrected probability combined (CS x SF)
	Probability	Odds	Corrected odds	Corrected probability	Probability	Odds	Corrected odds	Corrected probability	Probability	Odds	Corrected odds	Corrected probability	Probability	Odds	Corrected odds	Corrected probability				
M1689R	0.99975	3 999	3 809	0.99974	0.99975	3 999	3 809	0.99974	0.99425	173	165	0.99397	0.99975	3 999	3 809	0.99974	691 479	1	627 381	1
G1706E	0.99975	3 999	3 809	0.99974	0.99975	3 999	3 809	0.99974	0.99425	173	165	0.99397	0.99975	3 999	3 809	0.99974	691 479	1	627 381	1
A1708E	0.99975	3 999	3 809	0.99974	0.99975	3 999	3 809	0.99974	0.99425	173	165	0.99397	0.99975	3 999	3 809	0.99974	691 479	1	627 381	1
S1715R	0.99975	3 999	3 809	0.99974	0.99975	3 999	3 809	0.99974	0.99425	173	165	0.99397	0.99975	3 999	3 809	0.99974	691 479	1	627 381	1
L1764P	0.99975	3 999	3 809	0.99974	0.99975	3 999	3 809	0.99974	0.99425	173	165	0.99397	0.99975	3 999	3 809	0.99974	691 479	1	627 381	1
I1766S	0.99975	3 999	3 809	0.99974	0.99975	3 999	3 809	0.99974	0.99425	173	165	0.99397	0.99975	3 999	3 809	0.99974	691 479	1	627 381	1
G1788V	0.99975	3 999	3 809	0.99974	0.99975	3 999	3 809	0.99974	0.99425	173	165	0.99397	0.99975	3 999	3 809	0.99974	691 479	1	627 381	1
V1838E	0.99975	3 999	3 809	0.99974	0.99975	3 999	3 809	0.99974	0.99425	173	165	0.99397	0.99975	3 999	3 809	0.99974	691 479	1	627 381	1
G1738R	0.99975	3 999	3 809	0.99974	0.99975	3 999	3 809	0.99974	0.99425	173	165	0.99397	0.99975	3 999	3 809	0.99974	691 479	1	627 381	1
T1685I	0.99975	3 999	3 809	0.99974	0.99975	3 999	3 809	0.99974	0.99425	173	165	0.99397	0.99975	3 999	3 809	0.99974	691 479	1	627 381	1
T1685A	0.99975	3 999	3 809	0.99974	0.99975	3 999	3 809	0.99974	0.99425	173	165	0.99397	0.99975	3 999	3 809	0.99974	691 479	1	627 381	1
C44Y	0.99975	3 999	3 809	0.99974	0.99975	3 999	3 809	0.99974	0.99425	173	165	0.99397	0.99975	3 999	3 809	0.99974	691 479	1	627 381	1
C44F	0.99975	3 999	3 809	0.99974	0.99975	3 999	3 809	0.99974	0.99425	173	165	0.99397	0.99975	3 999	3 809	0.99974	691 479	1	627 381	1
C64Y	0.99975	3 999	3 809	0.99974	0.84875	5.61	5.39	0.84355	0.99425	173	165	0.99397	0.63725	1.76	1.72	0.63245	691 479	1	627 381	1
T37K	0.99975	3 999	3 809	0.99974	0.98325	59	56	0.98244	0.97425	38	36	0.97303	0.99975	3 999	3 809	0.99974	151 302	1	137 418	1
C39R	0.99975	3 999	3 809	0.99974	0.30025	0.43	0.44	0.30607	0.99425	173	165	0.99397	0.99975	3 999	3 809	0.99974	691 479	1	627 381	1
C61G	0.99975	3 999	3 809	0.99974	0.76275	3.21	3.11	0.75666	0.99425	173	165	0.99397	0.97825	45	43	0.97721	691 479	1	627 381	1
C47G	0.97425	38	36	0.97303	0.99225	128	122	0.99187	0.99425	173	165	0.99397	0.49675	0.99	0.99	0.49690	6 542	0.99985	5 943	0.99983
M18T	0.99525	210	200	0.99501	0.93475	14.33	13.69	0.93193	0.80725	4.19	4.04	0.80144	0.55375	1.24	1.23	0.55145	878	0.99886	806	0.99876
C39Y	0.99975	3 999	3 809	0.99974	0.99975	3 999	3 809	0.99974	0.99425	173	165	0.99397	0.00825	0.0083	0.0087	0.00866	691 479	1	627 381	1
M1775R	0.99975	3 999	3 809	0.99974	0.89225	8.28	7.93	0.88807	0.75825	3.14	3.03	0.75215	0.00025	0.00025	0.00026	0.00026	12 543	0.99992	11 558	0.99991
L22S	0.99975	3 999	3 809	0.99974	0.64925	1.85	1.81	0.64419	0.00025	0.00025	0.00026	0.00026	0.00025	0.00025	0.00026	0.00026	1	0.50000	1	0.50000
M1775K	0.99975	3 999	3 809	0.99974	0.81475	4.40	4.24	0.80903	0.00025	0.00025	0.00026	0.00026	0.00025	0.00025	0.00026	0.00026	1	0.50000	1	0.50000
C47F	0.96125	25	24	0.95947	0.27425	0.38	0.39	0.28028	0.00025	0.00025	0.00026	0.00026	0.00025	0.00025	0.00026	0.00026	0.0062	0.00616	0.0062	0.00618
R1699W	0.02325	0.024	0.025	0.02436	0.00025	0.00025	0.00026	0.00026	0.00025	0.00025	0.00026	0.00026	0.00025	0.00025	0.00026	0.00026	5.95E-6	5.95E-6	6.55E-6	6.55E-6
T1720A	0.00025	0.00025	0.00026	0.00026	0.00025	0.00025	0.00026	0.00026	0.00025	0.00025	0.00026	0.00026	0.00025	0.00025	0.00026	0.00026	6.25E-8	6.25E-8	6.89E-8	6.89E-8
P1859R	0.00025	0.00025	0.00026	0.00026	0.00425	0.0043	0.0045	0.00446	0.00025	0.00025	0.00026	0.00026	0.00025	0.00025	0.00026	0.00026	6.25E-8	6.25E-8	6.89E-8	6.89E-8
R1751Q	0.00025	0.00025	0.00026	0.00026	0.00025	0.00025	0.00026	0.00026	0.00025	0.00025	0.00026	0.00026	0.00025	0.00025	0.00026	0.00026	6.25E-8	6.25E-8	6.89E-8	6.89E-8
E1682K	0.00225	0.0023	0.0024	0.00236	0.00025	0.00025	0.00026	0.00026	0.00125	0.0013	0.0013	0.00131	0.00025	0.00025	0.00026	0.00026	2.82E-6	2.82E-6	3.11E-6	3.11E-6
M1652I	0.00025	0.00025	0.00026	0.00026	0.00025	0.00025	0.00026	0.00026	0.00025	0.00025	0.00026	0.00026	0.00525	0.0053	0.0055	0.00551	6.25E-8	6.25E-8	6.89E-8	6.89E-8
I31M	0.00025	0.00025	0.00026	0.00026	0.00025	0.00025	0.00026	0.00026	0.00875	0.0088	0.0093	0.00918	0.00325	0.0033	0.0034	0.00341	2.21E-6	2.21E-6	2.43E-6	2.43E-6
I1858L	0.00025	0.00025	0.00026	0.00026	0.00775	0.0078	0.0082	0.00813	0.00025	0.00025	0.00026	0.00026	0.00025	0.00025	0.00026	0.00026	6.25E-8	6.25E-8	6.89E-8	6.89E-8
P1776H	0.00125	0.0013	0.0013	0.00131	0.00025	0.00025	0.00026	0.00026	0.00025	0.00025	0.00026	0.00026	0.00025	0.00025	0.00026	0.00026	3.13E-7	3.13E-7	3.45E-7	3.45E-7
F1662S	0.00025	0.00025	0.00026	0.00026	0.00025	0.00025	0.00026	0.00026	0.00025	0.00025	0.00026	0.00026	0.01175	0.012	0.012	0.01232	6.25E-8	6.25E-8	6.89E-8	6.89E-8
V1804D	0.00025	0.00025	0.00026	0.00026	0.00175	0.0018	0.0018	0.00184	0.00025	0.00025	0.00026	0.00026	0.00025	0.00025	0.00026	0.00026	6.25E-8	6.25E-8	6.89E-8	6.89E-8
A1669S	0.00025	0.00025	0.00026	0.00026	0.00175	0.0018	0.0018	0.00184	0.01775	0.018	0.019	0.01860	0.00025	0.00025	0.00026	0.00026	4.52E-6	4.52E-6	4.98E-6	4.98E-6
N132K	0.00175	0.0018	0.0018	0.00184	0.01225	0.012	0.013	0.01285	0.00025	0.00025	0.00026	0.00026	0.06325	0.068	0.071	0.06600	4.38E-7	4.38E-7	4.83E-7	4.83E-7
D67Y	0.00225	0.0023	0.0024	0.00236	0.32175	0.47	0.49	0.32731	0.00025	0.00025	0.00026	0.00026	0.00025	0.00025	0.00026	0.00026	5.64E-7	5.64E-7	6.22E-7	6.22E-7
M1652T	0.99975	3 999	3 809	0.99974	0.75525	3.09	2.99	0.74915	0.00875	0.0088	0.0093	0.00918	0.03275	0.034	0.035	0.03428	35	0.97245	35	0.97244
K45Q	0.37925	0.61	0.62	0.38366	0.79275	3.83	3.69	0.78681	0.99425	173	165	0.99397	0.63725	1.76	1.72	0.63245	106	0.99062	103	0.99034
Sensitivity	0.96 (24/25)			0.96 (24/25)	0.64 (16/25)			0.64 (16/25)	0.76 (19/25)			0.76 (19/25)	0.64 (16/25)			0.64 (16/25)		0.84 (21/25)		0.84 (21/25)
Specificity	0.87 (13/15)			0.87 (13/15)	0.80 (12/15)			0.80 (12/15)	0.93 (14/15)			0.93 (14/15)	0.87 (13/15)			0.87 (13/15)		0.87 (13/15)		0.87 (13/15)
Accuracy	0.93 (37/40)			0.93 (37/40)	0.70 (28/40)			0.70 (28/40)	0.83 (33/40)			0.83 (33/40)	0.73 (29/40)			0.73 (29/40)		0.85 (34/40)		0.85 (34/40)

See S13 Table for details.

S16 Table. Quantitative analysis of S24 Fig

Assay	Method	Number of variants correctly classified					Number of variants not classified			Number of variants misclassified			Balance	Sensitivity	Specificity	Accuracy
		Pathogenic in class 5	Pathogenic in class 5 + 4	Neutral in class 1	Neutral in class 1 + 2	Total (class 1 + 2 + 4 + 5)	Pathogenic in class 3	Neutral in class 3	Total	Pathogenic in class 1 or 2	Neutral in class 5 or 4	Total		Pathogenic in class 5 + 4	Neutral in class 1 + 2	Total (class 1 + 2 + 4 + 5)
Colony Size	Standard	23	23	12	13	36	2	1	3	0	1	1	35	0.92	0.87	0.90
	Standard with reference	22	23	8	13	36	2	1	3	0	1	1	35	0.92	0.87	0.90
	MWW	22	24	9	13	37	0	1	1	1	1	2	35	0.96	0.87	0.93
Liquid Medium	Standard	18	20	8	12	32	4	2	6	1	1	2	30	0.80	0.80	0.80
	Standard with reference	18	19	8	10	29	5	4	9	1	1	2	27	0.76	0.67	0.73
	MWW	15	16	7	12	28	8	3	11	1	0	1	27	0.64	0.80	0.70
Spot Formation	Standard	15	17	13	14	31	4	1	5	4	0	4	27	0.68	0.93	0.78
	Standard with reference	15	16	10	14	30	5	1	6	4	0	4	26	0.64	0.93	0.75
	MWW	18	19	10	14	33	2	0	2	4	1	5	28	0.76	0.93	0.83
Yeast Localization	Standard	16	16	7	13	29	3	2	5	6	0	6	23	0.64	0.87	0.73
	Standard with reference	14	15	7	13	28	4	2	6	6	0	6	22	0.60	0.87	0.70
	MWW	15	16	9	13	29	3	2	5	6	0	6	23	0.64	0.87	0.73

Framed numbers highlight the differences with the results obtained using the probability system of classification (S6 Table).

S17 Table. Effect of functional assay parameters upon the quantile system of classification

Parameters	Standard method	Standard with reference method	MWW method	Figure
Position of pathogenic mutations	**+++** Shift from the neutral sector towards the pathogenic sector increases both sensitivity and specificity.	**+++** As in the standard method.	**+++** As in the standard method but the increase stops when no more overlap with the BRCA1 reference distribution.	S25A
Position of neutral mutations	**+++** Shift from the neutral sector towards the pathogenic sector decreases both sensitivity and specificity.	**+++** As in the standard method.	**+++** No effect, except a complete misclassification of the pathogenic mutations when the WT reference distribution is left on one side of all the neutral and pathogenic distributions with no overlap.	S25B
Position of WT reference	**0** No effect.	**+++** Shift of the median towards the null value decreases sensitivity and specificity. Negative values of the WT reference median invert the fluctuation of the best cut-off.	**+++** No effect, except a complete misclassification of the pathogenic mutations when the WT reference distribution falls outside of the range of the neutral and pathogenic distributions, with no overlap.	S25C
Experimental sensitivity	**+++** Decrease of the experimental sensitivity decreases specificity. This is advantageous, as the neutral region is contaminated by pathogenic mutations (presence of false negatives).	**+++** As in the standard method.	**+++** As in the standard method.	S25D
Experimental specificity	**+++** Decrease of the experimental specificity decreases sensitivity. This is advantageous, as the pathogenic region is contaminated by neutral mutations (presence of false positives).	**+++** As in the standard method.	**+++** As in the standard method.	S25D
Number of neutral mutations	**0** Decrease of neutral mutations slightly shifts the best cut-off distribution towards the neutral sector, but no impact on sensitivity and specificity.	**0** As in the standard method.	**0** As in the standard method.	S26A
Number of pathogenic mutations	**0** Decrease of pathogenic mutations slightly shifts the best cut-off distribution towards the pathogenic sector, but no impact on sensitivity and specificity.	**0** As in the standard method.	**0** No effect.	S26A
Number of mutant values	**0** No effect (and no more best cut-off fluctuation when n_{mutant} = 1).	**0** No effect (and no more best cut-off fluctuation when $n_{mutant} = n_{BRCA1}$ = 1).	**0** Decrease of the number of mutant values narrows the best cut-off fluctuation, with no effect on variant classification. No more best cut-off fluctuation when $n_{mutant} = n_{BRCA1}$ = 1.	S26B
Number of WT BRCA1 values	**0** No effect.	**+++** Loss of sensitivity with the decrease of the number of BRCA1 values, but maximal sensitivity when n_{BRCA1} = 1. No more best cut-off fluctuation when $n_{mutant} = n_{BRCA1}$ = 1.	**0** Decrease of the number of mutant values narrows the best cut-off fluctuation, with no effect on variant classification. No more best cut-off fluctuation when $n_{mutant} = n_{BRCA1}$ = 1.	S26B
Range of mutant distributions	**0** No effect (and no more best cut-off fluctuation when the range is null).	**0** No effect (and no more best cut-off fluctuation when the range of mutant and BRCA1 distributions are null).	**0** As in the standard with reference method.	S27
Range of WT BRCA1 distribution	**0** No effect.	**0** No effect (decrease of the range decreases the best cut-off fluctuation, with no more fluctuation when the range of mutant and BRCA1 distributions are null).	**0** No effect (and no more best cut-off fluctuation when the range of mutant and BRCA1 distributions are null).	S27

Sensitivity and specificity are defined here as the distance between the best cut-off fluctuation and the position of the pathogenic and neutral mutations, respectively, which reflects the accuracy of the quantile system of variant classification. For instance, the MWW method exhibits a null sensitivity in variant classification if the p values of the pathogenic mutations are in the grey area (class 3), and shows a maximal sensitivity in variant classification if the p values are in the pink area (class 4) or beyond (class 5). Misclassification is defined as class 4 or 5 for the neutral mutations and class 2 or 1 for the pathogenic mutations. See S9 Table for further details. The framed text indicates differences, as compared to the probability system of classification (S9 Table)
