# Supplementary material for: Functional Assessment of Genetic Variants with Outcomes Adapted to Clinical Decision-Making
Source: PLoS Genet. 2016 Jun 6;12(6):e1006096. doi: 10.1371/journal.pgen.1006096 (PMC4894565; doi:10.1371/journal.pgen.1006096)
Supplement: S1 Text — (PDF) [file pgen.1006096.s002.pdf]

Supplementary Methods

Outline

1. Definitions
2. Symbols frequently used
3. Colony size and Liquid Medium assays in glucose media
4. Western blot
5. The ProClass toolbox developed for the probabilistic classification of experimental data
6. Experimental data assembling
7. The MWW method
8. Sensitivity and specificity computation using the standard method
9. Difference between the standard method and the standard with reference methods
10. Sensitivity and specificity computation using the MWW method
11. Best cut-off, best sensitivity and best specificity computation
12. Bootstrap procedure A to define three different fluctuations of the best cut-off
13. Quantile computation after bootstrap procedure A
14. Probability system of classification
15. Exact best cut-off distribution
16. Properties of the probability of pathogenicity
    16.1. Associated risk
    16.2. Paucity of experimental data impairs the variant classification
    16.3. Paucity of bootstrap performed impairs the variant classification
    16.4. Accuracy of the probability system (exact distribution)
    16.5. Accuracy of the probability system (bootstrap)
17. Combined probability of pathogenicity
18. Independent functional assays
19. Corrected probability of pathogenicity
20. Incorporation of the probability of pathogenicity into posterior probability models (Bayesian inference)
21. The WT reference
22. Procedure to include new data (VUS) in the validated Colony Size, Liquid Medium, Spot Formation or Yeast Localization assays
23. Procedure to adapt the classification model to other functional assays
24. Advantages of these procedures compared to the 2-component models of variant classification
25. Limits of the nonparametric model (MWW method and probability system)
26. Procedure to adapt the model to other experiment system requiring a decision-making based on cut-off
27. The quantile system is not adapted to variant classification
    27.1. Presentation of the quantile system and variant classification
    27.2. The quantile system improves the variant classification when data is lacking
28. References

1. Definitions

Mutant, mutation: in this manuscript, these terms are equivalent to "variant", with no pathogenic connotation.

Ties: statistical term used to designate "identical values".

WT reference: backbone cDNA sequence carrying each assessed variant.

Cut-off: value that allows separation of the mutations into two categories: pathogenic or neutral. Depending on the functional assay, the pathogenic category lies above or below the cut-off, with the neutral category in the opposite position.

Sensitivity: proportion of pathogenic mutations correctly classified. In functional assessment, this is equivalent to the proportion of pathogenic mutations in the pathogenic area. This area lies above or below the cut-off, depending on the assay used.

Specificity: proportion of neutral mutations correctly classified. In functional assessment, this is equivalent to the proportion of neutral mutations in the neutral area. This area lies above or below the cut-off, depending on the assay used.

Accuracy: proportion of mutations correctly classified.

Best cut-off: cut-off value associated with the Youden's index (see **S2 Fig**).

Best sensitivity and specificity: values associated with the best cut-off, and thus with the Youden's index. The term "best" is frequently omitted as the sensitivity and specificity of an assay is always the best among the possible sensitivities and specificities.

Experimental data: data from experiments, as opposed to data from bootstrap analysis, which corresponds to a computer-assisted sampling of the experimental data.

Experimental best cut-off: best cut-off obtained from experimental data, as opposed to the best cut-off obtained from bootstrap analysis.

Experimental sensitivity, specificity and accuracy: best sensitivity, best specificity and best accuracy obtained from experimental data and associated with the experimental best cut-off, as opposed to the sensitivity, specificity and accuracy of the probability systems of classification, obtained after bootstrap analysis.

Initial sensitivity and specificity: equivalent to experimental best sensitivity and specificity. The term "initial" can be used to designate the sensitivity and specificity of the data (experimental or theoretical), since they are computed using the initial position of the neutral and pathogenic mutations, before bootstrap analysis.

Standard method: medians of the mutants are used to find the best cut-off.

Standard with reference method: as in the standard method, except that the best cut-off identified is divided by the median of the WT BRCA1 reference. Thus, the best cut-off is a value relative to the BRCA1 median value, with no unit. In fact, the standard with reference method is not different from the standard method for the experimental data. However, these methods differ during bootstrap analysis, since the raw and relative best cut-offs fluctuate differently.

Raw best cut-off: the term "raw" indicates that the best cut-off value is not divided by the WT BRCA1 median. Therefore, the raw best cut-off has the unit of the experimental data (e.g., cells per colony in the Colony Size assay).

Relative best cut-off: The term "relative" indicates that the best cut-off value is divided by the WT BRCA1 median.

MWW method: as in the standard method, except that each mutant is not represented by a median value but by a p value, as explained in **S4 Fig**.

CDF: cumulative distribution function, see **S6 Fig**.

Probability system of classification: system that uses the fluctuation of the best cut-off to derive probabilities of pathogenicity for each assessed variant (**S6 Fig**). Such probabilities allow the use of a five-class nomenclature to classify variants, as shown in **S1 Table**. Of note, the system is based on an average CDF.

Sensitivity of the probability system of classification: proportion of pathogenic mutations within the class 4 or 5 (see **Fig 2B**). This sensitivity has to be distinguished from the experimental sensitivity.

Specificity of the probability system of classification: proportion of neutral mutations within the class 1 or 2 (see **Fig 2B**). This specificity has to be distinguished from the experimental specificity.

Probability unit: lowest potential incrementation within the average CDF. In an exact distribution, this unit is equal to 1 / $n_{best\ exact}$ if at least three consecutive best cut-off values from the exact best cut-off distribution are not repeated. Otherwise, the value of the unit is higher. In an approximate distribution (bootstrap), this unit is equal to 1 / $n_{bootstrap}$ if at least three consecutive best cut-off values from the best cut-off distribution are not repeated. Otherwise, the value of the unit is higher.

Quantile system of classification: this system is an alternative to the probability system of classification. It is a very simple approach to generate probabilistic classifications.

However, as demonstrated below, this system is not adapted to the classification of variants.

System of classification: designates either the probability or the quantile system.

Accuracy of a system of classification: capability of the probability or quantile system to attribute a class 1 or 2 to the neutral mutations, and a class 4 or 5 to the pathogenic mutations. Of note, a completely accurate system never attributes the class 3 and never misclassifies variants. A system fully inaccurate attributes the class 3 only, or totally misclassifies the neutral and pathogenic variants.

Classification model: computational model that combines a method of best cut-off computation (standard, standard with reference or MWW method) and a system of classification (probability or quantile system).

2. Symbols frequently used

$n_{neutral}$	number of neutral mutations
$n_{pathogenic}$	number of pathogenic mutations
n_{mutant}	number of values within every mutant (implicating an equal number of values between each mutant)
n_{BRCA1}	number of values in the WT BRCA1 reference
$n_{bootstrap}$	number of bootstraps performed to estimate the best cut-off fluctuation
n_{best}	number of best cut-off values obtained after bootstrap ($n_{best} = n_{bootstrap}$)
n_{diff}	number of different best cut-off values obtained after bootstrap
$n_{best\ exact}$	number of best cut-off values in the exact best cut-off distribution (equal to the number of sampling possibilities)
$n_{exact\ diff}$	number of different best cut-off values in the exact best cut-off distribution

3. Colony size and Liquid Medium assays in glucose media

All of the clones from the Colony Size assay were assessed in glucose media to control the absence of any intrinsic growth defect, which would disturb the classification of the pathogenic missense mutations (**S3B Fig**). The rare clones, 10% below the median of the BRCA1 or Vector cells, after glucose induction, were removed from the study before analysis in galactose media.

As for the Colony Size assay, all of the clones from the Liquid Medium assay were assessed in glucose media to control the absence of any intrinsic growth defect (**S8B Fig**). Of note, the 126 clones used in the Colony Size and Liquid Medium assays were the same, except for 2 clones, due to the screening in glucose media.

4. Western blot

Western blots were performed as previously described [11]. One among three independent clones from each category was selected for analysis. Membranes were probed with an anti-BRCA1 monoclonal antibody (MS110, Calbiochem, Billerica, MA, USA, 1:200 dilution), then with a secondary peroxidase-conjugated anti-mouse antibody (Jackson Immunoresearch, West Grove, PA, USA, 1:10,000 dilution). To control for loading variation, stripped membranes were probed with an anti-Tubulin antibody (YL1/2, AbD serotec, Oxford, UK, 1:2,000 dilution) followed by a secondary peroxidase-conjugated anti-rat antibody (Jackson Immunoresearch, 1:5,000 dilution), or with an anti-β actin antibody (8224, abcam, 1:5,000 dilution) followed by a secondary peroxidase-conjugated anti-mouse antibody (Jackson Immunoresearch, 1:10,000 dilution). The ImageJ software was used to quantify signal intensities in every lane (full lanes quantified).

Western-blot analyses were performed to ascertain that growth recoveries were not related to any defect in BRCA1 protein expression (**S20 Fig**). In contrast, mutations showing the largest

number of cells per colony were associated with an increase in protein levels. This confirms what we previously reported for the Y1853X mutation and truncated forms of BRCA1 [11], and suggests that protein levels, clearly higher than the WT BRCA1 control level, predict a classification as pathogenic by the Colony Size, Liquid Medium, Spot Formation or Yeast Localization assay.

5. The ProClass toolbox developed for the probabilistic classification of experimental data

The Probabilistic Classification (ProClass) toolbox compiles the R codes [29] and raw data used in this article. The toolbox is designed with the purpose to facilitate the analysis of most kind of functional assay data, and to provide a probabilistic classification of variants. This toolbox is also adapted to other experimental data, provided that they include at least one positive and one negative control. ProClass is available on line at: http://xfer.curie.fr/get/tvsjyy4dUno/ProClass_toolbox.zip. The following sections 6 to 14 describe the computational procedures used in ProClass. Sections 22 and 23 explain how to integrate ProClass during functional assessment.

6. Experimental data assembling

As mentioned in the description of the functional assays, three independent transformants, also referred to as "clones", were selected for each transformation. This means that each strain, described in **S11 Table**, is represented by three clones. For the Colony Size assay, the 40 missense mutations were separated into four batches of 10 mutations. In the first batch, the three clones from each mutation were analyzed in three independent experiments. The three clones from the WT BRCA1 and the Vector strain were systematically used as a control. This gave 9 colony size values for each WT BRCA1, mutated BRCA1 or Vector control strain in the first batch. At this stage, no normalization was performed, meaning that the dispersion of the 9 values includes both the inter-experiment and the inter-clonal variation. The same was performed for the three other batches of 10 missense mutations, with the WT BRCA1 and the Vector strains as a control. Next, to assemble the results from the four batches, the following formula was applied: $x_{ijk} \times \tilde{x}_{BRCA1\ batch\ 1} / \tilde{x}_{BRCA1\ batch\ k}$, with x_{ijk} being the colony size value of the clone i (i = 1 to 3) in the experiment j (j = 1 to 3) for the batch k (k = 2 to 4), and with $\tilde{x}_{BRCA1\ batch\ 1}$ and $\tilde{x}_{BRCA1\ batch\ k}$ being the median value of the 9 colony size values from the WT BRCA1 strain in the batch 1 and k respectively. This means that, in batch 2, 3 and 4, the values were adjusted such that the median value of the 9 WT BRCA1 colony size values is equal to the WT BRCA1 median value of batch 1. This also means that the inter-batches variation was not considered. For the WT BRCA1 and Vector strains, the 9 adjusted values of the 4 batches were kept, meaning that the final distribution of these two strains was composed of 36 values.

The same method was applied to the Liquid Medium assay. Of note, the clones used in the Colony Size assay were mostly the same used in the Liquid Medium assay, which allows the comparison of the relative efficiency of these two assays. For the Spot Formation assay, the same method was used, except that the three clones from each strain were assessed once in independent experiments, which led to 3 instead of 9 values. Finally, after adjusting and assembling the values from the four batches, the distributions of the mutant and WT BRCA1 strains were composed of 3 and 12 values respectively. No Vector control strain was used for this assay ("no spot formation" is the theoretical negative control). For the Yeast Localization assay, the same method as for the Spot Formation assay was applied, but without data normalization, due to the WT BRCA1 values close to zero, which otherwise would severely amplify data variation. Of note, the images used in the Spot Formation assay were the same

used in the Yeast Localization assay, which allows the comparison of the relative efficiency of these two assays.

7. The MWW method

The distribution of each mutant was compared to the distribution of the WT BRCA1 using the Mann-Whitney-Wilcoxon (MWW) test. The p value of this test gives the probability to obtain the observed overlap of the two compared distributions, in the random sample, assuming that the two distributions are identical in the population from which is performed the sampling (statistical population). The null hypothesis H_0 is $P(x_{mutant\ i} > x_{BRCA1\ j}) = P(x_{mutant\ i} < x_{BRCA1\ j})$. If H_0 is true, this means that the probability $P(x_{mutant\ i} > x_{BRCA1\ j})$, of having a mutant value $x_{mutant\ i}$ above a BRCA1 value $x_{BRCA1\ j}$, is equal to the probability $P(x_{mutant\ i} < x_{BRCA1\ j})$, of having a mutant value $x_{mutant\ i}$ below a BRCA1 value $x_{BRCA1\ j}$. In an upper-sided MWW test, the alternative hypothesis is that the mutant values tend to be above the BRCA1 values, which is written as $P(x_{mutant\ i} > x_{BRCA1\ j}) > P(x_{mutant\ i} < x_{BRCA1\ j})$ [30]. Importantly, the p values computed here are not used to reject or not reject the null hypothesis of the test, but to quantify the overlap between the mutant and the WT BRCA1 distributions (**S4 Fig**). This defines relative positions of the mutant distributions using the WT BRCA1 distribution as a reference position. Thus, the assumptions necessary for the MWW test [30] are not required here. Relative positions are limited to the environment of the WT BRCA1 distribution. Indeed, pathogenic variants showing no overlap with the WT BRCA1 distribution have the same relative position (same p value), even if the functional assay identifies differences between them. Normal approximation and continuity correction were systematically applied to anticipate the presence of identical values (ties). For that, the R function used was wilcox.test(..., exact=FALSE, correct=TRUE). Of note, using these parameters, an increasing number of ties has a tendency to decrease the p value computed. With $n_{mutant} = 9$ and $n_{BRCA1} = 36$ (Colony Size and Liquid Medium assays), the p values of the MWW test theoretically range from 2.3e-6 to 1 without ties, and from 2e-11 to 1 with a maximum number of ties. With $n_{mutant} = 3$ and $n_{BRCA1} = 12$ (Spot Formation and Yeast Localization assay), the p values of the MWW test theoretically range from 0.0058 to 0.9962 without ties, and from 0.00014 to 1 with a maximum number of ties. In addition, particular results have to be mentioned when using correct= TRUE and one-tailed tests. For instance, the p value obtained for two distributions that perfectly overlap, (e.g., wilcox.test(1:3, 1:3, alternative = "less", exact=FALSE, correct=TRUE)) is slightly upper than 0.5. This has no consequence in variant classification, since p values are used as relative positions, as long as the number of values per variant remains identical. In addition, the p value obtained for two distributions that show the same unique value (e.g., wilcox.test(c(1,1,1), c(1,1,1), alternative = "less", exact=FALSE, correct=TRUE)) is equal to 1. In such extreme situations, it is recommended to use correct=FALSE.

For the Colony Size, Liquid Medium and Yeast Localization assays, upper-sided MWW tests were performed (the hypothesis being that the distributions of the pathogenic mutations are above the distribution of the WT BRCA1 reference). The R function used was wilcox.test(WT reference values, mutant i values, alternative = "less", exact=FALSE, correct=TRUE). For the Spot Formation assay, lower-sided MWW tests were performed (the hypothesis being that the distributions of the pathogenic mutations lie below the distribution of the WT BRCA1 reference). The R function used was wilcox.test(WT reference values, mutant i values, alternative = "greater", exact=FALSE, correct=TRUE). Results are summarized in **S4 Table**.

8. Sensitivity and specificity computation using the standard method

Medians from the mutant distributions were ordered (as in the waterfall representation, **Fig 1A**) and means were computed between every two consecutive medians. These mean values

were defined as all possible cut-offs within the ordered medians. Sensitivity and specificity were computed for each cut-off. Sensitivity corresponded to the proportion of pathogenic mutant medians above (Colony Size, Liquid Medium and Yeast Localization assays) or below (Spot Formation assay) the cut-off. Specificity corresponded to the proportion of neutral mutant medians below (Colony Size, Liquid Medium and Yeast Localization assays) or above (Spot Formation assay) the cut-off. Of note, cut-offs above or below all of the medians were not considered. This means that the sensitivity/specificity of (0, 1) and (1, 0) were excluded from the study. In **S2 Fig**, the confidence intervals of the sensitivity and specificity were computed with the binom.test(..., alternative = "two.sided", conf.level = 0.95) function of R, considering that the theoretical sensitivity or specificity is 100% (p = 1). The receiver operating characteristic (ROC) curve offers a visual representation of the sensitivities and specificities computed. For the x-axis, it is common to use 1-specificity more than specificity, as 1-specificity represents the false positive rate (FPR). The dotted line showed in **S2 Fig** indicates the positive diagonal, for which sensitivities = 1 - specificities. The top left corner of the ROC chart corresponds to sensitivity = 1 and specificity = 1 (1 - specificity = 0), which represents the optimal situation for any given assay.

9. Difference between the standard method and the standard with reference methods

We distinguish the standard method from the standard with reference method. The first uses raw cut-offs, meaning that the cut-off values are not divided by the median of the WT BRCA1 reference (see the definitions above). Thus, the cut-off values have the unit of the experimental data (e.g., cells per colony in the Colony Size assay). In the standard with reference method, cut-off values are divided by the median of the WT BRCA1 reference distribution. Nothing changes between these 2 methods when working with the experimental data (i.e., experimental sensitivity and specificity are the same for both methods). However, results change when performing bootstrap analysis, because in the standard with reference method, the fluctuation of the best cut-off is influenced by the fluctuation of the WT BRCA1 reference, which is not the case in the standard method, that only depends on sampling the neutral and pathogenic mutant values (described below).

10. Sensitivity and specificity computation using the MWW method

The same as the standard method was applied, but for ordered p values (e.g., **Fig 1B**) instead of medians. Thus, the final cut-offs analyzed in ROC curves were the intermediate p values between the ordered mutant p values.

11. Best cut-off, best sensitivity and best specificity computation

In the standard or MWW method, the best compromise between the highest sensitivity and specificity was determined by the nonparametric empirical Youden's index [31-33], which is defined as max[$sensitivity_i$ + $specificity_i$ - 1] for each cut-off i. The Youden's index corresponds to the dot on the ROC curve that maximizes the vertical distance between the positive diagonal and the ROC curve. Such dots were pinpointed as black numbers in **S2 Fig**. The best cut-off was defined as the cut-off of the Youden's index. The best sensitivity and best specificity were defined as those associated with the best cut-off. The standard method and the standard with reference methods generated systematically the same best experimental sensitivity and best experimental specificity, regardless of the data analyzed, since the best cut-off is the same in these two methods, except for the unit (raw best cut-off in the standard method, and relative best cut-off in the standard with reference method).

12. Bootstrap procedure A to define three different fluctuations of the best cut-off

Nonparametric random samplings [34] were performed as follows. The number of sampled values was systematically the number of values available in each mutant and in the WT BRCA1 reference. Thus, for each mutant, n_{mutant} values were randomly chosen with replacement. The same was performed for the BRCA1 distribution, with n_{BRCA1} values randomly chosen with replacement. As an example, using the Colony Size assay, the number of values randomly chosen was $n_{mutant} = 9$ and $n_{BRCA1} = 36$, since respectively 9 and 36 values were available in these distributions. In the Spot Formation assay, the number of values randomly chosen was $n_{mutant} = 3$ and $n_{BRCA1} = 12$, since respectively 3 and 12 values were available in these distributions. Next, using this new set of sampled data, we applied the three standard, standard with reference and MWW methods. In the standard method, medians of the mutant distributions were ordered, raw cut-offs were computed as described above, and the raw best cut-off value associated with the Youden's index of the ROC curve, was saved. In the standard with reference method, the raw best cut-off, identified by the standard method, was divided by the median of the WT BRCA1 reference computed on the new sample data and was saved. In the MWW method, p values for each mutant were computed as described above, and ordered. Next, cut-offs were computed as described above, and the best cut-off value, associated with the Youden's index of the ROC curve, was saved. In each of the three methods, if several cut-offs lead to the Youden's index, the median of these cut-offs was considered as the best cut-off. This procedure was repeated 2,000 times, to obtain 2,000 best cut-off values for each of the three methods. Next, the 2,000 bootstraps were repeated 20 times. This procedure is referred to as "bootstrap procedure A", which, in summary, generated 20 sets of 2,000 best cut-offs for each of the standard, standard with reference and MWW methods.

It is important to mention that, in the standard with reference method, best cut-off values are multiplication factors of the BRCA1 median value. This means that fluctuation of the BRCA1 median is included in the best cut-off fluctuation. For instance, with the Colony Size assay: during the random sampling i, if the raw best cut-off found is $b^*_{raw\,i} = 25{,}000$ cells per colony (the star indicates that the value comes from bootstrapping) and if the BRCA1 median is $\tilde{x}^*_{BRCA1\,i} = 10{,}000$, then the relative best cut-off is $b^*_i = b^*_{raw\,i}/\tilde{x}^*_{BRCA1\,i} = 2.5$. In the random sampling i+1, if $b^*_{raw\,i+1} = 25{,}000$ cells per colony and if $\tilde{x}^*_{BRCA1\,i+1} = 5{,}000$, then the relative best cut-off is $b^*_{i+1} = 5$. These examples highlight the fact that the relative best cut-off values can change, even if the raw best cut-off value of the standard method remains the same.

13. Quantile computation after bootstrap procedure A

In the first set of the bootstrap procedure A, a total of eleven quantiles, 0.1%, 0.5%, 1%, 2.5%, 5%, 50% (median), 95%, 97.5%, 99%, 99.5% and 99.9%, were computed from the 2,000 best cut-off values of the standard method (using the type 7 method of the quantile() function of R). This procedure was repeated for all of the 20 sets. Next, medians of the 20 values from each of the eleven quantiles were computed (of note, these quantiles could have been directly computed from the 20 × 2,000 = 40,000 best cut-off values, but medians of quantiles have the advantage to buffer the fluctuation of extreme quantiles). The same was performed for the standard with reference and MWW methods. Thus, eleven median quantiles were obtained for each of the three methods, shown in **S5 Table**. Of note, the basic (non-studentized pivotal) method was used to obtain the quantiles [35].

14. Probability system of classification

This system is based on rank methods [14]. The following procedure was separately applied to the three standard, standard with reference and MWW methods. In each of the 20 bootstrap

sets, resulting from the bootstrap procedure A, the 2,000 best cut-off values were sorted by ascending or descending order, depending on the position of the neutral and pathogenic sectors in the assay and depending on the method used: ascending order if the lowest values correspond to the neutral sector and the highest to the pathogenic sector (e.g., Colony Size assay + standard method), and descending order in the opposite case (e.g., MWW method, whatever assay is used). Next, the median of the 20 first values, from the 20 sorted sets, was computed, and the same for the next 1,999 subsequent ranks, to obtain a median distribution of the sorted best cut-offs. Next, the probability 1 / n_{best} = 0.0005 was attributed to each of the n_{best} = 2,000 values of the median distribution (see the theoretical example in **S6A-B Fig**). Probabilities were summed in the case of identical values, which finally resulted in n_{diff} values in the median distribution of the sorted best cut-offs (e.g., n_{diff} = 154 in the Yeast Localization assay, using the standard method, as shown in **S6C Fig** and **S12 Table**). From this, two cumulative distribution function (CDF) were generated, by simply carrying out the cumulative sum of the n_{diff} probabilities, with the first CDF that ranged from the probability p_1 to $p_{n\ diff} = 1$ and the second CDF that ranged from 0 to $p_{n\ diff\ -\ 1} < 1$. Next, the mean of the two CDF was computed, which resulted in an average CDF that associated a probability ($0 < p_i < 1$) for the n_{diff} different best cut-off values. This average CDF was obtained for each of the Colony Size, Liquid Medium, Spot Formation and Yeast Localization assays and for each of the standard, standard with reference and MWW methods (**S6C-E Fig**).

To attribute a probability of pathogenicity to an assessed variant, using the standard method, the median of this variant was positioned within the raw best cut-off values of the average CDF. Next, the probability of the average CDF, closest to the variant median, was assigned to the variant as a probability of pathogenicity (see the example in **S6B Fig**). The same procedure was applied to the standard with reference method, except that the median of the variant was divided by the median of the WT BRCA1 reference median (i.e., 11,200 cells per colony in the Colony Size assay), to fit the average CDF composed of relative best cut-off values. The same procedure was applied to the MWW method, except that the p value of the variant was used to fit the average CDF. The five-class nomenclature [26] was used to categorize the probabilities of pathogenicity, as in genetic/epidemiological methods (**S1 Table**).

Of note, interest in averaging the two initial CDF lies in the removal of the probabilities 0 and 1 in the resulting CDF, which could create infinite values during subsequent conversions. The second interest is that the average CDF copes with the absence of best cut-off fluctuation. In such situation, the average CDF is represented by a single best cut off value (equal to the experimental best cut-off) which has the probability 0.5. This means that in the absence of best cut-off fluctuation, the classification proposed by a given functional assay, is systematically "variant completely unknown". The convergence towards 0.5 is illustrated using the boundaries of the average CDF. If the number of best cut-off values is n_{best}, then the lowest probability within the CDF is $1/n_{best}$. Thus, in the CDF1, the boundaries of the cumulated probabilities are:

$$\left[\frac{1}{n_{best}}\ ;\ 1\right]$$

n_{best}: number of best cut-off values obtained after bootstrap

And in the CDF2:

$$\left[0\ ;\ 1-\frac{1}{n_{best}}\right]$$

Thus, in the average CDF, the boundaries are:

$$\left[\frac{\frac{1}{n_{best}}+0}{2} \; ; \; \frac{1+1-\frac{1}{n_{best}}}{2}\right] = \left[\frac{1}{2n_{best}} \; ; \; 1-\frac{1}{2n_{best}}\right]$$

And when $n_{best} = 1$, both boundaries of the average CDF are equal to 0.5.

15. Exact best cut-off distribution

In certain situations, it is possible to use the exact best cut-off distribution, instead of performing bootstrap analysis. This exact distribution is defined as all of the sampling possibilities, when sampling with replacement n_{mutant} values among the n_{mutant} available, for each variant, and n_{BRCA1} values among the n_{BRCA1} available for the WT reference. An example is shown in **S21B Fig**. The number of sampling possibilities can be predicted as follows. For one variant, composed of n_{mutant} different values, the number of sampling possibilities is:

$$\binom{2n_{mutant}-1}{n_{mutant}}$$

n_{mutant}: number of values in each mutant (implicating the same number in the different mutants)

With $n_{neutral}$ and $n_{pathogenic}$ variants, composed of the same number of values n_{mutant}, all different, the number of sampling possibilities is:

$$\binom{2n_{mutant}-1}{n_{mutant}}^{n_{neutral}+n_{pathogenic}}$$

$n_{neutral}$: number of neutral mutations
$n_{pathogenic}$: number of pathogenic mutations

Finally, with the inclusion of the WT reference (standard with reference and MWW methods), composed of n_{BRCA1} different values, the number of sampling possibilities is:

$$\binom{2n_{mutant}-1}{n_{mutant}}^{n_{neutral}+n_{pathogenic}} \times \binom{2n_{BRCA1}-1}{n_{BRCA1}}$$

n_{BRCA1}: number of values in the WT BRCA1 reference

As an example, with one neutral and one pathogenic mutation ($n_{neutral} = 1$ and $n_{pathogenic} = 1$), containing two values per mutant ($n_{mutant} = 2$) and two values in the WT BRCA1 reference ($n_{BRCA1} = 2$), the number of sampling possibilities is 27 (27 rows in the table of **S21B Fig**).
In the standard method, the formula shows that, if $n_{mutant} = 1$, then the exact best cut-off distribution corresponds to the experimental best cut-off, and the same for the standard with reference and MWW methods if, additionally, $n_{BRCA1} = 1$ (**S22A Fig**). The formula also shows that the number of sampling possibilities rapidly increases with the number of mutant

values, BRCA1 values, neutral variants and pathogenic variants, which prevents the use of the exact best cut-off distribution in most cases. For instance, in the Colony Size assay, the maximum number of sampling possibilities reaches 2e175 using the standard method and 6e195 using the MWW method. The bootstrap procedure has the advantage to by-pass this combinatory issue. However, it is important to mention that bootstrap affords an approximate distribution of the best cut-off, and that the quality of the approximation increases with the number of bootstraps $n_{bootstrap}$ performed.
Of note, two different sampling possibilities can result in the same best cut-off (for instance, the best cut-off between 10 and 20, and between 5 and 25, is 15 in both cases). Thus, even if the number of mutant values, BRCA1 values, neutral variants and pathogenic variants, are high (generating a high number of sampling possibilities), the final number of different best cut-off values $n_{exact\ diff}$, forming the average CDF of the probability system of classification, can be very low, even within an exact distribution.

16. Properties of the probability of pathogenicity

16.1. Associated risk

The probability of pathogenicity computed is the probability to have the best cut-off value below (ascending average CDF) or above (descending average CDF) the considered mutant, which is related to the probability of misclassification of this mutant, due to the fluctuation of the best cut-off. As an example, in the Colony Size assay using the standard method (ascending average CDF, **S6C Fig**, left panel), a variant with a probability of pathogenicity of 0.99 indicates that the best cut-off variable has a 99% chance to be below the variant median. In this example, the pathogenic area is above the best cut-off (**Fig 1A**), which means that this variant has a 1% probability of being classified as neutral (i.e., best cut-off above the variant median), due to the fluctuation of the best cut-off. In the same manner, again from the Colony Size assay using the standard method, a variant with a probability of pathogenicity of 0.001 indicates that the best cut-off variable has 0.1% chance to be below the variant median, which means that this variant has a 0.1% probability of being classified as pathogenic (i.e., best cut-off below the variant median), due to the fluctuation of the best cut-off, and, thus, has a 99.9% probability of being classified as neutral (i.e., best cut-off above the variant median). For descending average CDF, like in the Colony Size assay using the MWW method (**S6E Fig**, left panel), the reasoning is the opposite. It is important to mention that, contrary to genetic/epidemiological methods, for which the probability of pathogenicity computed measures a direct association of the variant with disease, here the probability of pathogenicity computed evaluates the risk to misclassify a variant, due to the fluctuation of the best cut-off, that depends on the fluctuation of the experimental data when performing the variant assessments. Finally, this probability of pathogenicity estimates the reproducibility of the variant classification obtained, following functional assessment.

16.2. Paucity of experimental data impairs the variant classification

As illustrated in the sections 16.4 and 16.5, the accuracy of the probability system decreases when the best cut-off distribution is composed of only a few different best cut-off values. Ultimately, when the best cut-off distribution is represented by one value (**S22A,C Figs**), the probability of pathogenicity 0.5 (class 3) is systematically attributed to all of the variants, regardless of their relative position. The weak number of different best cut-off values can result from (1) a low number of experimental replicates (**S18G Fig**), (2) a low number of neutral and pathogenic variants incorporated (except if the number of experimental replicates is high), and (3) a weak measurement accuracy leading to many ties (see the case of null ranges in **S19G Fig**). Thus, the probability system has the advantage to penalize functional assays with a paucity of experimental data. This situation is illustrated in the Yeast

localization assay using the standard method (**Fig 2B**). With n_{diff} = 154 (**S6C Fig** and **S12 Table**) none of the pathogenic mutations was classified as class 5.

16.3. Paucity of bootstrap performed impairs the variant classification

As illustrated in sections 16.4 and 16.5, accuracy of the probability system is decreased when the best cut-off distribution is composed of very few different best cut-off values, which is the case if the number of bootstraps performed ($n_{bootstrap}$) is low (**S22B Fig**). In contrast, if $n_{bootstrap}$ is high, then the accuracy of the probability system will only depends on the experimental data (**S22A Fig**).

16.4. Accuracy of the probability system (exact distribution)

The accuracy of a system of classification is defined as the capability to assign a class 1 or 2 to the neutral variant, and a class 4 or 5 to the pathogenic variants. A completely accurate system will never assign the class 3. A system fully inaccurate will assign the class 3 only, or will totally misclassify the neutral and pathogenic variants. In the probability system of classification, the accuracy is related to the probability unit of the average CDF. This probability unit is defined as the lowest potential incrementation within the average CDF or, which is equivalent, as the probability associated with a best cut-off value not repeated in the best cut-off distribution. In an exact distribution, this unit is equal to 1 / $n_{best\ exact}$, meaning 1 / 27 = 0.04 in **S21B Fig**. As shown in section 14, this defines the lowest and highest potential boundaries of the average CDF, derived from the exact distribution:

$$\left[\frac{1}{2n_{best\ exact}}\ ;\ 1-\frac{1}{2n_{best\ exact}}\right]$$

$n_{best\ exact}$: number of best cut-off values in the exact best cut-off distribution (equal to the number of sampling possibilities)

In the probability system of classification, this implies that a neutral variant cannot have a probability of pathogenicity less than 1 / ($2n_{best\ exact}$). In the same manner, a pathogenic variant cannot have a probability of pathogenicity more than 1 - 1 / ($2n_{best\ exact}$). Thus, if $n_{best\ exact} \leq 10$, the probability system will be unable to classify variants as class 2 or 1, because the lower boundary will not be less than 0.05 (**S1 Table**). Such limitations of the probability system of classification are recapitulated below:

Class 1 and 2: $\frac{1}{2n_{best\ exact}} < 0.05 \ \rightarrow\ n_{best\ exact} > 10$ (Relations 1)

Class 1: $\frac{1}{2n_{best\ exact}} < 0.001 \ \rightarrow\ n_{best\ exact} > 500$

Class 4 and 5: $1-\frac{1}{2n_{best\ exact}} \geq 0.95 \ \rightarrow\ n_{best\ exact} \geq 10$

Class 5: $1-\frac{1}{2n_{best\ exact}} > 0.99 \ \rightarrow\ n_{best\ exact} > 50$

Because $n_{best\ exact}$ is equal to the number of sampling possibilities, which is dependent on the number of values n_{mutant} in each mutant, the number of values n_{BRCA1} in the WT BRCA1 reference (except for the standard method), the number of neutral mutations $n_{neutral}$ (if n_{mutant} > 1) and the number of pathogenic mutations $n_{pathogenic}$ (if n_{mutant} > 1), this means that the accuracy of the probability system of classification is dependent on these parameters. As an example, in **S21D Fig**, with 1 neutral variant, 1 pathogenic variant, 2 values per variant and 2

values in the WT BRCA1 reference, the number of sampling possibilities is sufficient to allow the class 2 and class 4 classification, but not class 1 and class 5, in the standard with reference method.
Of note, an increasing number of ties (identical values) in the dataset, lowers the accuracy of the probability system of classification. Indeed, ties reduce the number of different best cut-off values, which reduces the interval of the average CDF (boundaries tend towards 0.5). Thus, the lowest $n_{best\ exact}$ values, indicated in Relation 1, are indicative. Moreover, the measurement accuracy has to be considered during functional assessment, to prevent ties.
The fact that two different sampling possibilities can result in the same computed best cut-off, also lowers the accuracy of the probability system of classification. As an example, even with no ties in the data set (**S21A Fig**), the reduced number of different best cut-off, finally obtained with the standard method (5 for 27 sampling possibilities), prevents the classification of variants other than class 3 (**S21C Fig**). This phenomenon, leading to identical best cut-off values, is not predictable, but it highlights again that the lowest $n_{best\ exact}$ values, indicated in Relation 1, are indicative.

16.5. Accuracy of the probability system (bootstrap)

The considerations, developed in the precedent section (16.4), are also valid when using the approximate best cut-off distribution (obtained by bootstrap), instead of the exact distribution, except that the number of bootstraps performed, $n_{bootstrap}$, is an additional parameter that influences the accuracy of the probability system of classification. More precisely, since the bootstrap procedure gives an estimation of the exact best cut-off distribution (**S22B Fig**), the effect of $n_{bootstrap}$ depends on $n_{best\ exact}$.
If $n_{bootstrap} << n_{best\ exact}$, then the lowest potential probability unit is:

$$\frac{1}{n_{bootstrap}}$$

$n_{bootstrap}$: number of bootstraps performed to estimate the best cut-off fluctuation

And the lowest and highest potential boundaries are:

$$\left[\frac{1}{2n_{bootstrap}}\ ;\ 1-\frac{1}{2n_{bootstrap}}\right]$$

If $n_{bootstrap} >> n_{best\ exact}$, then the lowest potential probability unit is:

$$\frac{1}{n_{best\ exact}}$$

$n_{best\ exact}$: number of best cut-off values in the exact best cut-off distribution (equal to the number of sampling possibilities)

And the lowest and highest potential boundaries are:

$$\left[\frac{1}{2n_{best\ exact}}\ ;\ 1-\frac{1}{2n_{best\ exact}}\right]$$

Using the probability system of classification, this implies that a neutral variant cannot have a probability of pathogenicity less than 1 / ($2n_{best\ exact}$), regardless of the number of bootstraps performed. This also implies that the lowest probability of pathogenicity of a neutral variant is overestimated if $n_{bootstrap} << n_{best\ exact}$. In the same manner, a pathogenic variant cannot have a probability of pathogenicity more than 1 - 1 / ($2n_{best\ exact}$), regardless of the number of bootstraps performed. This also implies that the highest probability of pathogenicity of a pathogenic variant is underestimated if $n_{bootstrap} << n_{best\ exact}$.

As an example, in **S21 Fig**, if two bootstraps are performed, then $n_{bootstrap} = 2 << n_{best\ exact} = 27$. If we consider that the two best cut off values, obtained with the standard with reference method, are different, then the average CDF is represented by the two values 0.25 and 0.75, with the boundaries [0.25; 0.75]. Thus, the probability of pathogenicity attributed to the neutral variant is 0.25, which is overestimated compared to the 0.02 probability given by the exact distribution (**S21D Fig**). In the same manner, the probability of pathogenicity attributed to the pathogenic variant is 0.75, which is underestimated as compared to the 0.98 probability given by the exact distribution (**S21D Fig**).

Of note, if the number of different best cut-off values is much lower than the number of bootstraps performed ($n_{diff} << n_{bootstrap}$), this suggests that the approximate best cut-off distribution is close to the exact one. For instance, in the Liquid Medium assay using the standard method (**S6C Fig**), the $n_{bootstrap}$ = 2,000 bootstraps generated an approximate best cut-off distribution, composed of n_{diff} = 126 different best cut-off values. Thus, in this case, the limitations of the probability system of classification are probably due to $n_{best\ exact}$ rather than $n_{bootstrap}$.

17. Combined probability of pathogenicity

Let us consider one of these three methods: standard, standard with reference or MWW. For each variant, four probabilities of pathogenicity were obtained, from the four Colony Size, Liquid Medium, Spot Formation and Yeast Localization assays (**S13-S15 Tables**). Combining these probabilities provides a final probability of pathogenicity. The model proposed was derived from the one used in genetic/epidemiological methods [15]. Probabilities were converted into odds in favor of pathogenicity using the formula:

$$O_i = \frac{p_i}{1 - p_i}$$

O_i: odds in favor of pathogenicity of the variant i ($0 \leq O_i < +\infty$)

p_i: probability of pathogenicity of the variant i ($0 \leq p_i \leq 1$)

With odds in favor of pathogenicity, the pathogenicity varies between 0 (absolutely neutral) and $+\infty$ (absolutely pathogenic). A variant i, of fully unknown significance, has $p_i = 0.5$ and thus $O_i = 1$. Only independent probabilities can be combined. Thus, probabilities from the Liquid Medium assays were excluded from the computation of the combined probabilities, since the Colony Size and Liquid Medium results were derived from the same yeast clones. In the same manner, probabilities from the Yeast Localization assays were excluded, since the Spot Formation and Yeast Localization results were derived from the same yeast clones and the same microscope picture acquisitions. Next, odds from the Colony Size and Spot Formation assays were multiplied:

$$O_{comb\ i} = O_{CS\ i} \times O_{SF\ i}$$

$O_{comb\ i}$: combined odds in favor of pathogenicity of the variant i ($0 \leq O_{comb\ i} < +\infty$)

And the combined probability of pathogenicity was obtained using the formula:

$$p_{comb\ i} = \frac{O_{comb\ i}}{1 + O_{comb\ i}}$$

$p_{comb\ i}$: combined probability of pathogenicity of the variant i ($0 \leq p_{comb\ i} < 1$)

As a reminder, the accuracy of the probability system of classification is decreased together with n_{mutant}, n_{BRCA1}, $n_{neutral}$ and $n_{pathogenic}$, or together with $n_{bootstrap}$ (see sections 16.4 and 16.5). This means that the probabilities of pathogenicity attributed to the assessed variants will tend towards 0.5. Thus, it is remarkable that an assay, showing low n_{mutant}, n_{BRCA1}, $n_{neutral}$ and $n_{pathogenic}$ values, or for which a low number of bootstraps has been performed, will have a low contribution in the combined probability of pathogenicity. Indeed, when the pathogenicity of a variant tends towards 0.5, the odds tend towards 1, which has a null effect in odds multiplication.

18. Independent functional assays

We estimate that the independence of two functional assays cannot be evaluated *a posteriori*, for instance by comparing the results of these assays. Indeed, in the *a posteriori* evaluation, the notion of independence is based on the hypothesis that the pathogenic variants can have a random position in the pathogenic area of a waterfall distribution. Thus, if we compare two assays, challenged by the same set of pathogenic mutations, the random position of the pathogenic variants, in each assay, should result in a low correlation between the variant medians, which would confirm the independence between the two assays. However, it is known that certain pathogenic mutations have intermediate effects on protein function [36]. Thus, it is difficult to ascertain a random position of the pathogenic mutations in the pathogenic area. Focusing on neutral mutations, rather than on pathogenic mutations, would not solve this issue, since intermediate effects cannot be excluded for neutral mutations. Thus, we propose to evaluate the independence of two assays *a priori*, with the following criteria:
1) The two assessments must have been performed independently. This means that the results from each assay must come from different transfections, different cellular clones, different experimental times, etc. The fact that the plasmids used are not exactly the same reinforces the independence. For instance, in the Spot Formation assay, the plasmids code for the mCherry-BRCA1 fusion protein, but not in the Colony Size assay. Thus, the plasmids used are different in these two assays.
2) What is monitored must be different. Following this, the Colony Size assay and the Liquid Medium assay are not independent, since both monitor the cell growth of yeast cells. If assays are not clearly associated with a protein function, then the measurements must be different. For instance, the Colony Size and the Spot Formation assays are not related to a known function of BRCA1, but the Colony Size assay monitors cell growth, while the Spot Formation assay monitors the formation of a cellular aggregate. Thus, the measurement is different for these two assays.
Based on these criteria, The Colony Size and the Liquid Medium assays cannot be considered as independent, and the same for the Spot Formation and the Yeast Localization assays.

19. Corrected probability of pathogenicity

The fluctuation of the best cut is influenced by the experimental (initial) sensitivity and specificity of a functional assay (**S16 Fig**) but not by the number of neutral and pathogenic mutations used to determine these parameters (**S17 Fig**). This could be problematic in variant

classification, since a 100% sensitivity and specificity of an assay, resulting from 2 neutral and 2 pathogenic mutations assessed, are not reliable. To overcome this, we propose an approach to correct the odds in favor of pathogenicity, that takes into account the number of mutants used to evaluate a functional assay, as follows:

$$O_{cor\ i} = (O_i - 1) \times f_{cor} + 1 \qquad \text{if } O_i \geq 1$$

$$O_{cor\ i} = \frac{1}{\left(\frac{1}{O_i} - 1\right) \times f_{cor} + 1} \qquad \text{if } O_i < 1$$

$$f_{cor} = \frac{n_{neutral} + n_{pathogenic}}{n_{neutral} + n_{pathogenic} + a}$$

$$p_{cor\ i} = \frac{O_{cor\ i}}{1 + O_{cor\ i}}$$

$O_{cor\ i}$:	corrected odds in favor of pathogenicity of the variant i
O_i:	odds in favor of pathogenicity of the variant i ($0 \leq O_i < +\infty$)
f_{cor}:	factor of correction
$n_{neutral}$:	number of neutral mutations used in the assay validation
$n_{pathogenic}$:	number of pathogenic mutations used in the assay validation
a:	integer that modulates the impact of $n_{neutral}$ and $n_{pathogenic}$
$p_{cor\ i}$:	corrected probability of pathogenicity of the variant i ($0 \leq p_{cor\ i} \leq 1$)

In this study, we chose a = 2 as a correcting factor (**S13-S15 Tables**), which results in f_{cor} = 0.5 for $n_{neutral} + n_{pathogenic} = 2$, f_{cor} = 0.8 for $n_{neutral} + n_{pathogenic} = 8$, f_{cor} = 0.9 for $n_{neutral} + n_{pathogenic} = 18$ and f_{cor} = 0.95 for $n_{neutral} + n_{pathogenic} = 38$ (**S22D Fig**). Since $n_{neutral}$ and $n_{pathogenic}$ are not distinguished in the correction, this method of correction is not appropriate if $n_{neutral}$ and $n_{pathogenic}$ are strongly unbalanced.

20. Incorporation of the probability of pathogenicity into posterior probability models (Bayesian inference)

The probability of pathogenicity derived from the best cut-off fluctuation can be used to compute a posterior probability of being pathogenic, considering the Bayes' theorem [37]:

$$O_{post\ i} = O_i \times O_{prior\ i}$$

$$O_{prior\ i} = \frac{p_{prior\ i}}{1 - p_{prior\ i}}$$

$O_{post\ i}$:	posterior odds in favor of pathogenicity of the variant i ($0 \leq O_{post\ i} < +\infty$)
O_i:	odds in favor of pathogenicity of the variant i ($0 \leq O_i < +\infty$)
$O_{prior\ i}$:	prior odds in favor of pathogenicity of the variant i ($0 \leq O_{prior\ i} < +\infty$)
$p_{prior\ i}$:	prior probability of pathogenicity of the variant i ($0 \leq p_{prior\ i} \leq 1$)

The prior probability represents the probability of pathogenicity of the variant i before any functional assessment. A prior probability of 0.5 can be used in the absence of any prior information. In this case, $O_{post\ i}$ = Oi. Prior probabilities can be provided by the GVGD Align model [38]. Then, the posterior probability of being pathogenic is obtained following:

$$p_{post\ i} = \frac{O_{post\ i}}{1 + O_{post\ i}}$$

$p_{post\ i}$: posterior probability of pathogenicity of the variant i ($0 \leq p_{post\ i} \leq 1$)

This method can also be applied to the combined odds ($O_{comb\ i}$) and the corrected odds ($O_{cor\ i}$), described above.

21. The WT reference

It is recommended to systematically add a WT reference control during variant assessment [4]. However, this may lead to divergent usage of this reference when interpreting results. The WT reference can either be included in the neutral category or be considered as a particular case, outside of the two neutral and pathogenic categories. The rational of the first choice is that the WT reference is by definition neutral (not pathogenic). The second choice is ruled by three concerns. First, since the WT reference distribution usually contains a number of values larger than in the other neutral distributions, the WT reference could have a strong weight in the final variant interpretation, if incorporated as an additional variant in the neutral category. Second, the WT reference corresponds to the backbone cDNA sequence, present in the expression plasmid, in which the mutations are introduced. In other words, neutral mutations have two differences in their sequences, compared two by two, while they only have one difference compared to the WT reference. Additionally, the variant classification obtained could be modified using another WT reference sequence, because of potential variant-sequence interactions. Thus, the WT reference represents more than an additional neutral variant. Third, variants showing intermediate effects have been reported [36]. This highlights the benefit of the WT reference taken as a special case, beyond the neutral and pathogenic categories, because a reference would be necessary for the identification of neutral variants with intermediate effects.

22. Procedure to include new data (VUS) in the validated Colony Size, Liquid Medium, Spot Formation or Yeast Localization assays

- Download the ProClass toolbox (see section 5).
- Read carefully the *README.doc* downloaded document.
- Request the desired plasmids, presented in this study.
- Generate the plasmids containing the VUS (new batch of variants).
- Perform the experiment as described above. For instance, with the Colony Size assay, test three independent clones in three independent experiments to obtain 9 final values for the VUS. Add the WT BRCA1 reference (plasmid pPT60 or pPT63 depending on the chosen assay), as well as the pathogenic G1706E (plasmid pPT147 or pPT161) and the neutral R1751Q (plasmid pPT119 or pPT120) mutations as a control. Add the Vector control (pJL48 plasmid) for the Colony Size or the Liquid Medium assays. This means that 9 values are also obtained for the WT BRCA1 reference and for each control.
- Consider the results as a new batch and include these results in the downloaded table (e.g., Colony Size data.txt table). For the G1706E, R1751Q and Vector controls, change the name of the new values, like G1706E.bis, in order to prevent the fusion of the new values with the values of G1706E already present in the table. In addition, set these G1706E.bis, R1751Q.bis and Vector.bis controls as "Other.reference" in the "Prior_classif" column.
- Execute the code of the *Code data analysis and representation.doc* file with the adapted settings (for the Colony Size assay, use the *Colony Size data proba 2000x20 type7 replac noref less (MWW).txt* file to generate the probability of being pathogenic using the MWW method).

- In the normalized data obtained, verify that the median of the controls (G1706E.bis, R1751Q.bis and Vector.bis) fit approximately the median of the preexisting values (G1706E, R1751Q and Vector).
- If the medians fit, use the probability of being pathogenic provided in the output results of the executed code. Results can be combined, corrected and included in a posterior probability model, as explained above.
- If the median of the controls (G1706E.bis, R1751Q.bis and Vector.bis) do not fit the median of the preexisting values (G1706E, R1751Q and Vector), a new best cut-off fluctuation has to be generated. For that, remove the ".bis" in the name of the controls, in the data table, and follow the complete instructions provided in the *README.doc* downloaded document.

23. Procedure to adapt the classification model to other functional assays

- Download the ProClass toolbox (see section 5).
- Select several neutral and pathogenic mutations formally classified by genetic/epidemiological methods. Favor, if possible, a similar number of neutral and pathogenic variants.
- Design the experiment. It is counseled to plan independent experiments and to have the WT reference systematically present in each experiment.
- Generate the expression vectors, carrying either the WT reference control, or the different neutral or pathogenic mutations.
- Fix the number of values per mutation that has to be obtained (e.g., 9 values per mutation in the Colony Size assay). This number must be the same for each variant (neutral, pathogenic and unknown). This number must also be systematically respected when subsequently adding neutral and pathogenic mutations, in order to improve the sensitivity and specificity of the functional assay, or when subsequently adding VUS for classification. In addition, the number of values expected for the WT reference control should be high, in order to improve the sensitivity of variant classification (**S18F Fig**).
- Of note, if the data show different numbers of values per neutral, pathogenic and unknown variants, the code of the *Code data analysis and representation.doc* file will reduce the data as explained in the *README.doc* file.
- Perform the experiments.
- Apply the complete instructions provided in the *README.doc* downloaded document.
- Results can be combined, corrected and included in a posterior probability model, as explained above.

24. Advantages of these procedures compared to the 2-component models of variant classification

Recently, statistical models of variant classification have been proposed [7,8]. These models: (1) are parametric (assumption about the distribution of the data in the statistical population), (2) require high statistical skills to analyze the data and fit the model and (3) need to be recomputed when additional VUS are included in the model, for classification purposes.

The model we propose alleviates these constraints. This model: (1) is nonparametric (no assumption about the distribution of the data in the statistical population), (2) does not require statistical skills to be handled and (3), following certain conditions, does not need recomputation when additional VUS are included. Moreover, the probability of pathogenicity computed with our model can be incorporated into posterior probability models (Bayesian inference), as described above, meaning that they can be handled in the manner as the probabilities computed by the 2-component models.

25. Limits of the nonparametric model (MWW method and probability system)

1) The number of values per variants (neutral, pathogenic and UV) must be the same. This allows a balanced contribution of each neutral and pathogenic variant to the best cut-off fluctuation, as well as a correct adequacy between the unknown variants assessed and the best cut-off distribution used to classify them. The R code, available online, manages different number of values but at the cost of loss of information, as the code reduces the number of values from all the variants to the lowest existing in the data.
2) A WT reference must be systematically present in each experiment performed. In addition, the MWW method requires that the WT reference is well embedded in the distribution of the neutral values (e.g., **S5A Fig**, left panel). A WT reference falling outside of the range of the neutral and pathogenic distributions impairs the sensitivity of the functional assay (**S15 Fig**). In such situation, it is recommended to use the standard method instead of the MWW method.
3) It is counseled to have at least 3 values per variant and more than 3 values for the WT reference (**S18 Fig**). In theory, our nonparametric model does not require a minimum number of values. However, the greater the number is, the better is the sensitivity and specificity of the model (see section 16 above). Of note, with a single value per mutation and per WT reference, the fluctuation of the best cut-off is null (the probability of being pathogenic, assigned to each variant, is systematically 0.5).

26. Procedure to adapt the model to other experiment system requiring a decision-making based on cut-off

The procedure described in section 23 can be applied to all situations, based on two categories (applying the standard method) or two categories + a reference category (applying the MWW method), as long as the best cut-off is able to fluctuate during bootstrap computation. If the two categories are divided into subcategories, as "variants" in the pathogenic and neutral categories, then the best cut-off fluctuation will be guaranteed with at least two different values in each subcategory. If the two categories are not subdivided, then the best cut-off fluctuation will be guaranteed with at least two different values in each category. Additionally, in the MWW method, the reference should be composed of at least two different values. Of note, if the data show different numbers of values per categories, the code of the *Code data analysis and representation.doc* file will reduce the data as explained in the *README.doc* file. Such data adjustment has been applied to the siRNA data presented in **Fig 3**. The initial data are made of 864 values for the positive control (siKIF11), 288 values for the negative controls (siGOLGA2 and siGL2) and 12 values for the unknown siRNAs assessed. The code reduces the data to 12 values for siKIF11, siGOLGA2 and siGL2, before any subsequent analysis. The reference category is not concerned by this adjustment.

27. The quantile system is not adapted to variant classification

<u>27.1. Presentation of the quantile system and variant classification</u>

We developed another approach to classify variants, referred to as "quantile system", which is very easy to apply. The quantile system is similar to the "grey zone approach" [39,40] but is extended to n zones. Since the classification as either pathogenic or neutral is dependent on the position of the variant above or below the best cut-off, we reasoned that the farther a variant is from the core of the best cut-off fluctuation, the more robust is its classification as either pathogenic or neutral. The quantile system consists of (1) overlapping the best cut-off fluctuation with the waterfall distribution of the mutants, (2) defining intervals in the best cut-off distribution associated to the five-class nomenclature proposed by Plon et al. (see **S1 Table** and **Fig 1**) and (3) classifying variants according to the position of their median (standard and standard with reference methods) or p value (MWW method) in the 5 intervals.

For instance, in **Fig 1B**, the p value of K45Q is located within the grey area. Thus, the variant is classified as class 3. The quantile system is fully documented in **S23 Fig**. The classification obtained in the four functional assays is depicted in **S24 Fig**. Globally, little differences were observed when comparing the classification obtained with the quantile (**S16 Table**) and the probability systems (**S6 Table**). Of note, the light blue, grey and pink areas depicted in **Fig 1**, **Fig 3A-B** and **S7, S9, S11, S13-S19**, **S25-S27 Figs** correspond to class 2, 3 and 4 of the quantile system, respectively.

<u>27.2. The quantile system improves the variant classification when data is lacking</u>

To detect potential flaws in the quantile system of classification, we recapitulated the analysis of theoretical situations, performed for the probability system (**S25-S27 Figs** and **S17 Table**). The results reveal a major flaw in the variant classification provided by the quantile system, as it does not penalize the paucity of data (**S26B** and **S27 Figs**). This was confirmed with the analysis from an exact best cut-off distribution (**S21F Fig**). In fact, the highest sensitivity, specificity and accuracy of the quantile system is reached when the best cut-off does not fluctuate (**S22C Fig**), which is favored by the paucity of data. In conclusion, the quantile system is not adapted to variant classification. This also suggests that the "grey zone approach" [39,40] is not an efficient method to identify a level of uncertainty within a given dataset.

28. References

27. Huh WK, Falvo JV, Gerke LC, Carroll AS, Howson RW, et al. (2003) Global analysis of protein localization in budding yeast. Nature 425: 686-691.
28. Winzeler EA, Shoemaker DD, Astromoff A, Liang H, Anderson K, et al. (1999) Functional characterization of the S. cerevisiae genome by gene deletion and parallel analysis. Science 285: 901-906.
29. R Core Team (2014) R: A Language and Environment for Statistical Computing. Vienna, Austria: R Foundation for Statistical Computing.
30. Conover WJ (1999) Practical nonparametric statistics third edition: Editions John Wiley & Sons, inc. 584 p.
31. Youden WJ (1950) Index for rating diagnostic tests. Cancer 3: 32-35.
32. Fluss R, Faraggi D, Reiser B (2005) Estimation of the Youden Index and its associated cutoff point. Biom J 47: 458-472.
33. Perkins NJ, Schisterman EF (2006) The inconsistency of "optimal" cutpoints obtained using two criteria based on the receiver operating characteristic curve. Am J Epidemiol 163: 670-675.
34. Kulesa A, Krzywinski M, Blainey P, Altman N (2015) Points of Significance: Sampling distributions and the bootstrap. Nat Meth 12: 477-478.
35. Carpenter J, Bithell J (2000) Bootstrap confidence intervals: when, which, what? A practical guide for medical statisticians. Stat Med 19: 1141-1164.
36. Spurdle AB, Whiley PJ, Thompson B, Feng B, Healey S, et al. (2012) BRCA1 R1699Q variant displaying ambiguous functional abrogation confers intermediate breast and ovarian cancer risk. J Med Genet 49: 525-532.
37. Lindor NM, Guidugli L, Wang X, Vallee MP, Monteiro AN, et al. (2012) A review of a multifactorial probability-based model for classification of BRCA1 and BRCA2 variants of uncertain significance (VUS). Hum Mutat 33: 8-21.
38. Tavtigian SV, Byrnes GB, Goldgar DE, Thomas A (2008) Classification of rare missense substitutions, using risk surfaces, with genetic- and molecular-epidemiology applications. Hum Mutat 29: 1342-1354.

39. Ray P, Le Manach Y, Riou B, Houle TT (2010) Statistical evaluation of a biomarker. Anesthesiology 112: 1023-1040.
40. Cannesson M, Le Manach Y, Hofer CK, Goarin JP, Lehot JJ, et al. (2011) Assessing the diagnostic accuracy of pulse pressure variations for the prediction of fluid responsiveness: a "gray zone" approach. Anesthesiology 115: 231-241.
