# Supplementary material for: Functional Assessment of Genetic Variants with Outcomes Adapted to Clinical Decision-Making
Source: PLoS Genet. 2016 Jun 6;12(6):e1006096. doi: 10.1371/journal.pgen.1006096 (PMC4894565; doi:10.1371/journal.pgen.1006096)
Supplement: S1 Fig — RING domain (amino acid 8–96); BRCT, BRCA1 C-terminal domains (amino acid 1646–1736 and 1760–1855). Pathogenic and neutral mutations are in red and blue, respectively. Fourteen mutations (3 neutral and 11 pathogenic) map within the RING domain. An additional neutral mutation, N132K, flanks the Cter part of this domain, resulting in 15 mutations located in the Nter extremity of BRCA1. Twenty-five mutations (11 neutral and 14 pathogenic) lie in the BRCT domain, at the Cter extremity of the protein. Of note, the RING domain suffers from a lack of neutral missense mutations classified by genetic/epidemiological methods, explaining why only 3 neutral mutations from our selected panel, lie in this domain. Moreover, no pathogenic missense mutations, between the amino acids 65 and 1684, are documented in the BRCA1 mutation databases (S2 Table). Therefore, this study was restricted to the RING and BRCT domains of BRCA1. (PDF) [file pgen.1006096.s003.pdf]

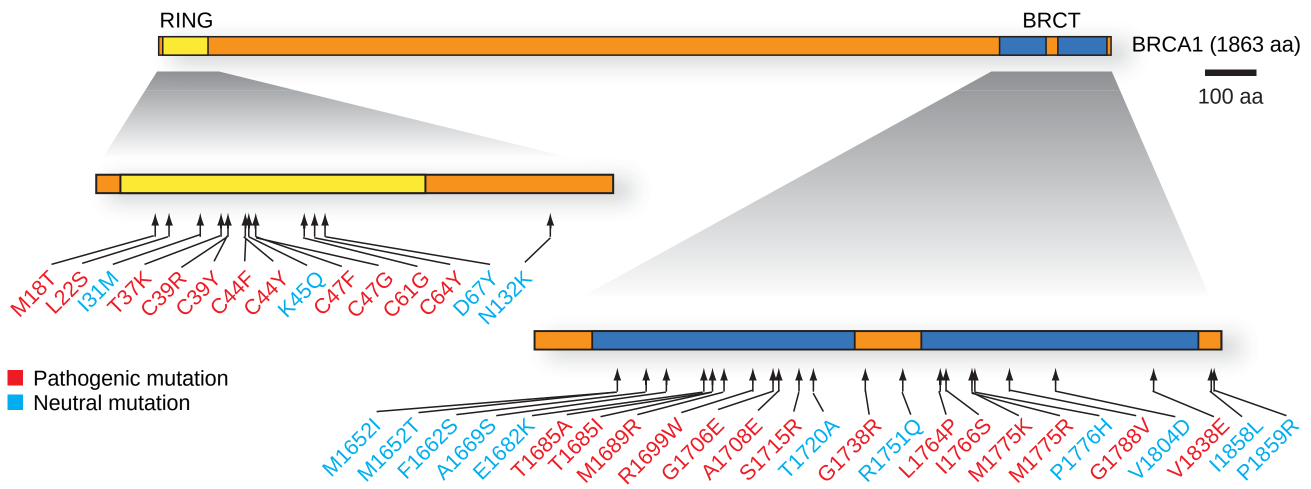

S1 Fig. Position of the BRCA1 missense mutations selected

RING domain (amino acid 8–96); BRCT, BRCA1 C-terminal domains (amino acid 1646–1736 and 1760–1855). Pathogenic and neutral mutations are in red and blue, respectively. Fourteen mutations (3 neutral and 11 pathogenic) map within the RING domain. An additional neutral mutation, N132K, flanks the Cter part of this domain, resulting in 15 mutations located in the Nter extremity of BRCA1. Twenty-five mutations (11 neutral and 14 pathogenic) lie in the BRCT domain, at the Cter extremity of the protein. Of note, the RING domain suffers from a lack of neutral missense mutations classified by genetic/epidemiological methods, explaining why only 3 neutral mutations from our selected panel, lie in this domain. Moreover, no pathogenic missense mutations, between the amino acids 65 and 1684, are documented in the BRCA1 mutation databases (S2 Table). Therefore, this study was restricted to the RING and BRCT domains of BRCA1.
