# Supplementary material for: Functional Assessment of Genetic Variants with Outcomes Adapted to Clinical Decision-Making
Source: PLoS Genet. 2016 Jun 6;12(6):e1006096. doi: 10.1371/journal.pgen.1006096 (PMC4894565; doi:10.1371/journal.pgen.1006096)
Supplement: S2 Fig — (A-D) Standard method. The medians of the mutant distributions were ordered (as in the waterfall distribution, Fig 1A) and each average position between two consecutive medians was defined as a cut-off. For example, in Fig 1A, the cut-off between the two first mutations, M1689R and V1838E, was (1,877,333 + 1,621,333) / 2 = 1,749,333 cells per colony. Next, sensitivity was defined as the proportion of pathogenic mutant medians above (for the Colony Size, Liquid Medium and Yeast Localization assays) or below (for the Spot Formation assay) a selected cut-off. The associated specificity was defined as the proportion of neutral mutant medians below (Colony Size, Liquid Medium and Yeast Localization assays) or above (Spot Formation assay) the same selected cut-off. For example, for the cut-off between M1689R and V1838E in Fig 1A, the sensitivity was 1/25 = 4% and the specificity was 15/15 = 100%. Sensitivity and specificity were computed for each cut-off (left panels). Areas surrounding the curves delimit the 95% confidence interval according to the binomial law. The ROC curve (right panel) pinpoints the best cut-off (black number), meaning the cut-off that maximizes both sensitivity and specificity of the assay. Precisely, the best cut-off is the one associated with the highest vertical distance of the ROC curve to the dotted diagonal. This highest vertical distance is referred to as "Youden's index", which is equal to max[sensitivity + specificity—1]. In other words, the best cut-off is the cut-off of the Youden's index. Other cut-off values are also positioned on the ROC curve (grey numbers). Blue, red and orange dots on the curves of the left and right panels represent the different cut-offs tested. The black vertical bar, in the left panel, pinpoints the best cut-off defined on the ROC curve. (E-H) MWW method. As in A-D for mutant p values, instead of mutant medians. In all assays, sensitivity was defined as the proportion of pathogenic mutant p values below a select [file pgen.1006096.s004.pdf]

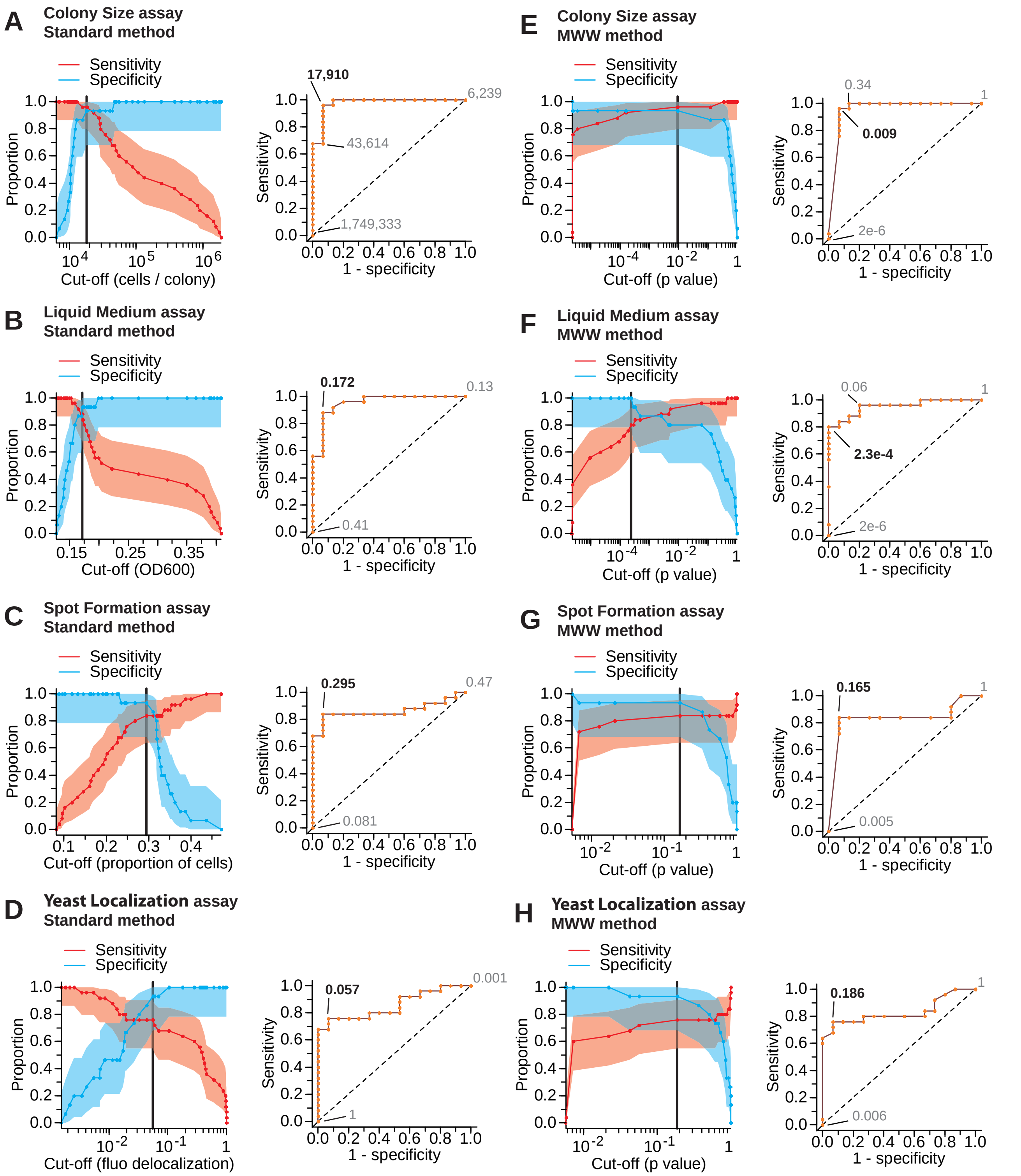

S2 Fig. Experimental best cut-off, experimental sensitivity and experimental specificity of functional assays

(**A-D**) Standard method. The medians of the mutant distributions were ordered (as in the waterfall distribution, Fig 1A) and each average position between two consecutive medians was defined as a cut-off. For example, in Fig 1A, the cut-off between the two first mutations, M1689R and V1838E, was (1,877,333 + 1,621,333) / 2 = 1,749,333 cells per colony. Next, sensitivity was defined as the proportion of pathogenic mutant medians above (for the Colony Size, Liquid Medium and Yeast Localization assays) or below (for the Spot Formation assay) a selected cut-off. The associated specificity was defined as the proportion of neutral mutant medians below (Colony Size, Liquid Medium and Yeast Localization assays) or above (Spot Formation assay) the same selected cut-off. For example, for the cut-off between M1689R and V1838E in Fig 1A, the sensitivity was 1/25 = 4% and the specificity was 15/15 = 100%. Sensitivity and specificity were computed for each cut-off (left panels). Areas surrounding the curves delimit the 95% confidence interval according to the binomial law. The ROC curve (right panel) pinpoints the best cut-off (black number), meaning the cut-off that maximizes both sensitivity and specificity of the assay. Precisely, the best cut-off is the one associated with the highest vertical distance of the ROC curve to the dotted diagonal. This highest vertical distance is referred to as "Youden's index", which is equal to max[sensitivity + specificity - 1]. In other words, the best cut-off is the cut-off of the Youden's index. Other cut-off values are also positioned on the ROC curve (grey numbers). Blue, red and orange dots on the curves of the left and right panels represent the different cut-offs tested. The black vertical bar, in the left panel, pinpoints the best cut-off defined on the ROC curve.

(**E-H**) MWW method. As in **A-D** for mutant p values, instead of mutant medians. In all assays, sensitivity was defined as the proportion of pathogenic mutant p values below a selected cut-off, and the associated specificity was defined as the proportion of neutral mutant p values above the same selected cut-off.

(**A**, **E**) Colony Size assay.

(**B**, **F**) Liquid Medium assay.

(**C**, **G**) Spot Formation assay.

(**D**, **H**) Yeast Localization assay.
