# Supplementary material for: Functional Assessment of Genetic Variants with Outcomes Adapted to Clinical Decision-Making
Source: PLoS Genet. 2016 Jun 6;12(6):e1006096. doi: 10.1371/journal.pgen.1006096 (PMC4894565; doi:10.1371/journal.pgen.1006096)
Supplement: S3 Fig — (A) Dotplot distribution of colony sizes. For each missense variant, the nine represented values result from three independent clones examined in three independent experiments. For the BRCA1 reference and the Vector control, the 36 values result from three independent clones examined in twelve independent experiments (represented in the three panels, except for the Vector values absent in the top panel). Grey bar, median; dotted horizontal line, median of BRCA1; black horizontal line, experimental best cut-off. The top panel (Nter extremity of BRCA1) has a y-axis scale magnified compared to the middle and bottom panels (Cter extremity of BRCA1). (B) As in A with glucose instead of galactose media (see the S1 Text) to verify that each clone had no intrinsic growth defect, independent of WT or mutated BRCA1 expression. The three independent clones from A were examined in one experiment. (PDF) [file pgen.1006096.s005.pdf]

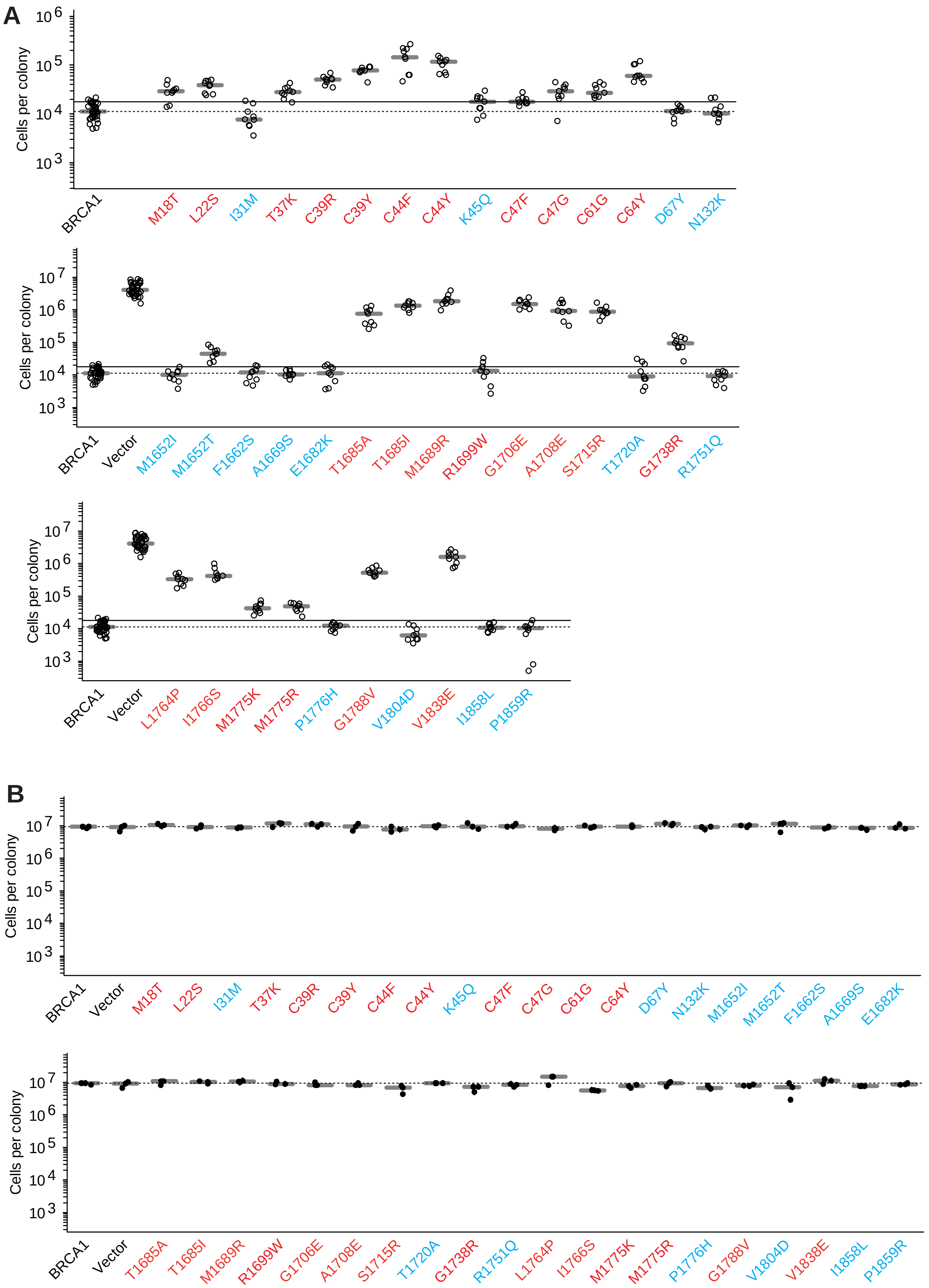

S3 Fig. Supplemental information in the Colony Size assay

(**A**) Dotplot distribution of colony sizes. For each missense variant, the nine represented values result from three independent clones examined in three independent experiments. For the BRCA1 reference and the Vector control, the 36 values result from three independent clones examined in twelve independent experiments (represented in the three panels, except for the Vector values absent in the top panel). Grey bar, median; dotted horizontal line, median of BRCA1; black horizontal line, experimental best cut-off. The top panel (Nter extremity of BRCA1) has a y-axis scale magnified compared to the middle and bottom panels (Cter extremity of BRCA1).

(**B**) As in **A** with glucose instead of galactose media (see the S1 Text) to verify that each clone had no intrinsic growth defect, independent of WT or mutated BRCA1 expression. The three independent clones from **A** were examined in one experiment.
