# Supplementary material for: Functional Assessment of Genetic Variants with Outcomes Adapted to Clinical Decision-Making
Source: PLoS Genet. 2016 Jun 6;12(6):e1006096. doi: 10.1371/journal.pgen.1006096 (PMC4894565; doi:10.1371/journal.pgen.1006096)
Supplement: S4 Fig — (A) Upper-sided MWW test. The theoretical examples are based on the Colony Size assay but are also valid for the Liquid Medium and Yeast Localization assays. Each distribution of the WT BRCA1 reference (black) and the missense mutation (purple) are composed of 8 theoretical values, represented by 8 dots in the diagram. The p value of the MWW test is used to score the overlap of the mutant and the WT BRCA1 distributions. See the S1 Text for full details. From left to right: (1) when all the mutant values are below the BRCA1 values, the upper-sided MWW test results in a p value close to 1; (2) the p value decreases when the mutant distribution begins to overlap the BRCA1 distribution; (3) the p value is approximately 0.5 when the two distributions completely overlap; (4) the p value continues to decrease when the mutant distribution is above the BRCA1 distribution, with a partial overlap; (5) finally, the p value is lowest when the mutant distribution is fully above the BRCA1 distribution. In theory, neutral and pathogenic mutations should have a p value close to 0.5 and 0, respectively, as depicted by the color scale below the diagram. However, the absolute p value attributed to each variant is not determinant. What is significant is the relative positions between the mutant distributions, indicated by the p values, using the WT BRCA1 distribution as a reference position. The lowest p values represent systematically the pathogenic mutations, and the highest the neutral mutations. Thus, the upper-sided MWW test is used when pathogenic mutations are above the neutral ones in the experimental data. (B) Lower-sided MWW test. All of the theoretical examples shown are based on the Spot Formation assay. As in the upper-sided MWW test, the lowest and highest p values still represent the pathogenic and neutral mutations, respectively, but the pathogenic mutations are below the neutral ones in the experimental data. (PDF) [file pgen.1006096.s006.pdf]

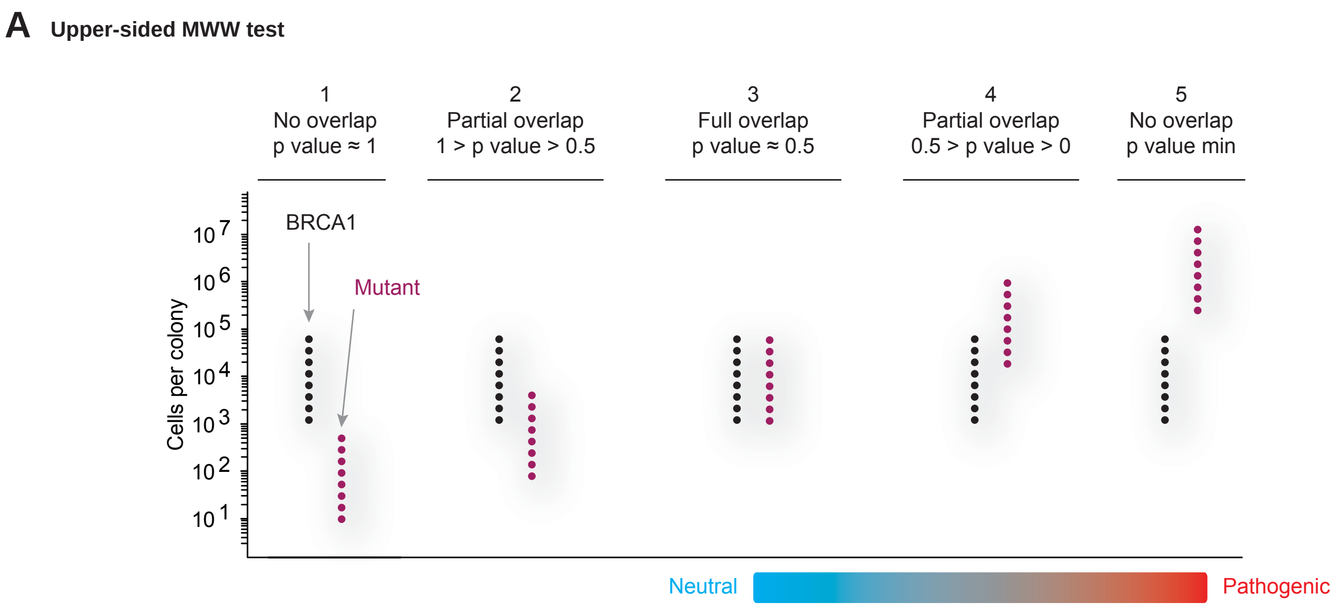

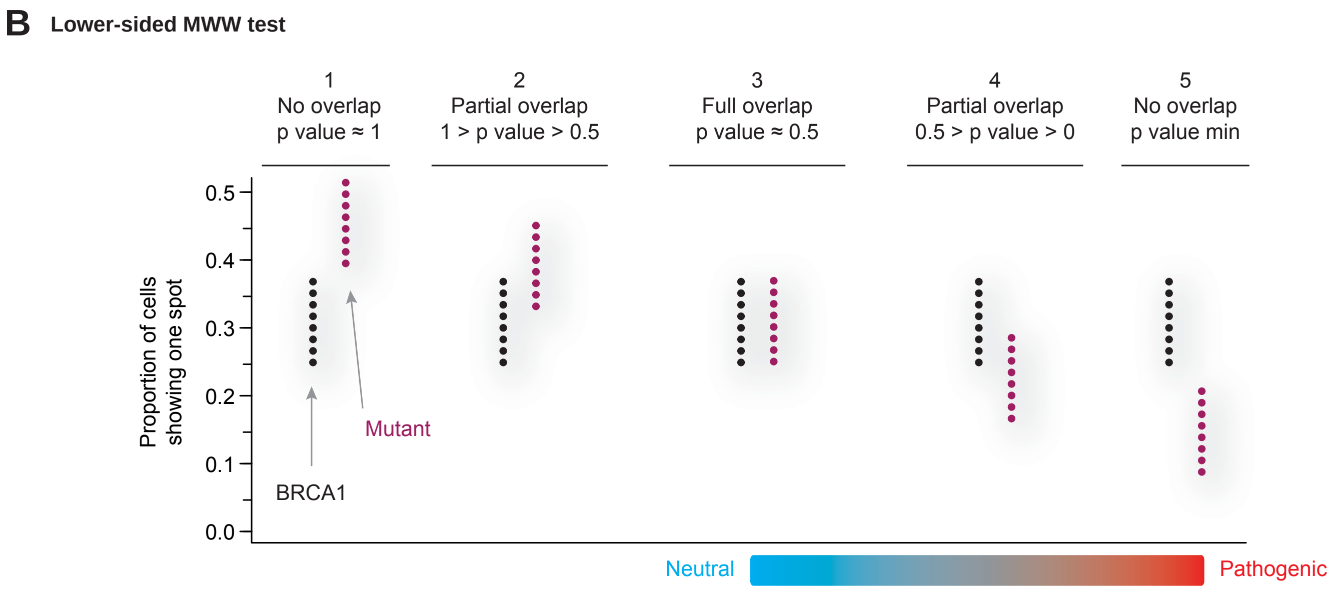

S4 Fig. The MWW method

(**A**) Upper-sided MWW test. The theoretical examples are based on the Colony Size assay but are also valid for the Liquid Medium and Yeast Localization assays. Each distribution of the WT BRCA1 reference (black) and the missense mutation (purple) are composed of 8 theoretical values, represented by 8 dots in the diagram. The p value of the MWW test is used to score the overlap of the mutant and the WT BRCA1 distributions. See the S1 Text for full details. From left to right: (1) when all the mutant values are below the BRCA1 values, the upper-sided MWW test results in a p value close to 1; (2) the p value decreases when the mutant distribution begins to overlap the BRCA1 distribution; (3) the p value is approximately 0.5 when the two distributions completely overlap; (4) the p value continues to decrease when the mutant distribution is above the BRCA1 distribution, with a partial overlap; (5) finally, the p value is lowest when the mutant distribution is fully above the BRCA1 distribution. In theory, neutral and pathogenic mutations should have a p value close to 0.5 and 0, respectively, as depicted by the color scale below the diagram. However, the absolute p value attributed to each variant is not determinant. What is significant is the relative positions between the mutant distributions, indicated by the p values, using the WT BRCA1 distribution as a reference position. The lowest p values represent systematically the pathogenic mutations, and the highest the neutral mutations. Thus, the upper-sided MWW test is used when pathogenic mutations are above the neutral ones in the experimental data.

(**B**) Lower-sided MWW test. All of the theoretical examples shown are based on the Spot Formation assay. As in the upper-sided MWW test, the lowest and highest p values still represent the pathogenic and neutral mutations, respectively, but the pathogenic mutations are below the neutral ones in the experimental data.
