# Supplementary material for: Functional Assessment of Genetic Variants with Outcomes Adapted to Clinical Decision-Making
Source: PLoS Genet. 2016 Jun 6;12(6):e1006096. doi: 10.1371/journal.pgen.1006096 (PMC4894565; doi:10.1371/journal.pgen.1006096)
Supplement: S5 Fig — (A) Colony Size assay. The left panel exhibits dotplot distributions. Boxplots provide distribution parameters: box central bar, median; box, interquartile range (50% of the distribution); whiskers, extreme values. The middle panel shows the normal Quantile-Quantile (QQ) plot of the pathogenic values. Dots forming a straight line suggest that the values are normally distributed. Black line, straight line through the quantiles 25% and 75%. The right panel shows the normal QQ plot of the neutral values. (B) Liquid Medium assay. (C) Spot Formation assay. (D) Yeast Localization assay. (PDF) [file pgen.1006096.s007.pdf]

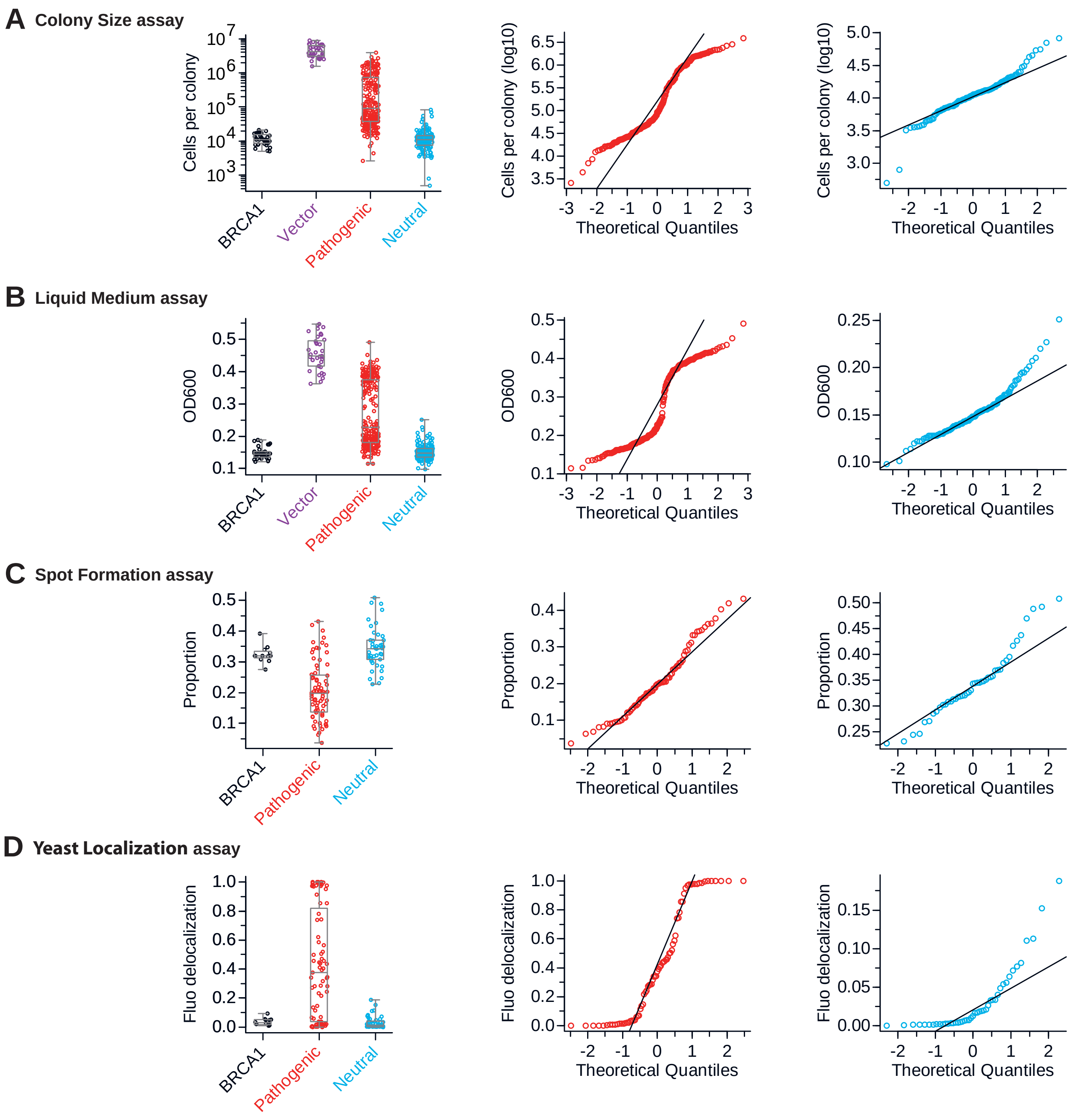

S5 Fig. Distribution of the pathogenic and neutral values

(**A**) Colony Size assay. The left panel exhibits dotplot distributions. Boxplots provide distribution parameters: box central bar, median; box, interquartile range (50% of the distribution); whiskers, extreme values. The middle panel shows the normal Quantile-Quantile (QQ) plot of the pathogenic values. Dots forming a straight line suggest that the values are normally distributed. Black line, straight line through the quantiles 25% and 75%. The right panel shows the normal QQ plot of the neutral values.

(**B**) Liquid Medium assay.

(**C**) Spot Formation assay.

(**D**) Yeast Localization assay.
