# Supplementary material for: Functional Assessment of Genetic Variants with Outcomes Adapted to Clinical Decision-Making
Source: PLoS Genet. 2016 Jun 6;12(6):e1006096. doi: 10.1371/journal.pgen.1006096 (PMC4894565; doi:10.1371/journal.pgen.1006096)
Supplement: S6 Fig — (A) As in Fig 2A. (B) Theoretical example showing how the values from the best cut-off fluctuation, derived from the MWW method, are converted into probabilities of pathogenicity. Top table: best cut-off distribution composed of 10 best cut-off values, resulting from 10 bootstraps (nbootstrap = 10). The probability attributed to each best cut-off value was 1 / nbootstrap. Bottom table: cumulative distribution functions (CDF) generated from the best cut-off distribution. In this table, probabilities of each repeated cut-off value were summed. For instance, the best cut-off value of 0.9 is repeated 4 times in the top table, leading to a probability of 0.4. The CDF represents the sum of the probabilities present in the second row of the bottom table. Three CDF were computed. The first reaches the cumulated probability of 1. The second begins with the cumulated probability of 0. The third is the average of the two first CDF. This average CDF delivers the probability of pathogenicity used to classify variants. Right panel: plot of the average CDF. To classify a variant (e.g., M18T), the variant p value, derived from the MWW method, is positioned on the x-axis (vertical grey bar). Next, the closest average CDF value is attributed to the variant as a probability of pathogenicity. In this example, the best cut-off value, closest to M18T, is 0.5. Thus, the corresponding probability 0.75 is attributed to M18T. (C-E) Average CDF of the Colony Size (CS), Liquid Medium (LM), Spot Formation (SF) and Yeast Localization (YL) assays, obtained with the standard (C), standard with reference (D) or MWW method (E). The same procedure, described in B, was applied to the 2,000 best cut off values obtained for each assay and each method used. The CDF is ascending when the pathogenic mutations are above the neutral ones, and descending when the pathogenic mutations are below. The number of different best cut-off values is indicated (n = 2,000 when no identical best cut-off values within dis [file pgen.1006096.s008.pdf]

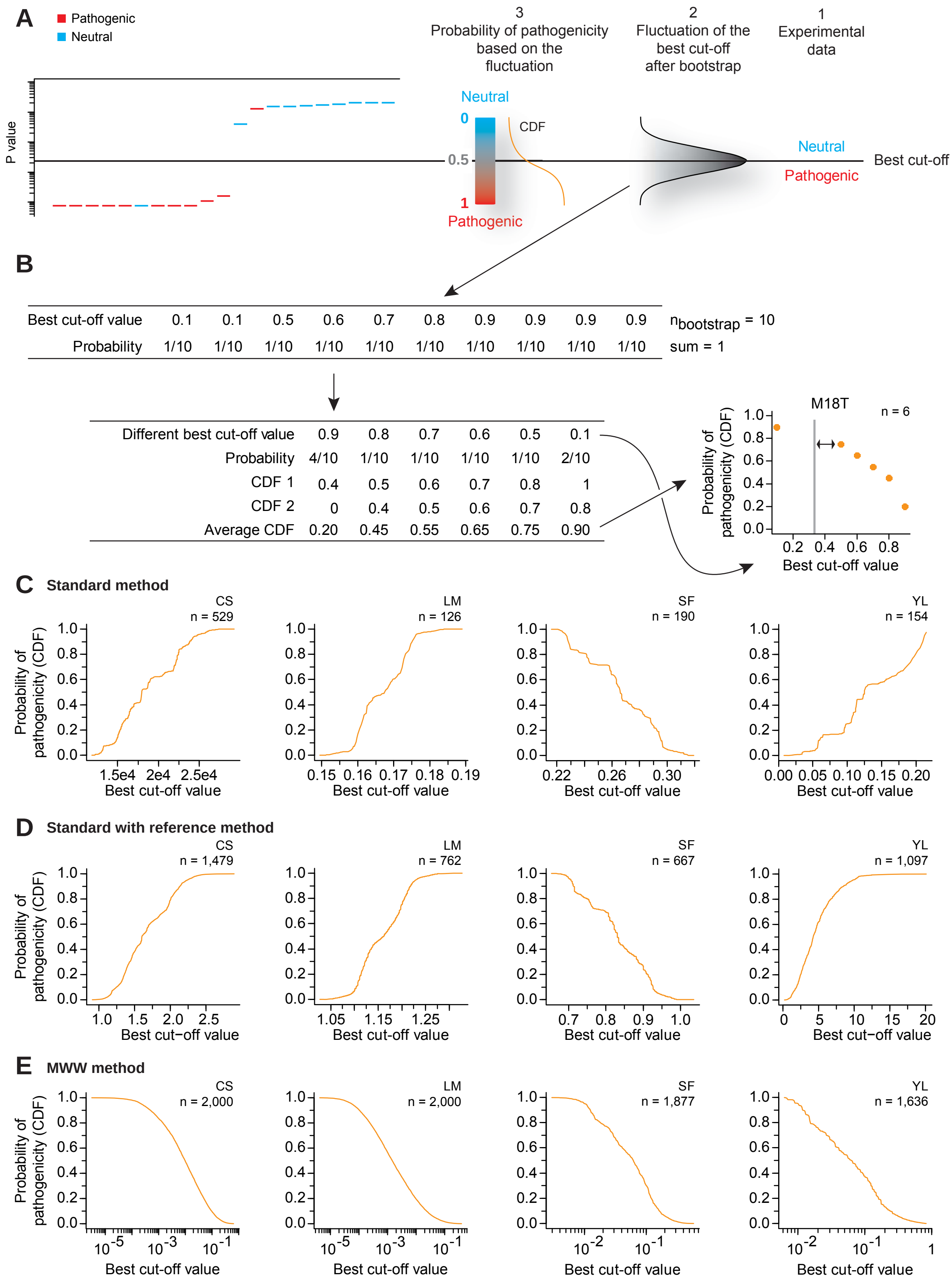

S6 Fig. Description of the probability system of classification

(**A**) As in Fig 2a.

(**B**) Theoretical example showing how the values from the best cut-off fluctuation, derived from the MWW method, are converted into probabilities of pathogenicity. Top table: best cut-off distribution composed of 10 best cut-off values, resulting from 10 bootstraps ($n_{bootstrap}$ = 10). The probability attributed to each best cut-off value was 1 / $n_{bootstrap}$. Bottom table: cumulative distribution functions (CDF) generated from the best cut-off distribution. In this table, probabilities of each repeated cut-off value were summed. For instance, the best cut-off value of 0.9 is repeated 4 times in the top table, leading to a probability of 0.4. The CDF represents the sum of the probabilities present in the second row of the bottom table. Three CDF were computed. The first reaches the cumulated probability of 1. The second begins with the cumulated probability of 0. The third is the average of the two first CDF. This average CDF delivers the probability of pathogenicity used to classify variants. Right panel: plot of the average CDF. To classify a variant (e.g., M18T), the variant p value, derived from the MWW method, is positioned on the x-axis (vertical grey bar). Next, the closest average CDF value is attributed to the variant as a probability of pathogenicity. In this example, the best cut-off value, closest to M18T, is 0.5. Thus, the corresponding probability 0.75 is attributed to M18T.

(**C-E**) Average CDF of the Colony Size (CS), Liquid Medium (LM), Spot Formation (SF) and Yeast Localization (YL) assays, obtained with the standard (**C**), standard with reference (**D**) or MWW method (**E**). The same procedure, described in **B**, was applied to the 2,000 best cut off values obtained for each assay and each method used. The CDF is ascending when the pathogenic mutations are above the neutral ones, and descending when the pathogenic mutations are below. The number of different best cut-off values is indicated (n = 2,000 when no identical best cut-off values within distributions).
