# Supplementary material for: Functional Assessment of Genetic Variants with Outcomes Adapted to Clinical Decision-Making
Source: PLoS Genet. 2016 Jun 6;12(6):e1006096. doi: 10.1371/journal.pgen.1006096 (PMC4894565; doi:10.1371/journal.pgen.1006096)
Supplement: S7 Fig — (A-B) As in Fig 1. One OD unit corresponds to 108 cells / ml. Arrows pinpoint the ranking of the L22S and C47G mutations, which is improved using the MWW method, as explained in the main text introducing this method. The incoherent ranking observed with the standard method results from L22S that exhibits four values below the experimental best cut-off while C47G has none (S8A Fig). (PDF) [file pgen.1006096.s009.pdf]

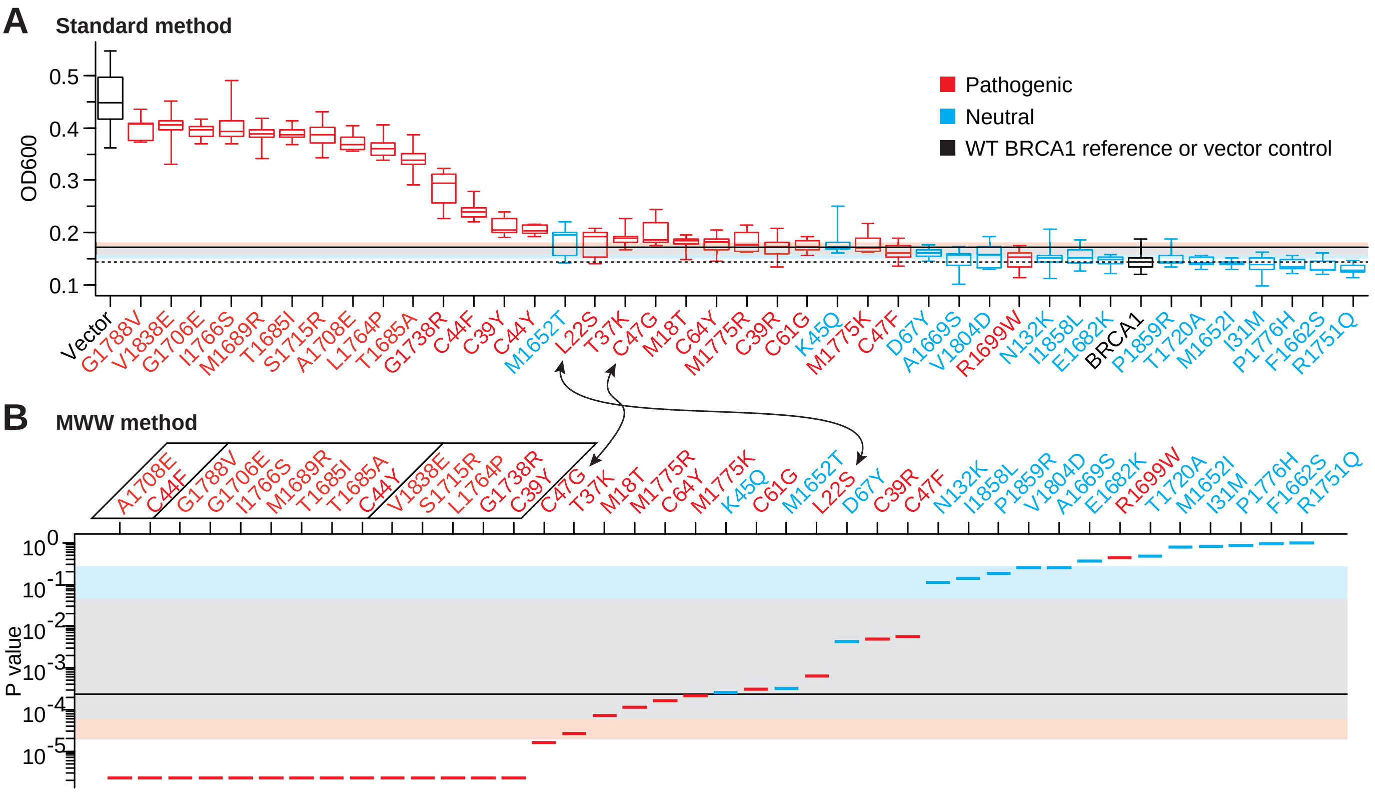

S7 Fig. Relative position of the variants in the Liquid Medium assay and fluctuation of the best cut-off

(**A-B**) As in Fig 1. One OD unit corresponds to 10^8 cells / ml. Arrows pinpoint the ranking of the L22S and C47G mutations, which is improved using the MWW method, as explained in the main text introducing this method. The incoherent ranking observed with the standard method results from L22S that exhibits four values below the experimental best cut-off while C47G has none (S8A Fig).
