# Supplementary material for: Functional Assessment of Genetic Variants with Outcomes Adapted to Clinical Decision-Making
Source: PLoS Genet. 2016 Jun 6;12(6):e1006096. doi: 10.1371/journal.pgen.1006096 (PMC4894565; doi:10.1371/journal.pgen.1006096)
Supplement: S8 Fig — (A-B) Same as for the Colony Size assay (S3 Fig). One OD unit corresponds to 108 cells / ml. (PDF) [file pgen.1006096.s010.pdf]

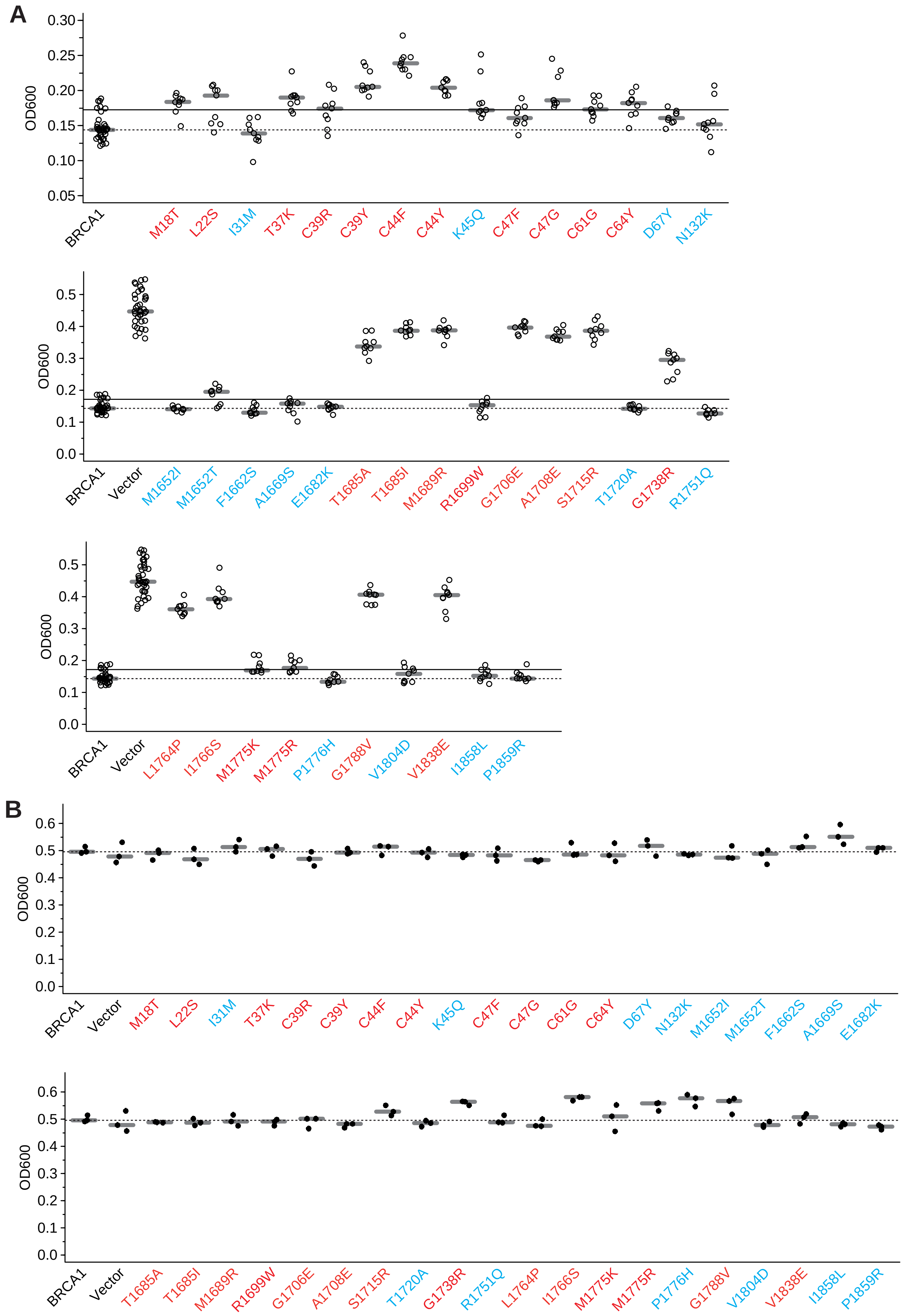

S8 Fig. Supplemental information in the Liquid Medium assay

(**A-B**) Same as for the Colony Size assay (S3 Fig). One OD unit corresponds to 10^8 cells / ml.
