# Supplementary material for: Functional Assessment of Genetic Variants with Outcomes Adapted to Clinical Decision-Making
Source: PLoS Genet. 2016 Jun 6;12(6):e1006096. doi: 10.1371/journal.pgen.1006096 (PMC4894565; doi:10.1371/journal.pgen.1006096)
Supplement: S9 Fig — (A-B) As in Fig 1, except that boxplots and p values resulted from 3 (mutants) or 12 (BRCA1) values. Arrows pinpoint the ranking of the M18T and C39Y mutations, which is improved using the MWW method, as explained in the main text introducing this method. The incoherent ranking observed with the standard method results from M18T that exhibits one value above the experimental best cut-off (shown by the top whisker overlaying the thick horizontal line) while C39Y has none. (PDF) [file pgen.1006096.s011.pdf]

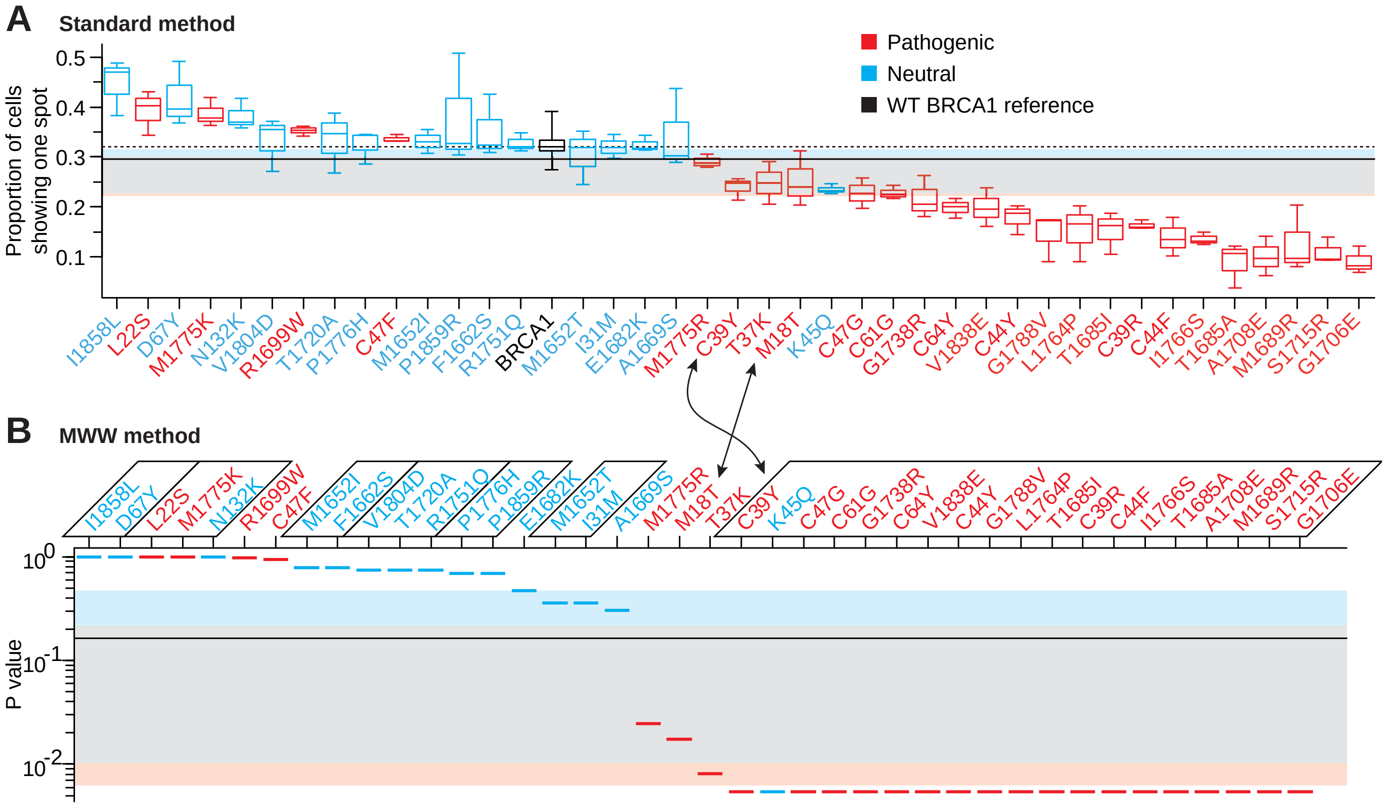

S9 Fig. Relative position of the variants in the Spot Formation assay and fluctuation of the best cut-off

(**A-B**) As in Fig 1, except that boxplots and p values resulted from 3 (mutants) or 12 (BRCA1) values. Arrows pinpoint the ranking of the M18T and C39Y mutations, which is improved using the MWW method, as explained in the main text introducing this method. The incoherent ranking observed with the standard method results from M18T that exhibits one value above the experimental best cut-off (shown by the top whisker overlaying the thick horizontal line) while C39Y has none.
