# Supplementary material for: Functional Assessment of Genetic Variants with Outcomes Adapted to Clinical Decision-Making
Source: PLoS Genet. 2016 Jun 6;12(6):e1006096. doi: 10.1371/journal.pgen.1006096 (PMC4894565; doi:10.1371/journal.pgen.1006096)
Supplement: S10 Fig — Nup133-GFP cells, expressing the WT or mutated BRCA1 protein, fused to mCherry, were analyzed using live fluorescent microscopy. (A) Examples of images acquired. Nup133-GFP allows visualization of the nuclear membrane within the cell, in the green channel. Overlayed images of GFP and mCherry (Merge) as well as transillumination images (Trans) are also shown. Scale bar, 5 μm. (B) Image quantifications. Bars and whiskers indicate median and extreme values for each distribution, respectively. For each assessed clone, the total number of cells showing one spot, two spots, more than two spots, or a diffusive signal, was counted. Three clones were assessed once, for each missense mutation, and 4 times for the WT BRCA1 reference. Thus, each bar in the diagram is the result of 3 values, for each missense mutation, and 12 values for the WT BRCA1 reference. In the Spot Formation assay, only the "1 spot" category is considered. The dotted horizontal line represents the median of BRCA1. (C) Dotplot representation of the 12 BRCA1 values. The equivalent dotplot distribution of each mutant is shown in B, with the 3 values from each mutant represented by the top of the dark grey bar and the two whisker extremities, and also in S9A Fig, where the 3 values correspond to the median bar and the two whisker extremities. (PDF) [file pgen.1006096.s012.pdf]

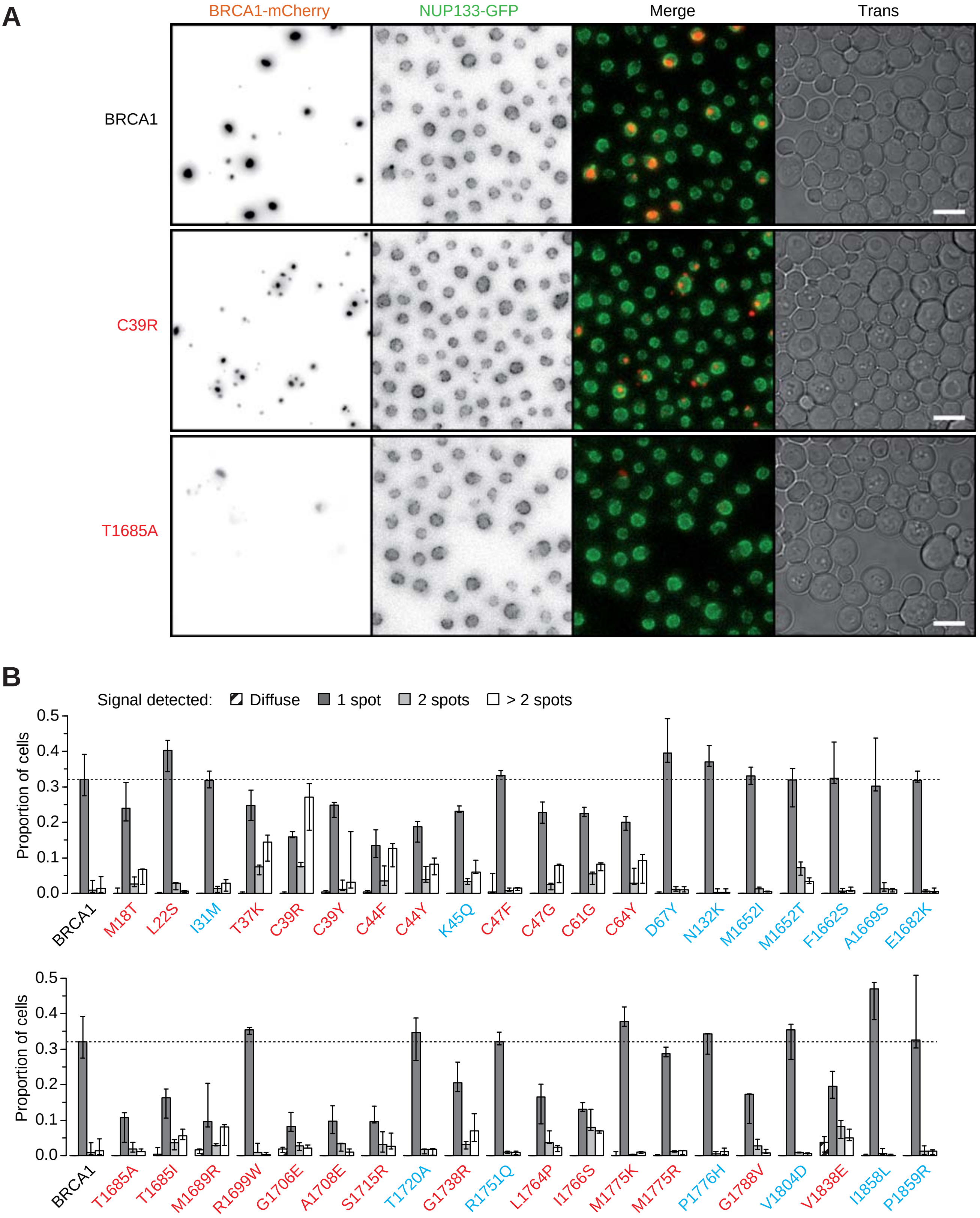

C

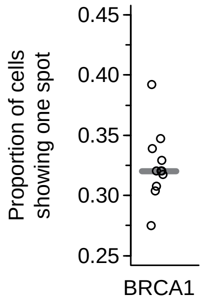

S10 Fig. Supplemental information in the Spot Formation assay

Nup133-GFP cells, expressing the WT or mutated BRCA1 protein, fused to mCherry, were analyzed using live fluorescent microscopy.

(**A**) Examples of images acquired. Nup133-GFP allows visualization of the nuclear membrane within the cell, in the green channel. Overlayed images of GFP and mCherry (Merge) as well as transillumination images (Trans) are also shown. Scale bar, 5 µm.

(**B**) Image quantifications. Bars and whiskers indicate median and extreme values for each distribution, respectively. For each assessed clone, the total number of cells showing one spot, two spots, more than two spots, or a diffusive signal, was counted. Three clones were assessed once, for each missense mutation, and 4 times for the WT BRCA1 reference. Thus, each bar in the diagram is the result of 3 values, for each missense mutation, and 12 values for the WT BRCA1 reference. In the Spot Formation assay, only the "1 spot" category is considered. The dotted horizontal line represents the median of BRCA1.

(**C**) Dotplot representation of the 12 BRCA1 values. The equivalent dotplot distribution of each mutant is shown in **B**, with the 3 values from each mutant represented by the top of the dark grey bar and the two whisker extremities, and also in S9A Fig, where the 3 values correspond to the median bar and the two whisker extremities.
