# Supplementary material for: Functional Assessment of Genetic Variants with Outcomes Adapted to Clinical Decision-Making
Source: PLoS Genet. 2016 Jun 6;12(6):e1006096. doi: 10.1371/journal.pgen.1006096 (PMC4894565; doi:10.1371/journal.pgen.1006096)
Supplement: S11 Fig — (A-B) As in Fig 1, except that the boxplots and p values are the results of 3 (mutants) or 12 (BRCA1) values. Delocalization of the mCherry fluorescent signal from the nucleus ranges from 0 (no cytoplasmic delocalization) to 1 (full cytoplasmic delocalization). Arrows pinpoint the ranking of the A1669S and D67Y mutations, which is improved using the MWW method, as explained in the main text introducing this method. The incoherent ranking observed with the standard method results from A1669S that exhibits one value above the experimental best cut-off while D67Y has none. (C) Dotplot representation of the 12 BRCA1 values forming the BRCA1 boxplot in A. The equivalent dotplot distribution of each mutant is shown in A, with the 3 values from each mutant represented by the median bar and the two whisker extremities. (PDF) [file pgen.1006096.s013.pdf]

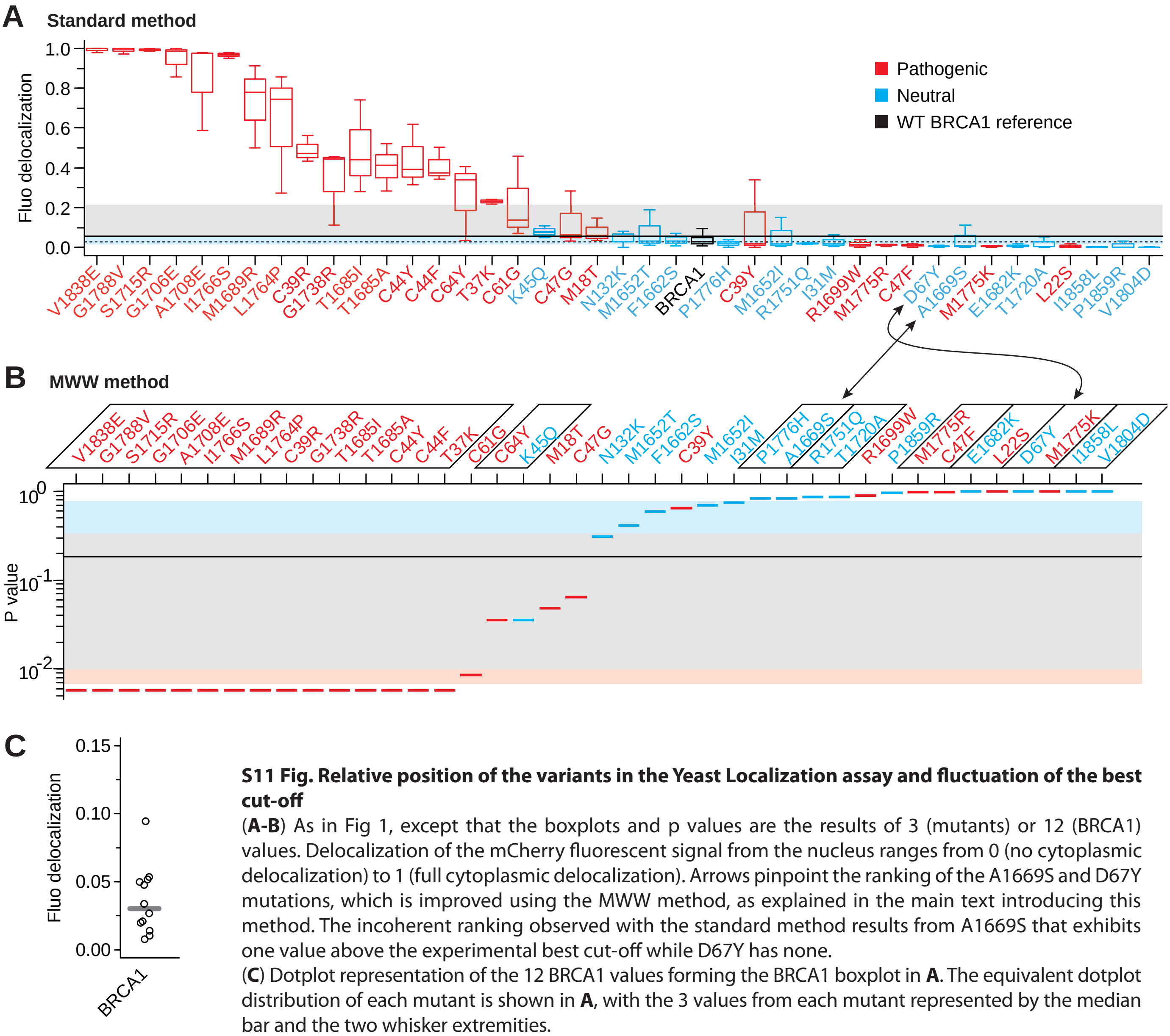

S11 Fig. Relative position of the variants in the Yeast Localization assay and fluctuation of the best cut-off

(**A-B**) As in Fig 1, except that the boxplots and p values are the results of 3 (mutants) or 12 (BRCA1) values. Delocalization of the mCherry fluorescent signal from the nucleus ranges from 0 (no cytoplasmic delocalization) to 1 (full cytoplasmic delocalization). Arrows pinpoint the ranking of the A1669S and D67Y mutations, which is improved using the MWW method, as explained in the main text introducing this method. The incoherent ranking observed with the standard method results from A1669S that exhibits one value above the experimental best cut-off while D67Y has none.

(**C**) Dotplot representation of the 12 BRCA1 values forming the BRCA1 boxplot in **A**. The equivalent dotplot distribution of each mutant is shown in **A**, with the 3 values from each mutant represented by the median bar and the two whisker extremities.
