# Supplementary material for: Functional Assessment of Genetic Variants with Outcomes Adapted to Clinical Decision-Making
Source: PLoS Genet. 2016 Jun 6;12(6):e1006096. doi: 10.1371/journal.pgen.1006096 (PMC4894565; doi:10.1371/journal.pgen.1006096)
Supplement: S12 Fig — Fluorescent images acquired in the Yeast Localization assay, as in S10A Fig. The arrow points to rare cytoplasmic spot in cells expressing the WT BRCA1-mCherry protein. Scale bar, 2 μm. (PDF) [file pgen.1006096.s014.pdf]

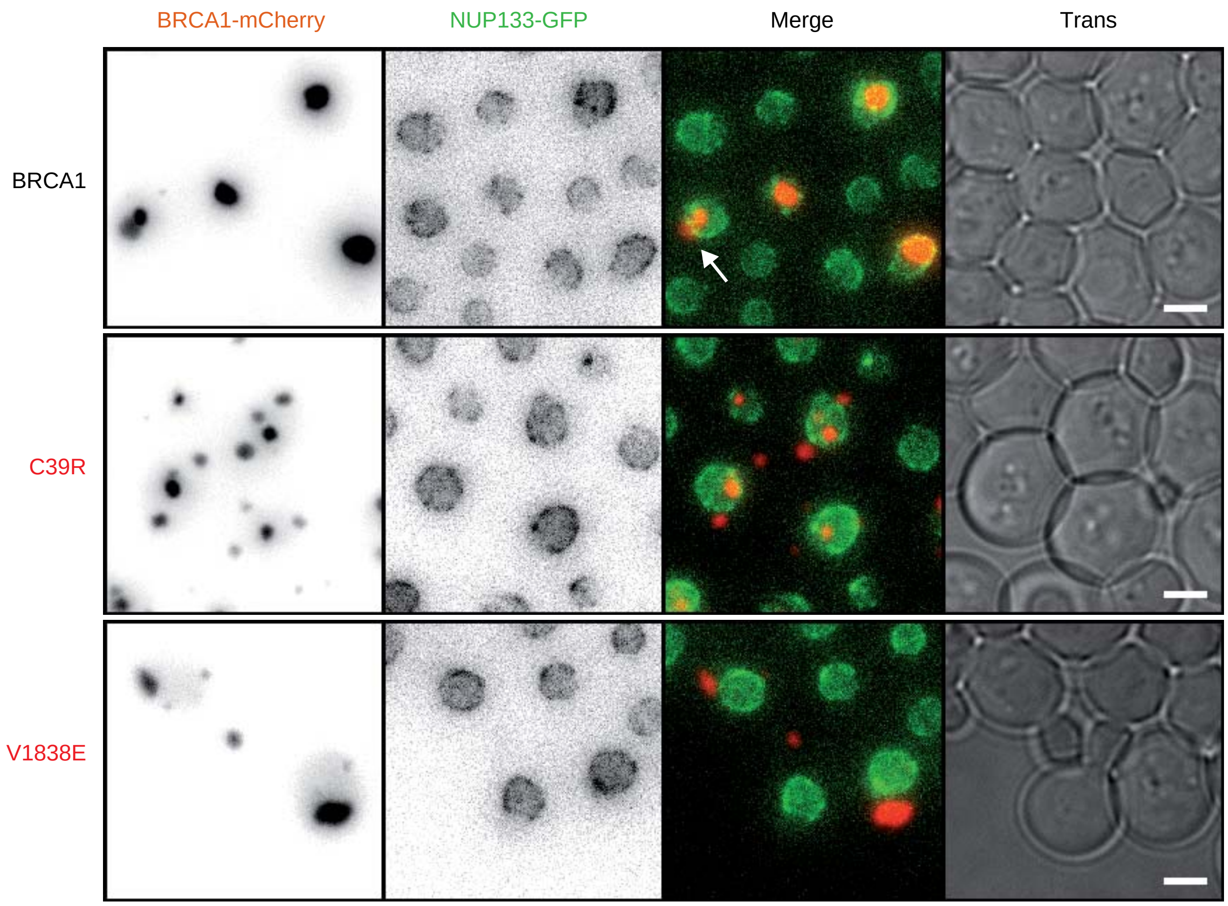

S12 Fig. Supplemental information in the Yeast Localization assay
Fluorescent images acquired in the Yeast Localization assay, as in S10A Fig. The arrow points to rare cytoplasmic spot in cells expressing the WT BRCA1-mCherry protein. Scale bar, 2 µm.
