# Supplementary material for: Functional Assessment of Genetic Variants with Outcomes Adapted to Clinical Decision-Making
Source: PLoS Genet. 2016 Jun 6;12(6):e1006096. doi: 10.1371/journal.pgen.1006096 (PMC4894565; doi:10.1371/journal.pgen.1006096)
Supplement: S13 Fig — The parameters of the theoretical distributions used are detailed in S8 Table. The reference situation is as follows: nmutant = 9, nBRCA1 = 36, nneutral = 15 and npathogenic = 25. In addition, medians and ranges of the neutral and WT BRCA1 distributions were made systematically equal. Distributions of the neutral and pathogenic mutations were identical, except for the shift of the pathogenic values from the neutral mutations, according to the formula vij + 36 × s, with s representing the shift intensity and vij representing the value i of the pathogenic mutation j. When s = 0, pathogenic and neutral distributions are identical. Fluctuations from the best cut-off were obtained exactly as performed for the Colony Size, Liquid Medium, Spot Formation and Yeast Localization assays. (A-C) Examples of shift intensities and best cut-off fluctuation results. The graphs depicted are similar to those in Fig 1, except that the standard, standard with reference and MWW methods are shown respectively on the left, middle and right of the figure. In the standard and standard with reference methods, boxplots are replaced by dotplots with the median of the distributions indicated by a grey segment. The s values are indicated (top left). In the subsequent supplemental figures, the position of the pathogenic mutation medians are as in C (s = 2). The grey horizontal line indicates the median of the best cut-off fluctuation. (D) Probabilities of pathogenicity obtained for the neutral (blue line) and pathogenic variants (red line), depending on the shift intensity of the pathogenic mutations. Y-axis, log10(p / (1—p)) with p being the probability of pathogenicity of the variants (0 corresponds to p = 0.5); right colored classes, five-class nomenclature with the horizontal grey lines showing the 0.99, 0.95, 0.05 and 0.001 limits of the classes (see S1 Table). In the standard method, the slight erratic curves and the lack of specificity sometimes observed (blue line in the class 2 instead of [file pgen.1006096.s015.pdf]

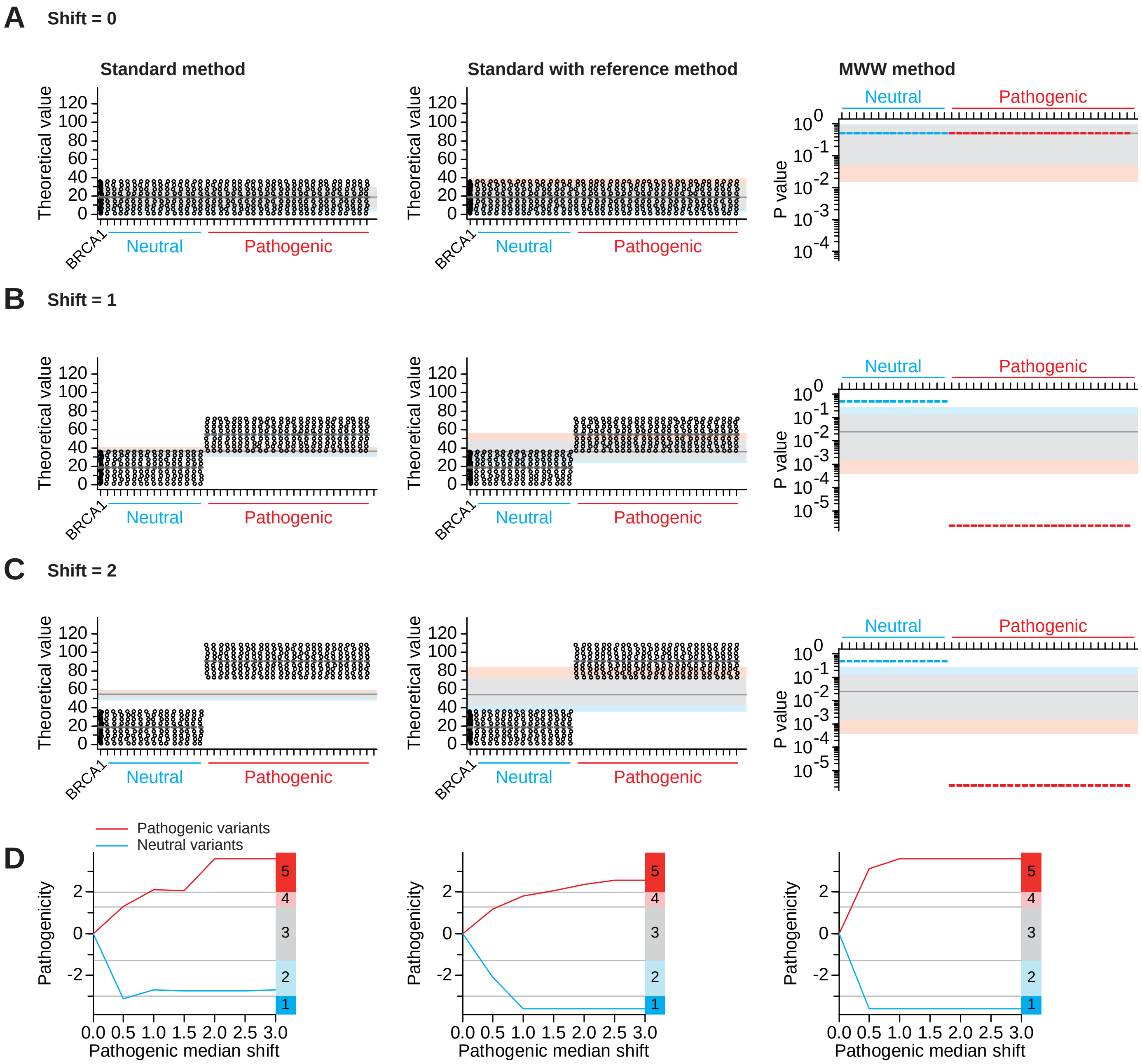


**S13 Fig. Effect of the position of the pathogenic mutations on the probability system of classification (theoretical situation)**
The parameters of the theoretical distributions used are detailed in Table S8. The reference situation is as follows: $n_{mutant} = 9$, $n_{BRCA1} = 36$, $n_{neutral} = 15$ and $n_{pathogenic} = 25$. In addition, medians and ranges of the neutral and WT BRCA1 distributions were made systematically equal. Distributions of the neutral and pathogenic mutations were identical, except for the shift of the pathogenic values from the neutral mutations, according to the formula $v_{ij} + 36 \times s$, with s representing the shift intensity and $v_{ij}$ representing the value i of the pathogenic mutation j. When s = 0, pathogenic and neutral distributions are identical. Fluctuations from the best cut-off were obtained exactly as performed for the Colony Size, Liquid Medium, Spot Formation and Yeast Localization assays.
(**A-C**) Examples of shift intensities and best cut-off fluctuation results. The graphs depicted are similar to those in Fig 1, except that the standard, standard with reference and MWW methods are shown respectively on the left, middle and right of the figure. In the standard and standard with reference methods, boxplots are replaced by dotplots with the median of the distributions indicated by a grey segment. The s values are indicated (top left). In the subsequent supplemental figures, the position of the pathogenic mutation medians are as in **C** (s = 2). The grey horizontal line indicates the median of the best cut-off fluctuation.
(**D**) Probabilities of pathogenicity obtained for the neutral (blue line) and pathogenic variants (red line), depending on the shift intensity of the pathogenic mutations. Y-axis, log10(p / (1 - p)) with p being the probability of pathogenicity of the variants (0 corresponds to p = 0.5); right colored classes, five-class nomenclature with the horizontal grey lines showing the 0.99, 0.95, 0.05 and 0.001 limits of the classes (see S1 Table). In the standard method, the slight erratic curves and the lack of specificity sometimes observed (blue line in the class 2 instead of class 1) is due to the fact that this method generates a low number of different best cut-off values (between 8 and 64) in the best cut-off distributions, as explained in the S1 Text.
As summarized in S9 Table, these results confirm that the probability system of classification is an efficient variant classifier. Indeed, whatever method is used, when the pathogenic and neutral distributions are strictly identical, they all locate inside the class 3 area (i.e., the system cannot classify any variants in such kind of functional assay). Moreover, the probability system of classification is improved when the pathogenic mutations shift from the neutral sector towards the pathogenic sector, since the probability of pathogenicity increases for the pathogenic variants and decreases for the neutral ones.
