# Supplementary material for: Functional Assessment of Genetic Variants with Outcomes Adapted to Clinical Decision-Making
Source: PLoS Genet. 2016 Jun 6;12(6):e1006096. doi: 10.1371/journal.pgen.1006096 (PMC4894565; doi:10.1371/journal.pgen.1006096)
Supplement: S14 Fig — See S13 Fig for details. Neutral mutations were shifted according to the formula vij + 36 × s, with s representing the shift intensity and vij representing the value i of the neutral mutation j (when s = 0, medians and extreme values of the BRCA1 and neutral distributions are identical. When s = 2, pathogenic and neutral distributions are identical). (A-D) Examples of shift intensities and best cut-off fluctuation results. The s values are indicated (top left). (E) Probabilities of pathogenicity obtained for the neutral (blue line) and pathogenic variants (red line), depending on the shift intensity of the neutral mutations. As summarized in S9 Table, these results highlight divergences between the different methods. With the standard method and the standard with reference methods (E, left and middle panels), sensitivity and specificity of the probability system of classification decrease when the neutral mutations approach the pathogenic mutations. With the MWW method (E, right panel), the probability system of classification results in a complete misclassification of the pathogenic mutations when the neutral distributions do not overlap the WT reference distribution (s ≥ 1). Of note, these analyses treat extreme situations. In practice, the WT reference should be well embedded within the neutral distributions. The opposite situation would raise question about the WT reference or neutral mutations used. (PDF) [file pgen.1006096.s016.pdf]

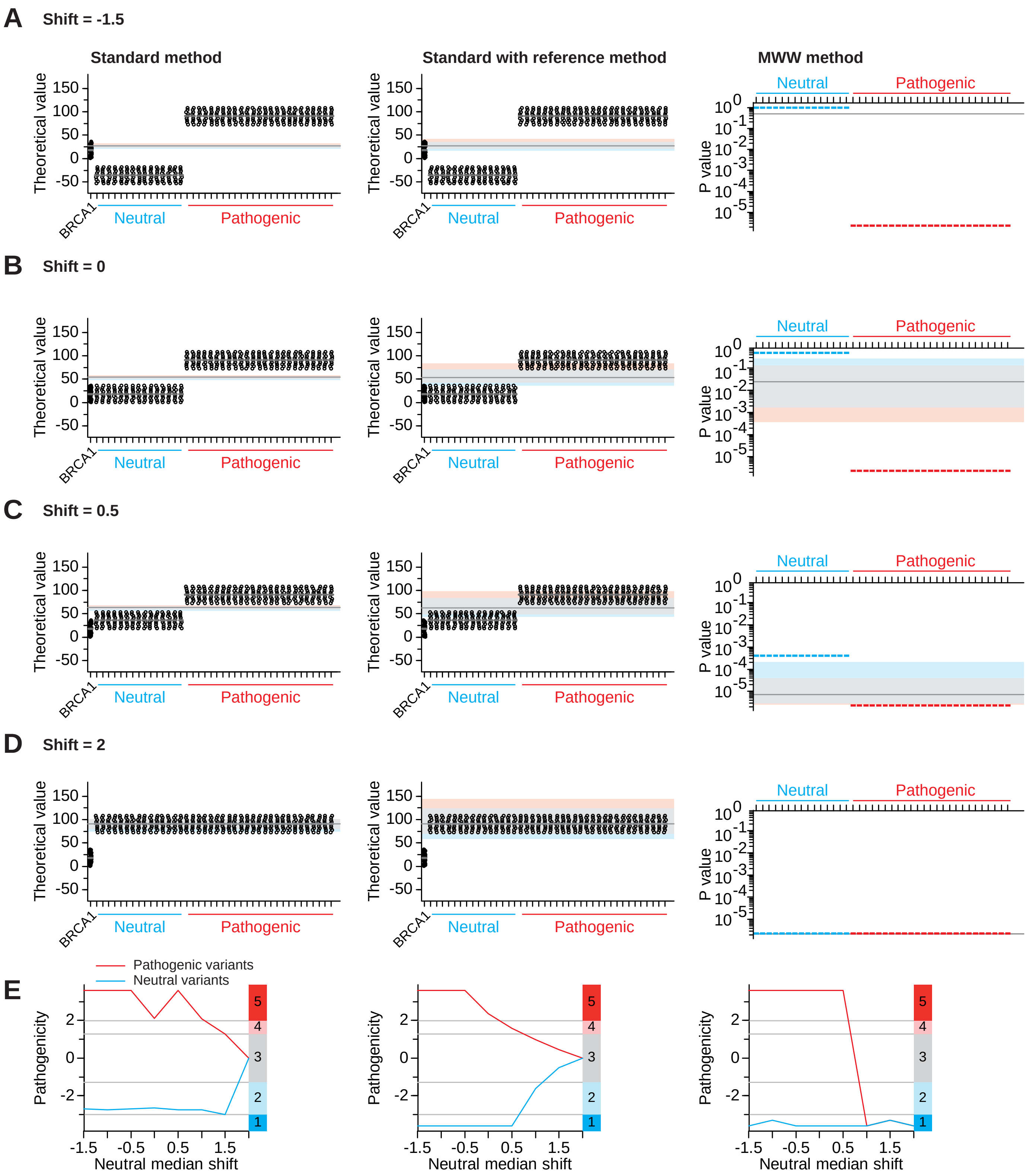

S14 Fig. Effect of the position of the neutral mutations on the probability system of classification (theoretical situation)

See S13 Fig for details. Neutral mutations were shifted according to the formula $v_{ij} + 36 \times s$, with s representing the shift intensity and v_{ij} representing the value i of the neutral mutation j (when s = 0, medians and extreme values of the BRCA1 and neutral distributions are identical. When s = 2, pathogenic and neutral distributions are identical).

(**A-D**) Examples of shift intensities and best cut-off fluctuation results. The s values are indicated (top left).

(**E**) Probabilities of pathogenicity obtained for the neutral (blue line) and pathogenic variants (red line), depending on the shift intensity of the neutral mutations.

As summarized in S9 Table, these results highlight divergences between the different methods. With the standard method and the standard with reference methods (**E**, left and middle panels), sensitivity and specificity of the probability system of classification decrease when the neutral mutations approach the pathogenic mutations. With the MWW method (**E**, right panel), the probability system of classification results in a complete misclassification of the pathogenic mutations when the neutral distributions do not overlap the WT reference distribution ($s \geq 1$). Of note, these analyses treat extreme situations. In practice, the WT reference should be well embedded within the neutral distributions. The opposite situation would raise question about the WT reference or neutral mutations used.
