# Supplementary material for: Functional Assessment of Genetic Variants with Outcomes Adapted to Clinical Decision-Making
Source: PLoS Genet. 2016 Jun 6;12(6):e1006096. doi: 10.1371/journal.pgen.1006096 (PMC4894565; doi:10.1371/journal.pgen.1006096)
Supplement: S15 Fig — See S13 Fig for details. Values of the WT BRCA1 distribution were shifted according to the formula vi + 36 × s, with s representing the shift intensity and vi representing the value i of the BRCA1 reference (when s = 0, medians and extreme values of the neutral and BRCA1 distributions are identical. When s = 2, medians and extreme values of the pathogenic and BRCA1 distributions are identical). Of note, these theoretical analyses treat extreme situations. In practice, the WT reference should be well embedded in the neutral distributions. The opposite situation would raise question about the WT reference or neutral mutations used. (A-D) Examples of shift intensities and best cut-off fluctuation results. The s values are indicated (top left). (E) Probabilities of pathogenicity obtained for the neutral (blue line) and pathogenic variants (red line), depending on the shift intensity of the WT reference. As summarized in S9 Table, these results highlight divergences between the different methods. As expected, the standard method is not affected by the position of the WT BRCA1 distribution (E, left panel). In contrast, the standard with reference method is strongly influenced by the position of this reference (E, middle panel). When the WT BRCA1 median shifts towards the null value, sensitivity and specificity of the probability system of classification are decreased, with a complete loss of sensitivity and specificity (i.e., systematic classification as class 3) when the WT BRCA1 median is null (s ≈ -0.514). This was expected since the standard with reference method is based on best cut-off values divided by the WT BRCA1 median. Thus, a division by zero generates relative best cut-offs with an infinite value. Such issues are compensated only when best cut-offs are close to the WT BRCA1 median. This was shown in the Liquid Medium and Yeast Localization assays. Using the standard or standard with reference method provided similar variant classification (Fig 2B), even if th [file pgen.1006096.s017.pdf]

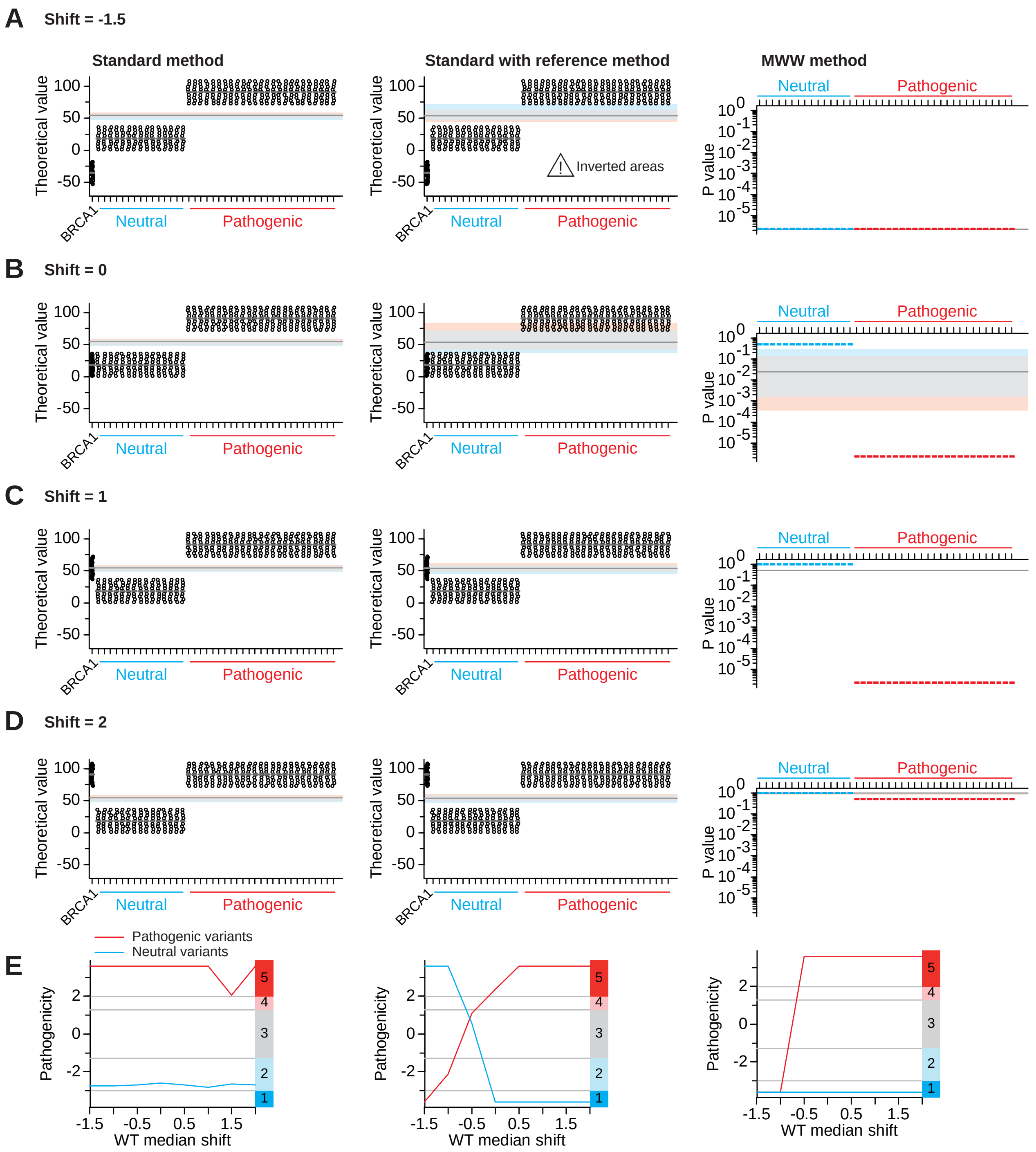

S15 Fig. Effect of the position of the WT BRCA1 reference on the probability system of classification (theoretical situation)
See S13 Fig for details. Values of the WT BRCA1 distribution were shifted according to the formula $v_i + 36 \times s$, with s representing the shift intensity and v_i representing the value i of the BRCA1 reference (when s = 0, medians and extreme values of the neutral and BRCA1 distributions are identical. When s = 2, medians and extreme values of the pathogenic and BRCA1 distributions are identical). Of note, these theoretical analyses treat extreme situations. In practice, the WT reference should be well embedded in the neutral distributions. The opposite situation would raise question about the WT reference or neutral mutations used.
(**A-D**) Examples of shift intensities and best cut-off fluctuation results. The s values are indicated (top left).
(**E**) Probabilities of pathogenicity obtained for the neutral (blue line) and pathogenic variants (red line), depending on the shift intensity of the WT reference.
As summarized in S9 Table, these results highlight divergences between the different methods. As expected, the standard method is not affected by the position of the WT BRCA1 distribution (**E**, left panel). In contrast, the standard with reference method is strongly influenced by the position of this reference (**E**, middle panel). When the WT BRCA1 median shifts towards the null value, sensitivity and specificity of the probability system of classification are decreased, with a complete loss of sensitivity and specificity (i.e., systematic classification as class 3) when the WT BRCA1 median is null ($s \approx -0.514$). This was expected since the standard with reference method is based on best cut-off values divided by the WT BRCA1 median. Thus, a division by zero generates relative best cut-offs with an infinite value. Such issues are compensated only when best cut-offs are close to the WT BRCA1 median. This was shown in the Liquid Medium and Yeast Localization assays. Using the standard or standard with reference method provided similar variant classification (Fig 2B), even if the WT BRCA1 medians of these assays approached zero, with 0.144 and 0.03 respectively (S4 Table). In conclusion, a situation, in which the WT reference median is close to zero, with the fluctuation of the raw best cut-off far from this median, will guarantee a weak sensitivity and specificity of the probability system of classification. Concerning the standard with reference method, it is also noteworthy that a negative value of the WT reference median ($s < -0.514$) inverts the classification (**E**, middle panel), as expected, regardless of the values from the neutral and pathogenic mutations. When comparing the standard with reference method versus the MWW method, the later has the advantage of being independent of the WT reference values, as only overlapping distributions matter. Specificity of the probability system of classification is not affected by the position of the WT reference, contrary to sensitivity (**E**, right panel). The main weakness of the MWW method occurs when the WT reference distribution falls outside of the range of the neutral and pathogenic distributions (as in **A**, left panel), which generates misclassification of the pathogenic mutations as neutral.
