# Supplementary material for: Functional Assessment of Genetic Variants with Outcomes Adapted to Clinical Decision-Making
Source: PLoS Genet. 2016 Jun 6;12(6):e1006096. doi: 10.1371/journal.pgen.1006096 (PMC4894565; doi:10.1371/journal.pgen.1006096)
Supplement: S16 Fig — See S13 Fig for details. The experimental sensitivity and specificity were modulated by assigning certain pathogenic mutants in the neutral region and certain neutral mutants in the pathogenic region, respectively. The experimental sensitivity and specificity values indicated were those obtained with the experimental best cut-off, as explained in S2 Fig. These values are referred to as "initial" sensitivity and specificity, as opposed to the sensitivity and specificity of the probability system of classification, obtained after bootstrap analysis. (A-D) Examples of experimental sensitivities/specificities and best cut-off fluctuation results. (E-G) Probabilities of pathogenicity obtained for the neutral (blue line) and pathogenic variants (red line), depending on decreases from experimental specificity (E), experimental sensitivity (F) or both (G). As summarized in S9 Table, these results confirm that the probability system of classification is an efficient variant classifier. A decrease of the experimental specificity indicates that the pathogenic area is contaminated by neutral variants, which reduces the probability of pathogenicity of the pathogenic variants (class 5 towards class 3). In the same manner, a decrease of the experimental sensitivity indicates that the neutral area is contaminated by pathogenic variants, which enhances the probability of pathogenicity of the neutral variants (class 1 towards class 3). This was observed using the three standard, standard with reference and MWW methods. Of note, the situations studied used systematically: experimental sensitivity + experimental specificity ≥ 1 (otherwise representing an inappropriate use of the experimental information, i.e., pathogenic and neutral sectors incorrectly positioned). (PDF) [file pgen.1006096.s018.pdf]

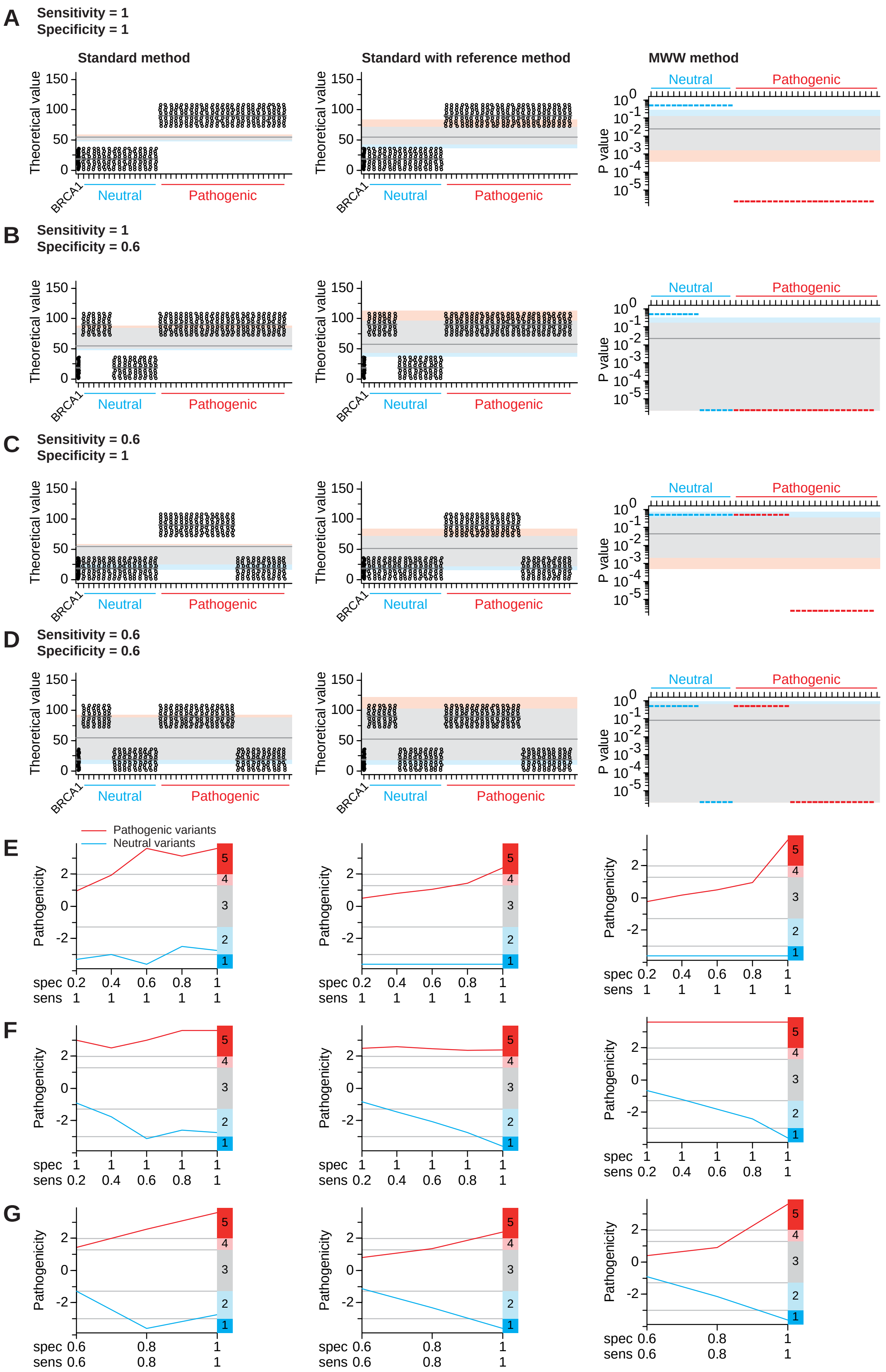

S16 Fig. Effect of the experimental sensitivity and specificity on the probability system of classification (theoretical situation)
See S13 Fig for details. The experimental sensitivity and specificity were modulated by assigning certain pathogenic mutants in the neutral region and certain neutral mutants in the pathogenic region, respectively. The experimental sensitivity and specificity values indicated were those obtained with the experimental best cut-off, as explained in S2 Fig. These values are referred to as "initial" sensitivity and specificity, as opposed to the sensitivity and specificity of the probability system of classification, obtained after bootstrap analysis.
(**A-D**) Examples of experimental sensitivities/specificities and best cut-off fluctuation results.
(**E-G**) Probabilities of pathogenicity obtained for the neutral (blue line) and pathogenic variants (red line), depending on decreases from experimental specificity (**E**), experimental sensitivity (**F**) or both (**G**).
As summarized in S9 Table, these results confirm that the probability system of classification is an efficient variant classifier. A decrease of the experimental specificity indicates that the pathogenic area is contaminated by neutral variants, which reduces the probability of pathogenicity of the pathogenic variants (class 5 towards class 3). In the same manner, a decrease of the experimental sensitivity indicates that the neutral area is contaminated by pathogenic variants, which enhances the probability of pathogenicity of the neutral variants (class 1 towards class 3). This was observed using the three standard, standard with reference and MWW methods. Of note, the situations studied used systematically: experimental sensitivity + experimental specificity ≥ 1 (otherwise representing an inappropriate use of the experimental information, i.e., pathogenic and neutral sectors incorrectly positioned).
