# Supplementary material for: Functional Assessment of Genetic Variants with Outcomes Adapted to Clinical Decision-Making
Source: PLoS Genet. 2016 Jun 6;12(6):e1006096. doi: 10.1371/journal.pgen.1006096 (PMC4894565; doi:10.1371/journal.pgen.1006096)
Supplement: S17 Fig — See S13 Fig for details. (A-D) Examples showing the number of neutral and pathogenic mutations tested, with best cut-off fluctuation results. (E-G) Probabilities of pathogenicity obtained for the neutral (blue line) and pathogenic variants (red line), following a decrease in the number of neutral mutations (E), pathogenic mutations (F) or both (G). As summarized in S9 Table, these results show that the probability system is poorly sensitive to the number of neutral and pathogenic mutations incorporated, whatever method is used. (PDF) [file pgen.1006096.s019.pdf]

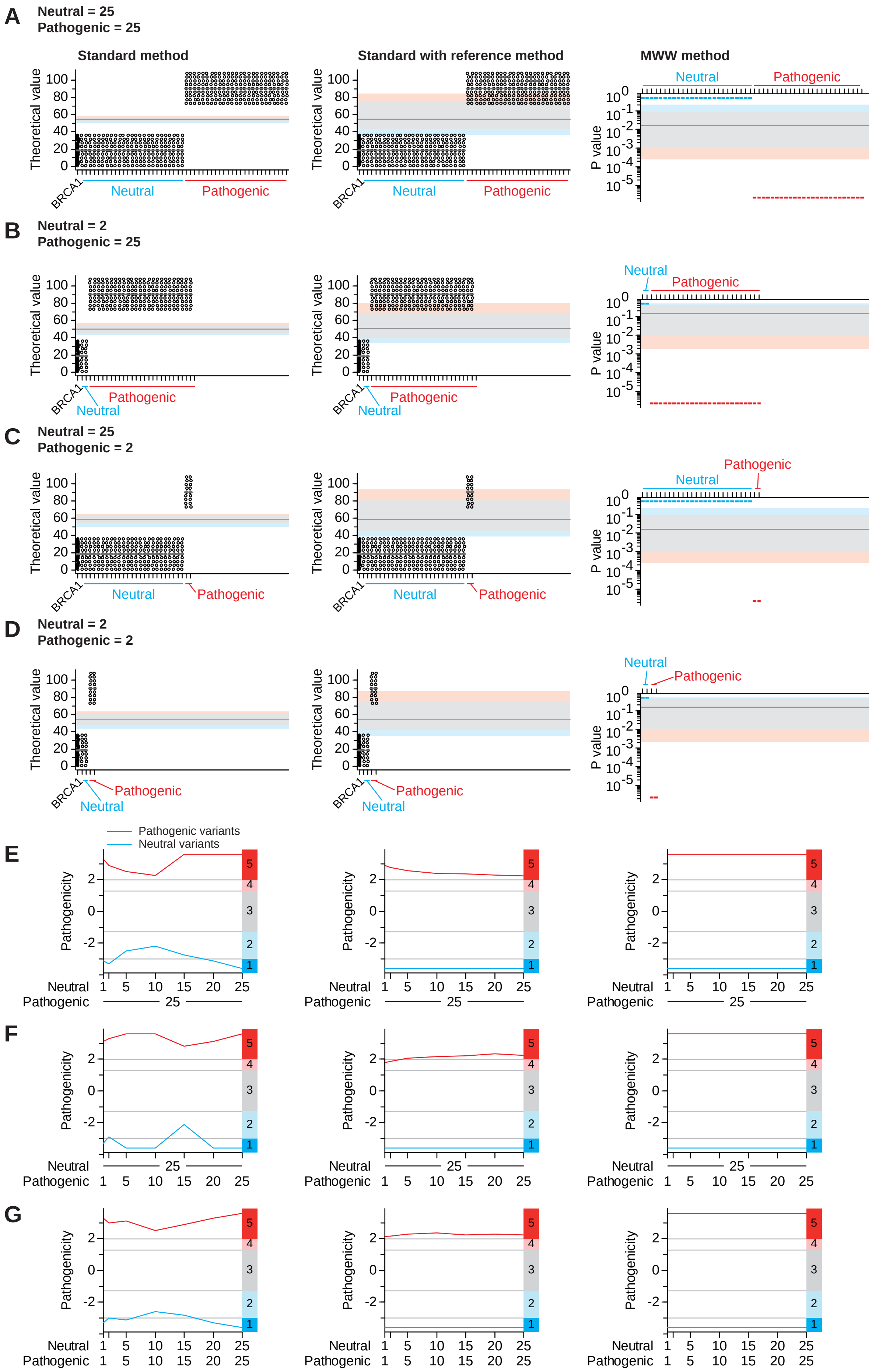

S17 Fig. Effect of the number of neutral and pathogenic mutations on the probability system of classification (theoretical situation)

See S13 Fig for details.

(**A-D**) Examples showing the number of neutral and pathogenic mutations tested, with best cut-off fluctuation results.

(**E-G**) Probabilities of pathogenicity obtained for the neutral (blue line) and pathogenic variants (red line), following a decrease in the number of neutral mutations (**E**), pathogenic mutations (**F**) or both (**G**).

As summarized in S9 Table, these results show that the probability system is poorly sensitive to the number of neutral and pathogenic mutations incorporated, whatever method is used.
