# Supplementary material for: Functional Assessment of Genetic Variants with Outcomes Adapted to Clinical Decision-Making
Source: PLoS Genet. 2016 Jun 6;12(6):e1006096. doi: 10.1371/journal.pgen.1006096 (PMC4894565; doi:10.1371/journal.pgen.1006096)
Supplement: S18 Fig — See S13 Fig for details. Number of values was modulated so that the range and median of the distributions remained the same, as shown in S8 Table. (A-D) Examples showing the number of mutant or BRCA1 values tested, with best cut-off fluctuation results. (E-G) Probabilities of pathogenicity obtained for the neutral (blue line) and pathogenic variants (red line), following a decrease in the number of mutant values (E), BRCA1 values (F), or both (G). As summarized in S9 Table, these results confirm that the probability system of classification is an efficient variant classifier. Whatever method is used, a decreasing number of values in the dataset affects the probabilities of both the pathogenic and neutral variants (G), which tend toward 0.5 (class 3). Thus, the probability system prevents decision-making when data is lacking. As expected, the standard method is not affected by the number of BRCA1 values (F, left panel). The standard with reference and the MWW methods are insensitive to the number of mutant values if the number of BRCA1 values is high (E, middle and right panels). However, a decrease in the number of BRCA1 values lowers the probability of pathogenicity of the pathogenic variants (F, middle and right panels), but with a strong recovery when the fluctuation of the best cut-off is no longer influenced by the fluctuation of the WT reference (nBRCA1 = 1). Of note, the best cut-off does not fluctuate when nmutant = 1 and nBRCA1 = 1 (G), which results in a probability of pathogenicity equal to 0.5 for both the pathogenic and neutral variants. Moreover, using the standard method, when nmutant = 9, the classification of the neutral mutations is class 2 (E, left panel), which explains the lack of specificity frequently observed in S13–S19 Figs. (PDF) [file pgen.1006096.s020.pdf]

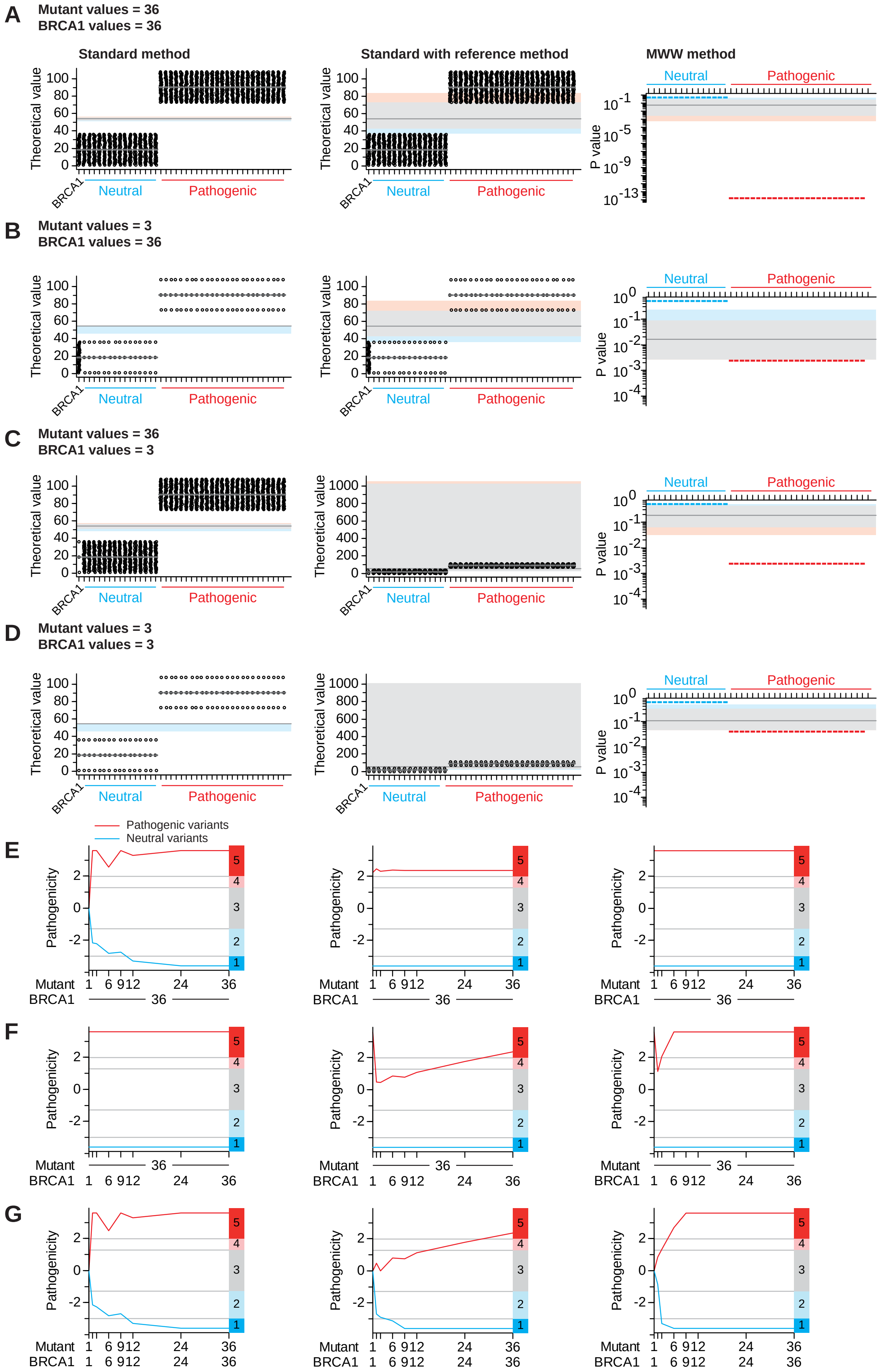

S18 Fig. Effect of the number of mutant and BRCA1 values on the probability system of classification (theoretical situation)

See S13 Fig for details. Number of values was modulated so that the range and median of the distributions remained the same, as shown in S8 Table.

(**A-D**) Examples showing the number of mutant or BRCA1 values tested, with best cut-off fluctuation results.

(**E-G**) Probabilities of pathogenicity obtained for the neutral (blue line) and pathogenic variants (red line), following a decrease in the number of mutant values (**E**), BRCA1 values (**F**), or both (**G**).

As summarized in S9 Table, these results confirm that the probability system of classification is an efficient variant classifier. Whatever method is used, a decreasing number of values in the dataset affects the probabilities of both the pathogenic and neutral variants (**G**), which tend toward 0.5 (class 3). Thus, the probability system prevents decision-making when data is lacking. As expected, the standard method is not affected by the number of BRCA1 values (**F**, left panel). The standard with reference and the MWW methods are insensitive to the number of mutant values if the number of BRCA1 values is high (**E**, middle and right panels). However, a decrease in the number of BRCA1 values lowers the probability of pathogenicity of the pathogenic variants (**F**, middle and right panels), but with a strong recovery when the fluctuation of the best cut-off is no longer influenced by the fluctuation of the WT reference (n_{BRCA1} = 1). Of note, the best cut-off does not fluctuate when n_{mutant} = 1 and n_{BRCA1} = 1 (**G**), which results in a probability of pathogenicity equal to 0.5 for both the pathogenic and neutral variants. Moreover, using the standard method, when n_{mutant} = 9, the classification of the neutral mutations is class 2 (**E**, left panel), which explains the lack of specificity frequently observed in S13-S19 Figs.
