# Supplementary material for: Functional Assessment of Genetic Variants with Outcomes Adapted to Clinical Decision-Making
Source: PLoS Genet. 2016 Jun 6;12(6):e1006096. doi: 10.1371/journal.pgen.1006096 (PMC4894565; doi:10.1371/journal.pgen.1006096)
Supplement: S19 Fig — See S13 Fig for details. Distribution ranges were modulated so that medians remained the same, as shown in S8 Table. The range factor r, indicated on the graphs, illustrates the relative dispersion of the distributions. When r = 0, the dispersion is null. (A-D) Examples showing the ranges of the mutant and BRCA1 distributions tested, with best cut-off fluctuation results. (E-G) Probabilities of pathogenicity obtained for the neutral (blue line) and pathogenic variants (red line), following a range decrease of the mutant distributions (E), BRCA1 distribution (F), or both (G). As summarized in S9 Table, these results indicate that the probability system of classification is affected mainly when the range of the BRCA1 and mutant distributions is null, whatever method is used. In this situation, the fluctuation of the best cut-off is null and all the mutations are considered as absolutely unknown (probability of pathogenicity equal to 0.5). (PDF) [file pgen.1006096.s021.pdf]

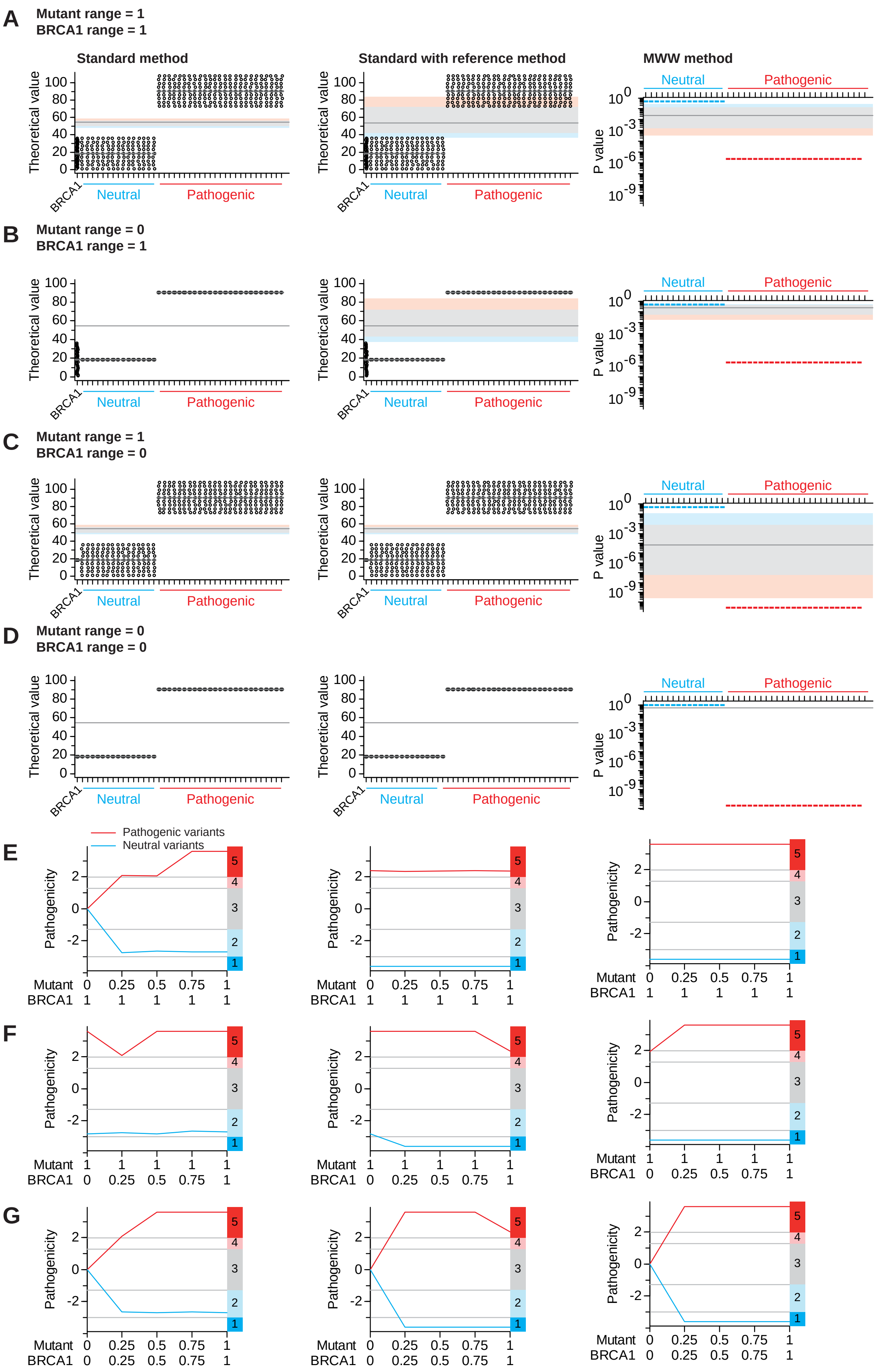

S19 Fig. Effect of the range of mutant and BRCA1 distributions on the probability system of classification (theoretical situation)
See S13 Fig for details. Distribution ranges were modulated so that medians remained the same, as shown in S8 Table. The range factor r, indicated on the graphs, illustrates the relative dispersion of the distributions. When r = 0, the dispersion is null.
(**A-D**) Examples showing the ranges of the mutant and BRCA1 distributions tested, with best cut-off fluctuation results.
(**E-G**) Probabilities of pathogenicity obtained for the neutral (blue line) and pathogenic variants (red line), following a range decrease of the mutant distributions (**E**), BRCA1 distribution (**F**), or both (**G**).
As summarized in S9 Table, these results indicate that the probability system of classification is affected mainly when the range of the BRCA1 and mutant distributions is null, whatever method is used. In this situation, the fluctuation of the best cut-off is null and all the mutations are considered as absolutely unknown (probability of pathogenicity equal to 0.5).
