# Supplementary material for: Functional Assessment of Genetic Variants with Outcomes Adapted to Clinical Decision-Making
Source: PLoS Genet. 2016 Jun 6;12(6):e1006096. doi: 10.1371/journal.pgen.1006096 (PMC4894565; doi:10.1371/journal.pgen.1006096)
Supplement: S20 Fig — After 4 hours of BRCA1 expression, lysates of 6 x 10^6^ cells were examined for the presence of the protein (theoretical size: 200 kDa) with an anti-BRCA1 antibody. Tubulin or Actin was used as a loading control and was probed using an anti-Tubulin or anti-Actin antibody on the same membrane after stripping the first labeling. Signal intensities of full lanes, relatively to the BRCA1 lane, are indicated below. Of note, protein levels three times higher than the WT BRCA1 protein level (normalized to 1) systematically correspond to pathogenic mutations. (A) BRCA1 (Colony Size and Liquid Medium assays). (B) BRCA1-mCherry (Spot Formation and Yeast Localization assays). (C-G) Dotplot with the Spearman coefficient of correlation indicated. Pathogenic and neutral mutations, as well as the WT BRCA1 reference, are represented by a red, blue or black dot, respectively. (C) Correlation between the relative signal intensities of A and B. (D-E) Correlation between the relative signal intensities of A and medians of the Colony Size or Liquid Medium assay. (F-G), correlation between the relative signal intensities of B and medians of the Spot Formation or Yeast Localization assay. (PDF) [file pgen.1006096.s022.pdf]

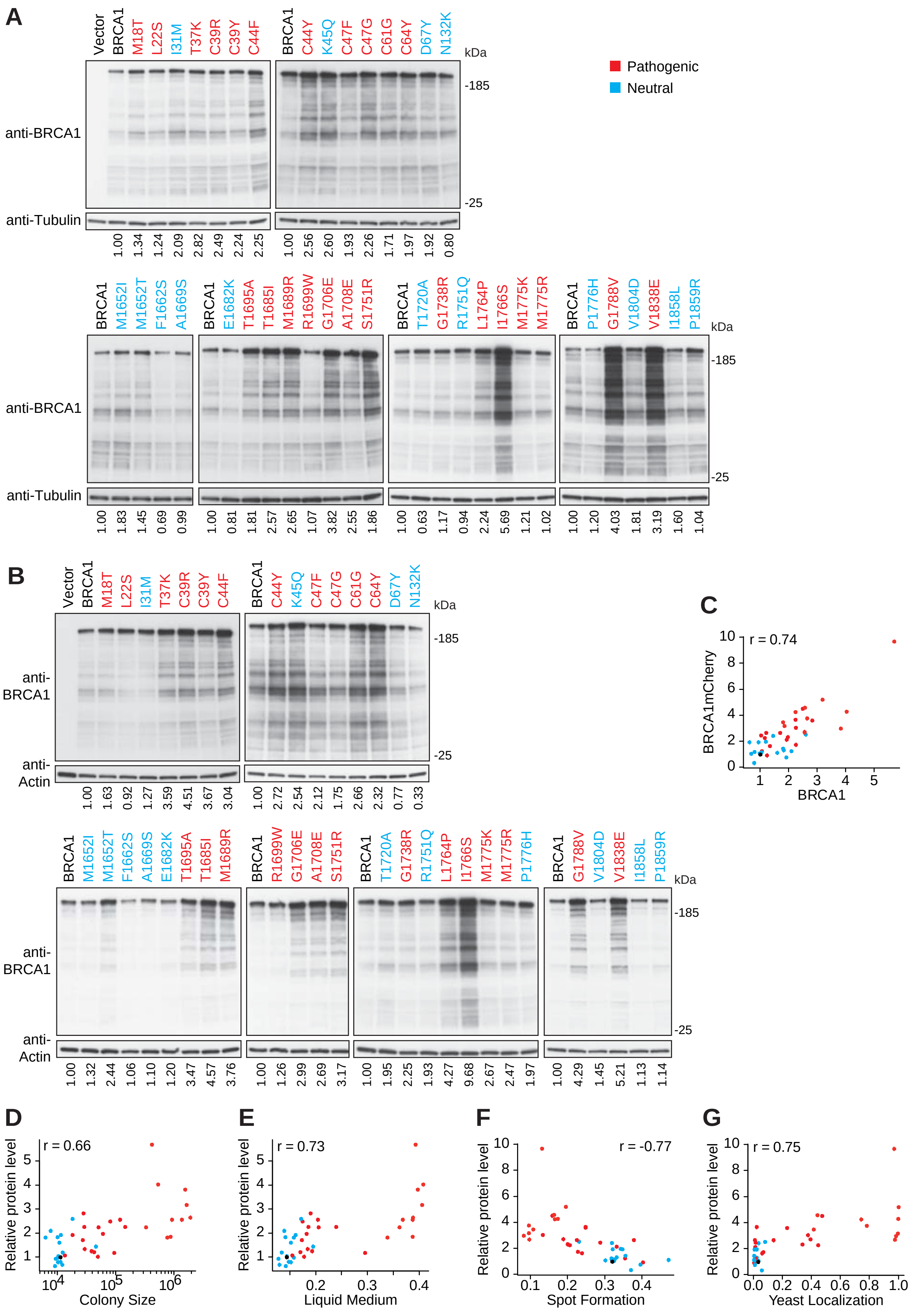

S20 Fig. Western blot analysis

After 4 hours of BRCA1 expression, lysates of 6 x 10^6 cells were examined for the presence of the protein (theoretical size: 200 kDa) with an anti-BRCA1 antibody. Tubulin or Actin was used as a loading control and was probed using an anti-Tubulin or anti-Actin antibody on the same membrane after stripping the first labeling. Signal intensities of full lanes, relatively to the BRCA1 lane, are indicated below. Of note, protein levels three times higher than the WT BRCA1 protein level (normalized to 1) systematically correspond to pathogenic mutations.

(**A**) BRCA1 (Colony Size and Liquid Medium assays).

(**B**) BRCA1-mCherry (Spot Formation and Yeast Localization assays).

(**C-G**) Dotplot with the Spearman coefficient of correlation indicated. Pathogenic and neutral mutations, as well as the WT BRCA1 reference, are represented by a red, blue or black dot, respectively.

(**C**) Correlation between the relative signal intensities of **A** and **B**.

(**D-E**) Correlation between the relative signal intensities of **A** and medians of the Colony Size or Liquid Medium assay.

(**F-G**), correlation between the relative signal intensities of **B** and medians of the Spot Formation or Yeast Localization assay.
