# Supplementary material for: Functional Assessment of Genetic Variants with Outcomes Adapted to Clinical Decision-Making
Source: PLoS Genet. 2016 Jun 6;12(6):e1006096. doi: 10.1371/journal.pgen.1006096 (PMC4894565; doi:10.1371/journal.pgen.1006096)
Supplement: S21 Fig — The theoretical situation was analyzed as follows: one neutral and one pathogenic mutation (nneutral = 1 and npathogenic = 1), with two values per mutant (nmutant = 2, value 1 and 2 for the neutral mutant, and value 3 and 4 for the pathogenic mutant) and two values in the WT BRCA1 reference (nBRCA1 = 2, value 1 and 2). (A) The graphs depicted are similar to those in Fig 1, except that boxplots are replaced by dotplots with median of the distributions indicated by a grey segment. The black horizontal line represents the experimental best cut-off. The best cut fluctuations (colored areas) are not represented but quantiles are shown in F. (B) Table recapitulating all of the possible results when sampling 2 values, with replacement, among the 2 neutral, 2 pathogenic and 2 WT BRCA1 values. Each row is a different combination that provides a best cut-off value, for each method used. The framed row highlights the combination identical to the experimental situation in A. In this simple situation (1 neutral and 1 pathogenic variant), the best cut off computed, in each row, is the median of the two variant medians (standard method), the median of the two variant medians divided by the WT BRCA1 median (standard with reference method) and the median of the two variant p values (MWW method). (C-E) Variant classification using the probability system, with the standard (C), standard with reference (D) and MWW (E) methods, as in S6B Fig. Colored numbers in the table correspond to the different probabilities of pathogenicity designed by the model. The color code respects the five-class nomenclature depicted in S1 Table: grey, class 3; light blue, class 2; pink, class 4. Positions of the neutral and pathogenic variants are represented by a blue and red arrow, respectively. The number below each arrow designates the variant value used in the probability system to attribute the probability of pathogenicity, which corresponds to the median, median divided by the WT reference median or p [file pgen.1006096.s023.pdf]

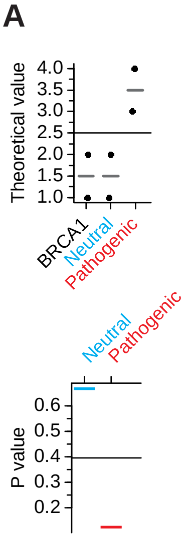

B

Sampling possibility n°	WT BRCA1 reference Value 1	WT BRCA1 reference Value 2	Neutral variant Value 1	Neutral variant Value 2	Neutral variant Median	Neutral variant P value	Pathogenic variant Value 1	Pathogenic variant Value 2	Pathogenic variant Median	Pathogenic variant P value	Best cut-off value: Standard method	Best cut-off value: Standard with reference method	Best cut-off value: MWW method
1	1	1	1	1	1.0	1.000	3	3	3.0	0.097	2.00	2.000	0.54848
2	1	1	1	1	1.0	1.000	3	4	3.5	0.110	2.25	2.250	0.55517
3	1	1	1	1	1.0	1.000	4	4	4.0	0.097	2.50	2.500	0.54848
4	1	1	1	2	1.5	0.309	3	3	3.0	0.097	2.25	2.250	0.20275
5	1	1	1	2	1.5	0.309	3	4	3.5	0.110	2.50	2.500	0.20944
6	1	1	1	2	1.5	0.309	4	4	4.0	0.097	2.75	2.750	0.20275
7	1	1	2	2	2.0	0.097	3	3	3.0	0.097	2.50	2.500	0.09697
8	1	1	2	2	2.0	0.097	3	4	3.5	0.110	2.75	2.750	0.10365
9	1	1	2	2	2.0	0.097	4	4	4.0	0.097	3.00	3.000	0.09697
10	1	2	1	1	1.0	0.933	3	3	3.0	0.110	2.00	1.333	0.52176
11	1	2	1	1	1.0	0.933	3	4	3.5	0.123	2.25	1.500	0.52792
12	1	2	1	1	1.0	0.933	4	4	4.0	0.110	2.50	1.667	0.52176
13	1	2	1	2	1.5	0.667	3	3	3.0	0.110	2.25	1.500	0.38892
14	1	2	1	2	1.5	0.667	3	4	3.5	0.123	2.50	1.667	0.39507
15	1	2	1	2	1.5	0.667	4	4	4.0	0.110	2.75	1.833	0.38892
16	1	2	2	2	2.0	0.309	3	3	3.0	0.110	2.50	1.667	0.20944
17	1	2	2	2	2.0	0.309	3	4	3.5	0.123	2.75	1.833	0.21559
18	1	2	2	2	2.0	0.309	4	4	4.0	0.110	3.00	2.000	0.20944
19	2	2	1	1	1.0	0.985	3	3	3.0	0.097	2.00	1.000	0.54089
20	2	2	1	1	1.0	0.985	3	4	3.5	0.110	2.25	1.125	0.54757
21	2	2	1	1	1.0	0.985	4	4	4.0	0.097	2.50	1.250	0.54089
22	2	2	1	2	1.5	0.933	3	3	3.0	0.097	2.25	1.125	0.51508
23	2	2	1	2	1.5	0.933	3	4	3.5	0.110	2.50	1.250	0.52176
24	2	2	1	2	1.5	0.933	4	4	4.0	0.097	2.75	1.375	0.51508
25	2	2	2	2	2.0	1.000	3	3	3.0	0.097	2.50	1.250	0.54848
26	2	2	2	2	2.0	1.000	3	4	3.5	0.110	2.75	1.375	0.55517
27	2	2	2	2	2.0	1.000	4	4	4.0	0.097	3.00	1.500	0.54848

C Probability system & standard method

Different best cut-off value	2	2.25	2.5	2.75	3
Repeat	3	6	9	6	3
Probability	0.11	0.22	0.33	0.22	0.11
CDF1	0.11	0.33	0.67	0.89	1.00
CDF2	0.00	0.11	0.33	0.67	0.89
Average CDF	0.06	0.22	0.50	0.78	0.94

1.5 3.5

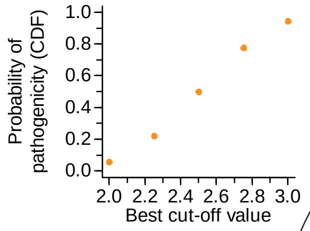

D Probability system & standard with reference method

Different best cut-off value	1.000	1.125	1.250	1.333	1.375	1.500	1.667	1.833	2.000	2.250	2.500	2.750	3.000
Repeat	1	2	3	1	2	3	3	2	2	2	3	2	1
Probability	0.04	0.07	0.11	0.04	0.07	0.11	0.11	0.07	0.07	0.07	0.11	0.07	0.04
CDF1	0.04	0.11	0.22	0.26	0.33	0.44	0.56	0.63	0.70	0.78	0.89	0.96	1.00
CDF2	0.00	0.04	0.11	0.22	0.26	0.33	0.44	0.56	0.63	0.70	0.78	0.89	0.96
Average CDF	0.02	0.07	0.17	0.24	0.30	0.39	0.50	0.59	0.67	0.74	0.83	0.93	0.98

1 2.33

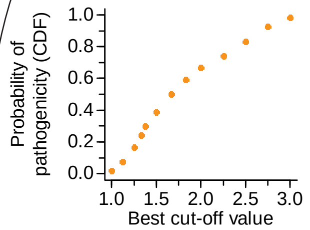

E Probability system & MWW method

Different best cut-off value	0.5552	0.5485	0.5476	0.5409	0.5279	0.5218	0.5151	0.3951	0.3889	0.2156	0.2094	0.2028	0.1037	0.0970
Repeat	2	4	1	2	1	3	2	1	2	1	3	2	1	2
Probability	0.07	0.15	0.04	0.07	0.04	0.11	0.07	0.04	0.07	0.04	0.11	0.07	0.04	0.07
CDF1	0.07	0.22	0.26	0.33	0.37	0.48	0.56	0.59	0.67	0.70	0.81	0.89	0.93	1.00
CDF2	0.00	0.07	0.22	0.26	0.33	0.37	0.48	0.56	0.59	0.67	0.70	0.81	0.89	0.93
Average CDF	0.04	0.15	0.24	0.30	0.35	0.43	0.52	0.57	0.63	0.69	0.76	0.85	0.91	0.96

0.667 0.123

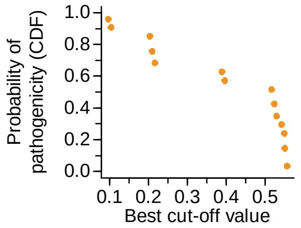

F Quantile system

	Standard method				Standard with reference method				MWW method			
Quantile	0.99	0.95	0.05	0.001	0.99	0.95	0.05	0.001	0.999	0.95	0.05	0.01
Best cut-off value	3	3	2	2	2.935	2.750	1.125	1.003	0.555	0.553	0.099	0.097

3.5 1.5 2.33 1 0.667 0.123

S21 Fig. Exact probability distribution of the best cut-off in the standard, standard with reference and MWW methods (theoretical situation)

The theoretical situation was analyzed as follows: one neutral and one pathogenic mutation ($n_{neutral} = 1$ and $n_{pathogenic} = 1$), with two values per mutant ($n_{mutant} = 2$, value 1 and 2 for the neutral mutant, and value 3 and 4 for the pathogenic mutant) and two values in the WT BRCA1 reference ($n_{BRCA1} = 2$, value 1 and 2).

(**A**) The graphs depicted are similar to those in Fig 1, except that boxplots are replaced by dotplots with median of the distributions indicated by a grey segment. The black horizontal line represents the experimental best cut-off. The best cut fluctuations (colored areas) are not represented but quantiles are shown in **F**.

(**B**) Table recapitulating all of the possible results when sampling 2 values, with replacement, among the 2 neutral, 2 pathogenic and 2 WT BRCA1 values. Each row is a different combination that provides a best cut-off value, for each method used. The framed row highlights the combination identical to the experimental situation in **A**. In this simple situation (1 neutral and 1 pathogenic variant), the best cut off computed, in each row, is the median of the two variant medians (standard method), the median of the two variant medians divided by the WT BRCA1 median (standard with reference method) and the median of the two variant p values (MWW method).

(**C-E**) Variant classification using the probability system, with the standard (**C**), standard with reference (**D**) and MWW (**E**) methods, as in S6B Fig. Colored numbers in the table correspond to the different probabilities of pathogenicity designed by the model. The color code respects the five-class nomenclature depicted in S1 Table: grey, class 3; light blue, class 2; pink, class 4. Positions of the neutral and pathogenic variants are represented by a blue and red arrow, respectively. The number below each arrow designates the variant value used in the probability system to attribute the probability of pathogenicity, which corresponds to the median, median divided by the WT reference median or p value, indicated in the framed row of **B**. For instance, in the standard method (**C**), the pathogenic variant, with a median of 3.5, has the probability 0.94 (class 3).

(**F**) Variant classification using the quantile system. Quantiles were computed from the 27 best cut-off values from **B**, for each method. The colored background defines the intervals within the best cut-off distribution, as explained in S23 Fig. Arrows depict the position of the neutral and pathogenic variants, as in **C-E**.
