# Supplementary material for: Functional Assessment of Genetic Variants with Outcomes Adapted to Clinical Decision-Making
Source: PLoS Genet. 2016 Jun 6;12(6):e1006096. doi: 10.1371/journal.pgen.1006096 (PMC4894565; doi:10.1371/journal.pgen.1006096)
Supplement: S22 Fig — (A) Schematic of the exact best cut-off distribution influenced by different parameters, assuming no ties. The number of neutral (nneutral) and pathogenic (npathogenic) variants influence the exact best cut-off distribution only if the number of values per mutant (nmutant) is above 1. The number of values in the WT reference (nBRCA1) does not influence the exact best cut-off distribution in the standard method, only in the standard with reference and MWW methods. (B) Schematic of the approximate best cut-off distribution influenced by the number of bootstraps performed. Importantly, a single bootstrap (nbootstrap = 1) does not lead to the experimental best cut-off, except if nbest exact = 1. (C) Accuracy of the probability and quantile systems of classification. The schematic illustration is valid, using either the exact or approximate best cut-off distribution. (D) Correcting factor fcor used in the probability system of classification, depending on the parameter a (see the S1 Text). fcor = (nneutral + npathogenic) / (nneutral + npathogenic + a). The framed value (a = 2) was the value used in S13–S15 Tables. (PDF) [file pgen.1006096.s024.pdf]

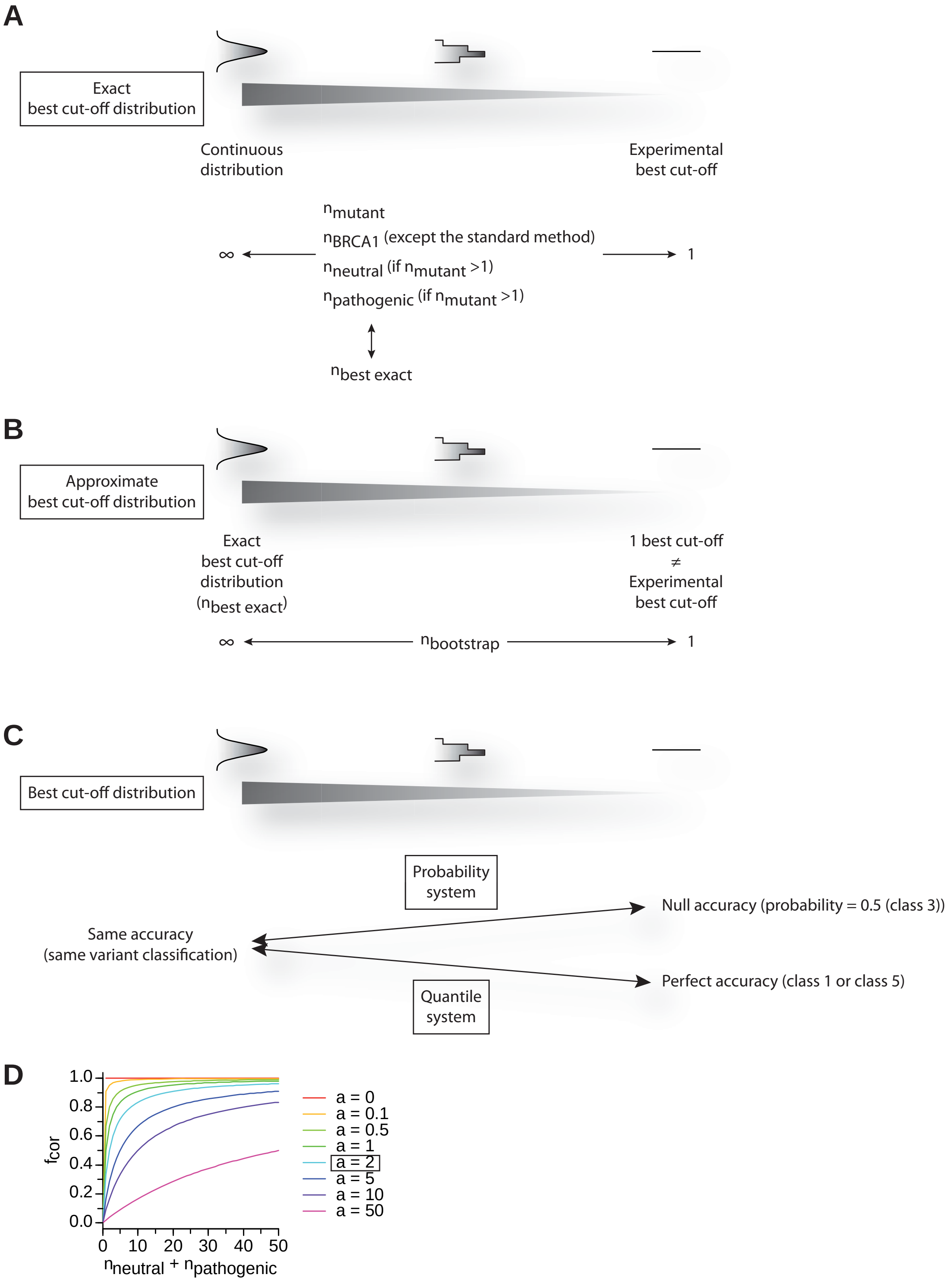

S22 Fig. Additional information about the classification model

(**A**) Schematic of the exact best cut-off distribution influenced by different parameters, assuming no ties. The number of neutral ($n_{neutral}$) and pathogenic ($n_{pathogenic}$) variants influence the exact best cut-off distribution only if the number of values per mutant (n_{mutant}) is above 1. The number of values in the WT reference (n_{BRCA1}) does not influence the exact best cut-off distribution in the standard method, only in the standard with reference and MWW methods.

(**B**) Schematic of the approximate best cut-off distribution influenced by the number of bootstraps performed. Importantly, a single bootstrap ($n_{bootstrap} = 1$) does not lead to the experimental best cut-off, except if $n_{best\ exact} = 1$.

(**C**) Accuracy of the probability and quantile systems of classification. The schematic illustration is valid, using either the exact or approximate best cut-off distribution.

(**D**) Correcting factor fcor used in the probability system of classification, depending on the parameter a (see the S1 Text). $f_{cor} = (n_{neutral} + n_{pathogenic}) / (n_{neutral} + n_{pathogenic} + a)$. The framed value (a = 2) was the value used in S13-S15 Tables.
