# Supplementary material for: Functional Assessment of Genetic Variants with Outcomes Adapted to Clinical Decision-Making
Source: PLoS Genet. 2016 Jun 6;12(6):e1006096. doi: 10.1371/journal.pgen.1006096 (PMC4894565; doi:10.1371/journal.pgen.1006096)
Supplement: S23 Fig — (A) The left figure depicts a theoretical waterfall distribution of pathogenic and neutral missense mutations, as in Fig 1A. (1) Variant classification according to the experimental best cut-off. This cut-off (horizontal black line), that maximizes the experimental sensitivity and specificity in the waterfall distribution, is obtained by ROC curve analysis, as in S2 Fig. In the case of the Colony Size assay, mutations above the best cut-off are classified as pathogenic and mutations below are classified as neutral. (2) Bootstrap analysis provides a fluctuation of the best cut-off, depending on the values of the mutations and the WT BRCA1 reference randomly chosen. The fluctuating best cut-off values form a distribution, as depicted in the schematic. (3) Quantile system of variant classification according to the fluctuation of the best cut-off. The reasoning is the following: the distribution of the fluctuating best cut-off defines quantiles (Q) that delimit the probability of the presence of this variable. As an example, the quantile Q0.99 is the value that separates the 99% lowest values from the 1% highest values in a distribution. This means that the probability to have the best cut-off above the quantile Q0.99 is 1%. Thus, in the Colony Size assay using the standard method, a mutation with the median above the quantile Q0.99 can be considered as pathogenic with a 1% probability of error. Indeed, this mutation could be neutral, but only if the best cut-off is above the median, which has a 1% probability, or less, to occur. This reasoning allows separation of the best cut-off distribution into 5 intervals, based on the five-class nomenclature proposed by Plon et al [26], with each interval defining the probability of the best cut-off presence within the waterfall distribution. (B) Quantiles that delimit the 5 intervals of classification according to the assay and the method used. CS, Colony Size; LM, Liquid Medium; SF, Spot Formation; YL, Yeast Localization assay. [file pgen.1006096.s025.pdf]

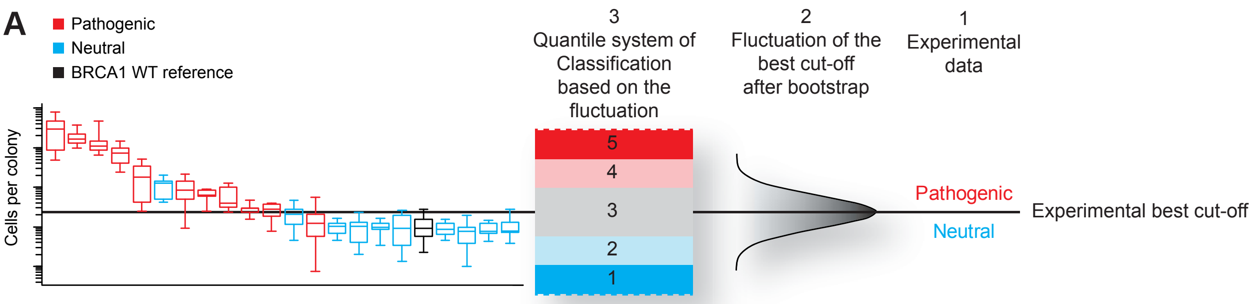

B

Class		Quantile in the 2 standard methods				Quantile in the MWW method			
		CS	LM	SF	YL	CS	LM	SF	YL
Definitely pathogenic	5	Q0.990	Q0.990	Q0.010	Q0.990	Q0.010	Q0.010	Q0.010	Q0.010
Likely pathogenic	4	Q0.950	Q0.950	Q0.050	Q0.950	Q0.050	Q0.050	Q0.050	Q0.050
Uncertain	3	Q0.050	Q0.050	Q0.950	Q0.050	Q0.950	Q0.950	Q0.950	Q0.950
Likely neutral	2	Q0.001	Q0.001	Q0.999	Q0.001	Q0.999	Q0.999	Q0.999	Q0.999
Definitely neutral	1								

C **Colony Size assay**
Standard methods

Class	Probability	Corresponding cut-off values (standard method)		Corresponding cut-off values (standard with reference method)		
		Quantile	Cut-off value (cells per colony)	Quantile	Cut-off value (BRCA1 median fold)	Cut-off value (cells per colony)
5	P(X > Q0.99) = 1%	Q0.99	26,222	Q0.99	2.416	27,062
4	P(Q0.95 ≤ X ≤ Q0.99) = 4%	Q0.95	24,728	Q0.95	2.236	25,047
3	P(Q0.05 ≤ X < Q0.95) = 95%	Q0.05	13,200	Q0.05	1.173	13,143
2	P(Q0.001 ≤ X < Q0.05) = 4.9%	Q0.001	12,133	Q0.001	0.960	10,754
1	P(X < Q0.001) = 0.1%					

x 11,200

D **Colony Size assay**
MWW method

Class	Probability	Corresponding cut-off values	
		Quantile	Cut-off value (p value)
5	P(X < Q0.01) = 1%	Q0.01	5.49e-5
4	P(Q0.01 ≤ X ≤ Q0.05) = 4%	Q0.05	0.00023
3	P(Q0.05 < X ≤ Q0.95) = 95%	Q0.95	0.152
2	P(Q0.95 < X ≤ Q0.999) = 4.9%	Q0.999	0.313
1	P(X > Q0.999) = 0.1%		

Figure S23. Description of the quantile system of classification

(**A**) The left figure depicts a theoretical waterfall distribution of pathogenic and neutral missense mutations, as in Fig 1A. (1) Variant classification according to the experimental best cut-off. This cut-off (horizontal black line), that maximizes the experimental sensitivity and specificity in the waterfall distribution, is obtained by ROC curve analysis, as in S2 Fig. In the case of the Colony Size assay, mutations above the best cut-off are classified as pathogenic and mutations below are classified as neutral. (2) Bootstrap analysis provides a fluctuation of the best cut-off, depending on the values of the mutations and the WT BRCA1 reference randomly chosen. The fluctuating best cut-off values form a distribution, as depicted in the schematic. (3) Quantile system of variant classification according to the fluctuation of the best cut-off. The reasoning is the following: the distribution of the fluctuating best cut-off defines quantiles (Q) that delimit the probability of the presence of this variable. As an example, the quantile Q0.99 is the value that separates the 99% lowest values from the 1% highest values in a distribution. This means that the probability to have the best cut-off above the quantile Q0.99 is 1%. Thus, in the Colony Size assay using the standard method, a mutation with the median above the quantile Q0.99 can be considered as pathogenic with a 1% probability of error. Indeed, this mutation could be neutral, but only if the best cut-off is above the median, which has a 1% probability, or less, to occur. This reasoning allows separation of the best cut-off distribution into 5 intervals, based on the five-class nomenclature proposed by Plon et al, with each interval defining the probability of the best cut-off presence within the waterfall distribution.

(**B**) Quantiles that delimit the 5 intervals of classification according to the assay and the method used. CS, Colony Size; LM, Liquid Medium; SF, Spot Formation; YL, Yeast Localization assay. Note that the quantiles differ, depending on whether the pathogenic mutations are above or below the best cut-off. For instance, in the standard method, the quantiles of the Colony Size assay are Q0.99, Q0.95, Q0.05 and Q0.001 (pathogenic mutants above the best cut-off), while quantiles are Q0.01, Q0.05, Q0.95 and Q0.999 in the Spot Formation assay (pathogenic mutants below the best cut-off). However, these two cases generate the same intervals (e.g., probability 1% for the class 5, see **C** and **D**). Cut-off values corresponding to these quantiles are listed in S5 Table for each assay and for each method.

(**C**) Interval limits in the case of the Colony Size assay, using the standard or the standard with reference method. P(X > Q0.99) = 1% is the probability to obtain the best cut-off variable X strictly over the quantile Q0.99, shown here as 26,222 cells per colony for the standard method, and 2.416 x 11,200 (BRCA1 median of the experimental data) = 27,062 cells per colony for the standard with reference method

(**D**) Interval limits in the case of the Colony Size assay, using the MWW method.
