# Supplementary material for: Functional Assessment of Genetic Variants with Outcomes Adapted to Clinical Decision-Making
Source: PLoS Genet. 2016 Jun 6;12(6):e1006096. doi: 10.1371/journal.pgen.1006096 (PMC4894565; doi:10.1371/journal.pgen.1006096)
Supplement: S24 Fig — Names in red and in blue indicate the pathogenic and neutral mutations, respectively, according to their prior classification. See also S16 Table. The black frames pinpoint the divergent classification compared to that in the probability system (Fig 2B). (PDF) [file pgen.1006096.s026.pdf]

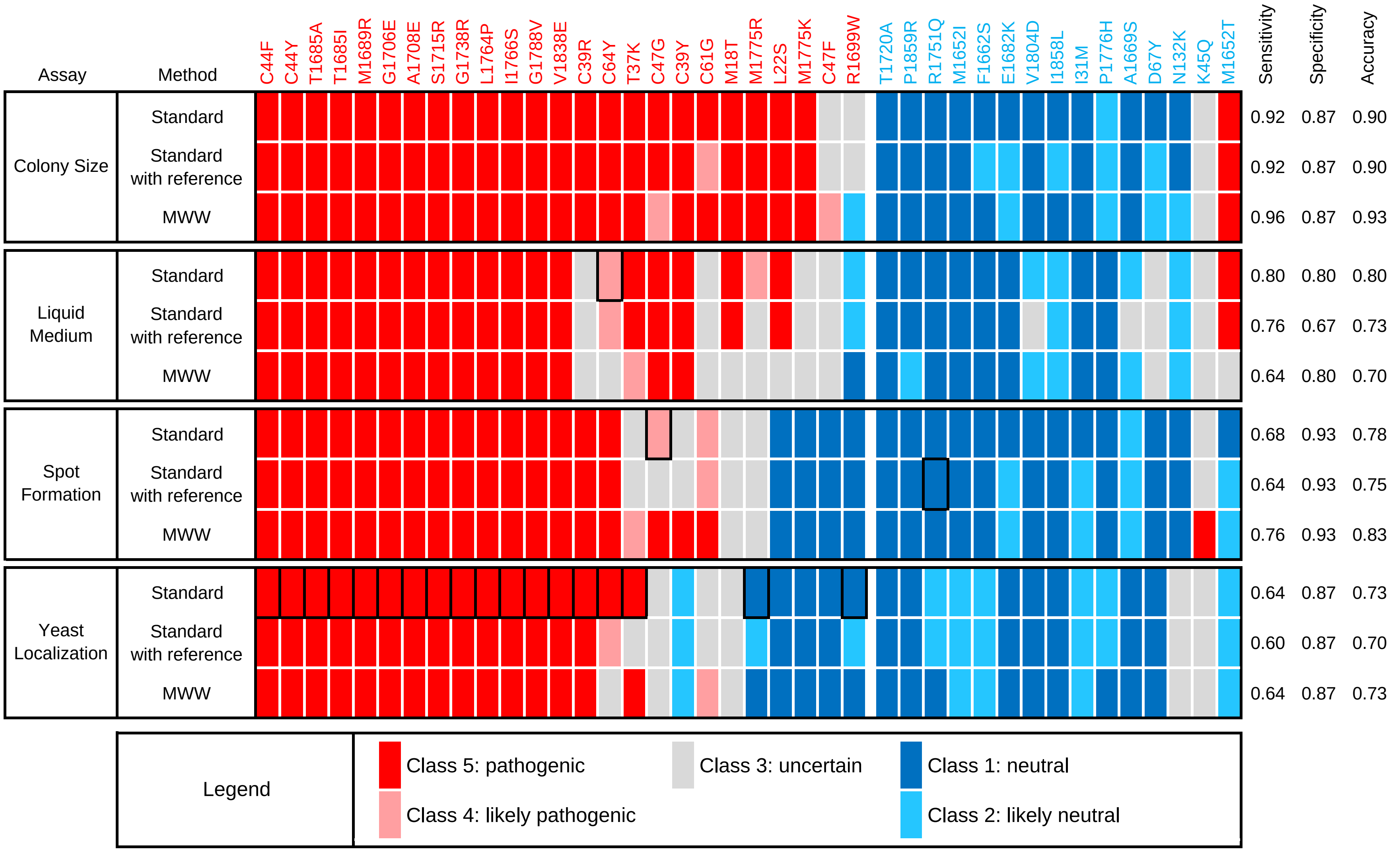

S24 Fig. Variant classification using the quantile system

Names in red and in blue indicate the pathogenic and neutral mutations, respectively, according to their prior classification. See also S16 Table. The black frames pinpoint the divergent classification compared to that in the probability system (Fig 2B).
