# Supplementary material for: Functional Assessment of Genetic Variants with Outcomes Adapted to Clinical Decision-Making
Source: PLoS Genet. 2016 Jun 6;12(6):e1006096. doi: 10.1371/journal.pgen.1006096 (PMC4894565; doi:10.1371/journal.pgen.1006096)
Supplement: S25 Fig — Effect of different experimental parameters was assessed in theoretical situations, exactly as for the probability system of classification, meaning that the best cut-off fluctuations depicted were those used in S13–S19 Figs. Red line, position of the median or p value of the pathogenic mutants; blue line, position of the median or p value of the neutral mutants. The pink, grey and blue areas define intervals within the best cut-off distribution, as explained in S23 Fig. For clarity, the extreme red and blue areas were not displayed. Sensitivity of the quantile system is maximal when the red line is beyond the pink area. Specificity is maximal when the blue line is beyond the light blue area. Accuracy is maximal when sensitivity and specificity are maximal. Finally, sensitivity, specificity and accuracy of the quantile system are null when both lines are in the grey area, or in the wrong side of the best cut-off fluctuation. (A-D) Evolution of the best cut-off fluctuation depending on either the shift intensity of the pathogenic mutations (A), or the shift intensity of the neutral mutations (B), or the shift intensity of the WT reference (C), or the experimental sensitivity and specificity (D). The corresponding panels, depicted for the probability system of classification, are shown in S13D Fig for A, S14E Fig for B, S15E Fig for C and S16E–S16G Fig for D. As summarized in S17 Table, these results did not reveal any flaws. The quantile system behaves as the probability system, in these situations. (PDF) [file pgen.1006096.s027.pdf]

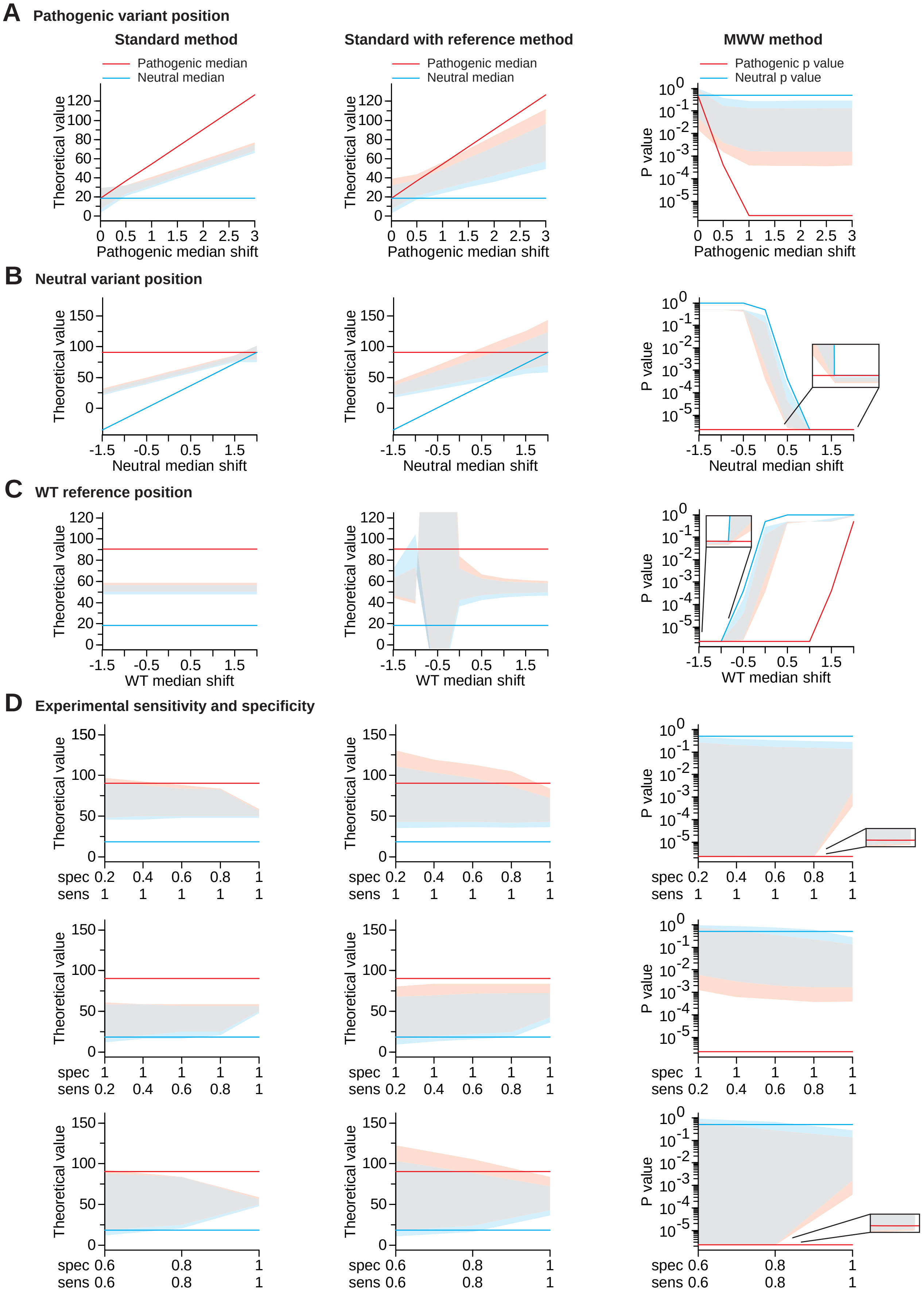

S25 Fig. Quantile system of classification (theoretical situation)

Effect of different experimental parameters was assessed in theoretical situations, exactly as for the probability system of classification, meaning that the best cut-off fluctuations depicted were those used in S13-S19 Figs. Red line, position of the median or p value of the pathogenic mutants; blue line, position of the median or p value of the neutral mutants. The pink, grey and blue areas define intervals within the best cut-off distribution, as explained in S23 Fig. For clarity, the extreme red and blue areas were not displayed. Sensitivity of the quantile system is maximal when the red line is beyond the pink area. Specificity is maximal when the blue line is beyond the light blue area. Accuracy is maximal when sensitivity and specificity are maximal. Finally, sensitivity, specificity and accuracy of the quantile system are null when both lines are in the grey area, or in the wrong side of the best cut-off fluctuation.

(**A**-**D**) Evolution of the best cut-off fluctuation depending on either the shift intensity of the pathogenic mutations (**A**), or the shift intensity of the neutral mutations (**B**), or the shift intensity of the WT reference (**C**), or the experimental sensitivity and specificity (**D**). The corresponding panels, depicted for the probability system of classification, are shown in S13D Fig for **A**, S14E Fig for **B**, S15E Fig for **C** and S16E-G Fig for **D**.

As summarized in S17 Table, these results did not reveal any flaws. The quantile system behaves as the probability system, in these situations.
