# Supplementary material for: Functional Assessment of Genetic Variants with Outcomes Adapted to Clinical Decision-Making
Source: PLoS Genet. 2016 Jun 6;12(6):e1006096. doi: 10.1371/journal.pgen.1006096 (PMC4894565; doi:10.1371/journal.pgen.1006096)
Supplement: S26 Fig — Evolution of the best cut-off fluctuation depending on either the number of neutral and pathogenic mutations (A) or the number of mutant and BRCA1 values (B), as in S25 Fig. The corresponding panels, depicted for the probability system of classification, are shown in S17E–S17G Fig for A and S18E–S18G Fig for B. As summarized in S17 Table, these results reveal a major flaw in the quantile system of classification. Using the standard and MWW methods, the sensitivity and specificity is maximal, regardless of the number of values present within the mutant or BRCA1 distributions (B). Using the standard with reference method, the sensitivity is affected when the number of values in the BRCA1 distribution is decreased, but is maximal when nBRCA1 = 1. Thus, contrary to the probability system, the quantile system is not correctly influenced by the amount of experimental values resulting from functional assessment. (PDF) [file pgen.1006096.s028.pdf]

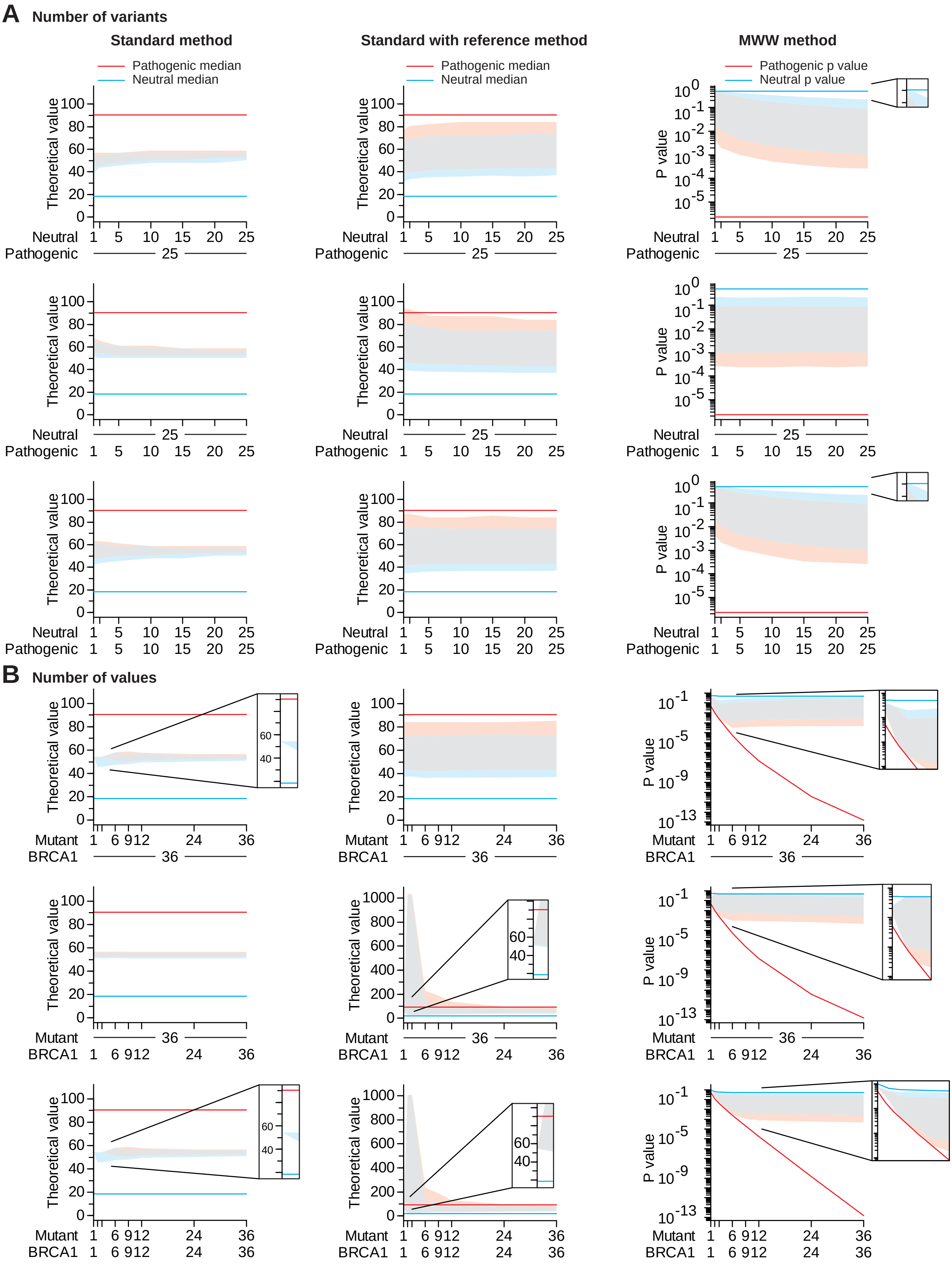

S26 Fig. Quantile system of classification (theoretical situation)

Evolution of the best cut-off fluctuation depending on either the number of neutral and pathogenic mutations (**A**) or the number of mutant and BRCA1 values (**B**), as in S25 Fig. The corresponding panels, depicted for the probability system of classification, are shown in S17E-G Fig for **A** and S18E-G Fig for **B**.

As summarized in S17 Table, these results reveal a major flaw in the quantile system of classification. Using the standard and MWW methods, the sensitivity and specificity is maximal, regardless of the number of values present within the mutant or BRCA1 distributions (**B**). Using the standard with reference method, the sensitivity is affected when the number of values in the BRCA1 distribution is decreased, but is maximal when $n_{BRCA1} = 1$. Thus, contrary to the probability system, the quantile system is not correctly influenced by the amount of experimental values resulting from functional assessment.
