# Supplementary material for: Functional Assessment of Genetic Variants with Outcomes Adapted to Clinical Decision-Making
Source: PLoS Genet. 2016 Jun 6;12(6):e1006096. doi: 10.1371/journal.pgen.1006096 (PMC4894565; doi:10.1371/journal.pgen.1006096)
Supplement: S27 Fig — Evolution of the best cut-off fluctuation, depending on the range of the mutant and BRCA1 distributions, as in S25 Fig. The corresponding panels, depicted for the probability system of classification, are shown in S19E–S19G Fig. As summarized in S17 Table, these results reveal a major flaw in the quantile system of classification. A null range means that all of the values, present in a distribution, are identical (ties). Because ties are related to a low measurement accuracy, an efficient variant classifier should penalize a high number of ties in a dataset, which is not observed here, whatever method is used. (PDF) [file pgen.1006096.s029.pdf]

G Distribution range

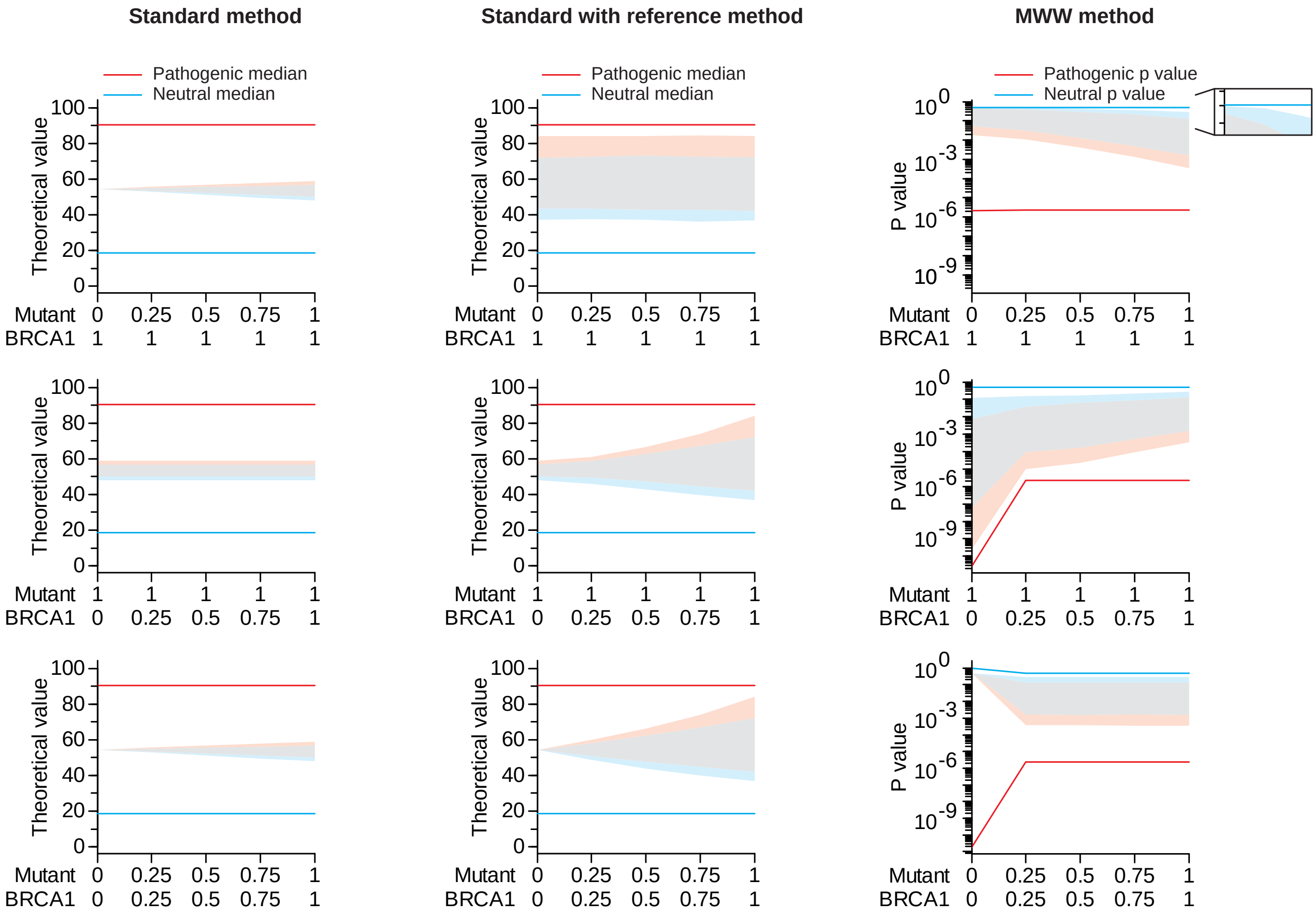

S27 Fig. Quantile system of classification (theoretical situation)
Evolution of the best cut-off fluctuation, depending on the range of the mutant and BRCA1 distributions, as in S25 Fig. The corresponding panels, depicted for the probability system of classification, are shown in S19E-G Fig.
As summarized in S17 Table, these results reveal a major flaw in the quantile system of classification. A null range means that all of the values, present in a distribution, are identical (ties). Because ties are related to a low measurement accuracy, an efficient variant classifier should penalize a high number of ties in a dataset, which is not observed here, whatever method is used.
